# FGFR1/KLB-MFG-E8 Maintains Microvascular Integrity and Adaptive Cardiac Remodeling

**DOI:** 10.1161/CIRCRESAHA.125.327950

**Published:** 2026-09-29

**Authors:** Sanskruti R. Gare, Nasser H. Alatawi, Xinyi Chen, Jiayan Zhang, Claire Ross, Oveena Fonseka, Yihua Han, Namrita Kaur, Andrea Ruiz-Velasco, Xiangjun Zhao, Yingjuan Liu, Riham R.E. Abouleisa, Susanne S. Hille, Tao Wang, Sabu Abraham, Bernard D. Keavney, Martin K. Rutter, Elizabeth J. Cartwright, Gareth Howell, Tamer M.A. Mohamed, Oliver J. Müller, Wei Liu

**Affiliations:** Faculty of Biology, Medicine and Health, The University of Manchester 5292 214021 5292 , United Kingdom (S.R.G., N.H.A., X.C., J.Z., C.R., O.F., Y.H., N.K., A.R.-V., X.Z., Y.L., T.W., S.A., B.D.K., M.K.R., E.J.C., G.H., T.M.A.M., W.L.).; Surgery Department, Baylor College of Medicine 3989 , Houston, TX (R.R.E.A., T.M.A.M.).; Department of Internal Medicine V, University of Kiel, Germany (S.S.H., O.J.M.).; DZHK, German Centre for Cardiovascular Research, Kiel (S.S.H., O.J.M.).; Manchester Heart Centre, Manchester University NHS Foundation Trust, Manchester Academic Health Science Centre, United Kingdom (B.D.K.).; Diabetes, Endocrinology and Metabolism Centre, NIHR Manchester Biomedical Research Centre, Manchester University Hospitals NHS Foundation Trust, Manchester Academic Health Science Centre, United Kingdom (M.K.R.).; Institute of Molecular Cardiology, University of Louisville, KY (T.M.A.M.).

**Keywords:** endothelial cells, fibroblasts, heart failure, luciferases, streptozotocin

## Abstract

**BACKGROUND:**

Endothelial defects in the heart are a contributor to cardiac pathological remodeling and dysfunction, which can be influenced by cardiomyocytes. Here, we explore cardioprotective crosstalk between cardiomyocytes and endothelial cells under diabetic conditions.

**METHODS:**

Type 2 diabetes was induced in male and female mice using a high-fat, high-sucrose diet in conjunction with low-dose streptozotocin. Cardiomyocyte-specific deletion of FGFR1 (fibroblast growth factor receptor 1) or KLB (beta-Klotho) was achieved by crossing floxed mice with αMHC (alpha-myosin heavy chain)-Cre transgenic mice, while the treatment potential was assessed by recombinant adeno-associated virus 9 delivery systems or administration of recombinant protein. Various in vitro models were used to investigate the mechanisms under a diabetes-like condition. The molecular mechanisms were explored through transcriptomics, proteomics, cytokine arrays, angiogenesis assays, histology, and luciferase reporter assays.

**RESULTS:**

Human diabetic hearts exhibited impaired angiogenic pathways with a marked reduction in myocardial capillary density. Multiomics profiling also revealed alterations in the FGFR1 (fibroblast growth factor receptor 1) pathway in mouse diabetic hearts. Both cardiomyocyte-specific FGFR1- and KLB- knockout mice exhibited reduced capillary abundance and developed decompensated cardiac remodeling. Mechanistic studies identified CEBPβ (CCAAT/enhancer binding protein beta) as a downstream transcription factor of the FGFR1/KLB pathway regulating MFG-E8 (milk fat globule-EGF factor 8) expression in cardiomyocytes. Cardiomyocyte-secreted MFG-E8 enhanced endothelial viability and branching. Importantly, the detrimental effects on the heart were rescued by corestoration of cardiac FGFR1 and KLB, whereas neither alone was sufficient, underscoring their synergistic action against diabetic stress. Finally, genetic or pharmacological enhancement of MFG-E8 reversed myocardial capillary rarefaction and led to an improvement in cardiac function, albeit with more pronounced therapeutic effects in males.

**CONCLUSIONS:**

Our findings demonstrate FGFR1/KLB-mediated cardioprotective mechanisms through cardiomyocyte-endothelial cell crosstalk and provide evidence that preserving adaptive remodeling represents a promising strategy to alleviate heart failure in both sexes.

**Novelty and Significance**

**What Is Known?**

- Microvascular impairment is a central feature of diabetes.
- Loss of myocardial capillary networks triggers maladaptive cardiac remodeling and cardiac dysfunction.
- Crosstalk between cardiomyocytes and endothelial cells is essential for preserving vascular integrity and cardiac homeostasis.

**What New Information Does This Article Contribute?**

- Diabetic human hearts and multiple mouse models exhibit marked capillary rarefaction accompanied by impaired angiogenic pathways.
- Cardiomyocyte FGFR1 (fibroblast growth factor receptor 1) and KLB (beta-Klotho) function cooperatively to maintain microvascular integrity and protect against maladaptive cardiac remodeling.
- MFG-E8 (milk fat globule-EGF factor 8) is identified as a cardiomyocyte-derived paracrine downstream effector of FGFR1/KLB and is transcriptionally regulated by CEBPβ (CCAAT/enhancer binding protein beta).
- Enhancement of MFG-E8 restores capillary density and mitigates maladaptive remodeling and heart failure in diabetic mice.

This study provides functional evidence that FGFR1 and KLB synergistically maintain angiogenic capacity and protect the heart from diabetes-induced dysfunction. Loss of either FGFR1 or KLB disrupts cardiomyocyte-endothelial communication by suppressing MFG-E8 expression. Mechanistically, FGFR1 and KLB cooperation activates the transcription factor CEBPβ, which drives MFG-E8 production and enables paracrine support of endothelial survival and angiogenesis. Therapeutic modulation of this FGFR1/KLB-CEBPβ-MFG-E8 axis preserves microvascular integrity and attenuates heart failure progression. These findings reveal an unrecognized mechanism of cardiomyocyte-endothelial interaction that safeguards the diabetic myocardium.

**Meet the First Author, see p e000772**

**Editorial, see Article by Elbatreek and Lefer**

Microvascular dysfunction is recognized as the earliest and critical manifestation of type 2 diabetes (T2DM).^1^ In particular, cardiac microvascular impairment is one of the key factors influencing adaptive cardiac remodeling, ultimately contributing to the development and progression of cardiac dysfunction and heart failure (HF).^2,3^

Impaired microvasculature hinders the supply of oxygen and nutrients to the heart, further accelerating maladaptive cardiac remodeling and HF progression.^4–6^ In the diabetic heart, endothelial cells (ECs) exhibit persistent alterations, which impair angiogenic responses.^7^ Additionally, cardiomyocytes, the most abundant cell type in the heart, play a role in regulating cardiomyocyte EC crosstalk through paracrine signals.^6^ For instance, cardiomyocytes produce and secrete multiple angiogenic mediators. Since these cardiokines trigger signaling cascades that modulate EC proliferation and promote capillary expansion,^8^ the intimate communication between cardiomyocytes and ECs is fundamental to preserving both cardiac and vascular integrity. Despite its clinical importance, the molecular mechanisms that regulate cardiomyocyte-EC interaction in diabetic HF remain incompletely understood. Moreover, effective therapeutic strategies aimed at restoring this intercellular pathway in diabetes-associated cardiac dysfunction are currently lacking.

FGFR1 (fibroblast growth factor receptor 1) plays essential biological roles in various stages of human development and metabolic health.^9^ In the heart, FGFR1 contributes to the maintenance of early cardiac development, cardiomyocyte proliferation, and supports mitochondrial homeostasis.^10–12^ KLB (beta-Klotho) is a member of the membrane-bound klotho family and acts as a coreceptor facilitating the binding of FGF (fibroblast growth factor) ligands (such as FGF19 [fibroblast growth factor 19] and FGF21 [fibroblast growth factor 21]) to FGFRs.^13^ Recent studies have highlighted the important function of KLB in modulating FGFR signaling activation.^14–16^ Our previous in vitro study demonstrated that FGFR1 and KLB in myoblasts facilitate the molecular pathways involved in angiogenesis^17^; however, the functional evidence and underlying mechanisms in the heart remain unexplored.

Emerging evidence demonstrates that MFG-E8 (milk fat globule-epidermal growth factor 8) is a multifunctional glycoprotein involved in key physiological processes, including anti-inflammation, wound healing, and clearance of apoptotic cells.^18–21^ Notably, MFG-E8 also exerts fundamental functions in modulating vasculogenic and angiogenic properties.^22,23^ In the context of cardiac function, MFG-E8 has been shown to confer cardioprotective effects by mitigating hypertrophic growth, fibrosis formation, and proinflammatory responses.^24–26^ However, the regulatory mechanisms governing cardiac MFG-E8 and its roles in the heart in diabetic settings are yet to be investigated.

In this study, we have characterized a cardiomyocyte EC crosstalk mechanism governed by FGFR1 and KLB in cardiomyocytes. We first observed that failing diabetic hearts exhibit impaired angiogenic pathways, concomitant with a reduction in cardiac FGFR1 and KLB expression. Loss of either FGFR1 or KLB in cardiomyocytes aggravates microvascular rarefaction in the heart, contributing to cardiac dysfunction under diabetic conditions. Mechanistically, we identified that MFG-E8 expression in cardiomyocytes is transcriptionally regulated by CEBPβ (CCAAT/enhancer binding protein-beta), which serves as a downstream mediator of the FGFR1/KLB signaling pathway. MFG-E8 acts as a crucial angiogenic factor that promotes EC differentiation and mitigates FGFR1/KLB deficiency-triggered capillary rarefaction and cardiac dysfunction under diabetic stress. Importantly, we determined that the FGFR1 pathway in cardiomyocytes exerts its cardioprotective effects in T2DM, requiring KLB as an indispensable cofactor. In summary, our findings unveil the vital synergistic roles of the FGFR1/KLB signaling pathway in cardiomyocytes in preserving microvasculature and cardiac function under diabetic stress.

## Methods

### Data Availability

All data, experimental materials, and protocols are available from the corresponding author on request for the purpose of replicating procedures. Comprehensive methodological details are provided in the Supplemental Methods, as well as the Major Resources Table in the Supplemental Material.

### Animal Studies

All animal experiments were conducted in accordance with the United Kingdom Animals (Scientific Procedures) Act 1986 and the ARRIVE guidelines. Cardiomyocyte-specific FGFR1 and KLB knockout mice (FGFR1^cKO^ and KLB^cKO^) were generated for the loss-of-function study. Cardiac overexpression of FGFR1 and KLB was achieved via adeno-associated virus (AAV9) gene delivery. To address treatment potential, intraperitoneal injections of recombinant MFG-E8 (40 μg/kg body weight/wk)^27–29^ and AAV9-*CEBPB* were administered. T2DM was induced through a high-fat, high-sucrose diet (HFHSD) combined with 3 consecutive intraperitoneal injections of low-dose streptozotocin.

### Human Samples

Human hearts were obtained ethically from the United Network for Organ Sharing through IIAM and Novabiosis with informed consent from the next of kin.

### Human Induced Pluripotent Stem Cells-Derived Cardiomyocytes

The human induced pluripotent stem cells (hiPSC) 02C9 were generated from adult human dermal fibroblasts using the CytoTune-iPS 2.0 Sendai Reprogramming Kit. The hiPSCs were maintained in mTeSR Plus media and differentiated into cardiomyocytes using 4 μmol/L CHIR99021 and 5 μmol/L IWP2.

### Human Embryonic Stem Cell-Derived Cardiomyocytes

H9 human embryonic stem cells (WA09; WiCell) were differentiated into cardiomyocytes using temporal WNT (wingless-related integration site) modulation with 6 µmol/L CHIR99021 and 2 µmol/L C59.

### Human Umbilical Vein ECs

Human umbilical vein ECs (HUVECs) were cultured in endothelial growth medium supplemented with a low serum supplement mix. HUVEC viability following exposure to neonatal rat cardiomyocytes (NRCMs)-conditioned media was assessed using the CellTiter-Glo Luminescent Cell Viability Assay. HUVEC cytotoxicity was determined using the CellTox Green Cytotoxicity Assay. The paracrine effects of treated cardiomyocytes on endothelial angiogenesis were assessed using a tube formation angiogenesis assay following the manufacturer’s instructions. A 10-plex LEGENDplex angiogenesis assay was conducted to quantify secreted angiogenic factors from HUVECs exposed to hiPSC-cardiomyocytes-derived conditioned media. Gating was applied to differentiate between the 10 beads, and data were analyzed using BioLegend’s LegendPlex Data Analysis Software.

### Statistics

Data are presented as bar/dot plots showing mean±SEM. For data sets with sample sizes ≥6, normality was assessed using the Shapiro-Wilk test. Normally distributed data were analyzed using ordinary 1-way or 2-way ANOVA with appropriate post hoc tests; comparisons between 2 groups were performed using the 2-tailed unpaired Student *t* test. For skewed data or sample sizes <6, nonparametric tests, such as permutation-based analyses, the Kruskal-Wallis test, or the Mann-Whitney *U* test, were used. Statistical analyses were performed using GraphPad Prism version 10 or RStudio (R 4.6.21), and a *P* or *q*<0.05 was considered statistically significant.

## Results

### Disrupted Angiogenic and Cardiac FGFR1/KLB Signaling in the Human and Mouse Diabetic Heart

RNA sequencing followed by gene ontology pathway enrichment analysis (*P*_*adj.*_<0.10) in human diabetic failing hearts revealed that among the altered molecular pathways, those associated with endothelial function and angiogenesis exhibited some of the most pronounced changes and are presented (Figure 1A). Concomitantly, a reduction in CD31^+^ ECs (Figure 1B) indicated microvascular impairment in diabetic failing hearts.

**Figure 1.**
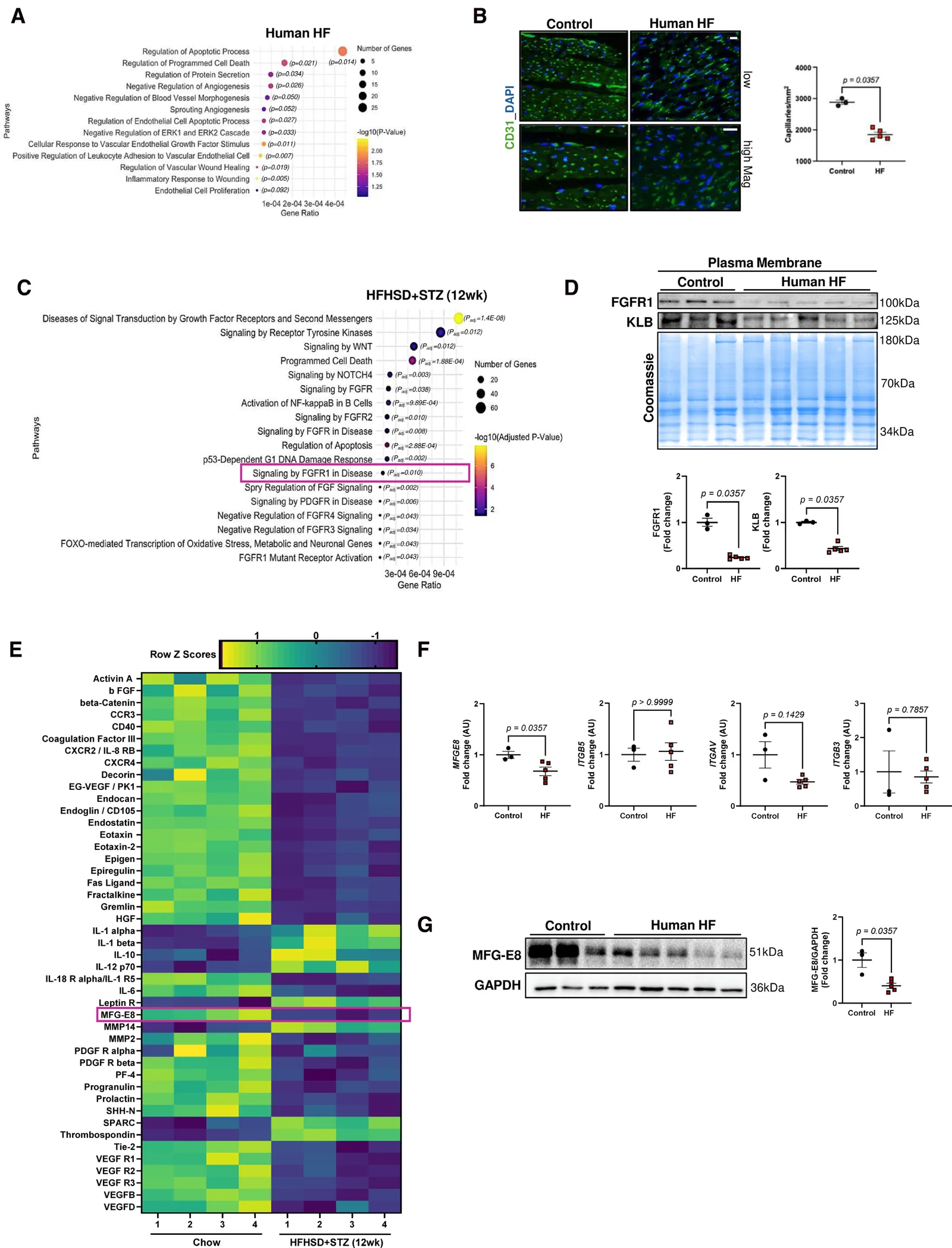
**Cardiac FGFR1 (fibroblast growth factor receptor 1)/KLB (beta-klotho) signaling pathway and angiogenesis are dysregulated in human and mouse diabetic failing hearts. A**, Gene ontology (GO) pathway enrichment analyses of the differentially expressed genes (*P*_*adj*_ <0.1) identified via RNA sequencing in human diabetic heart failure (HF) vs healthy controls (N=3 hearts/control group and N=5 hearts/HF group). Enriched angiogenic pathways (*P*<0.1) are presented in the figure. **B**, Immunofluorescent co-staining with CD31 (green) and DAPI (blue) in human heart tissues with quantification of capillaries per mm^2^ (N=3 hearts/control group and N=5 hearts/HF group; scale =20 µm). **C**, GO pathway enrichment analyses of the differentially expressed genes (*P*_*adj*_ <0.05; absolute Log_2_FC >1) identified via RNA sequencing in mouse hearts subjected to a high-fat, high-sucrose diet (HFHSD) and streptozotocin (STZ) injections for 12 weeks vs chow-fed mice (N=4 mice of both sexes/group). Enriched angiogenic pathways (*P*_*adj*_.<0.05) are presented specifically. **D**, Representative immunoblot and quantification of plasma membrane expression of FGFR1 and KLB in human failing hearts (HF) compared with controls (N=3 hearts/control group and N=5 hearts/HF group). Coomassie blue staining was used to assess total protein as a loading control. **E**, Heatmap of cytokine array analysis showing differentially expressed cytokines (*q*<0.05) in mouse hearts following HFHSD+STZ stress for 12 weeks compared with chow-fed controls (N=4 hearts pooled from 8 hearts from both sexes/group). **F**, Quantitative PCR analysis of *MFGE8*, *ITGB5, ITGAV*, and *ITGB3* in human diabetic failing hearts (N=3 hearts/control group and N=5 hearts/HF group). **G**, Representative immunoblots and quantification of cardiac MFG-E8 protein expression in HF patients (N=3 hearts/control group and N=5 hearts/HF group). GAPDH was used as the loading control. Data are presented as mean±SEM. Statistical significance (*P values* shown in the panel) was assessed using the Mann-Whitney *U* test (**B**, **D**, **F**, **G**) and multiple unpaired Student *t*-tests with false discovery rate (FDR) correction set at 1% (**E**).

To further elucidate the signaling pathways dysregulated in T2DM-associated HF, we performed RNA sequencing on hearts from mice subjected to 12 weeks of HFHSD combined with streptozotocin. Gene ontology pathway enrichment analyses of the 2771 significantly downregulated genes (*P*_*adj*_<0.05; absolute Log_2_FC >1) revealed a marked disruption in the FGFR1 signaling pathway in diabetic hearts (Figure 1C). Consistent with these findings, proteomics followed by gene ontology pathway enrichment analyses in another preclinical diabetic model, induced by a 24-week high-fat diet (HFD), also detected that the cardiac FGFR1 signaling pathway was attenuated under prolonged diabetic stress (Figure S1A). Importantly, the expression of both FGFR1 and its co-receptor KLB was reduced in the myocardial plasma membrane fractions of human diabetic failing hearts (Figure 1D), as well as in mice subjected to 12 weeks of HFHSD+ streptozotocin and 24 weeks of HFD (Figure S1B through S1C).

Therefore, we sought to understand how plasma membrane FGFR1 and KLB are reduced under diabetic stress. Firstly, the transcripts of *FGFR1* and *KLB* in human diabetic hearts were unchanged (Figure S2A), while more ubiquitinated FGFR1 and KLB in hearts (Figure S2B) supported the observed decrease in protein expression. In vitro studies further confirmed that both FGFR1 and KLB were prone to be ubiquitinated (Figure S2C through S2F) and underwent lysosome-dependent degradation under the stress of palmitic acid plus high glucose (HG; Figure S2G). Under physiological conditions, membrane receptors undergo dynamic recycling and reuse. Inefficient recycling, often resulting from postendocytic degradation, leads to diminished receptor abundance at the plasma membrane.^30,31^ Thus, we found that co-localization of FGFR1 and KLB with Rab11b, a marker of efficient recycling endosomes, was reduced (Figure S2H), suggesting impaired recycling of FGFR1 and KLB under prolonged stress. On the contrary, proteasome-dependent degradation also likely occurred (Figure S2I). Both ways reasonably explain their reduction under diabetic stress (Figure 1D; Figure S1B and S1C). Together, these findings indicate that diabetic stress promotes degradation of FGFR1 and KLB in cardiomyocytes, which is likely associated with impaired angiogenesis in T2DM.

Given that enhanced cardiac FGFR1/KLB signaling increases the secretion of proangiogenic cytokines,^17^ we assessed whether impaired cardiac FGFR1/KLB signaling in vivo is accompanied by altered cytokine expression. A cytokine array on diabetic hearts detected a reduction in VEGFs (vascular endothelial growth factors) and their receptors. Importantly, a reduction in the angiogenic mediator, MFG-E8, was seen in diabetic mouse hearts (Figure 1E; Table S1). This observation was further validated in human diabetic failing hearts, where MFG-E8 was significantly downregulated at both transcript (Figure 1F) and protein levels (Figure 1G), although its putative endothelial receptors remained unchanged (Figure 1F). Prompted by consistent observations showing that FGFR1/KLB is correlated with MFG-E8 in vitro,^17^ these findings imply that, in response to diabetic stress, the disrupted cardiac FGFR1/KLB pathway is linked to compromised microvascular integrity, presumably through dysregulation of angiogenic mediators including MFG-E8.

### Cardiomyocyte FGFR1 or KLB Deletion Aggravates Capillary Rarefaction and Cardiac Dysfunction under Diabetic Stress

To gain functional evidence of FGFR1 or KLB loss in cardiomyocytes, cardiomyocyte-specific FGFR1 and KLB knockout mice (FGFR1^cKO^ and KLB^cKO^; Figure S3) and their floxed littermate controls (FGFR1^Flox^ and KLB^Flox^) were subjected to an 8-week regimen of chow or HFHSD feeding combined with streptozotocin injections to induce diabetic stress (Figure 2A). This time point was chosen because endogenous FGFR1 and KLB were unchanged until 12 weeks of HFHSD+ streptozotocin stress (Figure S1A). Both male and female mice developed diabetes, confirmed by comparably elevated fasting blood glucose levels and impaired glucose tolerance (Figure S4) following stress. However, FGFR1 or KLB deficiency exacerbated T2DM-induced cardiac dysfunction, irrespective of sex (Tables S2 and S3; Figure S5). Diastolic function was evidenced by isovolumic relaxation time (Figure 2B and 2F) and the ratio of peak velocity of blood flow from left ventricular relaxation in early diastole (E wave) to that in late diastole (A wave; E/A ratio; Figure 2C and 2G), while systolic function was reflected by ejection fraction (Figure 2D and 2H) and fractional shortening (Figure 2E and 2I). These findings suggest that FGFR1 or KLB deficiency in cardiomyocytes is a causative factor for inducing cardiac dysfunction under diabetic stress.

**Figure 2.**
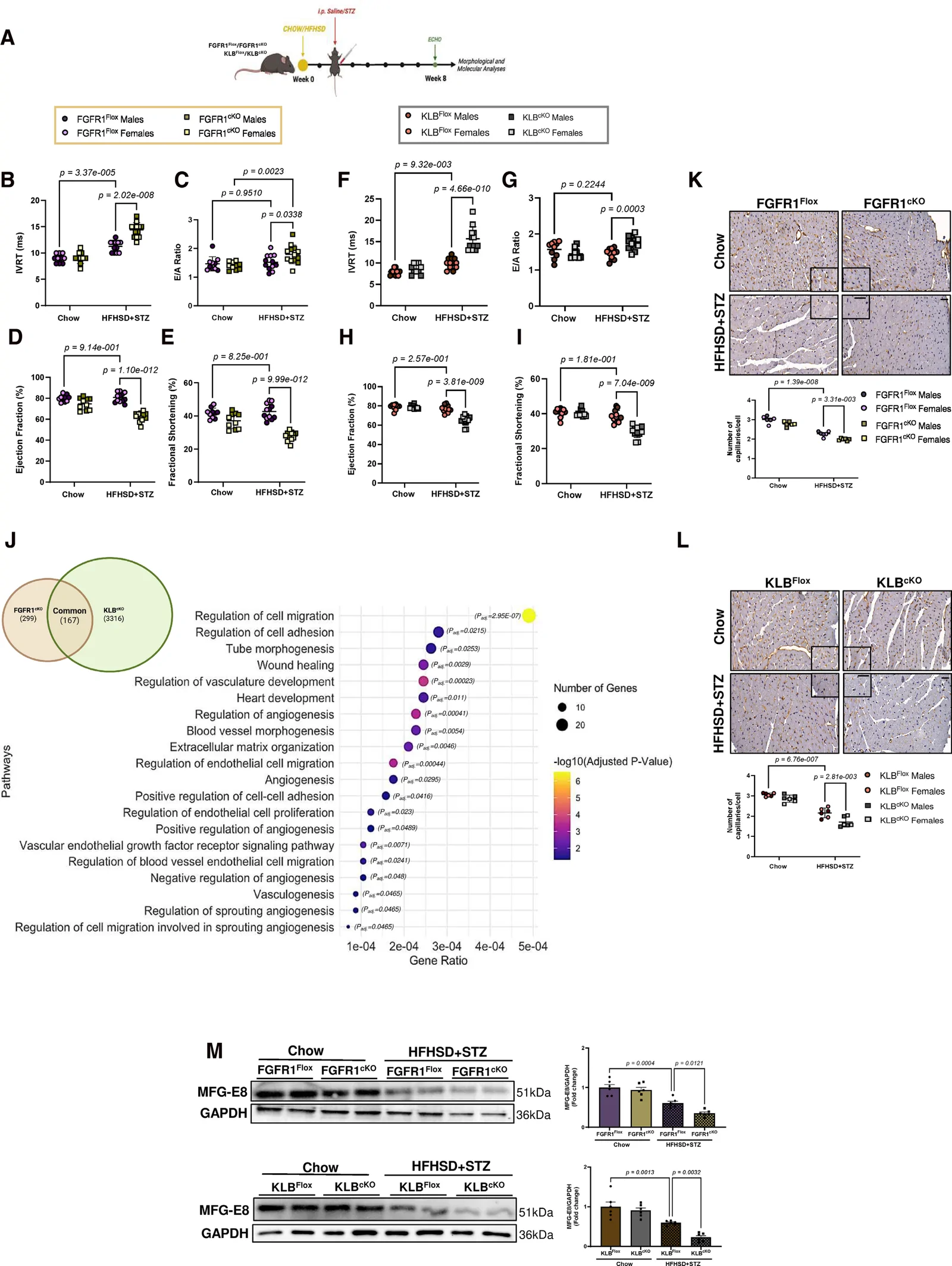
**Cardiomyocyte-specific FGFR1 (fibroblast growth factor receptor 1) or KLB (beta-klotho) deficiency exacerbates cardiovascular impairment under diabetes. A**, Schematic illustration of experimental design. **B** through **E**, Cardiac functional parameters in FGFR1^cKO^ and flox littermates. **B**, Isovolumic relaxation time (IVRT), (**C**) ratio of peak velocity blood flow from left ventricular relaxation in early diastole to that in late diastole (E/A ratio), (**D**) percentage of left ventricular ejection fraction, (**E**) percentage of left ventricular fractional shortening (N=10 mice of both sexes/chow-fed group and N=12–13 mice of both sexes/high-fat, high-sucrose diet plus streptozotocin [HFHSD+STZ] group). **F** through **I**, Cardiac functional parameters in KLB^cKO^ and flox littermates. **F**, Isovolumic relaxation time (IVRT); (**G**) ratio of peak velocity of blood flow from left ventricular relaxation in early diastole to that in late diastole (E/A), (**H**) percentage of left ventricular ejection fraction; (**I**) percentage of left ventricular fractional shortening (N=10 mice of both sexes/chow-fed group and N=12–13 mice of both sexes/HFHSD+STZ-fed group). **J**, Gene ontology (GO) pathway enrichment analyses of 167 commonly differentially expressed genes (*P*_*adj*_.<0.1) identified by RNA sequencing from FGFR1^cKO^ and KLB^cKO^ hearts compared with their flox littermates under HFHSD+STZ stress (N=4 hearts from both sexes/group). Enriched angiogenic pathways (*P*_*adj*_.<0.05) are presented. **K** through **L**, Immunohistochemical staining of CD31 and quantification of capillary density (capillaries/cell) in mice (N=6 hearts from both sexes/group; scale=20 µm). **M**, Representative immunoblots and quantification of cardiac MFG-E8 (milk fat globule-epidermal growth factor 8) protein expression in FGFR1^cKO^ (**top**) and KLB^cKO^ (**bottom**) mice under chow or HFHSD+STZ conditions (N=6 hearts from both sexes/group). GAPDH was used as the loading control. Data are presented as mean±SEM. Statistical significance (*P* values shown in the panel) was assessed using ordinary 2-way ANOVA followed by Šidák multiple comparisons (**B–I**, **K–M**).

To explore the underlying mechanisms, RNA sequencing was performed on hearts from diabetic knockout mice. Among 299 and 3316 differentially expressed genes identified in FGFR1^cKO^ and KLB^cKO^ mice compared with their controls under diabetes, respectively, 167 genes were in common (Table S4). Furthermore, pathway enrichment analyses of these 167 overlapping genes altered due to FGFR1 or KLB disruption revealed dysregulated angiogenic signaling and EC activation pathways (Figure 2J). Noticeably, deletion of FGFR1 or KLB in cardiomyocytes triggered microvascular impairment in both male and female diabetic mice, indicated by reduced CD31^+^ cells in the myocardium (Figure 2K and 2L), more endothelial apoptosis (Figure S6A and S6B), and decreased pericyte density (Figure S6C through S6F), conceivably through paracrine mechanisms. Correspondingly, cardiac MFG-E8 decline in response to diabetic stress was exacerbated in the knockout hearts (Figure 2M), supporting a mechanistic link between cardiac FGFR1/KLB signaling and MFG-E8, mirroring the observations in human diabetic hearts. Nevertheless, MFG-E8 in other organs was unaffected by cardiac FGFR1 or KLB deletion (Figure S6G and S6H).

Additionally, FGFR1 or KLB loss aggravated pathological cardiac remodeling in both sexes under diabetes, as evidenced by increased myocardial fibrosis and apoptosis (Figure S7A through S7D), although cardiomyocyte hypertrophy remained comparable between knockout and control groups under diabetic stress (Figure S7E and S7F). Transcript assessments further demonstrated upregulation of fibrosis and pathological remodeling markers (*Acta1*, *Nppb*, *Col1a2*, *Col3a1*, *Tgfb*) and downregulation of angiogenic genes (*Cdh5*, *Vegfa*) in both diabetic knockout models (Figure S7G and S7H). Together, these findings demonstrate that cardiomyocyte-specific deletion of FGFR1 or KLB exacerbates capillary rarefaction and maladaptive cardiac remodeling under diabetic conditions.

### FGFR1 and KLB Synergize to Preserve Cardiovascular Function Under Diabetic Stress

To assess whether FGFR1 and KLB are simultaneously required for maintaining cardiac function in T2DM, we used FGFR1^cKO^ mice under diabetic stress (as a cardiac and microvascular dysfunction model) to examine the effects of overexpressing cardiac FGFR1 or KLB. To do so, cardiac-specific overexpression of either FGFR1 or KLB using AAV9 vectors driven by the cTnT (cardiac troponin T) promoter (AAV9-*Fgfr1* and AAV9-*Klb*, respectively; Figure S8A through S8D) was employed in FGFR1^cKO^ diabetic mice (Figure 3A). The fasting blood glucose in mice was unchanged with overexpression of either gene (Figure S8E), confirming that the observed cardiac effects were not due to glucose differences. Of note, FGFR1 restoration markedly improved both diastolic (Figure 3B through 3D; Table S5) and systolic cardiac function in FGFR1-deficient male and female mice (Figure 3B, 3E, and 3F; Table S5) after 8-week diabetic stress (endogenous KLB remained). However, KLB overexpression failed to confer similar benefits in the absence of FGFR1 (Figure 3B through 3F; Table S5). Cytokine array analyses revealed that FGFR1 restoration led to a substantial upregulation of angiogenic cytokines, including MFG-E8, in FGFR1-loss hearts compared with the untreated group (AAV9-*Gfp*; Figure 3G; Table S6). Consequently, improved vascular integrity in both sexes (Figure 3H) by FGFR1 reinstatement was accompanied by enhanced MFG-E8 (Figure 3I). However, these beneficial effects were absent with KLB overexpression, due to FGFR1 deficiency, consistent with observations of cardiac function (Figure 3H and 3I).

**Figure 3.**
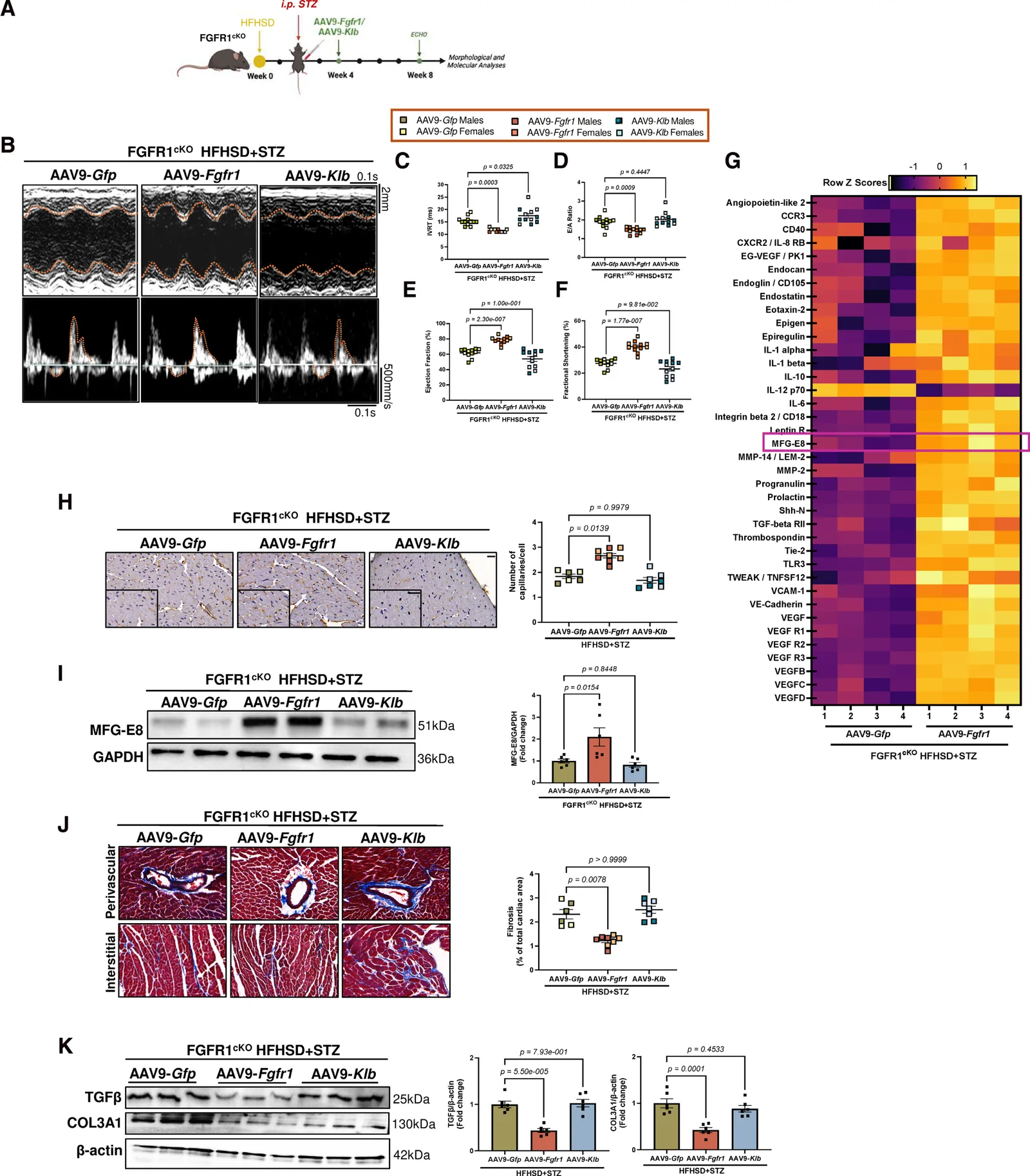
**Cardiac FGFR1 (fibroblast growth factor receptor 1) overexpression, but not KLB (beta-klotho), rescues FGFR1 deficiency-triggered cardiac dysfunction and microvascular impairment under diabetic stress. A**, Schematic illustration of experimental design. **B**, Representative echocardiographic M-mode images (**top**, short-axis view) and pulsed-wave Doppler tracings (**bottom**) from FGFR1^cKO^ mice following the indicated treatments. **C** through **F**, Cardiac functional parameters posttreatment. **C**, Isovolumic relaxation time (IVRT), (**D**) ratio of peak velocity of blood flow from left ventricular relaxation in early diastole to that in late diastole (E/A); (**E**) percentage of left ventricular ejection fraction; (**F**) percentage of left ventricular fractional shortening (N=12 mice of both sexes/group). **G**, Heatmap of differentially expressed cytokines (*q*<0.05) identified by cytokine array analysis in FGFR1^cKO^ mice treated with adeno-associated virus (AAV9)-*Fgfr1* vs AAV9-*Gfp* high-fat, high-sucrose diet combined with streptozotocin (HFHSD+STZ) stress (N=4 hearts from both sexes/group). **H**, Immunohistochemical staining of CD31 and quantification of capillary density (capillaries/cell; N=6 hearts from both sexes/AAV9-*Gfp*-treated group and N=7–8 hearts from both sexes/AAV9-*Fgfr1* or AAV9-*Klb*-treated group; scale=20 µm). **I**, Representative immunoblots and quantification of MFG-E8 protein expression in the whole heart lysates (N=6 hearts from both sexes/group). GAPDH was used as the loading control. **J**, Representative Masson trichrome-stained heart sections (N=6 hearts from both sexes/AAV9-*Gfp*-treated group and N=7–8 hearts from both sexes/AAV9-*Fgfr1* or AAV9-*Klb*-treated group, scale=50 µm). **K**, Representative immunoblots and quantification of TGFβ and COL3A1 protein in the whole heart lysates (N=6 hearts from both sexes/group). β-actin was used as the loading control. Data are presented as mean±SEM. Statistical significance (*P* values shown in the panel) was assessed using ordinary 1-way ANOVA with Šidák multiple comparisons (**C–F**, **H**, **I**, **J**, **K**), and multiple unpaired Student *t* tests with FDR correction set at 1% (**G**).

Furthermore, FGFR1 reinforcement attenuated FGFR1 loss-provoked pathological remodeling under diabetic stress, including myocardial fibrosis (Figure 3J and 3K) and apoptosis (Figure S9A), while KLB restoration alone did not deliver such improvement. Although cardiac hypertrophy was similar in both sexes with treatment of AAV9-*Fgfr1* and AAV9-*Klb* (Figure S9B), only FGFR1 restoration significantly suppressed FGFR1 deficiency-triggered genes indicative of pathological remodeling (*Acta1*, *Nppb*, *Col1a2*, *Col3a1*, *Tgfb*), while enhancing angiogenic genes (*Cdh5*, *Vegfa*); however, these outcomes were not achieved by KLB sole overexpression (Figure S9C).

Furthermore, we evaluated the treatment potential of individual FGFR1 and KLB overexpression or combined overexpression during prolonged diabetic stress. As noted above, 12 weeks of HFHSD+ streptozotocin significantly reduced endogenous FGFR1 and KLB (Figure S1A). To rebuild them in cardiomyocytes, wild-type mice under diabetic stress for 12 weeks were administered AAV9-mediated gene delivery (Figure S10A). Overexpression of FGFR1 or KLB alone was insufficient to rescue diabetes-induced cardiac dysfunction (Figure S10B through S10E; Table S7). Similarly, single-gene overexpression failed to improve capillary density or attenuate pathological fibrosis and cardiac MFG-E8 levels (Figure S10F through S10H). However, dual overexpression of FGFR1 and KLB restored cardiac function in both sexes, with restored microvascular density, cardiac morphology, and MFG-E8 expression (Figure S10B through S10H).

The above data demonstrate that cardiac FGFR1 restoration, in the presence of endogenous KLB, rescues diabetes-induced abnormalities. However, FGFR1 or KLB individual overexpression is insufficient to confer protection under prolonged stress. These findings reinforce the concept that FGFR1 and KLB function cooperatively to preserve cardiac and vascular integrity in both sexes.

### Cardiomyocyte FGFR1/KLB Signaling Preserves Endothelial Survival Via MFG-E8-Mediated Paracrine Effects

We next investigated the mechanistic basis by which FGFR1/KLB signaling in cardiomyocytes exerts proangiogenic effects on ECs. First, we determined that endogenous FGFR1 and KLB levels were unaltered after 8 hours of palmitic acid plus HG stress and declined after 16 hours (Figure S11A). To determine the effects of reduced FGFR1/KLB in cardiomyocytes on microvascular function, NRCMs were transfected with si*RNA* targeting FGFR1 (si*Fgfr1*; Figure S11B) or KLB (si*Klb*; Figure S11C), followed by 8 hours of stress, allowing for assessment of gene-specific effects. The conditioned media from NRCMs were then applied to HUVECs to assess angiogenic outcomes (Figure 4A). First, akin to the in vivo observations, media obtained from cardiomyocytes with loss of either FGFR1 or KLB impaired ECs and their activity, evidenced by decreased CD31^+^ intensity (Figure 4B) and reduced vascular branching (Figure 4C; Table S8) of HUVECs. Additionally, HUVECs cultured in FGFR1- or KLB-loss NRCMs following stress exhibited diminished viability (Figure 4D), compared with HUVECs cultured in the conditioned media from control (si*Negative*) cardiomyocytes. Of note, media from stressed control NRCMs showed no adverse effects on ECs relative to BSA control media (Figure 4B through 4D), indicating that the detrimental effects observed were specific to FGFR1 or KLB knockdown rather than diabetes-like stimulation alone. These effects corresponded with MFG-E8 levels in the media obtained from NRCMs (Figure 4E).

**Figure 4.**
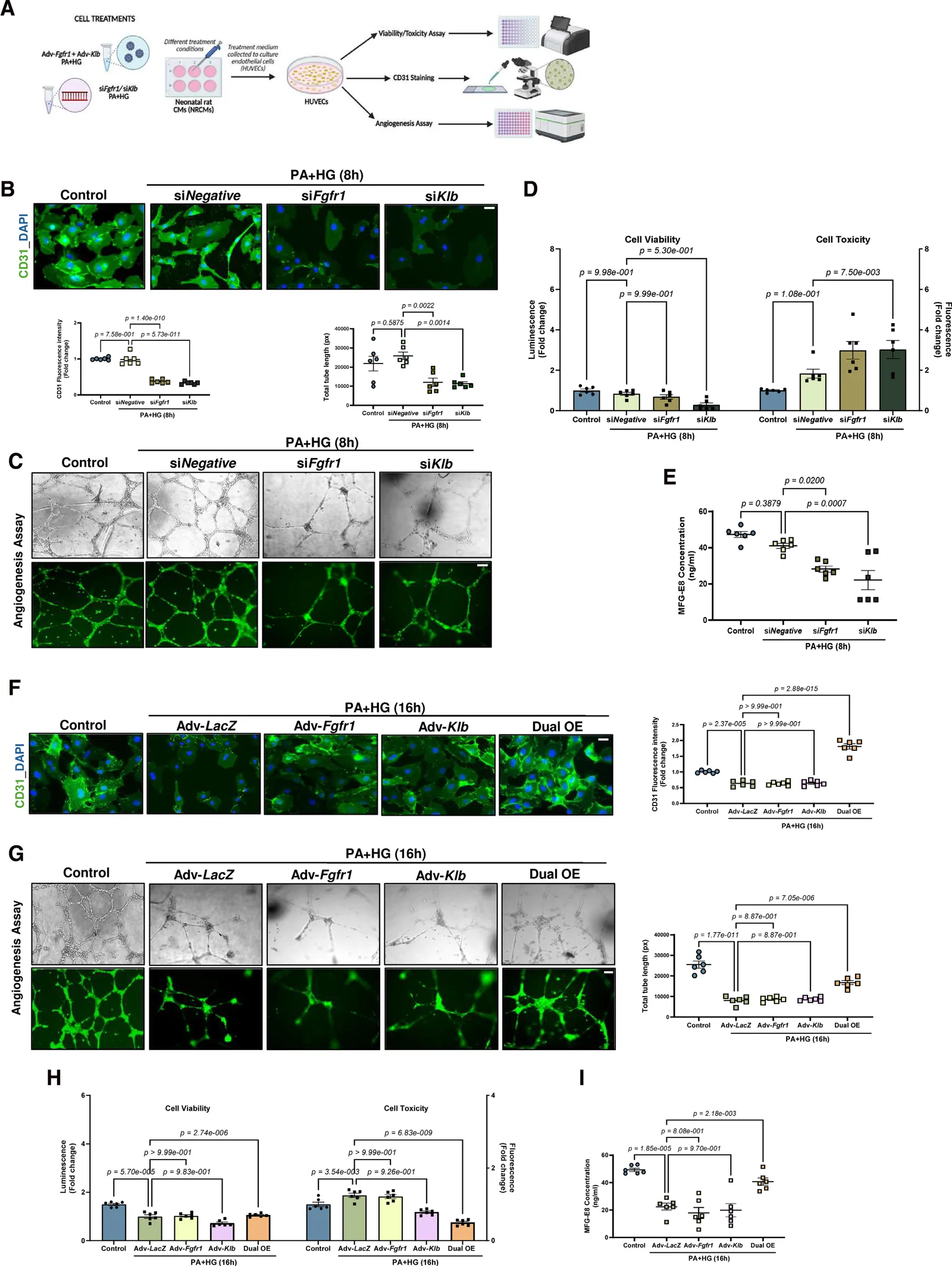
**Cardiomyocyte FGFR1 (fibroblast growth factor receptor 1)/KLB (beta-klotho) signaling supports endothelial maintenance. A**, Schematic of experimental design. Neonatal rat cardiomyocytes (NRCMs) were transfected with si*RNA* targeting FGFR1 (si*Fgfr1*) or KLB (si*Klb*) or infected with adenoviruses expressing FGFR1 (Adv-*Fgfr1*) or KLB (Adv-*Klb*) in the presence of palmitic acid (PA; 500 μmol/L) and high glucose (HG; 25 mmol/L) for varying durations. The conditioned media from NRCMs were subsequently applied to human umbilical vein endothelial cells (HUVECs) to evaluate endothelial cell (EC) responses. **B** through **D**, Effects of FGFR1 or KLB knockdown in NRCMs on ECs. **B**, Immunofluorescence costaining of CD31 (green) and DAPI (blue) in HUVECs cultured in the conditioned media, with quantification of CD31 fluorescence intensity (N=6 experiments; scale=20 µm). **C**, Representative images (fluorescent, **top**; brightfield, **bottom**) and quantification of the angiogenesis assay performed in HUVECs cultured in the conditioned media as in (**B**; N=6 independent biological replicates/group; scale=20 µm). **D**, Quantification of HUVEC viability and cytotoxicity under similar conditions (N=6 experiments). **E**, ELISA of secreted MFG-E8 (milk fat globule-epidermal growth factor 8) in the conditioned media from FGFR1- or KLB-knockdown NRCMs under short-term PA+HG stimulation (N=6 independent biological replicates/group). **F** through **H**, Effects of single or combined FGFR1 and KLB overexpression in NRCMs on ECs. **F**, CD31 and DAPI costaining and fluorescence intensity quantification in HUVECs (N=6 experiments; scale=20 µm). **G**, Representative images (fluorescent, **top**; brightfield, **bottom**) and quantification of the angiogenesis assay performed in HUVECs cultured in the conditioned media as in (**F**; N=6 experiments; scale=20 µm). **H**, HUVEC viability and cytotoxicity measurements under similar conditions (N=6 independent biological replicates/group). **I**, ELISA of secreted MFG-E8 in the conditioned media from NRCMs with FGFR1/KLB single or dual overexpression under long-term stress (N=6 independent biological replicates/group). Data are presented as mean±SEM. Statistical significance (*P* values shown in the panel) was assessed using ordinary 1-way ANOVA with Holm-Šidák multiple comparisons (**B–I**).

Prompted by the synergistic action of FGFR1 and KLB on cardiac function in vivo, we also evaluated whether enhancing either FGFR1 or KLB individual expression or both in cardiomyocytes could rescue endothelial impairment. NRCMs were transduced with adenoviral vectors expressing FGFR1 (Adv-*Fgfr1*; Figure S11D through S11E) and KLB (Adv-*Klb*; Figure S11D and S11F), followed by prolonged palmitic acid +HG exposure for 16 hours. Media from dual FGFR1/KLB-overexpressing NRCMs restored long-term stress-induced endothelial defects, as shown by improved CD31^+^ HUVECs (Figure 4F), enhanced vascular branching (Figure 4G; Table S8), and preserved HUVEC survival (Figure 4H) compared with HUVECs cultured in the conditional media from control (Adv-*LacZ*) cells. Accordingly, MFG-E8 secretion was also enhanced from dual FGFR1/KLB-overexpressing NRCMs (Figure 4I). However, single-gene overexpression failed to achieve the effects (Figure 4F through 4I). Overall, these findings suggest the crucial role of FGFR1/KLB signaling in mediating cardiomyocyte EC crosstalk and angiogenesis, likely with cardiomyocyte-sourced MFG-E8 involved.

### MFG-E8 Recombinant Protein Restores FGFR1/KLB Loss-induced Capillary Rarefaction and Cardiac Dysfunction

To functionally validate MFG-E8 as a downstream effector of cardiac FGFR1/KLB signaling, recombinant MFG-E8 (rMFG-E8; 40 μg/kg body weight/wk)^27–29^ was administered intraperitoneally to FGFR1^cKO^ and KLB^cKO^ mice to determine whether it could rescue the cardiovascular defects caused by FGFR1 or KLB deficiency. The treatment commenced after 4 weeks of diabetic stress and continued weekly for an additional 4 weeks (Figure 5A). Elevated MFG-E8 was detected in cardiac tissue and serum in both knockout mice following rMFG-E8 treatment (Figure S12A through S12D). Although systemic metabolic parameters remained unaffected by rMFG-E8 treatment (Figure S12E and S12F), it improved overall cardiac function in both diabetic FGFR1^cKO^ (Figure 5B through 5E; Table S9) and KLB^cKO^ mice (Figure 5F through 5I; Table S10). Importantly, proteomic profiling revealed that rMFG-E8 treatment upregulated pathways of EC proliferation and angiogenesis-associated pathways in KLB-deficient hearts (Figure 5J). This was further corroborated by improved capillary density in both FGFR1^cKO^ (Figure 5K) and KLB^cKO^ (Figure 5L) diabetic hearts, irrespective of sex. Moreover, rMFG-E8 treatment improved myocardial pericyte density as well (Figure S13A and S13B).

**Figure 5.**
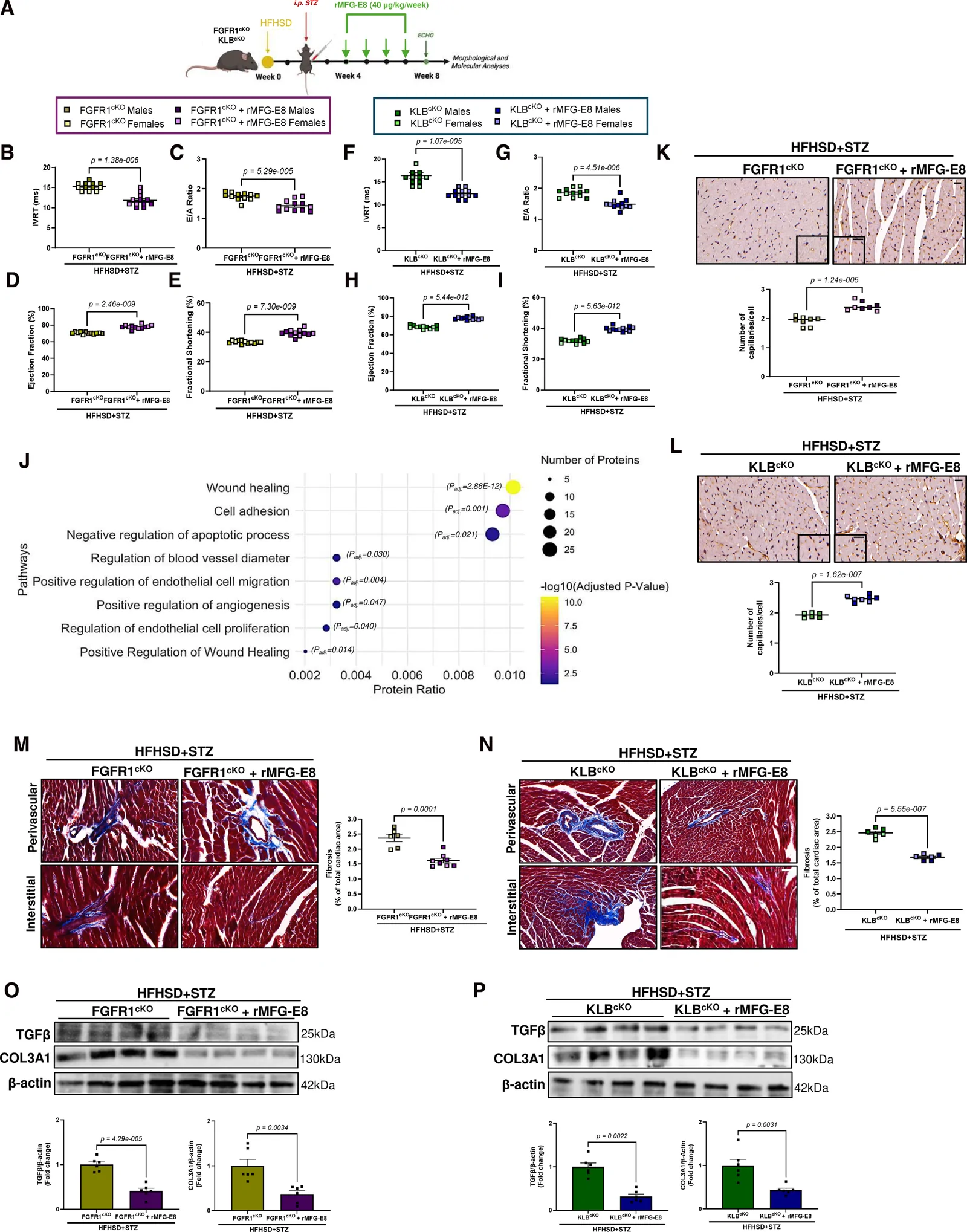
**Recombinant MFG-E8 (milk fat globule-epidermal growth factor 8) treatment attenuates FGFR1 (fibroblast growth factor receptor 1) or KLB (beta-klotho) loss-induced cardiac dysfunction and restores vascular integrity. A**, Schematic of experimental design. **B** through **E**, Cardiac functional parameters in FGFR1^cKO^ with or without rMFG-E8 treatment under high-fat, high-sucrose diet plus streptozotocin (HFHSD+STZ) stress. **B**, Isovolumic relaxation time (IVRT); (**C**) ratio of peak velocity of blood flow from left ventricular relaxation in early diastole to that in late diastole (E/A); (**D**) percentage of left ventricular ejection fraction; (**E**) percentage of left ventricular fractional shortening (N=12 mice of both sexes/group). **F** through **I**, Cardiac functional parameters in KLB^cKO^ with or without rMFG-E8 treatment under HFHSD+STZ stress. **F**, Isovolumic relaxation time (IVRT); (**G**) ratio of peak velocity of blood flow from left ventricular relaxation in early diastole to that in late diastole (E/A ratio), (**H**) percentage of left ventricular ejection fraction, (**I**) percentage of left ventricular fractional shortening (N=12 mice of both sexes/group). **J**, Pathway enrichment analysis of differentially expressed proteins (*P*_*adj.*_<0.05) identified by liquid chromatography-mass spectrometry (LC-MS/MS) in KLB^cKO^ hearts treated with rMFG-E8 compared with the untreated group (N=4 hearts from both sexes/group). Enriched angiogenic pathways (*P*_*adj*_.<0.05) are presented. **K** through **L**, Immunohistochemical staining of CD31 and capillary density quantification (capillaries/cell) in (**K**) FGFR1^cKO^ and (**L**) KLB^cKO^ hearts in the absence and presence of rMFG-E8 treatment under diabetic stress (N=6–8 hearts from both sexes/control group and N=8 hearts from both sexes/rMFG-E8-treated group; scale=20 µm). **M** and **N**, Representative Masson trichrome-stained heart sections of (**M**) FGFR1^cKO^ and (**N**) KLB^cKO^ mice with or without rMFG-E8 treatment (N=6 hearts from both sexes/control group and N=6–8 hearts from both sexes/rMFG-E8-treated group, scale=50 µm). **O** and **P**, Representative immunoblots and quantification of TGFβ and COL3A1 protein expression in the whole heart lysates of (**O**) FGFR1^cKO^ and (**P**) KLB^cKO^ mice (N=6 hearts from both sexes/group). β-actin was used as the loading control. Data are presented as mean±SEM. Statistical significance (*P* values shown in the panel) was assessed using 2-tailed unpaired Student *t* test (**B–E**, **G–I**, **K–P**) and the Mann-Whitney *U* test (**F**).

At the transcriptional level, rMFG-E8 treatment resulted in profound upregulation of angiogenic markers (*Cdh5*, *Vegfa*) in both FGFR1- and KLB-deficient hearts (Figure S13C and S13D). Concurrently, cardiac pathological remodeling was alleviated by rMFG-E8 treatment, as indicated by a reduction in pathological hypertrophic and fibrotic markers (*Acta1*, *Nppb*, *Col1a2*, *Col3a1*, *Tgfb*; Figure S13C and S13D). Histological analyses further confirmed that rMFG-E8 treatment reduced myocardial fibrosis (Figure 5M through 5P) and apoptosis (Figure S13E and S13F) in the hearts. Cardiomyocyte size was unaffected (Figure S13G and S13H), in line with observations in myoblasts (Figure S14A and S14B), indicating that the beneficial effects of rMFG-E8 are predominantly through vascular mechanisms rather than direct modulation of cardiomyocyte growth. Collectively, these findings provide compelling functional evidence that MFG-E8 operates as a key effector of FGFR1/KLB signaling in cardiomyocytes, and its restoration via recombinant protein therapy improves microvascular integrity and preserves cardiac function under diabetic stress.

### CEBPβ as a Downstream Mediator of FGFR1/KLB Signaling Transcriptionally Regulates MFG-E8 in Cardiomyocytes

Given the observed reduction in *Mfge8* level due to FGFR1 or KLB deletion, we hypothesized that the FGFR1/KLB pathway governs transcriptional regulation of MFG-E8. *In silico* promoter analyses using Ensembl and the UCSC Genome Browser identified conserved CEBP (CCAAT/enhancer-binding protein) binding motifs within the MFG-E8 promoter across mammalian species (Figure 6A and 6B). Among the CEBP family, CEBPβ was reduced significantly on prolonged diabetic stress (Figure S15). A luciferase reporter assay first indicated CEBPβ direct binding to *Mfge8* putative promoter regions (Figure 6C). Supportively, 2 mutations in the predicted binding regions abolished transcriptional activity (Figure 6C and 6D), supporting CEBPβ as a direct transcriptional regulator of *Mfge8*.

**Figure 6.**
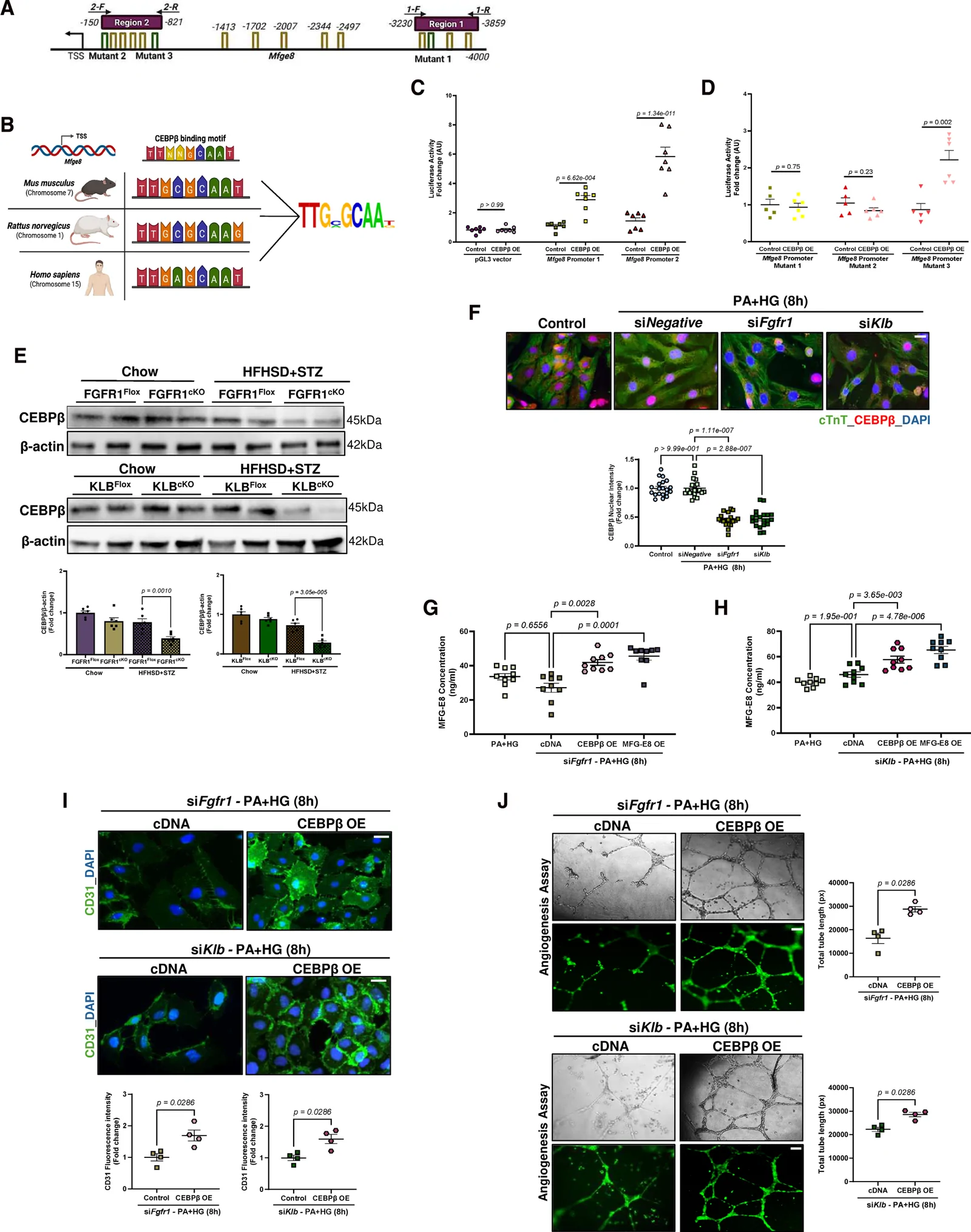
**FGFR1 (fibroblast growth factor receptor 1)/KLB (beta-klotho)-activated CEBPβ (CCAAT/enhancer-binding protein beta) transcriptionally upregulates MFG-E8 (milk fat globule-epidermal growth factor 8) in cardiomyocytes to promote angiogenesis. A**, Schematic illustrating conserved CEBPβ binding regions within the *Mfge8* promoter. Two targeted regions (region 1 and region 2) are shown. Yellow bars denote putative binding sites; green bars indicate confirmed binding regions. The 3 confirmed binding regions were mutated as illustrated. **B**, Schematic demonstrating the conserved CEBPβ binding site within the *Mfge8* promoter across mammalian species. **C**, Luciferase reporter assay in neonatal rat cardiomyocytes (NRCMs) showing increased *Mfge8* promoter activity following CEBPβ overexpression (N=7 independent biological replicates/group). **D**, Luciferase reporter assay in NRCMs showing that mutation of CEBPβ binding sites abolishes CEBPβ-induced *Mfge8* promoter activity (N=5–6 independent biological replicates/group). **E**, Representative immunoblots and quantification of CEBPβ expression in the hearts of FGFR1^cKO^ (**top**) and KLB^cKO^ (**bottom**) mice (N=6 hearts from both sexes/group) under chow or a high-fat, high-sucrose diet combined with streptozotocin (HFHSD+STZ) stress. β-actin was used as the loading control. **F**, Immunofluorescent images showing nuclear CEBPβ (red), cTnT (cardiac troponin T; green), and DAPI (blue) in FGFR1- or KLB- knockdown NRCMs using their si*RNA* under control or short-term palmitic acid (PA; 500 μmol/L) and high glucose (HG; 25 mmol/L) stress (N=20 cells from 4 independent biological replicates/group, scale=20 µm). **G** and **H**, ELISA-based quantification of secreted MFG-E8 in the conditioned media from (**G**) FGFR1-knockdown NRCMs and (**H**) KLB-knockdown NRCMs with CEBPβ or MFG-E8 overexpression under short-term stress (N=9 independent biological replicates/group). **I**, CD31 (green) and DAPI (blue) immunofluorescence of human umbilical vein endothelial cells (HUVECs) cultured in the conditioned media obtained from FGFR1- (**top**) or KLB- (**bottom**) knockdown NRCMs following CEBPβ overexpression (N=4 independent biological replicates/group; scale=20 µm). **J**, Representative images (fluorescent, **top**; brightfield, **bottom**) and quantification of the angiogenesis assay performed in HUVECs cultured in the conditioned media as in (**H**), with CEBPβ overexpression (N=4 independent biological replicates/group; scale=20 µm). Data are presented as mean±SEM. Statistical significance (*P* values shown in the panel) was assessed using ordinary 2-way ANOVA (**C**, **E**, and **F**) with Šidák multiple comparisons, permutation-based 2-way ANOVA (**D**), 1-way ANOVA with Šidák multiple comparisons (**H**), the Kruskal-Wallis test with Dunn multiple comparisons (**G**), and the Mann-Whitney *U* test (**I** and **J**).

Moreover, both FGFR1^cKO^ and KLB^cKO^ mice exhibited significantly reduced cardiac CEBPβ under diabetes (Figure 6E), supporting their potential influence on CEBPβ. Consistently, knockdown of FGFR1 or KLB in NRCMs blocked nuclear localization and expression of CEBPβ (Figure 6F; Figure S16A) under short-term diabetes-like stress. Conversely, simultaneous overexpression of FGFR1 and KLB in NRCMs maintained nuclear localization of CEBPβ even under prolonged stress, while single-gene overexpression was insufficient (Figure S16B). Notably, overexpression of KLB alone failed to enhance CEBPβ in FGFR1-knockout hearts (Figure S16C), endorsing the synergistic function of FGFR1 and KLB in preserving CEBPβ expression.

CEBPβ requires phosphorylation for its activation and nuclear translocation, with ERK1/2 (extracellular signal-regulated kinases 1 and 2) serving as a kinase that phosphorylates and enhances CEBPβ transcriptional activity.^32–35^ Since ERK1/2 can be regulated by FGFR1 signaling,^36,37^ a reduction in phosphorylated ERK1/2 was observed in FGFR1- and KLB-deficient hearts under diabetic stress, accompanied by decreased phosphorylated CEBPβ (Figure S16D and S16E), indicating impaired CEBPβ activation. These data indicate that FGFR1/KLB signaling governs CEBPβ transcription factor action through a kinase activation pathway.

We, therefore, examined the consequences of the FGFR1/KLB-CEBPβ axis on MFG-E8 expression and secretion from cardiomyocytes. First, overexpression of the transcriptionally active isoform of CEBPβ (Figure S16F) restored MFG-E8 in FGFR1- or KLB-deficient NRCMs (Figure S16G and S16H). Of note, secreted MFG-E8 levels in the conditioned media from CEBPβ-overexpressing NRCMs were enhanced, despite FGFR1 or KLB deficiency (Figure 6G and 6H). Additionally, these phenomena were reproduced by direct MFG-E8 overexpression (Figure S16F) under identical conditions (Figure S16G and S16H; Figure 6G and 6H). Most importantly, the conditioned media from CEBPβ-overexpressing FGFR1- or KLB-knockdown NRCMs restored angiogenesis in HUVECs, as evidenced by increased CD31^+^ intensity (Figure 6I) and improved vascular branching (Figure 6J; Table S11).

Furthermore, we also assessed the responsiveness of the FGFR1/KLB pathway using FGF21, a canonical ligand of the FGFR1/KLB receptor complex.^9,16,17^ As expected, recombinant FGF21 (rFGF21, 500 ng/mL)^17^ treatment increased MFG-E8 luciferase activity, which was diminished by mutations observed above (Figure S17A and S17B). Furthermore, rFGF21 also enhanced CEBPβ nuclear localization (Figure S17C). However, rFGF21 treatment failed to restore MFG-E8 secretion from FGFR1- or KLB-deficient cardiomyocytes (Figure S17D). These results support that FGFR1/KLB activation by FGF21 promotes *Mfge8* transcription via CEBPβ. Moreover, CEBPβ knockdown (Figure S17E) abolished MFG-E8 expression and secretion despite rFGF21 treatment (Figure S17F through S17G). Overall, these findings reinforce that FGFR1/KLB signaling activation in cardiomyocytes facilitates CEBPβ activity, which transcriptionally regulates MFG-E8 expression and its secretion to promote angiogenesis in a paracrine manner.

### Cardiac CEBPβ Overexpression Rescues Cardiovascular Impairment

To further gain functional evidence of CEBPβ in mediating FGFR1/KLB pathway-dependent cardiovascular protection, we evaluated whether cardiac-specific overexpression of CEBPβ (AAV9-*CEBPB*) reverses detrimental effects in FGFR1^cKO^ and KLB^cKO^ mice (Figure S18A, Figure 7A). Despite comparable fasting blood glucose levels across groups (Figure S18B and S18C), overall cardiac detrimental effects in FGFR1^cKO^ (Figure 7B through 7E) and KLB^cKO^ mice (Figure 7F through 7I) were mitigated by cardiac CEBPβ overexpression when analyses were performed without sex stratification. However, a comparatively reduced protective effect on diastolic function was observed in female diabetic mice (Tables S12 and S13).

**Figure 7.**
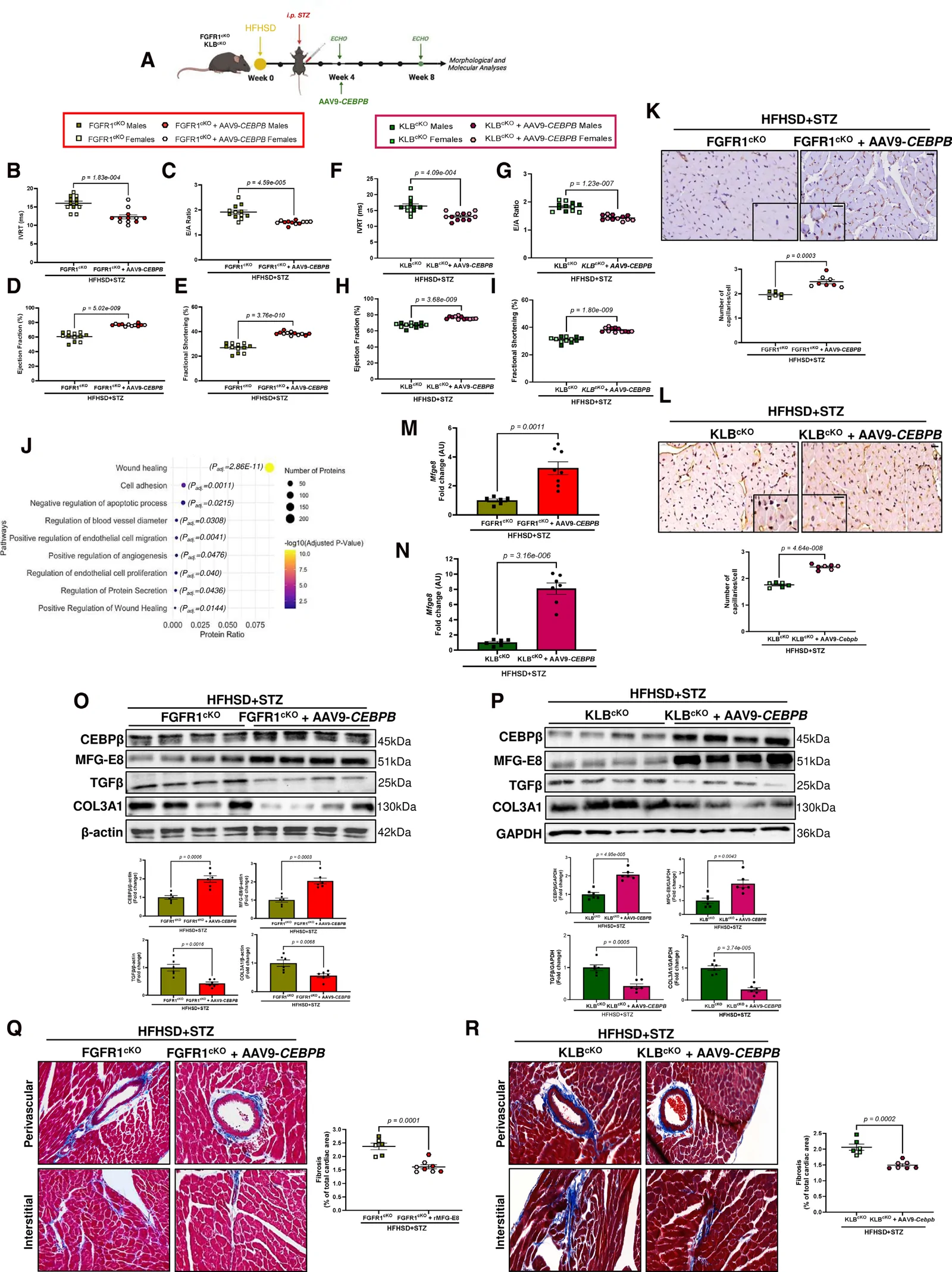
**Cardiac CEBPβ (CCAAT/enhancer-binding protein beta) overexpression mitigates cardiac dysfunction and restores microvascular integrity in FGFR1^cKO^ and KLB^cKO^ mice. A**, Schematic of experimental design. **B** through **E**, Cardiac functional parameters in FGFR1^cKO^ with or without CEBPβ (AAV9-*CEBPB*) overexpression under high-fat, high-sucrose diet plus streptozotocin (HFHSD+STZ) stress. **B**, Isovolumic relaxation time (IVRT), (**C**) ratio of peak velocity of blood flow from left ventricular relaxation in early diastole to that in late diastole (E/A), (**D**) percentage of left ventricular ejection fraction, (**E**) percentage of left ventricular fractional shortening (N=12 mice of both sexes/group). **F** through **I**, Cardiac functional parameters in KLB^cKO^ with or without CEBPβ overexpression under HFHSD+STZ stress. **F**, Isovolumic relaxation time (IVRT); (**G**) ratio of peak velocity of blood flow from left ventricular relaxation in early diastole to that in late diastole (E/A Ratio); (**H**) percentage of left ventricular ejection fraction; (**I**) percentage of left ventricular fractional shortening (N=12 mice of both sexes/group). **J**, Gene ontology (GO) pathway enrichment analyses of differentially expressed proteins (*P*_*adj.*_<0.05) identified via liquid chromatography-mass spectrometry (LC-MS/MS) in KLB^cKO^ hearts following AAV9-*CEBPB* injection vs AAV9-*Gfp* (N=4 hearts from both sexes/group). Enriched angiogenic pathways (*P*_*adj*_.<0.05) are presented. **K** and **L**, Immunohistochemical staining of CD31 and quantification of capillary density (capillaries/cell) in (**K**) FGFR1^cKO^ and (**L**) KLB^cKO^ hearts in the absence and presence of AAV9-*CEBPB* treatment under diabetic stress (N=6 hearts from both sexes/control group and N=7–8 hearts from both sexes/AAV9-*CEBPB*-treated group; scale=20 µm). **M** and **N**, Quantitative PCR analysis of *Mfge8* in (**M**) FGFR1^cKO^ and (**N**) KLB^cKO^ hearts (N=6 hearts from both sexes/control group and N=7–8 hearts from both sexes/AAV9-*CEBPB*-treated group). **O** and **P**, Representative immunoblots and quantification of cardiac CEBPβ, MFG-E8, TGFβ, and COL3A1 expression in (**O**) FGFR1^cKO^ and (**P**) KLB^cKO^ hearts (N=6 hearts from both sexes/group). β-actin or GAPDH was used as the loading control. **Q** and **R**, Representative images and quantification of Masson trichrome-stained heart sections of (**Q**) FGFR1^cKO^ and (**R**) KLB^cKO^ hearts with or without treatment (N=6 hearts from both sexes/control group and N=7–8 hearts from both sexes/AAV9-*CEBPB*-treated group). Data are presented as mean±SEM. Statistical significance (*P* values shown in the panel) was assessed using 2-tailed unpaired Student *t* test (**C–I**, **K–R**) and the Mann-Whitney *U* test (**B**).

Proteomics and pathway enrichment analyses demonstrated that angiogenesis-related and EC activation pathways were altered by CEBPβ overexpression in KLB^cKO^ mice (Figure 7J). Furthermore, capillary density was recovered in knockout hearts (Figure 7K and 7L), irrespective of sex, which was paralleled by the transcript and protein levels of MFG-E8 in the myocardium (Figure 7M through 7P), affirming that CEBPβ positively regulates MFG-E8 expression in vivo.

Moreover, the increase in other angiogenic markers (*Vegfa*, *Cdh5*) also supported preservation of microvasculature by CEBPβ restoration in both FGFR1- and KLB-deficient models (Figure S19A and S19B). Consistent with the observations from rMFG-E8 treatment, cardiac-specific CEBPβ overexpression ameliorated cardiac pathological remodeling, as determined by repression of key genes (*Acta1*, *Nppb*, *Col1a2*, *Col3a1*, *Tgfb*; Figure S19A and S19B) and decreased myocardial fibrosis (Figure 7O through 7R) and apoptosis (Figure S19C and S19D), despite no significant change in cardiomyocyte size (Figure S19E and S19F). Together, these results provide functional evidence that CEBPβ, acting downstream of the FGFR1/KLB signaling pathway, can facilitate MFG-E8 expression and sustain angiogenic response and cardiac function in T2DM.

### FGFR1/KLB-CEBPβ Axis in Human Stem Cell-Derived Cardiomyocyte Mediates Endothelial Protection via MFG-E8

Finally, we evaluated the translational relevance of the FGFR1/KLB-CEBPβ-MFG-E8 axis in hiPSC cardiomyocytes Figure S20) and human embryonic stem cell-derived cardiomyocytes under diabetic-like stress. Recapitulating the phenotypes observed above, prolonged palmitic acid +HG exposure downregulated FGFR1 and KLB expression, accompanied by decreased total and nuclear CEBPβ levels in hiPSC cardiomyocytes (Figure 8A and 8B). This was paralleled by reduced MFG-E8 expression and secretion (Figure 8A and 8C). Additionally, human embryonic stem cell-derived cardiomyocytes also displayed the same phenomenon (Figure S21A and S21B).

**Figure 8.**
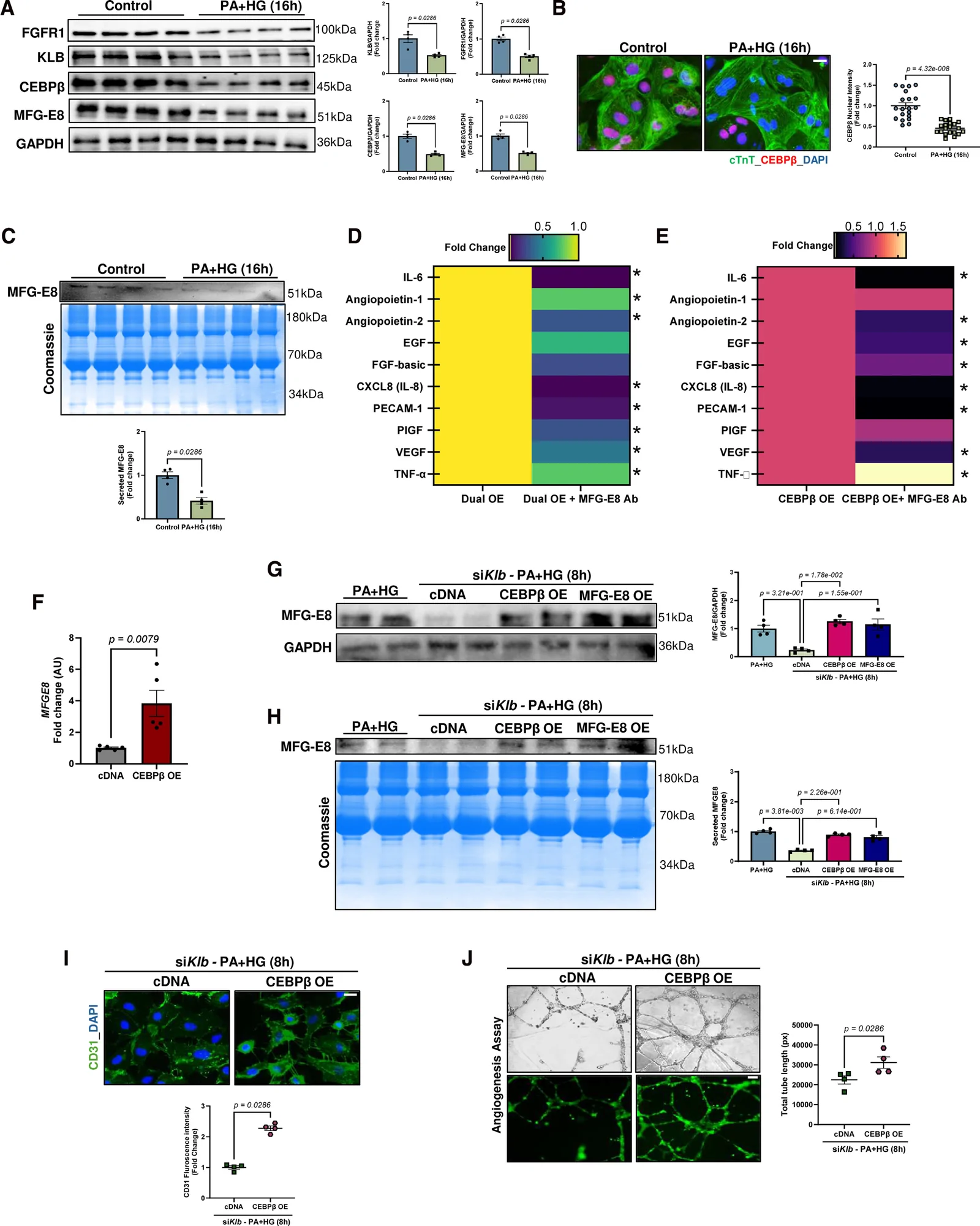
**FGFR1 (fibroblast growth factor receptor 1)/KLB (beta-klotho)-CEBPβ (CCAAT/enhancer-binding protein beta) signaling preserves angiogenesis via an MFG-E8 (milk fat globule-epidermal growth factor 8)-dependent manner in human cardiomyocytes. A**, Representative immunoblots and quantification in human induced pluripotent stem cell-derived cardiomyocytes (hiPSC-CMs) following prolonged exposure to palmitic acid (PA; 500 μmol/L) and high glucose (HG; 25 mmol/L; N=4 independent biological replicates/group). GAPDH was used as the loading control. **B**, Immunofluorescent images and quantification showing nuclear localization of CEBPβ (red), cTnT (cardiac troponin T; green), and DAPI (blue) in response to longer-term stress (N=20 cells from 4 independent biological replicates/group; scale=20 µm). **C**, Representative immunoblots and quantification of secreted MFG-E8 in the media culturing hiPSC-CMs under PA+HG stress (N=4 independent biological replicates/group). Coomassie blue staining assessed total protein as a loading control. **D** and **E**, Heatmaps showing angiogenic cytokine expression in human umbilical vein endothelial cells (HUVECs) cultured in the conditioned media obtained from (**D**) dual FGFR1- and KLB-overexpressing hiPSC-CMs, in the absence and presence of anti-MFG-E8 antibody (MFG-E8 Ab) in the media (1 µg/mL; N=6 independent biological replicates/group) and (**E**) CEBPβ-overexpressing hiPSC-CMs, in addition to anti-MFG-E8 antibody in the media (1 µg/mL; N=6 experiments; cytokines with *q*<0.05 are marked with*). **F**, Quantitative PCR analysis of *MFGE8* in hiPSC-CMs with CEBPβ overexpression (N=5 independent biological replicates/group). **G** and **H**, Representative immunoblots and quantification of (**G**) intracellular and (**H**) secreted MFG-E8 protein levels (N=4 independent biological replicates/group). GAPDH and Coomassie blue staining served as the loading control for cell lysates and media, respectively. **I**, Immunofluorescent staining for CD31 (green) and DAPI (blue) of HUVECs cultured in the conditioned media obtained from CEBPβ-overexpressing KLB-deficient hiPSC-CMs (N=4 independent biological replicates/group; scale=20 µm). **J,** Representative images (fluorescent, top; brightfield, bottom) and quantification of the angiogenesis assay performed on HUVECs cultured in the conditioned media as in (**I**; N=4 independent biological replicates/group; scale=20 µm). Data are presented as mean±SEM. Statistical significance (*P* values shown in the panel) was assessed using the Mann-Whitney *U* test (**A**, **C**, **F**, **I**, **J**), 2-tailed unpaired Student *t* test (**B**), multiple unpaired Mann-Whitney U tests with FDR correction set at 1% (**D** and **E**), and the Kruskal-Wallis test with Dunn multiple comparisons (**G** and **H**).

Functionally, the conditioned media from hiPSC cardiomyocytes overexpressing FGFR1 and KLB significantly increased the secretion of angiogenic cytokines in HUVECs, as determined by LEGENDplex multiplex angiogenetic analyses (Figure 8D; Figure S22A through S22C; Table S14). This paracrine angiogenic response was attenuated on neutralization of MFG-E8 with a blocking antibody (Figure 8D; Table S14). Similarly, the conditioned media from CEBPβ-overexpressing hiPSC cardiomyocytes also augmented angiogenic cytokine secretion from HUVECs, an effect that was abolished by MFG-E8 blockade in the media (Figure 8E; Table S14). Collectively, these results substantiate the fact that FGFR1/KLB and CEBPβ in cardiomyocytes govern MFG-E8-dependent effects on ECs.

More convincingly, overexpression of CEBPβ in hiPSC cardiomyocytes (Figure S23A) increased MFG-E8 transcripts (Figure 8F). Notably, in KLB-knockdown hiPSC cardiomyocytes (Figure S23B), CEBPβ overexpression rescued MFG-E8 expression and secretion under palmitic acid +HG stimulation (Figure 8G and 8H), which was comparable to the outcomes obtained by direct MFG-E8 overexpression (Figure S23C; Figure 8G and 8H). Importantly, the conditioned media from CEBPβ-overexpressing KLB-deficient hiPSC cardiomyocytes maintained angiogenic responses in HUVECs (Figure 8I) and improved vascular branching complexity (Figure 8J; Table S15). Together, these data confirm that the FGFR1/KLB-CEBPβ signaling axis in cardiomyocytes regulates MFG-E8-mediated proangiogenic paracrine effects on ECs, thereby highlighting a conserved and targetable mechanism of cardiomyocyte EC crosstalk under diabetic stress.

## Discussion

In this study, prompted by observations of impaired angiogenic pathways in diabetic hearts, we identified FGFR1/KLB signaling in cardiomyocytes as a critical moderator of cardiomyocyte EC crosstalk and microvasculature, mediated by MFG-E8. Various loss-of-function and gain-of-function studies have demonstrated that cardiac FGFR1/KLB is required to maintain microvascular integrity and support adaptive cardiac remodeling to tackle stress.

### Cardiac Capillary Defects Trigger Cardiac Dysfunction

Microvascular injury is recognized as an important contributor to the development and progression of HF in response to ischemia, hypertension, metabolic disorders, and aging.^38–41^ High-energy demands of the myocardium necessitate a well-structured and responsive microvascular network to ensure adequate delivery of oxygen and nutrients.^3,42^ Under stimulation, vascular expansion determines whether cardiac growth is developed physiologically (adaptive) or pathologically (decompensated).^43^ During acute stages, the heart undergoes remodeling to maintain cardiac output; however, prolonged stress disrupts microvascular angiogenesis, which cannot meet the oxygen and nutrient demands of the heart, leading to decompensated hypertrophy and ultimately cardiac dysfunction.^44^ Microvascular dysfunction also triggers activation of cardiac fibroblasts, promoting interstitial fibrosis and reduced ventricular compliance, which is especially a feature of HF with preserved ejection fraction.^45,46^ Since clinical studies demonstrate that patients with HF with preserved ejection fraction exhibit capillary rarefaction and fibrosis,^1^ our findings underscore the critical need to preserve microvascular integrity and enhance angiogenic capacity as a strategy for cardioprotection.

### FGFR1 and KLB in Cardiomyocytes Synergistically Mediate Cardioprotection

In the heart, FGFR1 is required for cardiomyocyte proliferation at embryonic stages,^10^ while repression of FGFR1 is associated with cardiomyocyte cycle arrest and inhibition of dedifferentiation in adults, highlighting its importance beyond developmental stages.^11^ Similar to the previous findings showing that endothelial FGFR1 deficiency exacerbates myocardial fibrosis in diabetes, we demonstrate that cardiomyocyte-specific FGFR1 deletion rendered the hearts more vulnerable to decompensated remodeling under diabetic stress, evidenced by elevated pathological hypertrophic markers, such as *Nppb*. Interestingly, our findings contradict the observations that the TLR4 (toll-like receptor 4) pathway enhances myocardial inflammation and fibrosis via FGFR1 under hyperglycemic stress.^47^ This discrepancy may be attributed to differences in experimental models, including their use of an inducible Cre-LoxP system and a more aggressive type 1 diabetes model.

In parallel, KLB, a mandatory coreceptor for FGFR1 function, assists ligand binding and FGFR1 activation. For instance, KLB is essential for FGF21-FGFR1 binding to confer beneficial metabolic actions in adipose tissue.^48^ Suppression of KLB in ECs promotes endothelial-to-mesenchymal transition and fibrotic response.^49^ Additionally, KLB mediates cardioprotective potential during exercise.^50^ Our study extends the role of KLB, showing that the reduction in cardiac FGFR1/KLB contributed to microvascular dysfunction and aggravation of diabetic HF. Notably, KLB overexpression alone failed to rescue the deleterious cardiac effects observed in the setting of FGFR1 deficiency, indicating that KLB is contingent to protect the heart in an FGFR1-dependent manner.

Of note, we found that FGFR1 and KLB expression on the plasma membrane was reduced in diabetic failing hearts, implicating their loss in the pathogenesis of T2DM-associated HF. Importantly, their transcripts were unaltered, indicating that their reduction is due to post-translational regulation under prolonged diabetes. The membrane receptors can be either internalized and recycled for functioning or degraded by the lysosomal system, but the impaired balance affects the expression and function of the receptors.^30^ Polyubiquitin-mediated degradation of membrane receptors can lower their expression and inhibit their activity, including FGFRs.^31^ This is in line with our observations that FGFR1 and KLB recycling was decreased under diabetic stress, while their ubiquitination was increased, indicating more degradation. However, future studies are needed to determine whether FGFR1/KLB is tagged with lysine (K) 63-linked polyubiquitin chains to be targeted by the lysosome and whether the endosomal machinery delivered to lysosomes is predominant during the process. Although enhanced FGFR1 and KLB degradation was accompanied by reduced receptor recycling, it remains unclear whether impaired reuse leads to more degradation, or vice versa. Additionally, ubiquitination is increased under hyperglycemic conditions, also driving cytosolic proteasome-dependent protein degradation,^51^ which is in line with our observations for FGFR1 and KLB; however, K48-linked polyubiquitin chains for the proteasome also need to be tested in the future. Clarifying the detailed mechanistic interplay between these processes, understanding the molecular basis of dysregulation of FGFR1 and KLB under diabetic stress conditions, and identifying potential therapeutic targets will require further dedicated investigation beyond the scope of the current study. Nevertheless, our findings demonstrate that cardiac-specific dual overexpression of FGFR1 and KLB mitigated adverse remodeling, reinforcing the cardioprotective role of the signaling pathway and supporting the therapeutic potential of targeting this axis.

### CEBPβ as a Downstream Effector of the FGFR1/KLB Pathway

CEBPβ plays roles in cellular proliferation, differentiation, and survival. In the heart, suppression of CEBPβ during exercise attenuates hypertrophy.^52,53^ Additionally, CEBPβ is upregulated in phenylephrine-induced hypertrophic cardiomyocytes in vitro,^54^ which may be associated with compromised cardiac function.^52^ Conversely, restoration of CEBPβ mitigates adverse cardiac phenotypes in ischemic heart disease.^55^ CEBPβ’s function is likely context-dependent; therefore, the longer-term impact of CEBPβ modulation on cardiac remodeling on distinct stresses should be investigated in depth. Our study based on diabetic conditions showed that reconstitution of CEBPβ alleviated pathological remodeling.

Mechanistically, after identifying CEBPβ as a transcription factor of MFG-E8, we determined that it functions as an unrecognized downstream effector of FGFR1/KLB in cardiomyocytes. Prior evidence suggests that FGFR1 activates CREBBP (cAMP response element-binding protein binding protein),^56^ which can transcriptionally upregulate CEBPβ.^57^ In addition, our findings indicated that FGFR1/KLB regulates CEBPβ activation likely via ERK1/2 as an intermediate kinase in cardiomyocytes. We therefore hypothesized that FGFR1/KLB deficiency suppressed ERK1/2 action on CEBPβ phosphorylation and nuclear translocation, thereby promoting its degradation, like other transcription factors.^58,59^ This provides a plausible indirect pathway through which the FGFR1/KLB pathway enhances CEBPβ action, resulting in its protective roles. However, the reduction of MFG-E8 in floxed diabetic mice despite preserved CEBPβ levels suggests that additional regulatory pathways or increased MFG-E8 degradation may contribute to its downregulation. Thus, further investigation into the molecular regulation of MFG-E8 in the heart is warranted.

### Dual Roles of MFG-E8 in the Heart

Cardiac cells communicate through soluble paracrine factors, and we identified MFG-E8 as an essential mediator in this context. A cytokine array of the conditioned medium obtained from myoblasts overexpressing FGFR1/KLB uncovered an increase in secreted levels of MFG-E8.^17^ Although cardiac MFG-E8 was slightly reduced in the diabetic heart in floxed mice, probably due to other upregulating signaling, FGFR1/KLB deficiency was the main cause of downregulating it in cardiomyocytes. MFG-E8 is a secreted integrin-binding protein known to interact with integrins to regulate neovascularization and angiogenesis by influencing VEGF (vascular endothelial growth factor) in ECs or PDGF (platelet-derived growth factor) in pericytes.^19,22^ We did not detect alterations of *ITGAV*, *ITGB3*, and *ITGB5*, indicative of unchanged alpha V beta 3 (α_V_β_3_) and alpha V beta 5 (α_V_β_5_) integrins in human diabetic failing hearts. Thus, we hypothesized that reduced capillaries in the diabetic myocardium are due to reduced ligand/growth factors and therefore explored the paracrine role of cardiomyocyte-derived MFG-E8 in endothelial maintenance. Notably, rMFG-E8 treatment led to upregulation of *Vegfa*, suggesting a stimulatory effect on angiogenic pathways. Although the precise downstream mechanisms of cardiomyocyte-secreted MFG-E8-mediated angiogenesis remain to be elucidated, prior evidence suggests that integrin receptors on ECs are likely critical mediators of this effect.^22^ It is worth noting that we observed more EC death and loss of pericytes in the failing hearts; these phenomena may be due to EC-pericyte or cardiomyocyte-pericyte feedback loops regulated by other paracrine mechanisms, which need to be further investigated to advance the understanding of the regulation of microvascular function in diabetic hearts.

Moreover, although MFG-E8 also exerts systemic effects across multiple organs, primarily due to its roles in clearing apoptotic cells and preventing inflammation,^60^ the current study only demonstrated that cardiac FGFR1/KLB did not influence its systemic effects. Its reduction in cardiomyocytes suggested a heart-specific effect. In turn, the cardiac phenotype was due to local MFG-E8 rather than systemic influence. Nevertheless, since microvascular health is also central to the development of nephropathy, retinopathy, neuropathy, and other complications of T2DM, our findings highlight the potential relevance of MFG-E8 function beyond the heart.

Apart from its angiogenic properties, MFG-E8 also demonstrates protective effects against adverse cardiac remodeling under pressure overload stress, with phenotypes of less hypertrophic growth, fibrotic response, and apoptotic cell clearance.^24,25,61^ Consistently, rMFG-E8 treatment in our study reduced FGFR1/KLB deficiency-induced pathological hypertrophic markers, fibrosis, and apoptotic cells in the heart. Collectively, these results provide compelling evidence that MFG-E8 may serve dual roles in preserving angiogenesis and alleviating pathological remodeling in the diabetic heart. Therefore, dissection of the signaling pathways downstream of MFG-E8 may offer a promising therapeutic strategy for preserving cardiovascular function in diabetes.

### Clinical Implications of the FGFR1/KLB Pathway in the Heart

Our findings support that FGF21 acts as a direct ligand to activate the FGFR1/KLB pathway in cardiomyocytes. Although FGF21 has demonstrated therapeutic promise in HF, clinical and experimental observations indicate that elevated endogenous FGF21 in diabetes fails to mitigate cardiac dysfunction, likely due to myocardial FGF21 resistance.^62,63^ In line with the fact that KLB deficiency results in FGF21 insensitivity in the heart and worse remodeling post‑infarction,^64^ our study supports that FGFR1/KLB loss contributes to FGF21 resistance in the myocardium, blunting the beneficial effects of FGF21 during metabolic stress.

On the other hand, angiogenesis remains a central goal in myocardial repair.^65^ However, clinical trials using VEGF-based therapies display limited benefits, largely due to the complex process of angiogenesis that requires multiple signals and cell types.^66,67^ In contrast, preclinical studies demonstrated more encouraging outcomes using a combination treatment of FGF2 (fibroblast growth factor 2) and HGF (hepatocyte growth factor) in chronic HF,^68^ highlighting the feasibility of multitargeted approaches. In this context, our study elicits an unidentified mechanistic target involving the FGFR1/KLB axis, which may support the development of gene- and cell-based therapies to enhance endothelial angiogenesis. Furthermore, we used different human stem cell-derived cardiomyocytes to achieve valuable human-related evidence. Similar responses in strains ensure a nongenetic basis for cardiac signaling pathway alterations, as different genetic backgrounds did not alter the major observations we hypothesized. Thus, our findings give reference for the development of pharmacological modulators of the FGFR1/KLB pathway and the potential to perform clinical trials using these agents in the future.

### Sex Differences in Myocardial Microvasculature Maintenance

Sex-based differences play an under-appreciated role in cardiac repair, particularly in microvascular biology. Several studies suggest the overall angiogenic responses tend to be less robust in females, giving rise to a greater predisposition to cardiac dysfunction.^69–73^ Of note, endothelial injury in diabetic females is more severe and associated with diastolic dysfunction.^74^ In our study, disruption of cardiomyocyte FGFR1/KLB induced microvascular impairment in male and female hearts, while maintaining the intact signaling displayed beneficial effects in both sexes, supporting FGFR1/KLB-mediated mechanisms as promising treatment targets. However, single treatment of KLB restoration delivered better outcomes in males during prolonged diabetic stress, which indicates KLB may also act as a co-factor for other receptors in a sex-dependent manner.

Additionally, rMFG-E8 treatment mitigated the progression of cardiac dysfunction in both male and female FGFR1- or KLB-knockouts, supporting that MFG-E8 is sufficient to facilitate microvascular health as cardioprotective. Although restoring CEBPβ showed beneficial effects on cardiac function in FGFR1^cKO^ or KLB^cKO^ mice without sex stratification, it was less effective in females in terms of isovolumic relaxation time, despite notable enhancements in angiogenesis and cardiac remodeling. A possibility is that CEBPβ, as a transcription factor, may mediate other downstream targets that could likely be involved in estrogen-related signals in female hearts, particularly when its amount is higher than the levels physiologically needed. Further identification of CEBPβ downstream effectors and evaluation of treatment dose may provide more insights into optimal treatment by targeting CEBPβ and coordinating other molecular processes in females.

## Conclusions

In summary, our results establish FGFR1/KLB signaling in cardiomyocytes as an essential regulator of cardiomyocyte EC crosstalk through MFG-E8. Sustaining cardiac FGFR1/KLB preserves angiogenic capacity and adaptive cardiac remodeling, therefore alleviating cardiac dysfunction, and highlighting its potential as a preventive and therapeutic target for diabetes-associated cardiovascular dysfunction in both sexes.

## Article Information

### Acknowledgments

The authors would like to thank the staff working in different departments of the University of Manchester for providing valuable assistance throughout this project. The authors sincerely thank Steven Kliewer (University of Texas Southwestern Medical Center, United States) for providing one of the key models, the *Klb*^fl/fl^ model. The authors express our gratitude to Steven Marsden, Peter March, and James Bagnall of the Bioimaging Facility for their technical training on fluorescent microscope imaging. The authors also appreciate Bharatkumar Rash, Claire Morrisroe, Andy Hayes, Ian Donaldson, Rachel Scholey, and Leo Zeff in the Genomic Technologies Facility and Bioinformatics Facility for their technical support regarding RNA sequencing and data analysis. The authors would like to acknowledge Stacey Warwood, James Allsey, Melissa Kidd, and David Knight of the Mass Spectrometry Facility for their professional advice, sample processing, conducting, and analyzing proteomics. The authors also thank Alis Hales and Antony Adamson of the Genome Editing Unit Facility for generating mutant plasmids. Furthermore, we thank Samantha Forbes and Aleksandr Mironov of the Electron Microscopy Core Facility for TEM technical training and support. The authors are also grateful to Yiming Zhang from Boston Children’s Hospital for assistance with method development and writing during revision. Finally, we appreciate Charlotte Beck, Victoria Perks, Sarah Lawton, and Christopher Macaulay of the Biological Services Facility for their help in training, handling, and animal maintenance.

### Author Contributions

The project was conceptualized by W. Liu. S.R. Gare contributed significantly to experimental design, animal work, molecular studies, data acquisition, analysis, and interpretation. N.H. Alatawi contributed to animal functional assessment and additional molecular data acquisition during revision. X. Chen and J. Zhang helped with animal work and performed additional molecular assessments. C. Ross and O. Fonseka helped with some animal work. Y. Han, X. Zhao, Y. Liu, and T. Wang helped with the culture of hiPSCs, human embryonic stem cell-derived cardiomyocytes, and NRCMs. A. Ruiz-Velasco and N. Kaur did initial screenings and provided pilot data. R.R.E. Abouleisa and T.M.A. Mohamed provided human heart tissue samples. S.S. Hille and O.J. Müller prepared key AAVs and reviewed the article. S. Abraham and G. Howell provided mentorship. E.J. Cartwright reviewed the in vivo studies and the article and provided financial support as Head of the Division. W. Liu drafted this article with contributions from S.R. Gare, C. Ross, and O. Fonseka. X. Chen, J. Zhang, and Y. Han made major contributions to data checking and analyses during revision. M.K. Rutter provided mentorship and reviewed the article. B.D. Keavney, supported by a British Heart Foundation Personal Chair, also provided mentorship for the work.

### Disclosures

T.M.A. Mohamed holds equities in Tenaya Therapeutics. The other authors report no conflicts.

### Supplemental Material

Supplemental Methods

Major Resources Table

Tables S1–S15

Figure S1–S23

References 75–86

## Supplementary Material


