# Supplementary material for: FGFR1/KLB-MFG-E8 Maintains Microvascular Integrity and Adaptive Cardiac Remodeling
Source: Circ Res. 2026 Sep 29;139(10):e327950. doi: 10.1161/CIRCRESAHA.125.327950 (PMC13633393; doi:10.1161/CIRCRESAHA.125.327950)

Supplemental data

Raw immunoblot images

Red frames indicate the representative figure in manuscript and used for quantification

Yellow frames indicate the additional samples used for quantification, with group labelled

Figure 1 – Full unedited representative and quantification immunoblots

Figure 1D

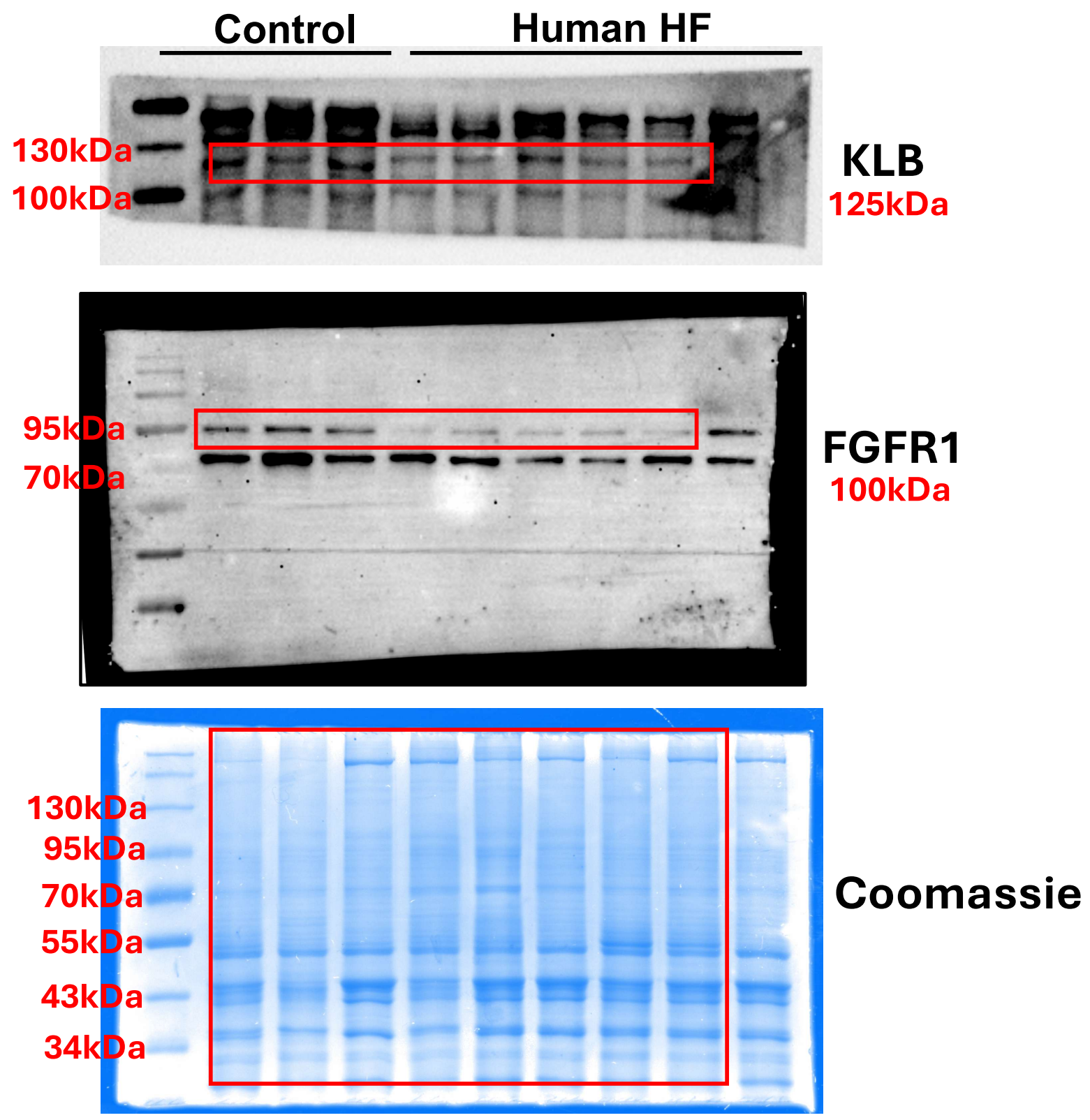

Figure 1G

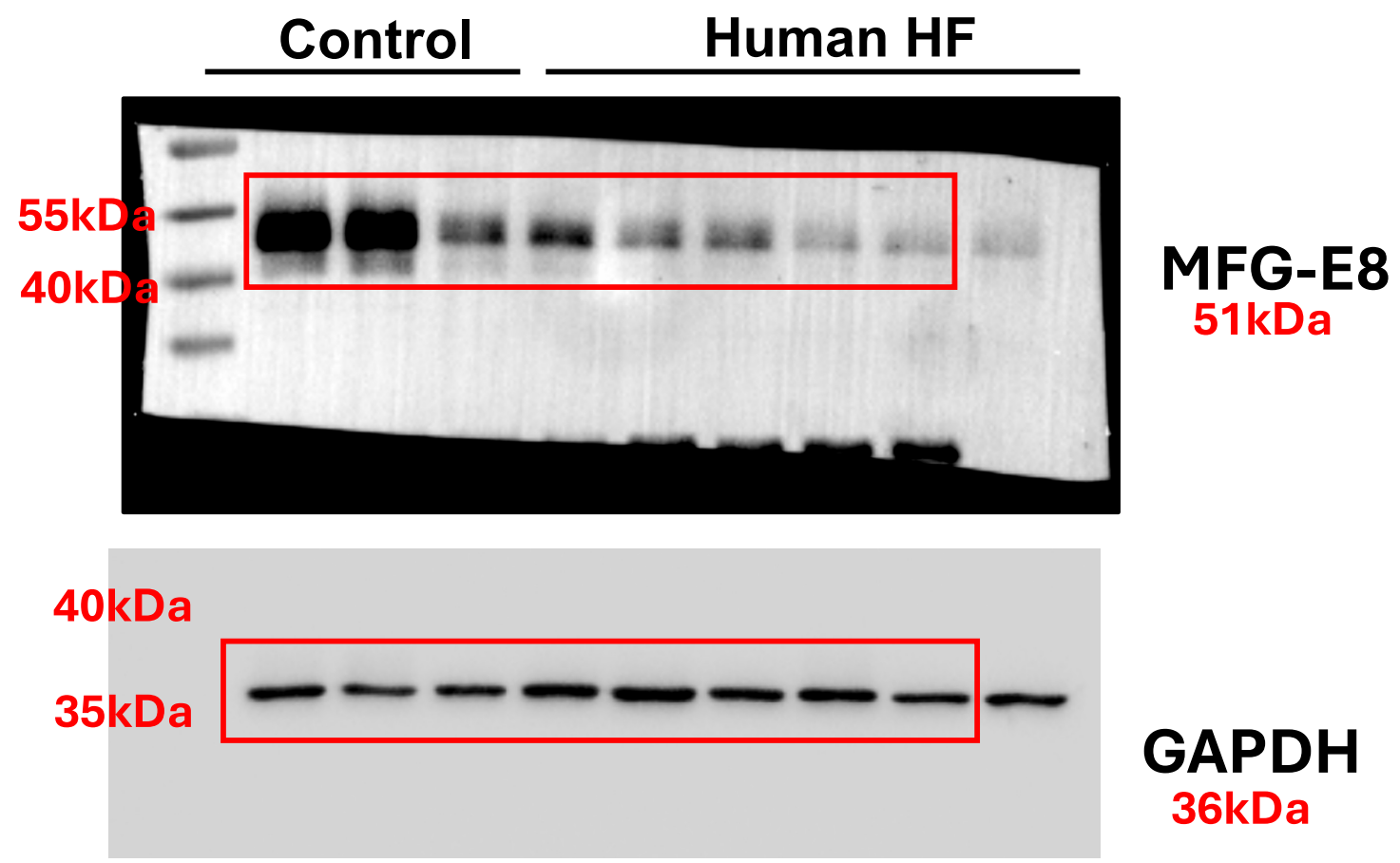

Figure 2 – Full unedited representative and quantification immunoblots

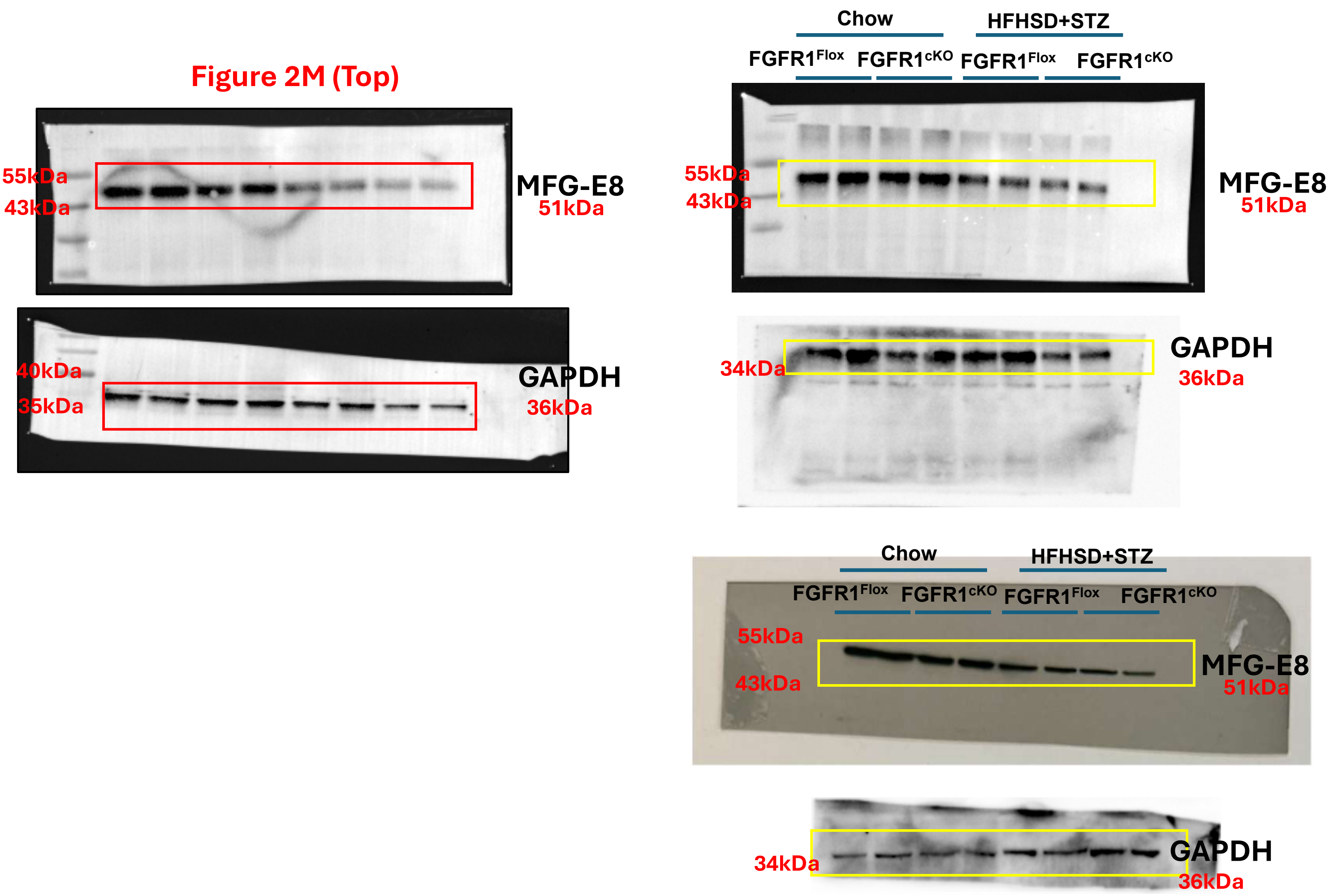

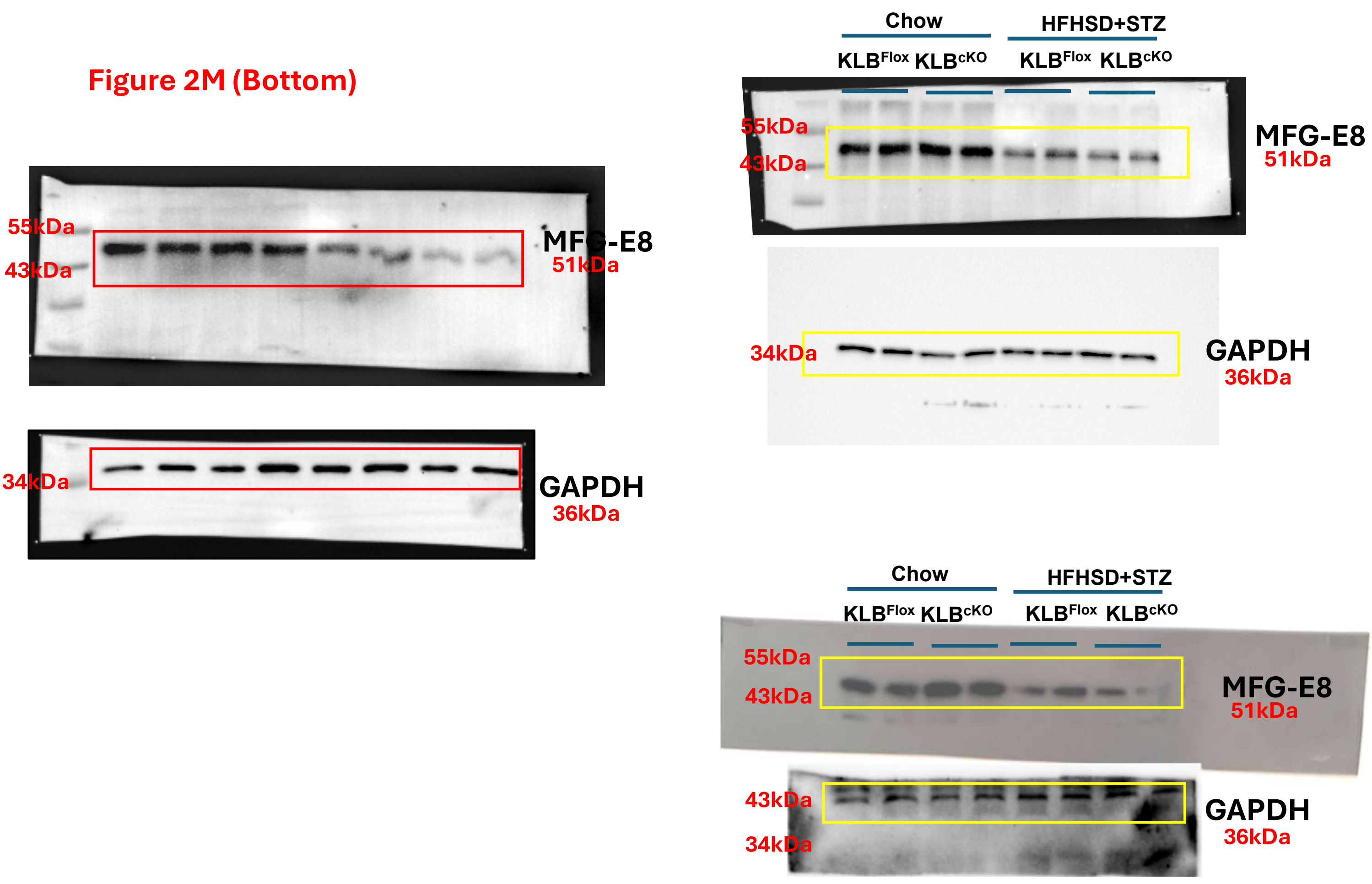

Figure 3 – Full unedited representative and quantification immunoblots

Figure 3I

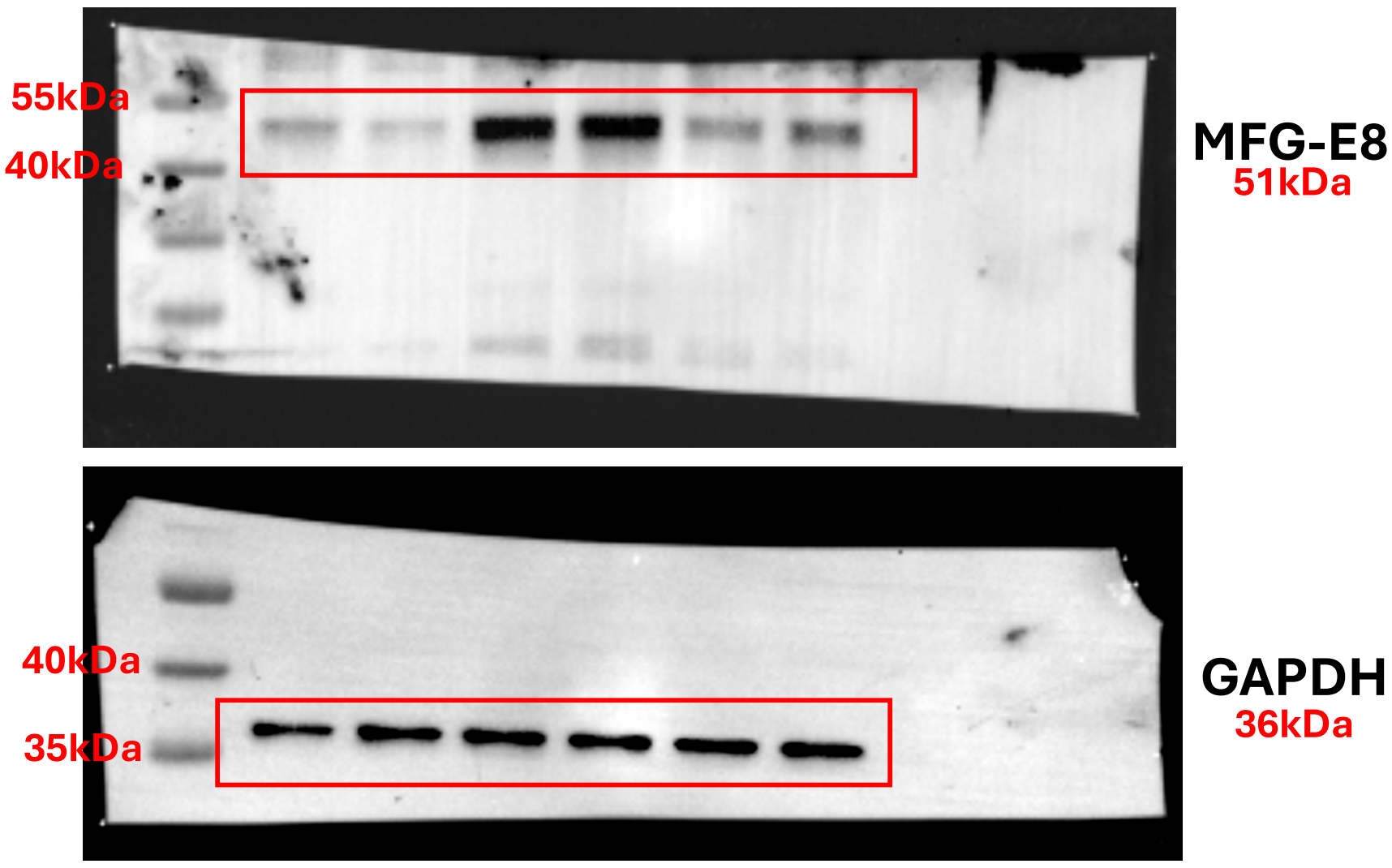

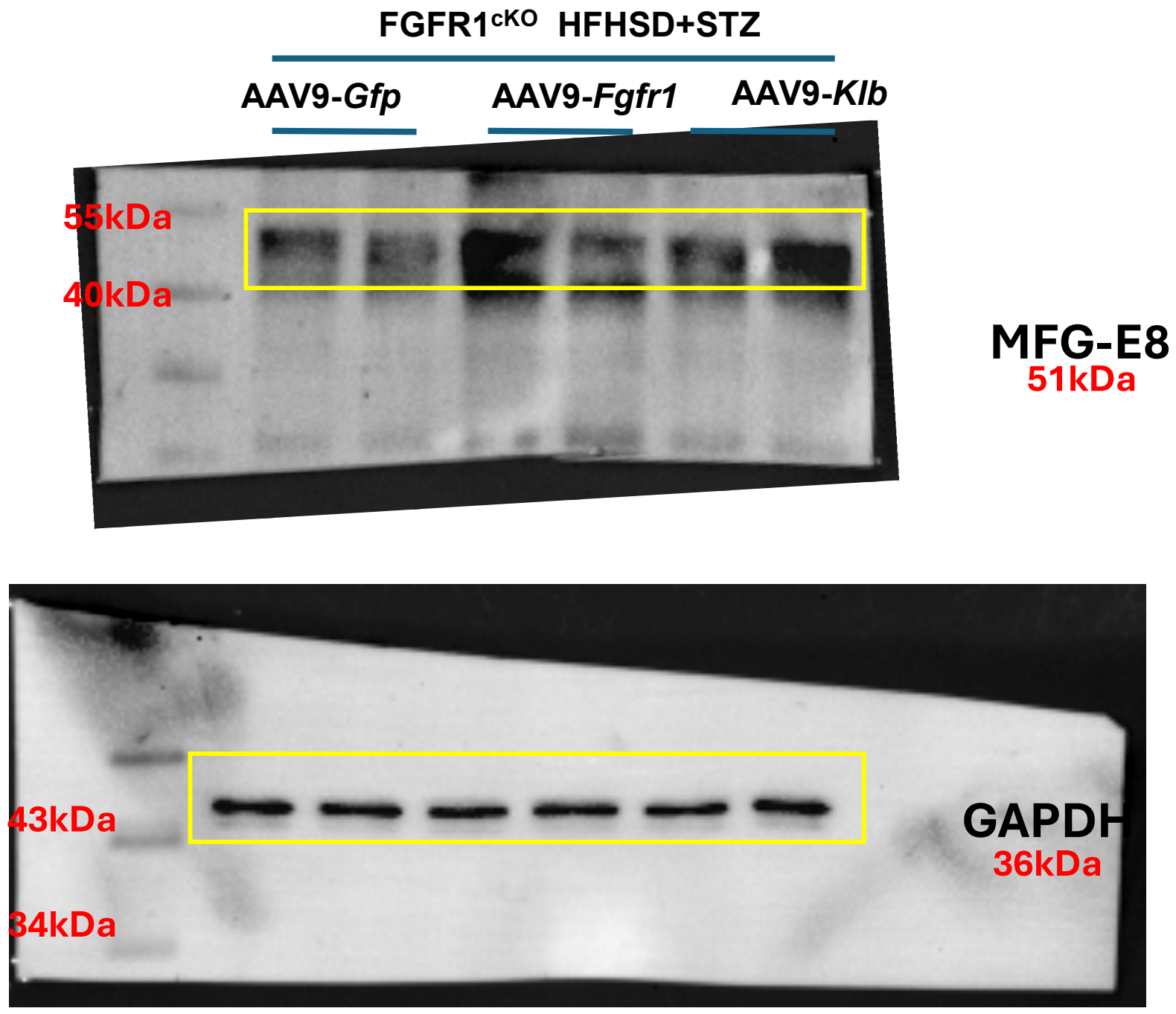

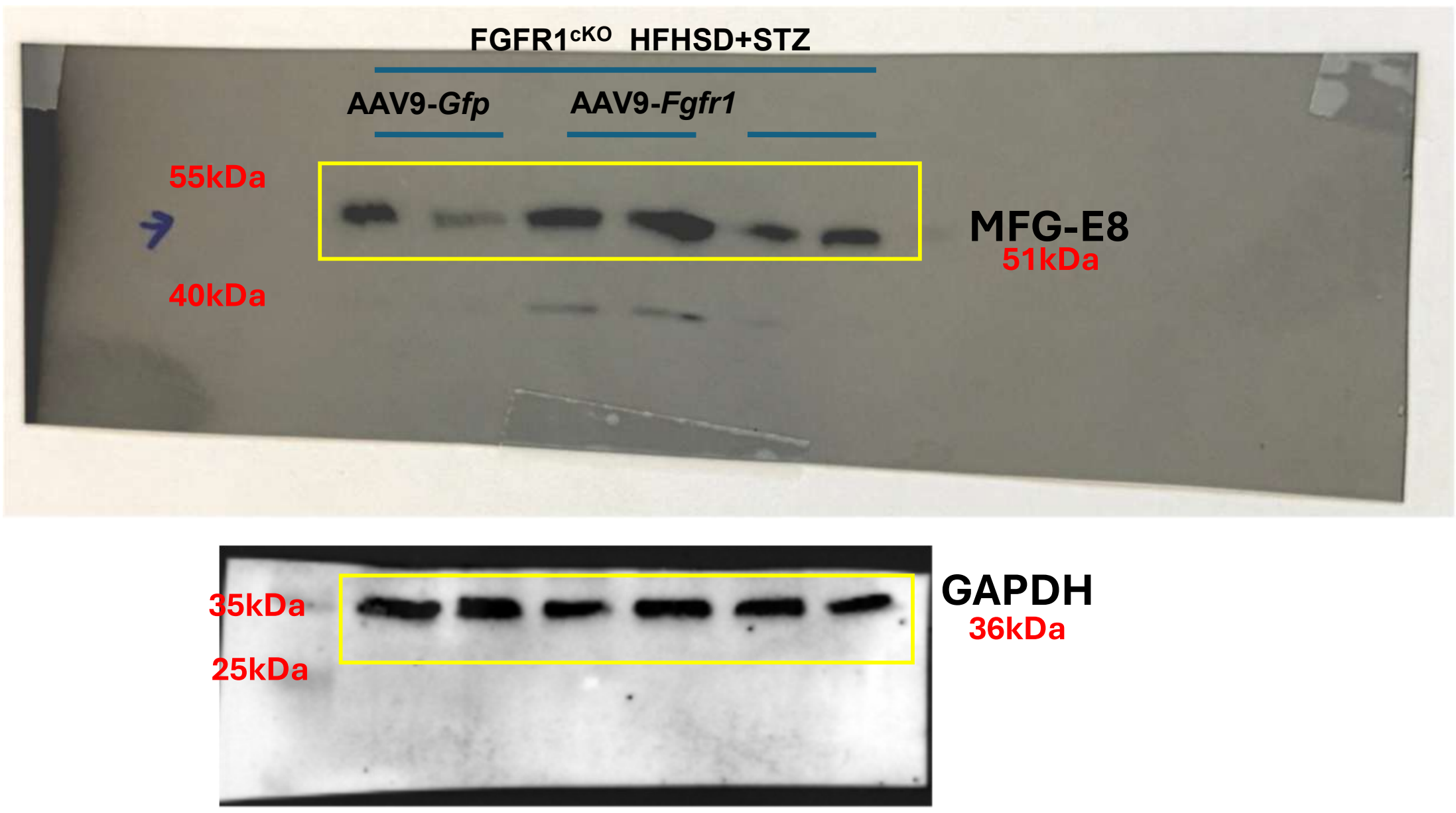

Figure 3 – Full unedited representative and quantification immunoblots continued

Figure 3K

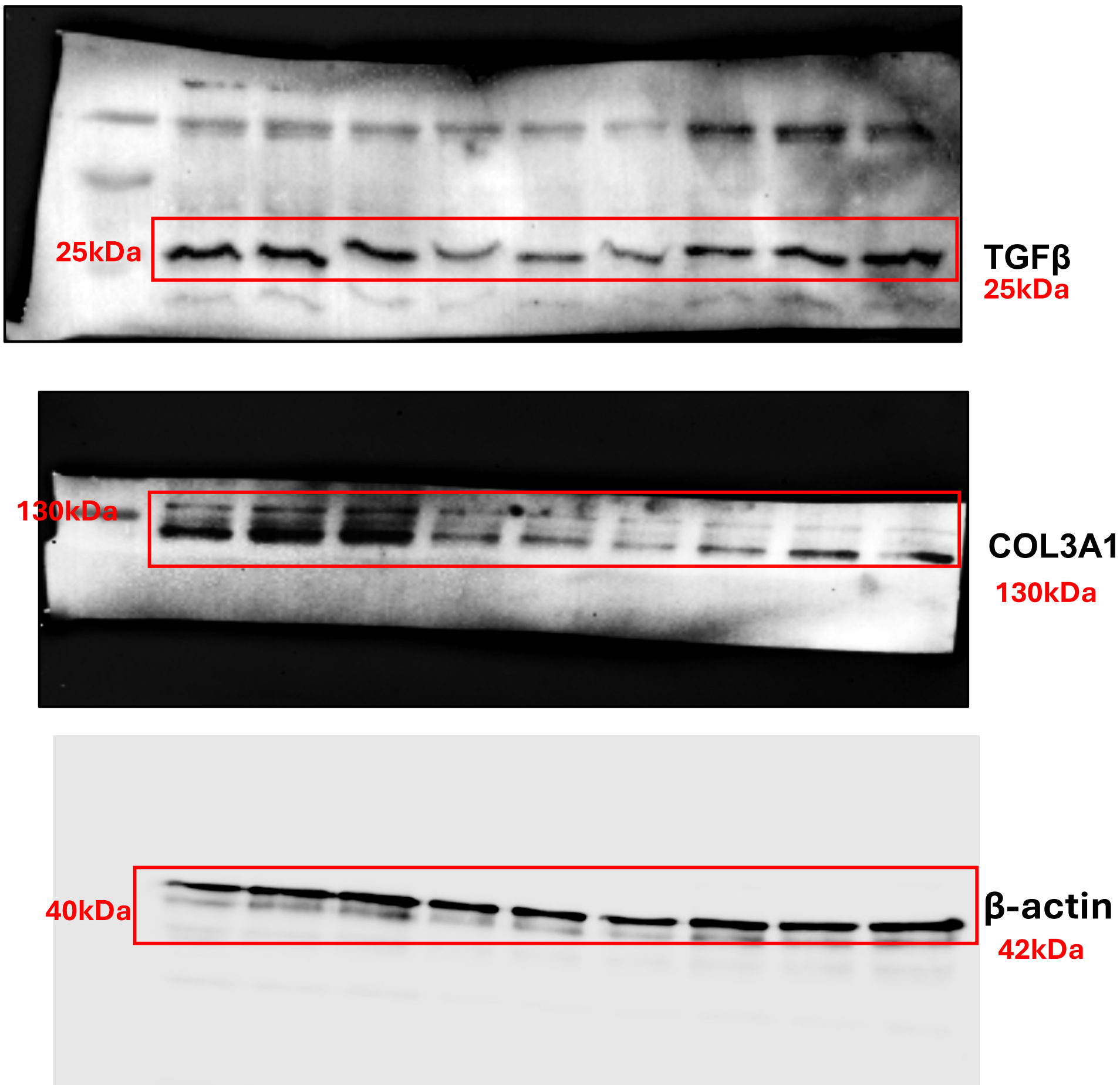

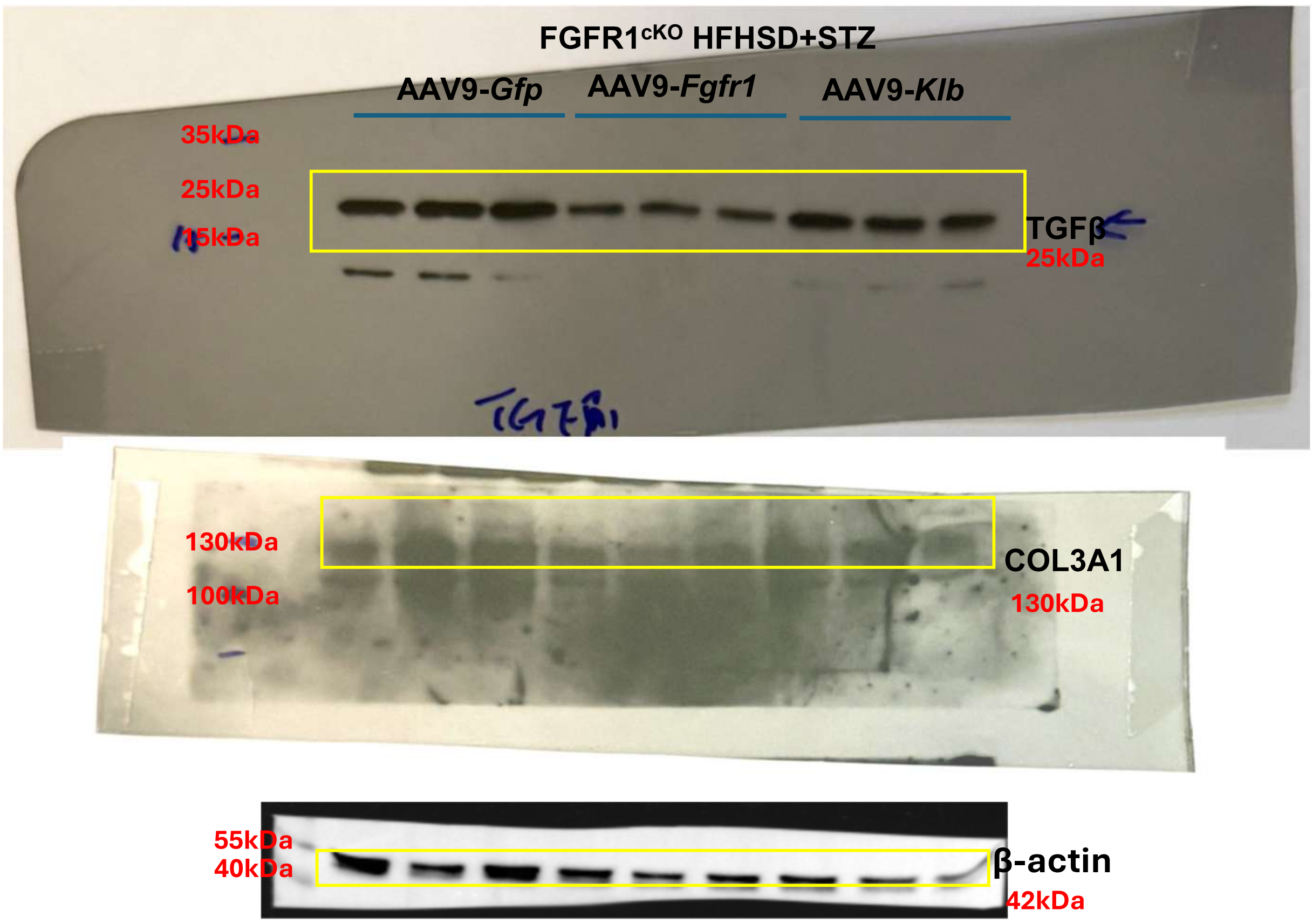

Figure 5 – Full unedited representative and quantification immunoblots

Figure 5O

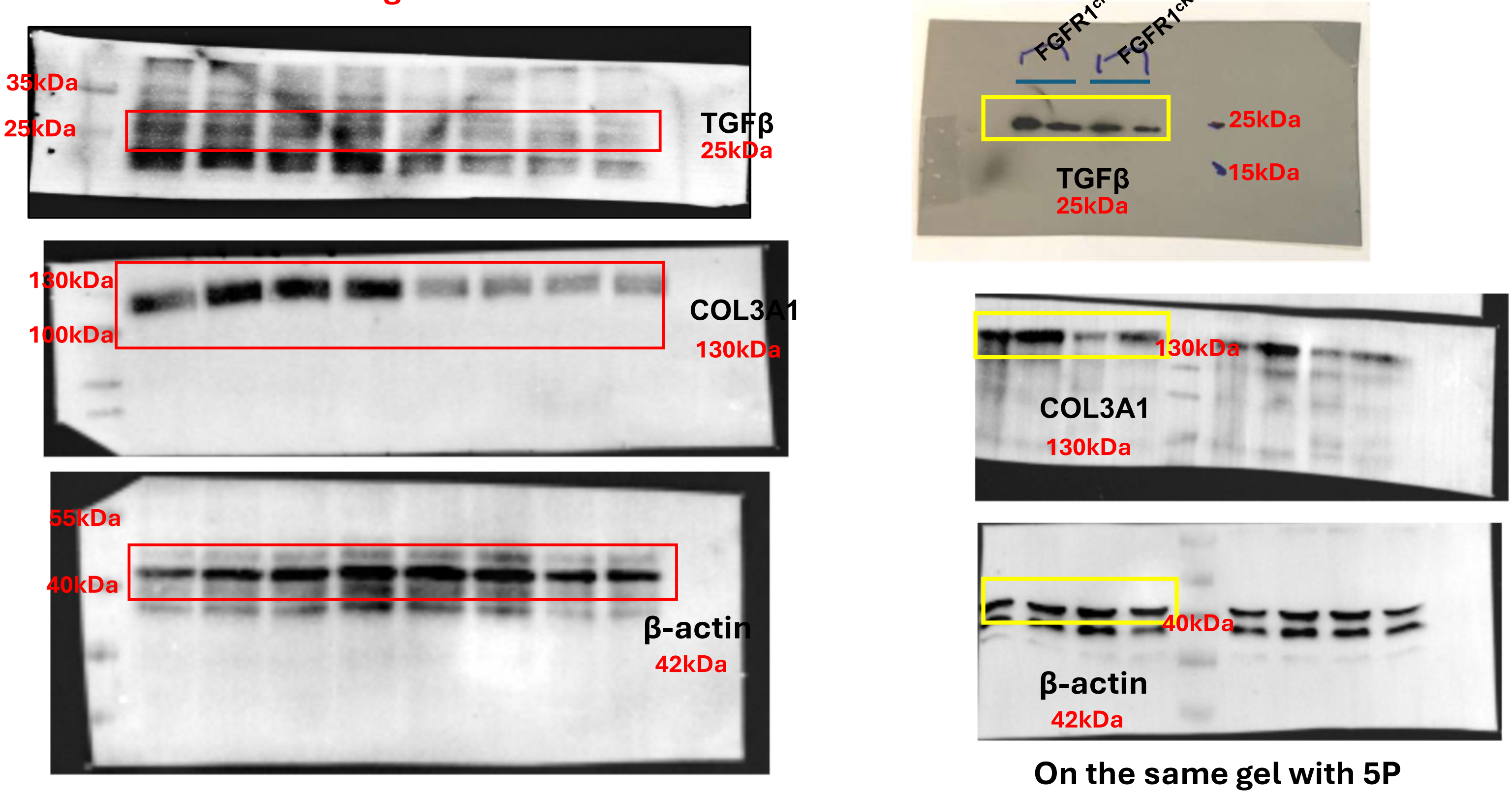

On the same gel with 5P

Figure 5P

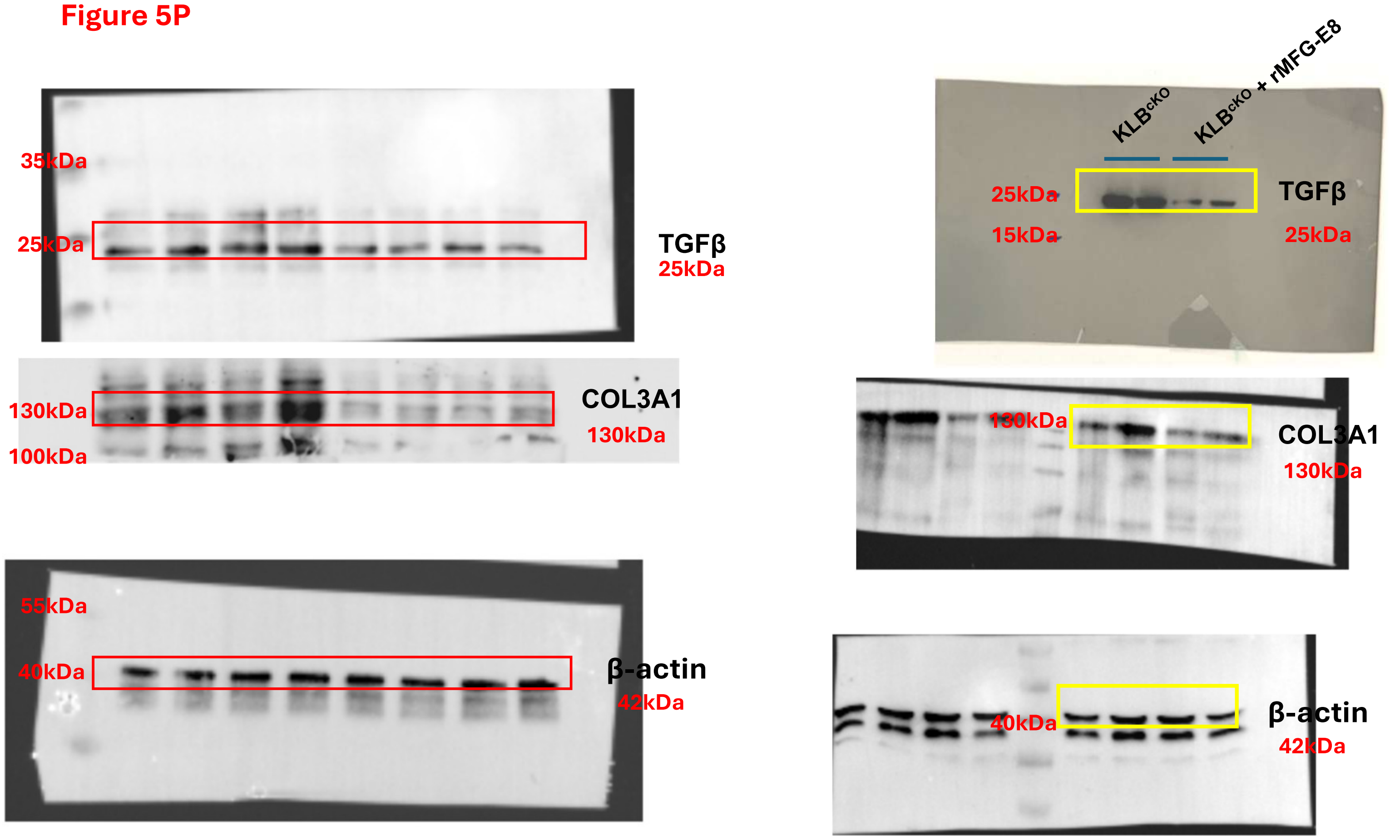

On the same gel with 5C

Figure 6 – Full unedited representative and quantification immunoblots

Figure 6E (top)

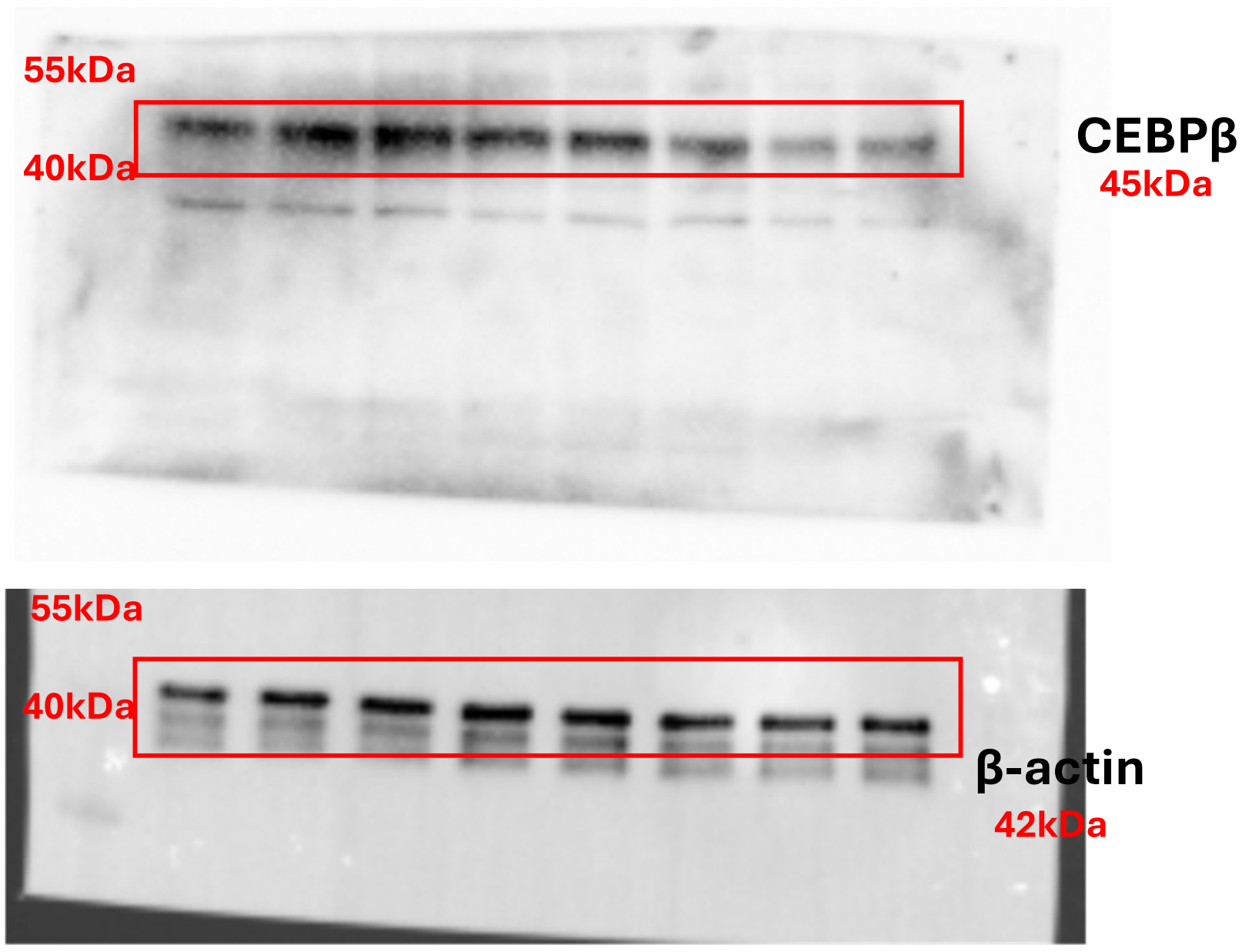

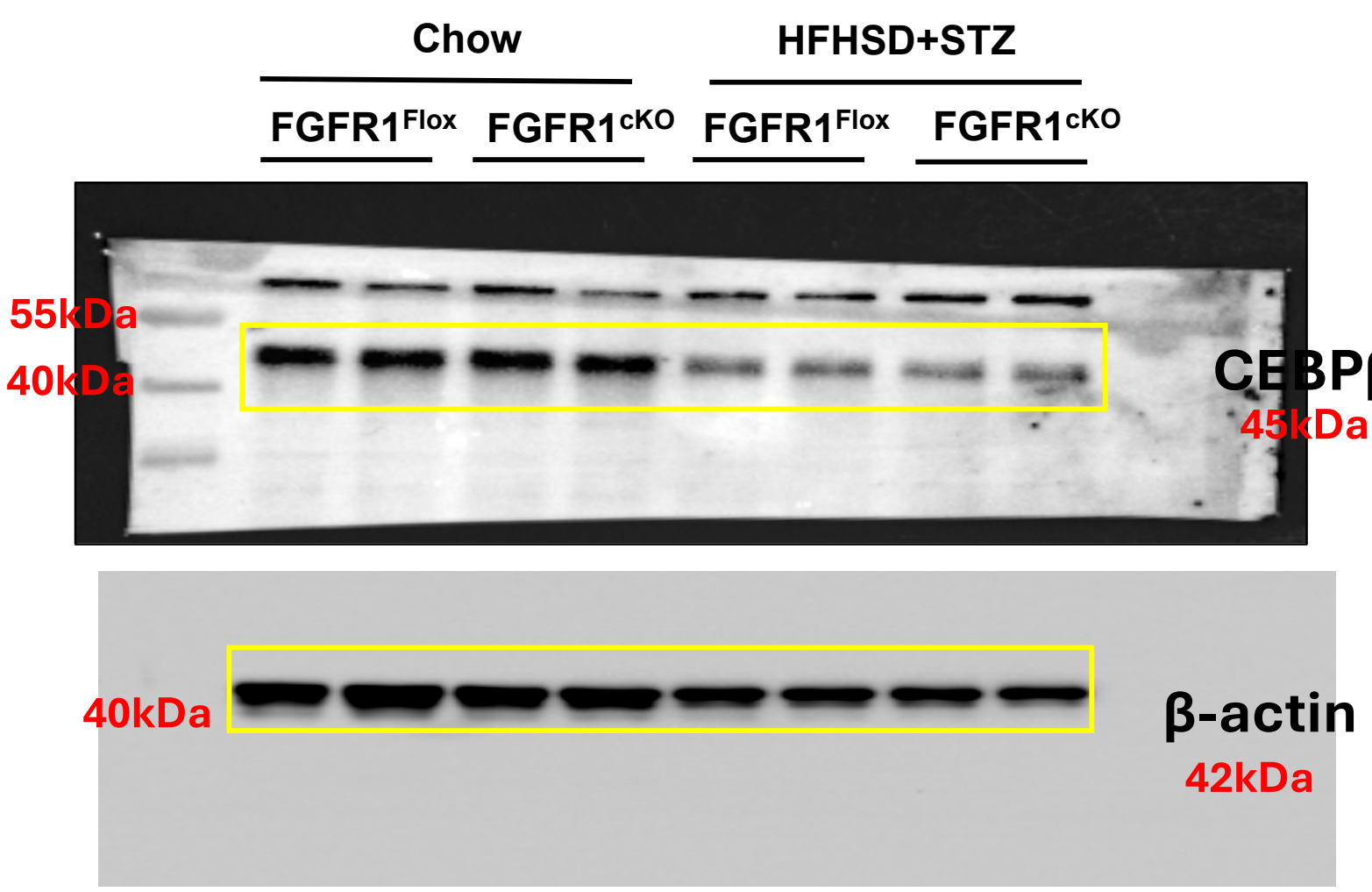

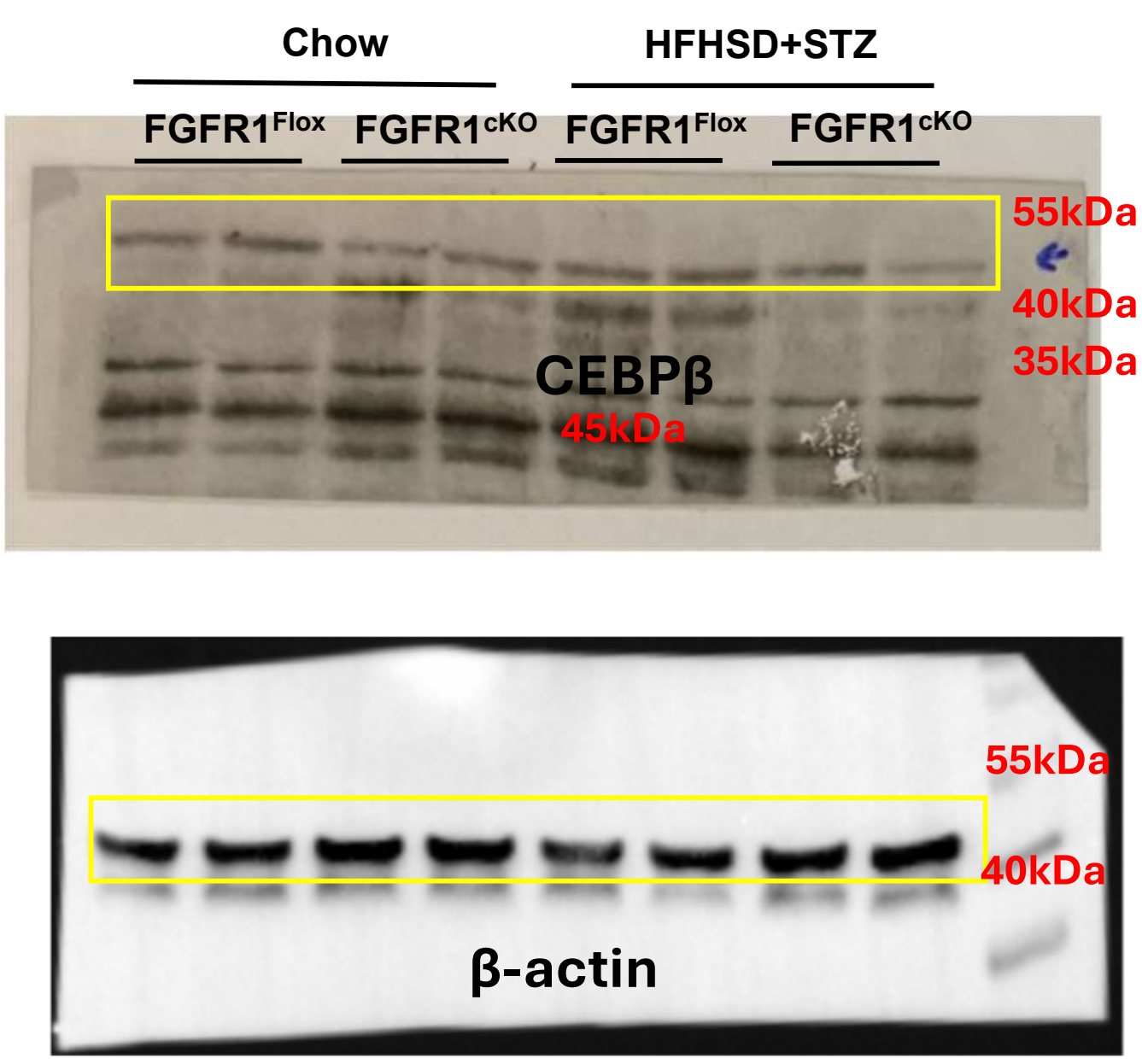

Figure 6 – Full unedited representative and quantification immunoblots

Figure 6E (Bottom)

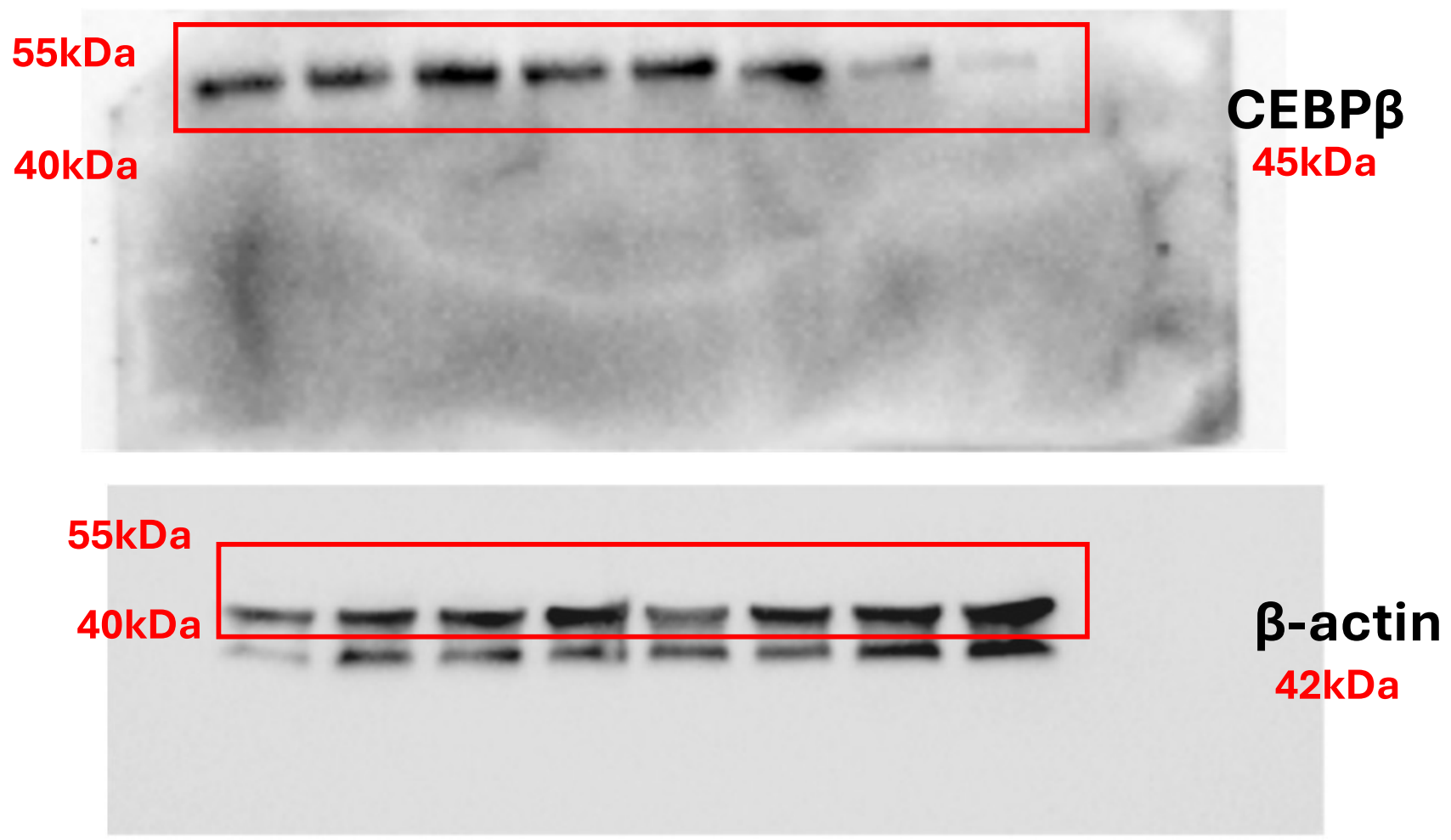

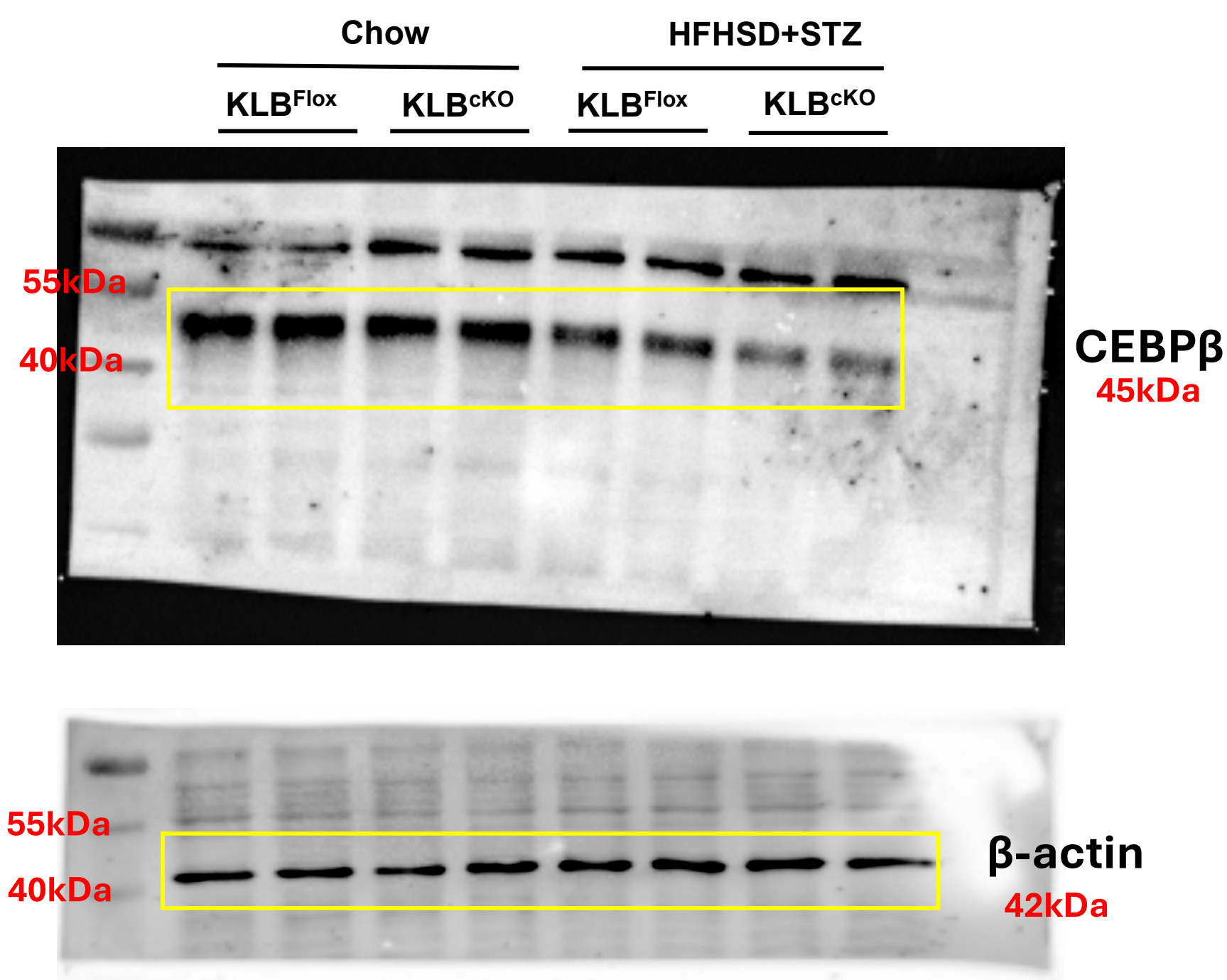

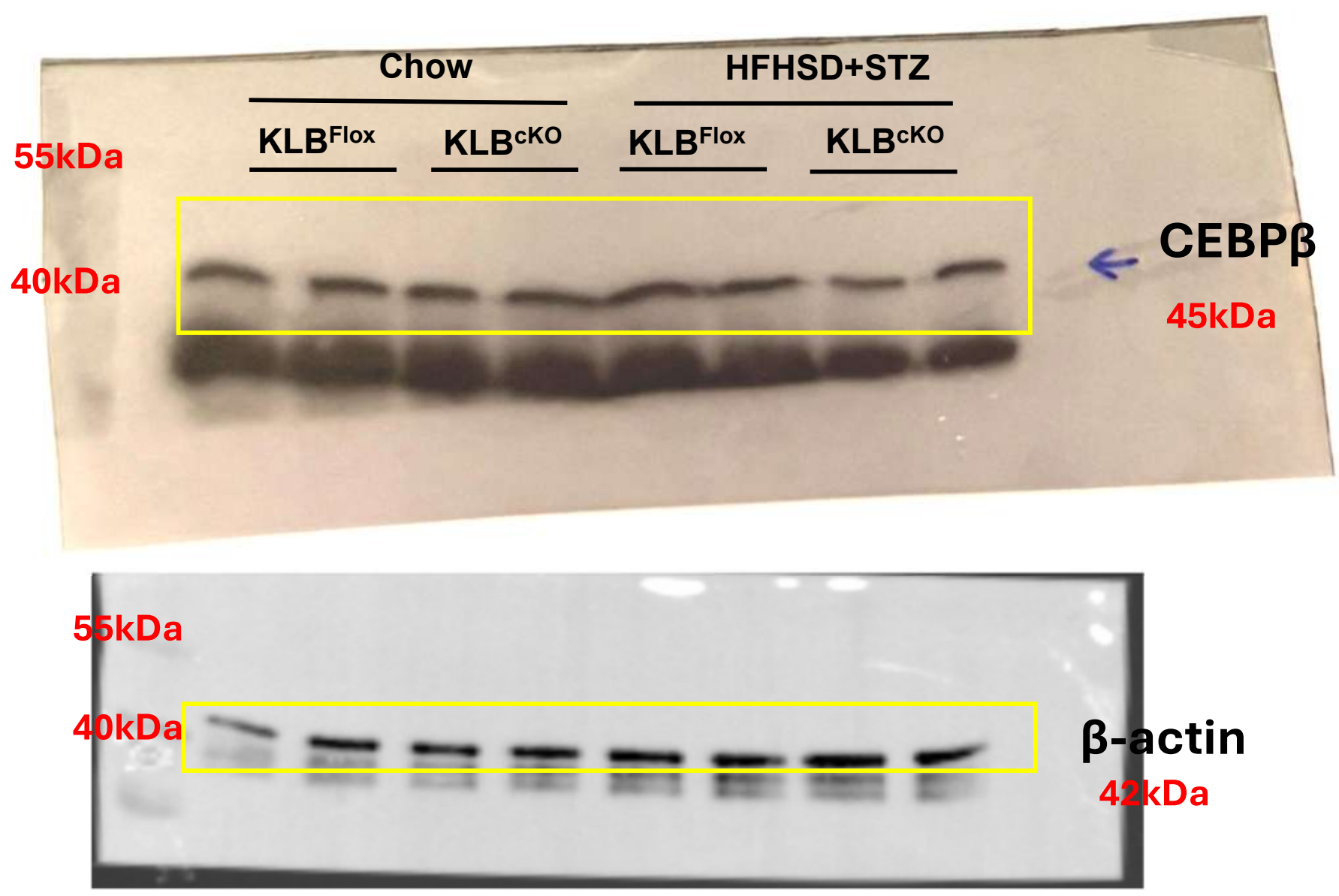

Figure 7 – Full unedited representative and quantification immunoblots

Figure 7O

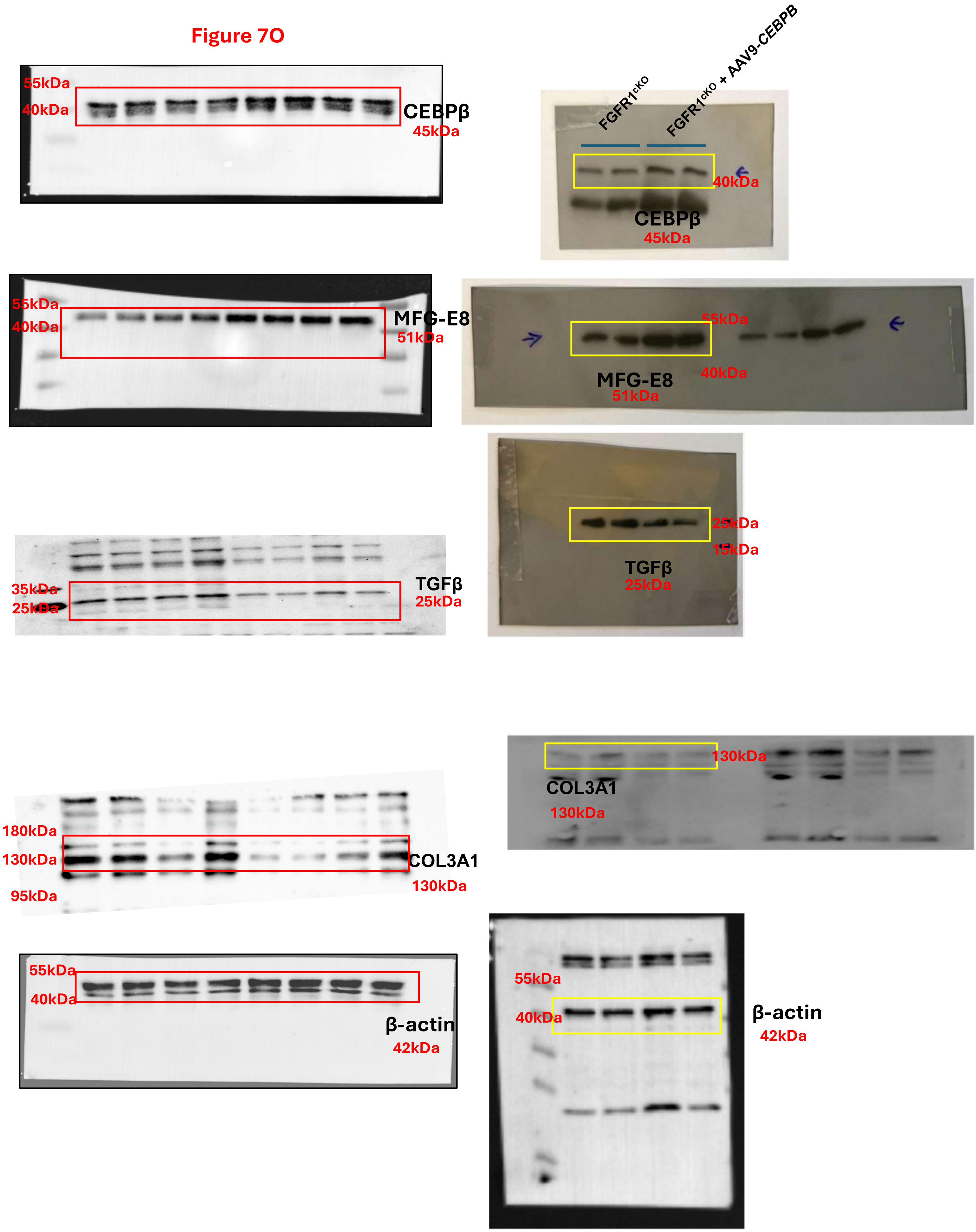

On the same gel with 7P

Figure 7 – Full unedited representative and quantification immunoblots

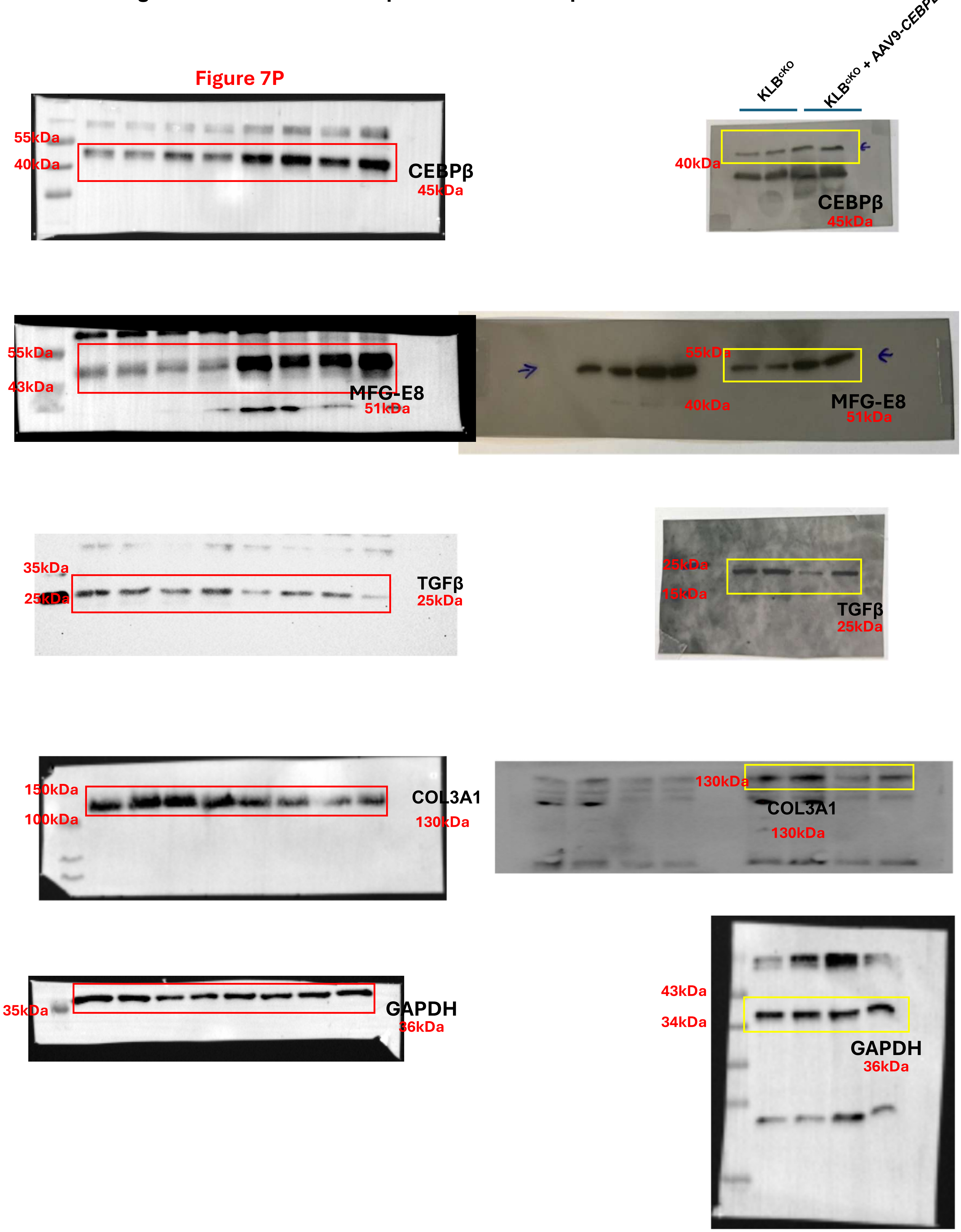

On the same gel with 7O

Figure 8 – Full unedited representative and quantification immunoblots

Figure 8A

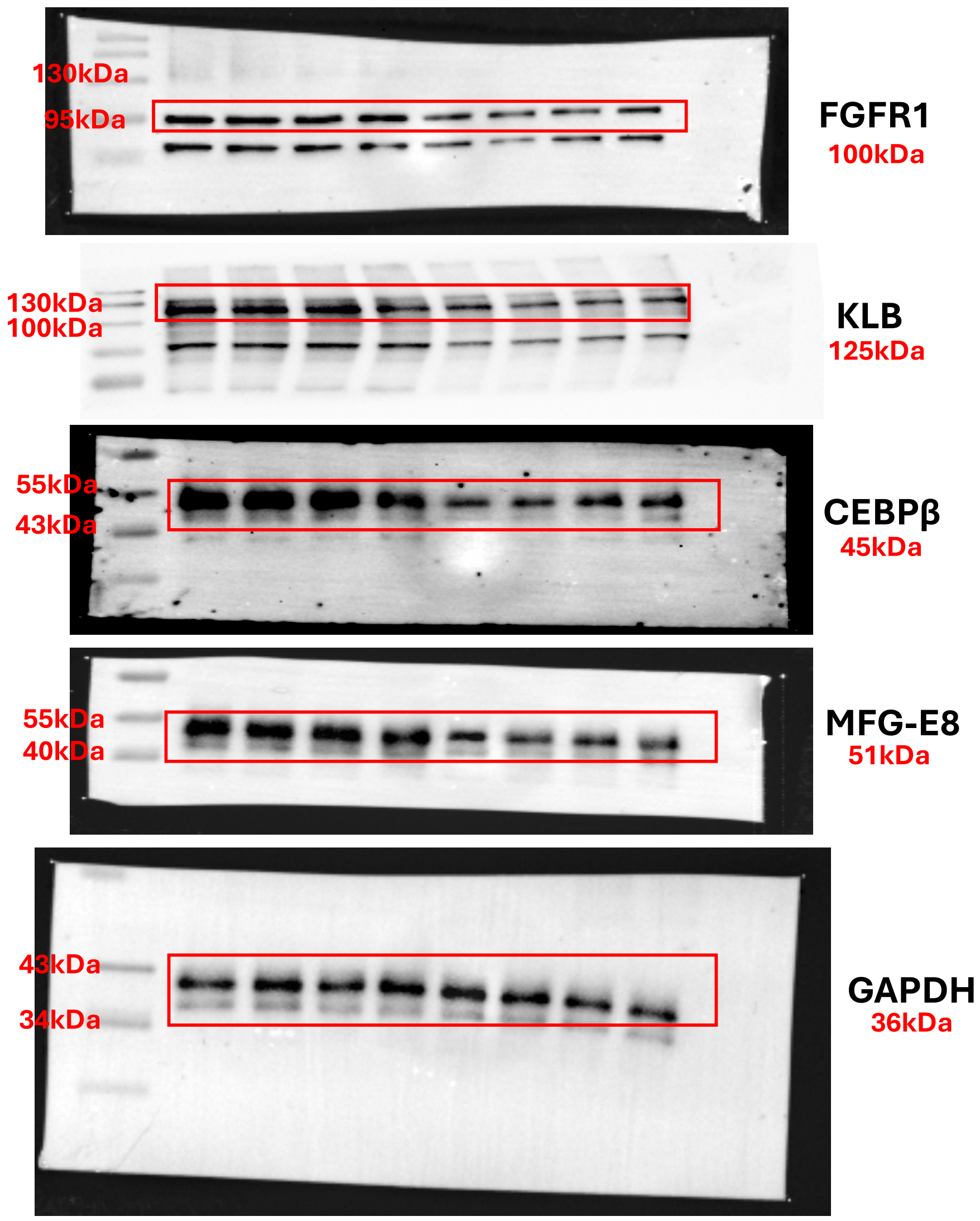

Figure 8C

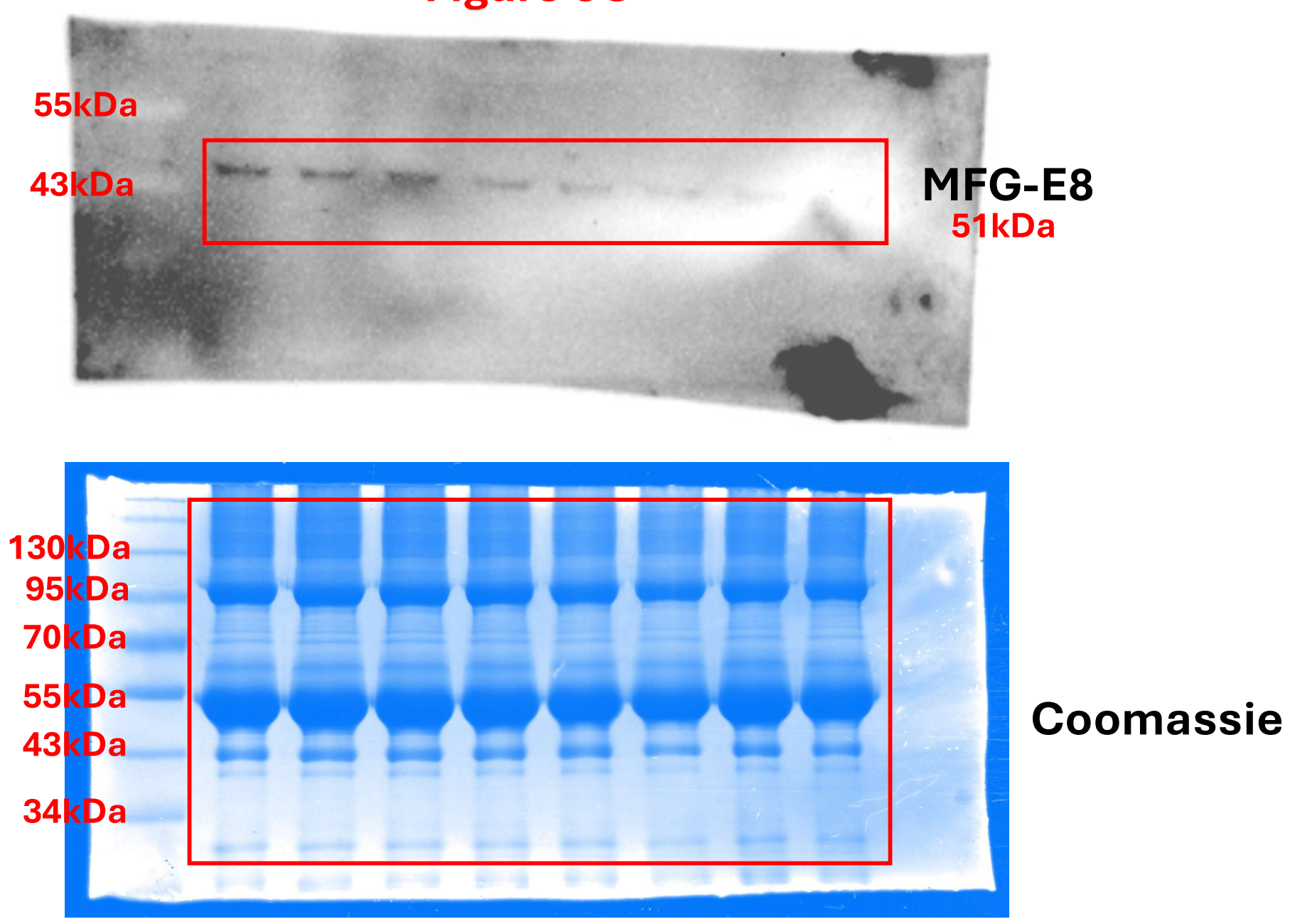

Figure 8 – Full unedited representative immunoblots continued

Figure 8G

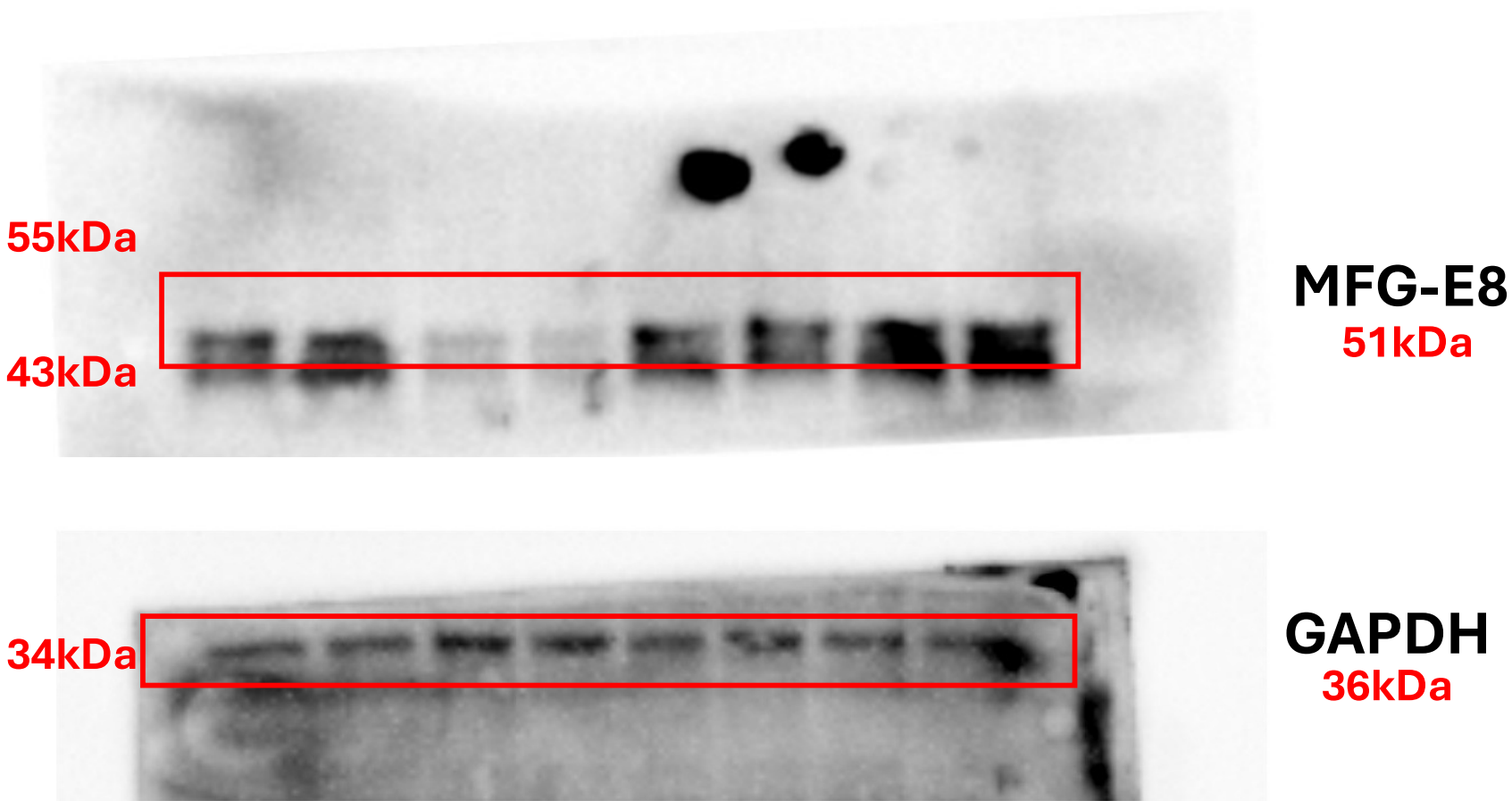

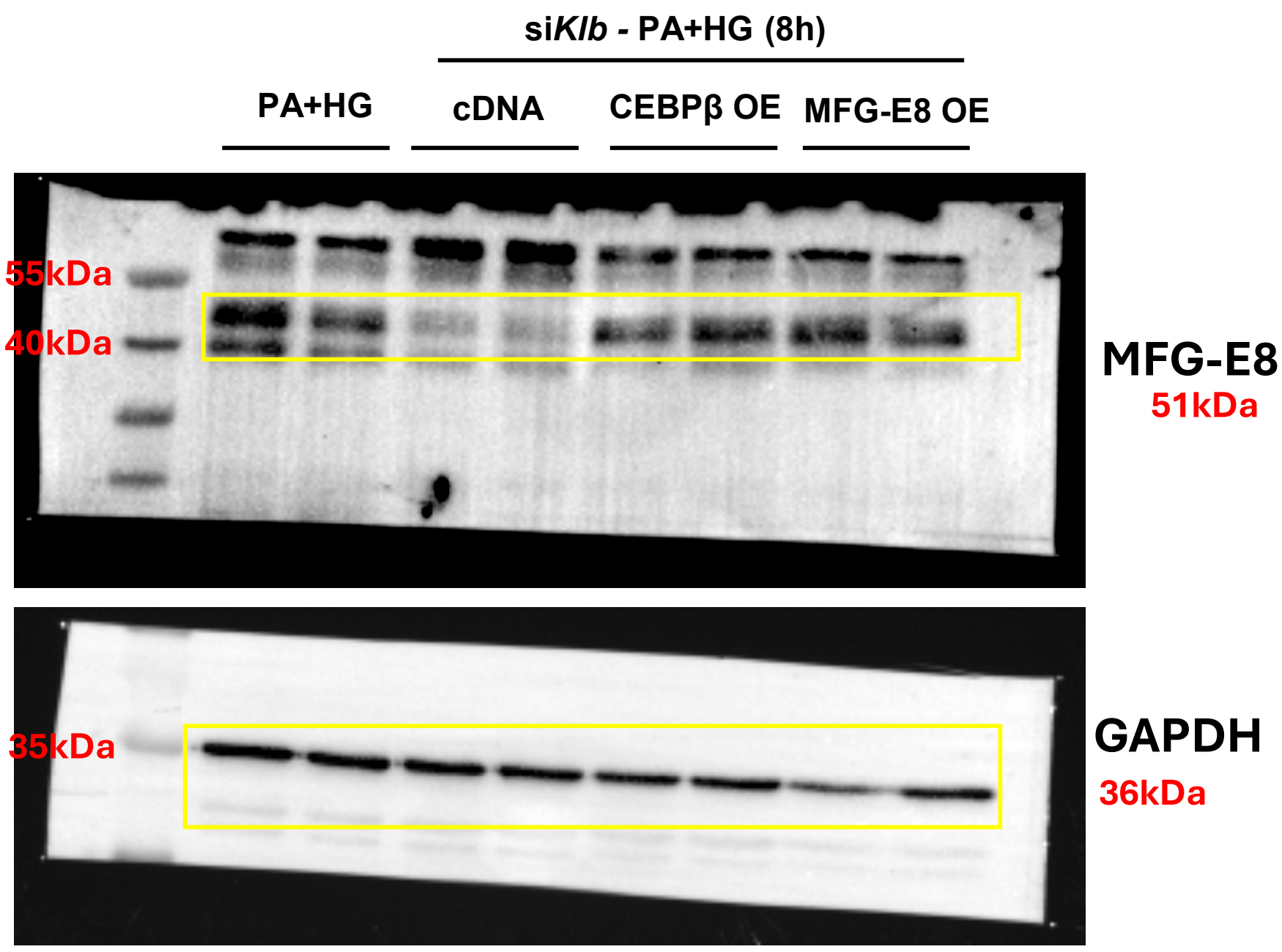

Figure 8 – Full unedited representative and quantification immunoblots

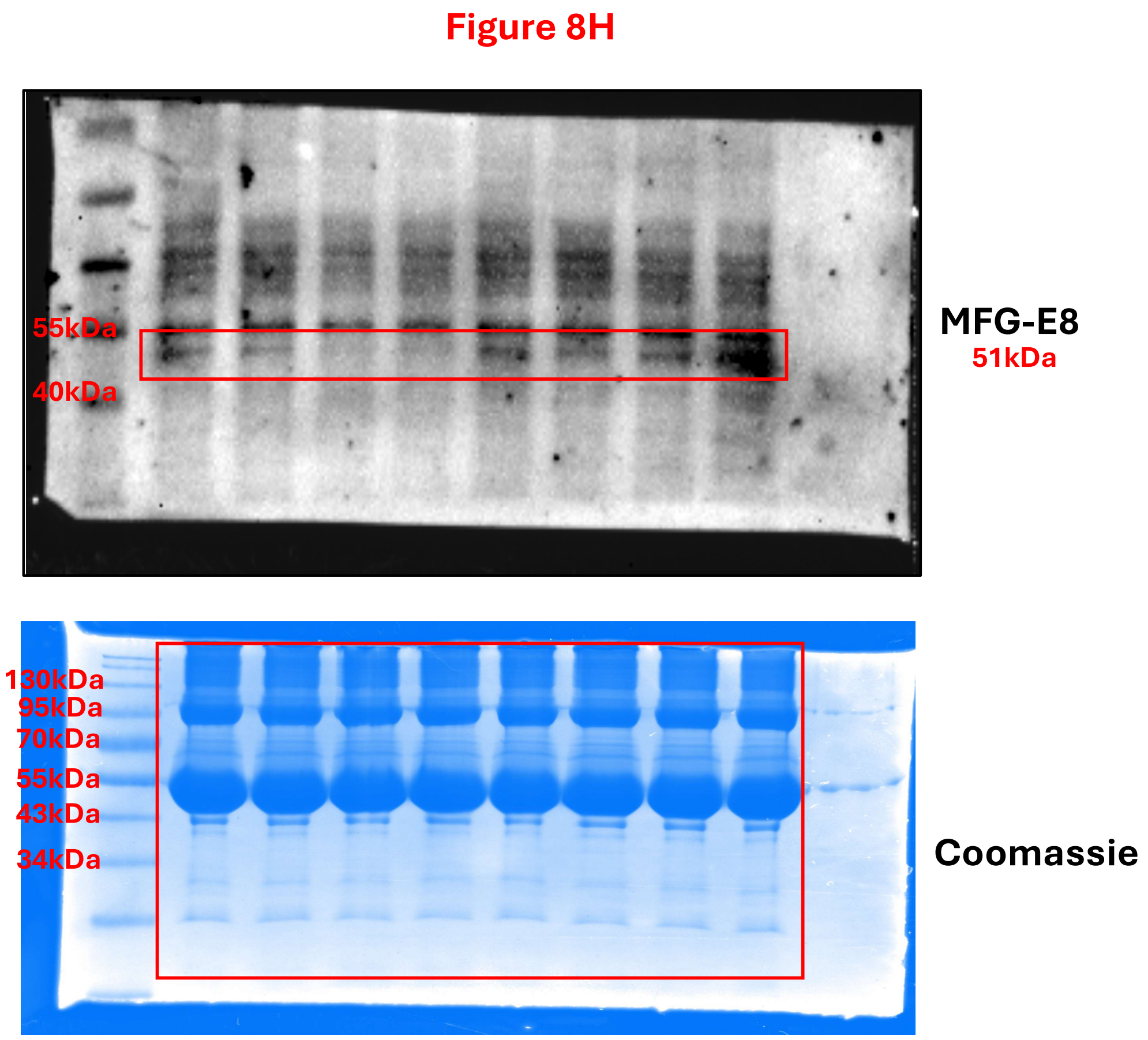

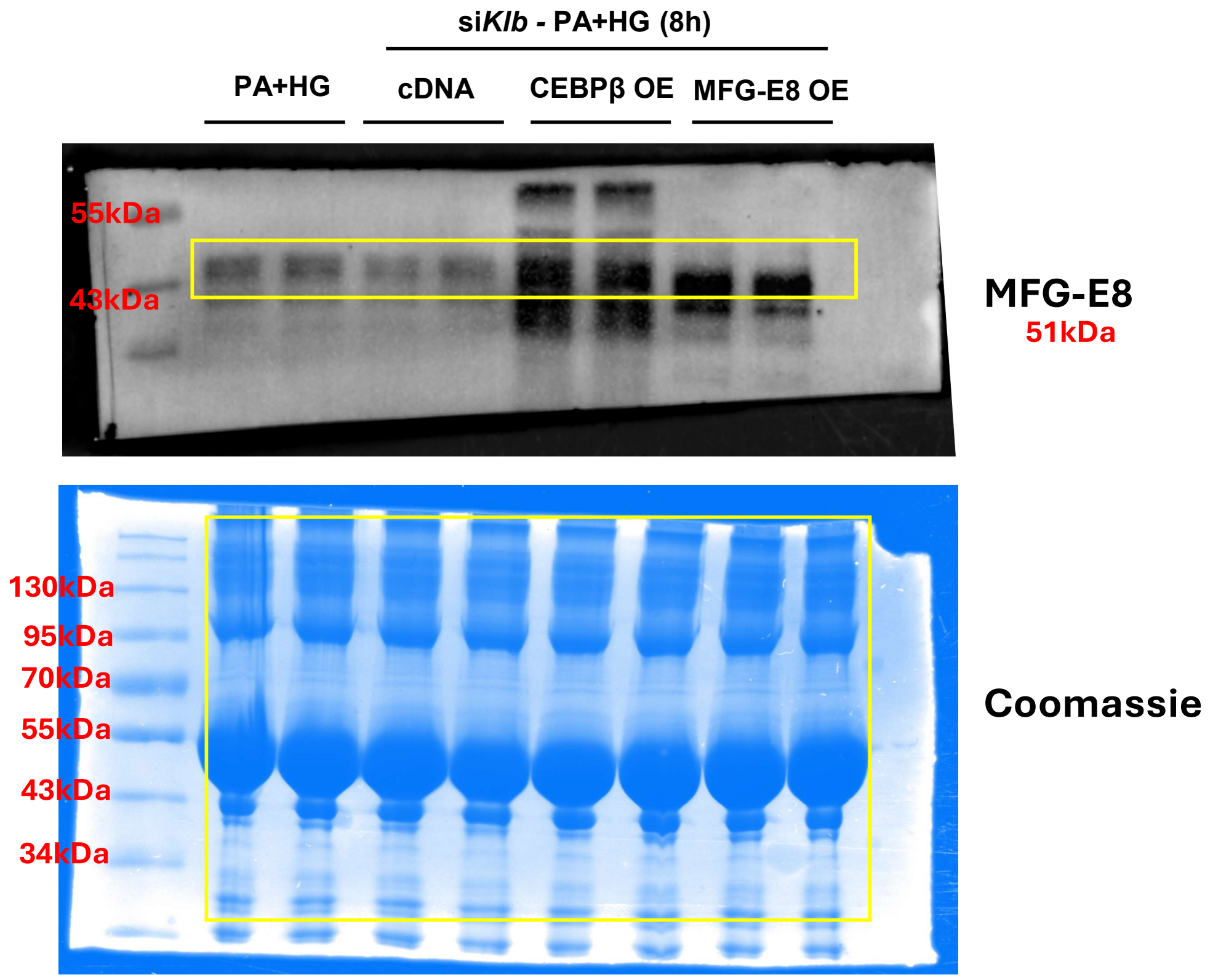

Figure S1 – Full unedited representative immunoblots

Figure S1B

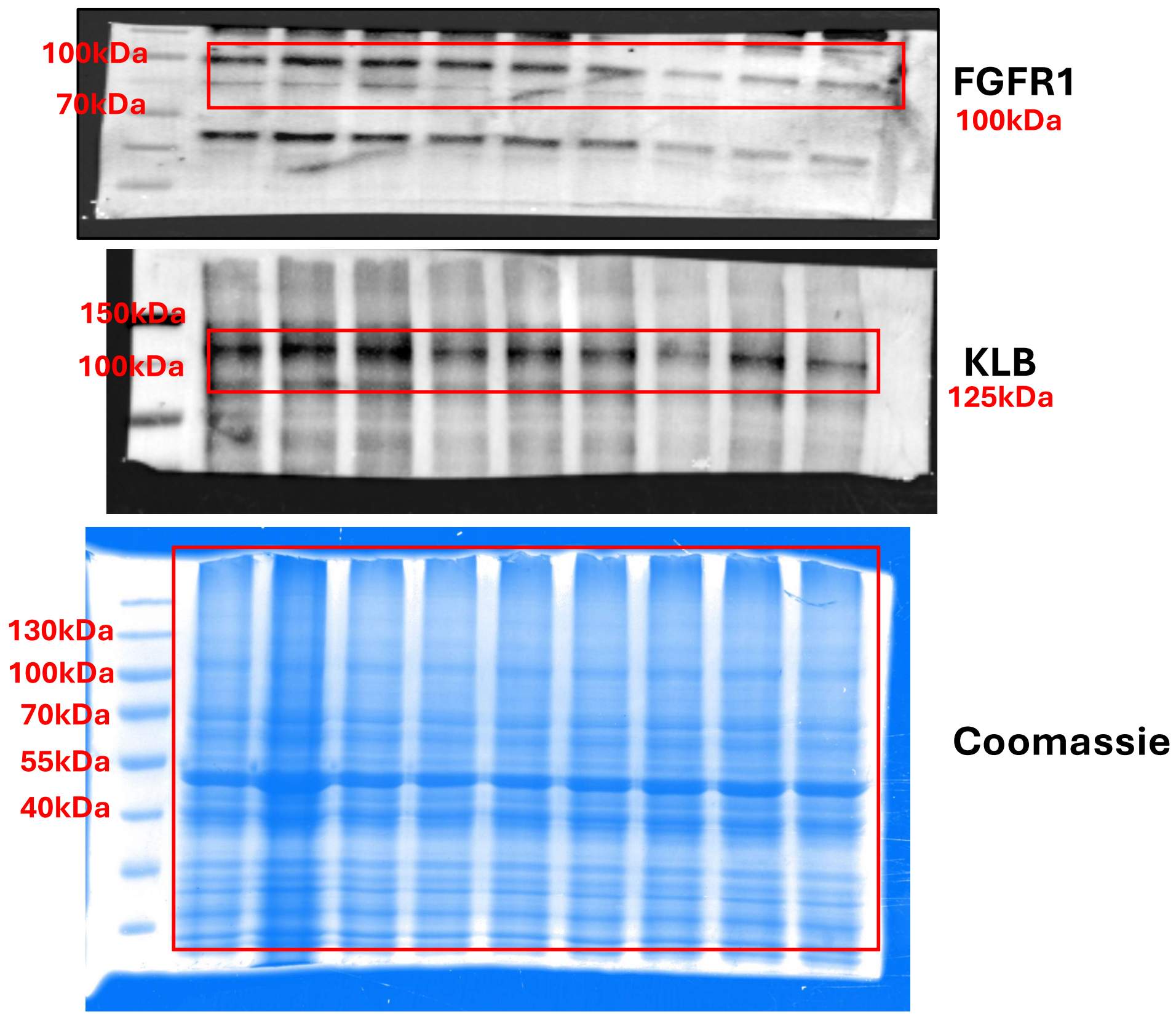

Figure S1C

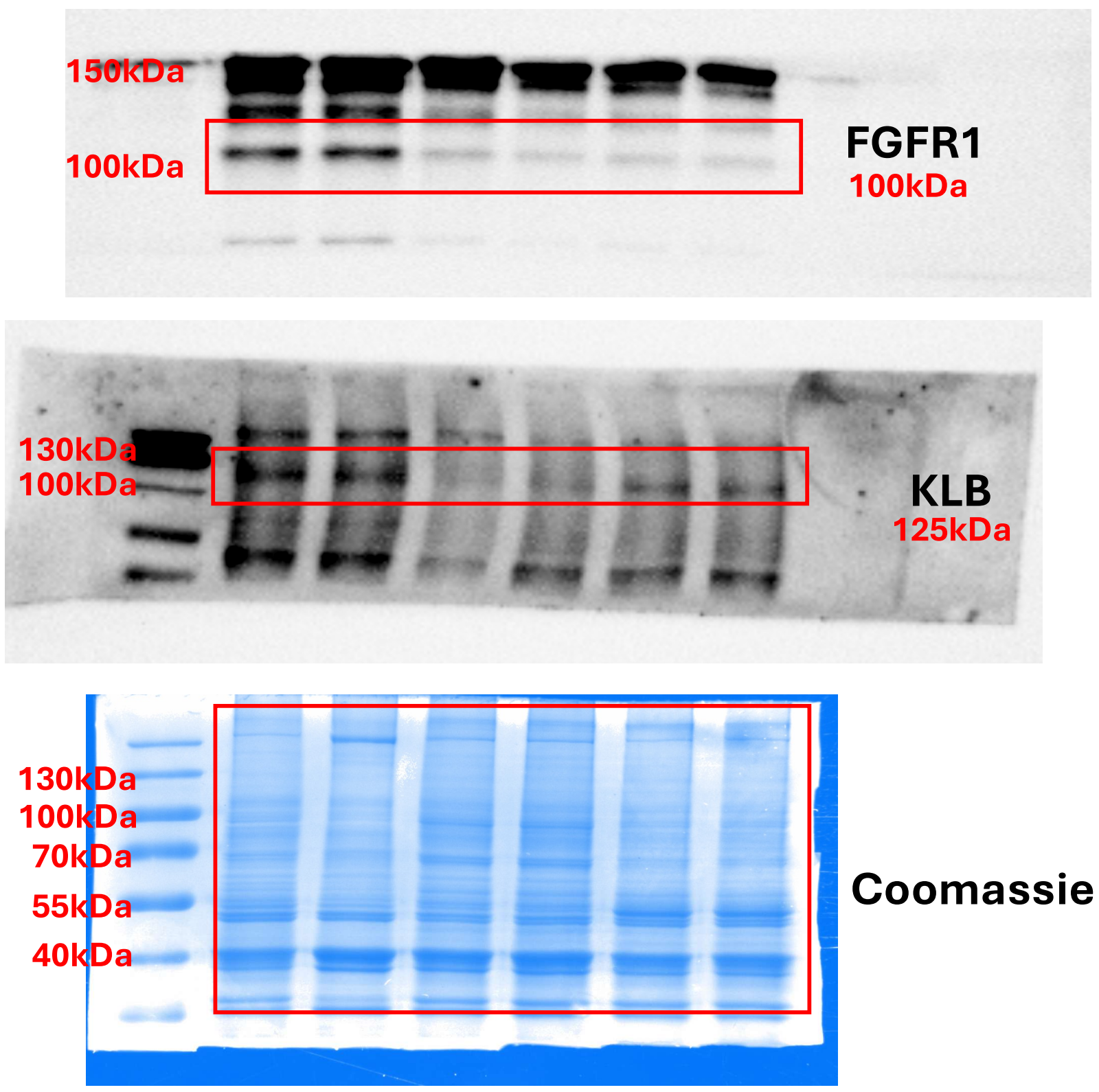

Figure S2 – Full unedited representative immunoblots

Figure S2B

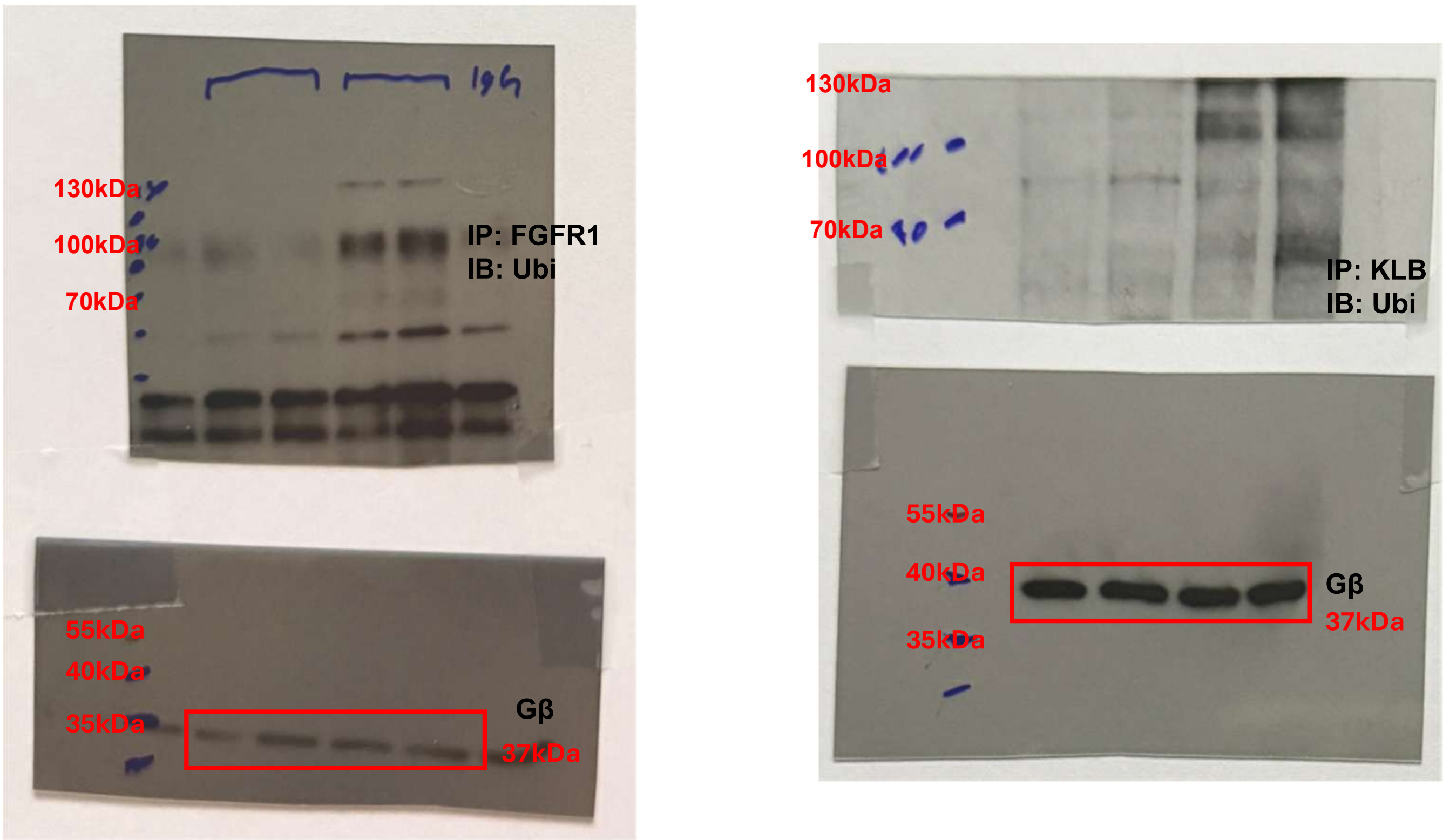

Figure S2C

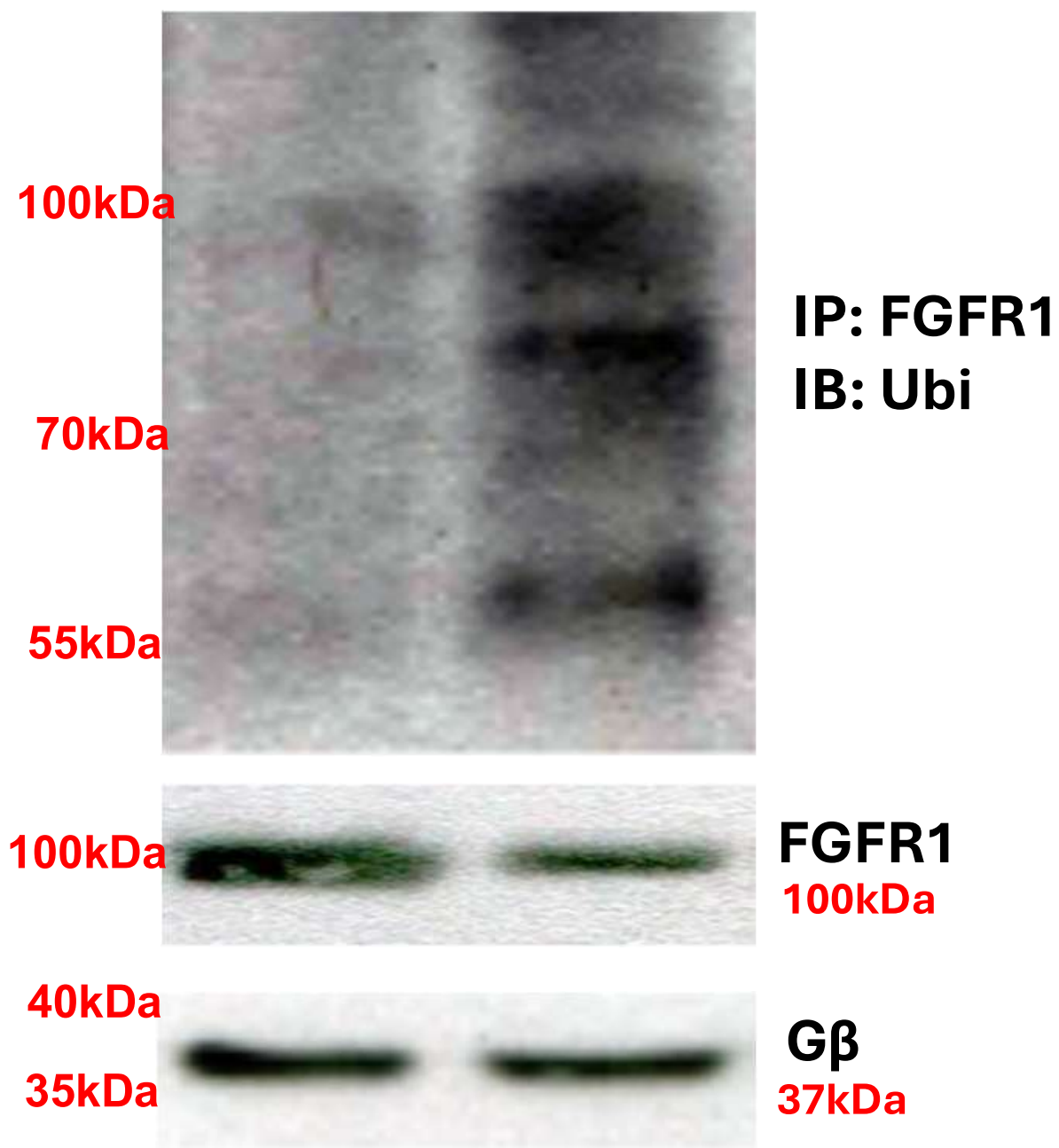

Figure S2D

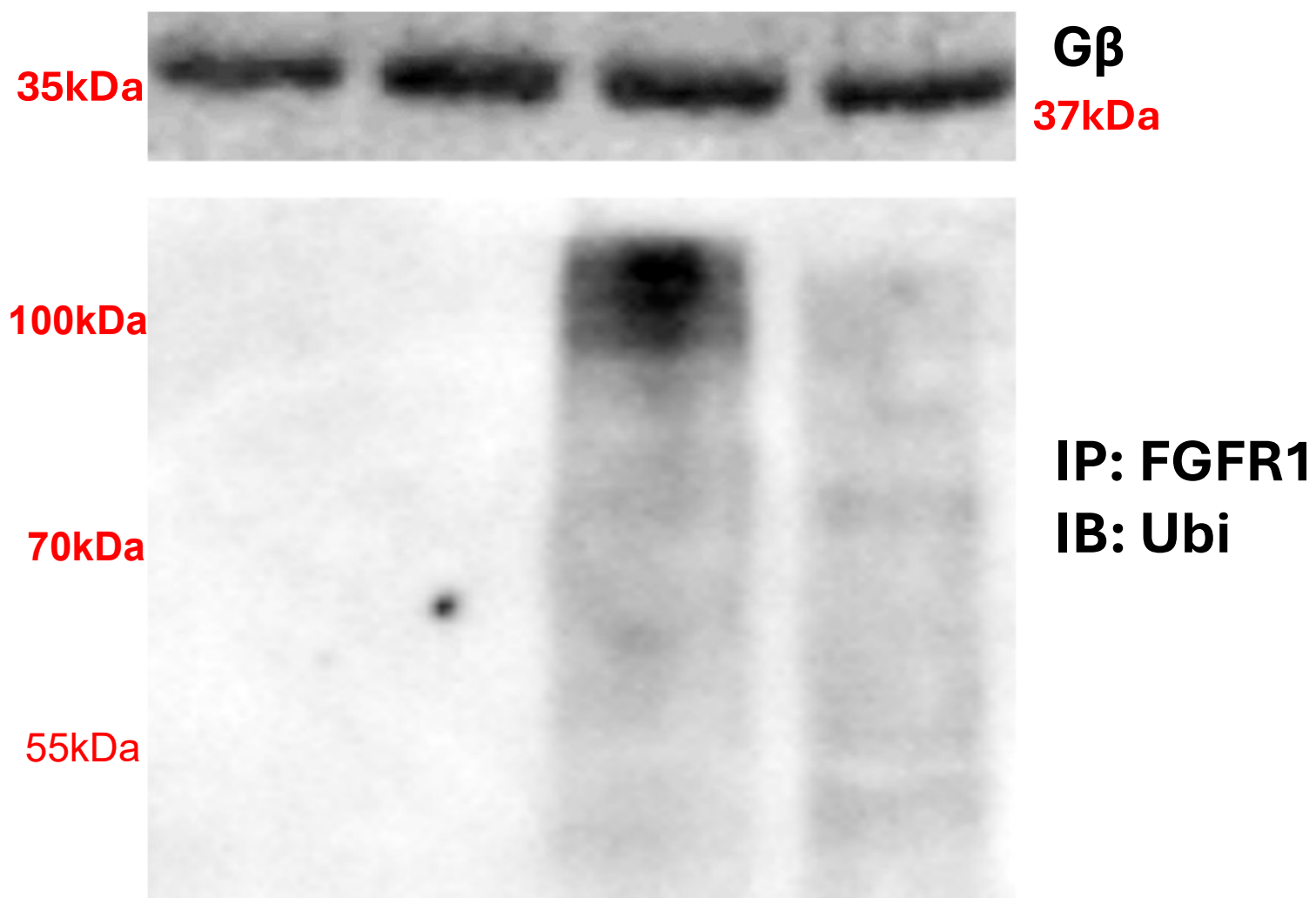

Figure S2 – Full unedited representative immunoblots continued

Figure S2E

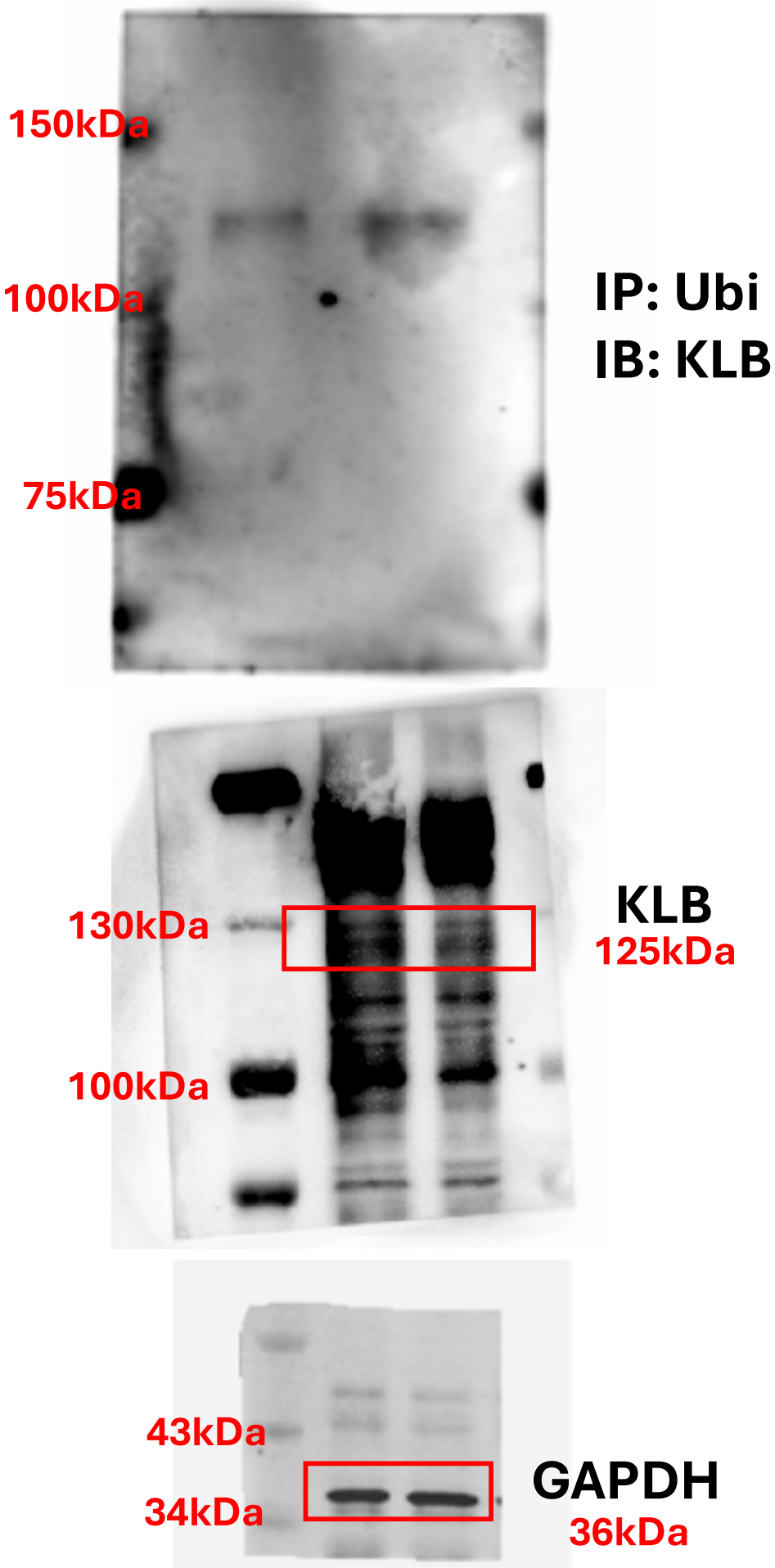

On the same gel with S2F

Figure S2F

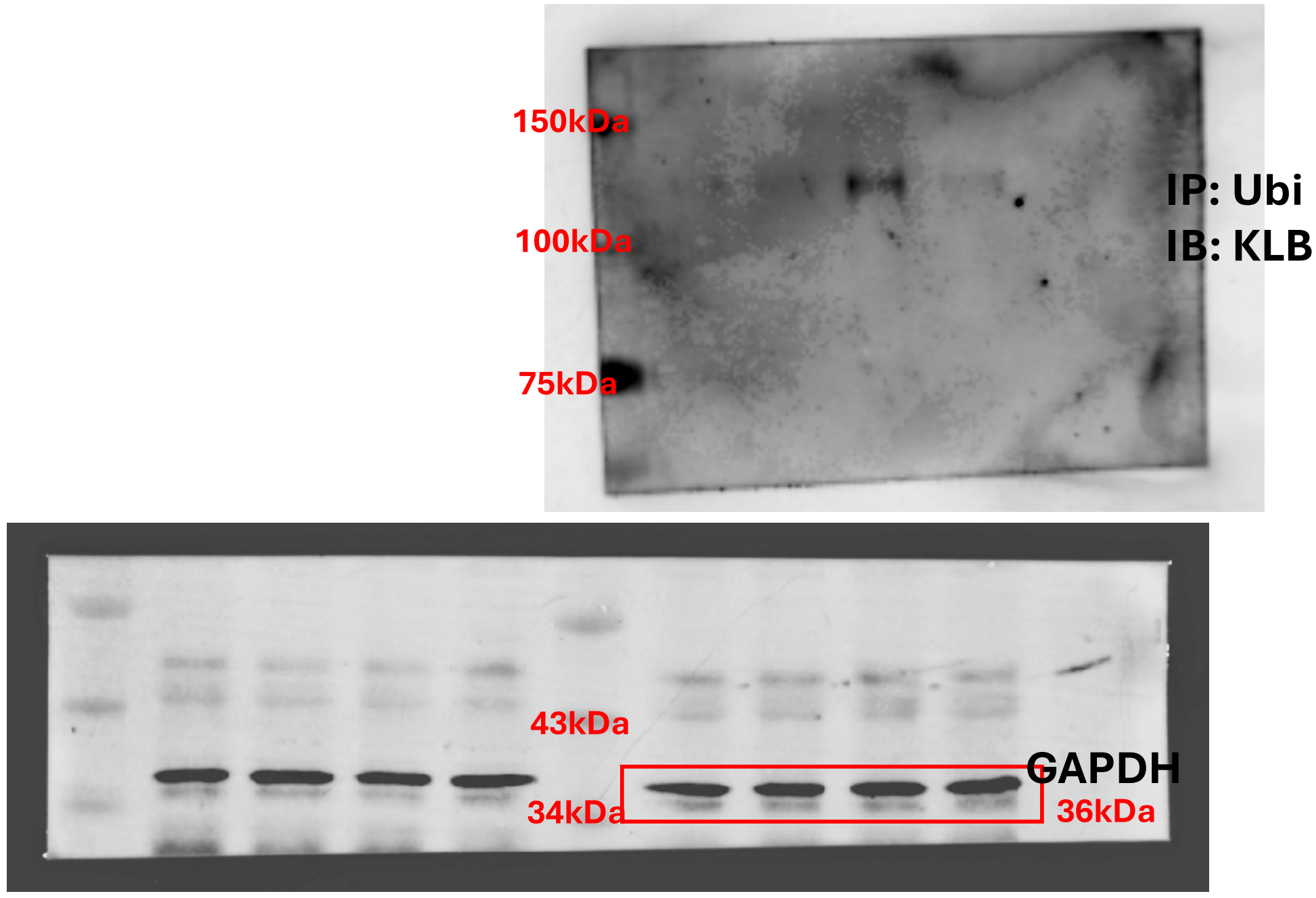

On the same gel with S2E

Figure S2G

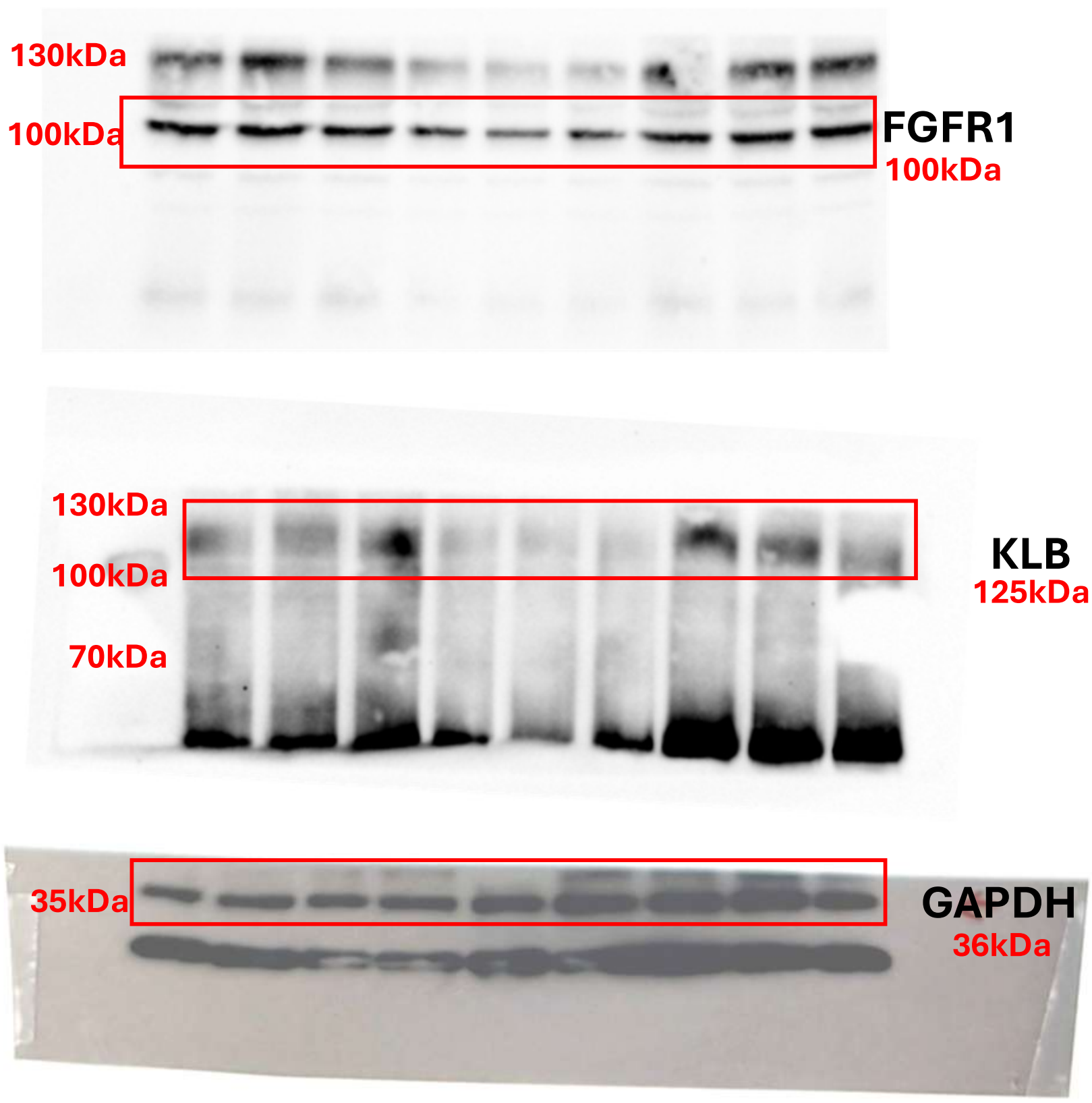

Figure S2 – Full unedited representative immunoblots continued

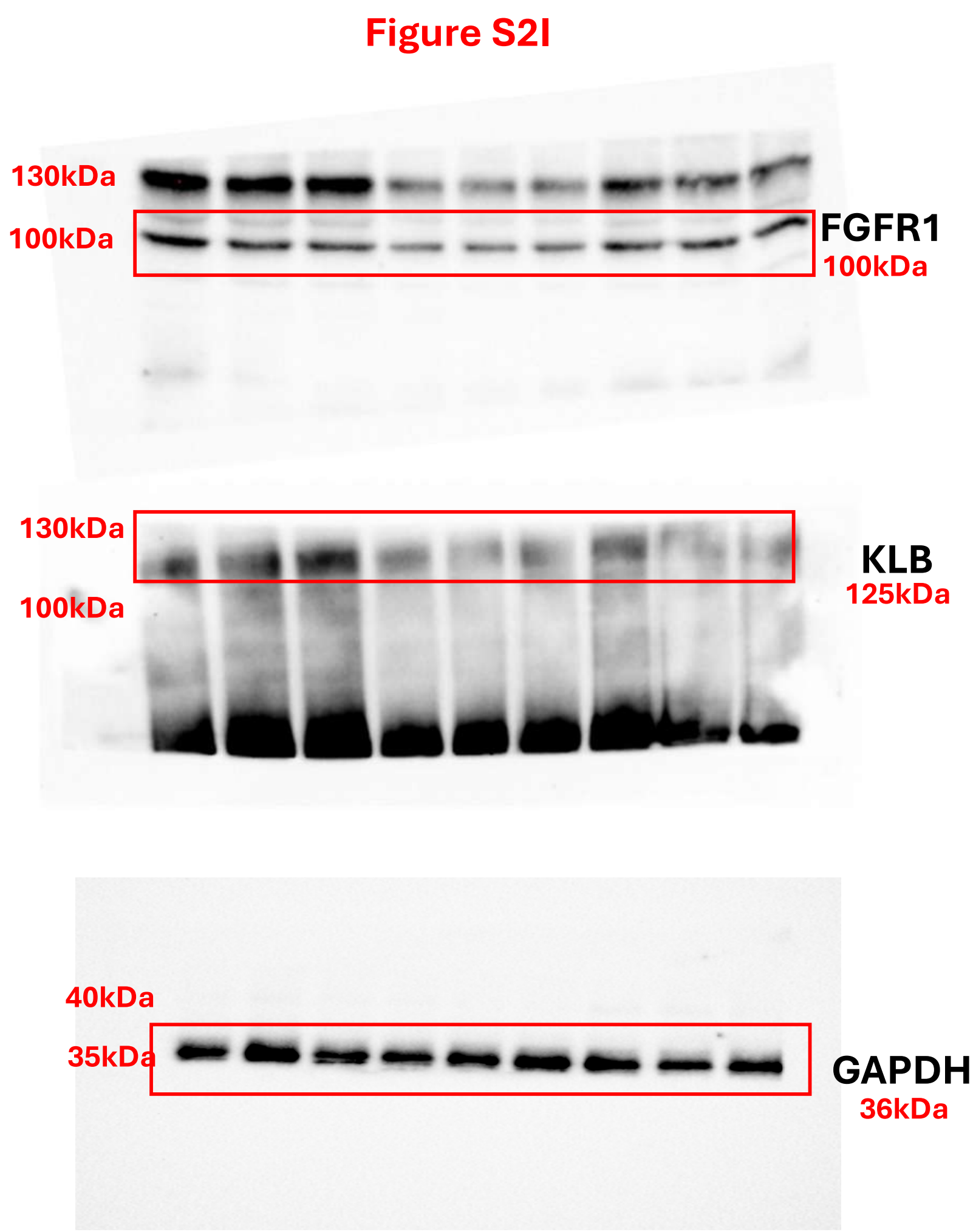

Figure S3 – Full unedited representative and quantification immunoblots

Figure S3A

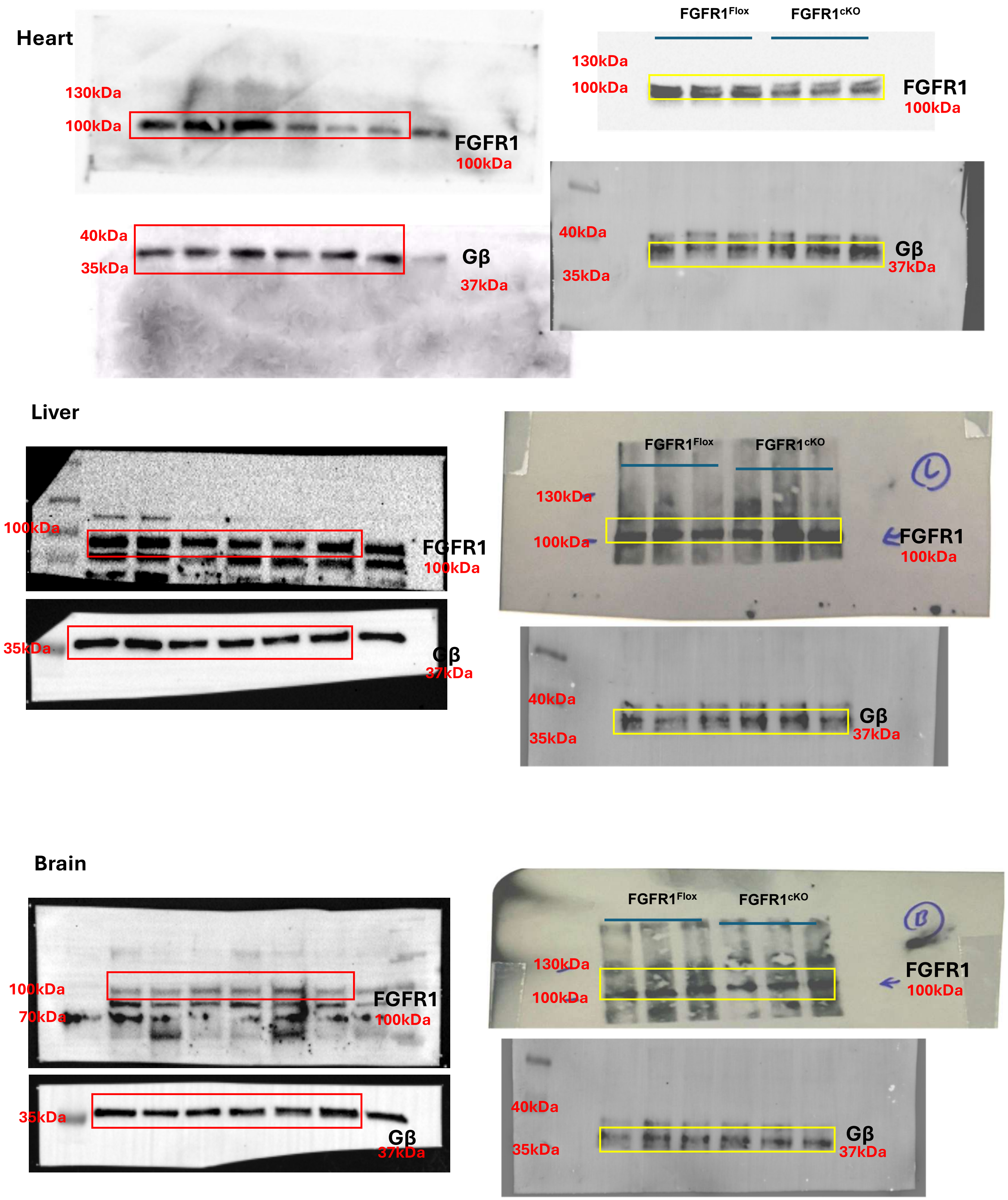

Figure S3 – Full unedited representative and quantification immunoblots continued

Figure S3B

Heart

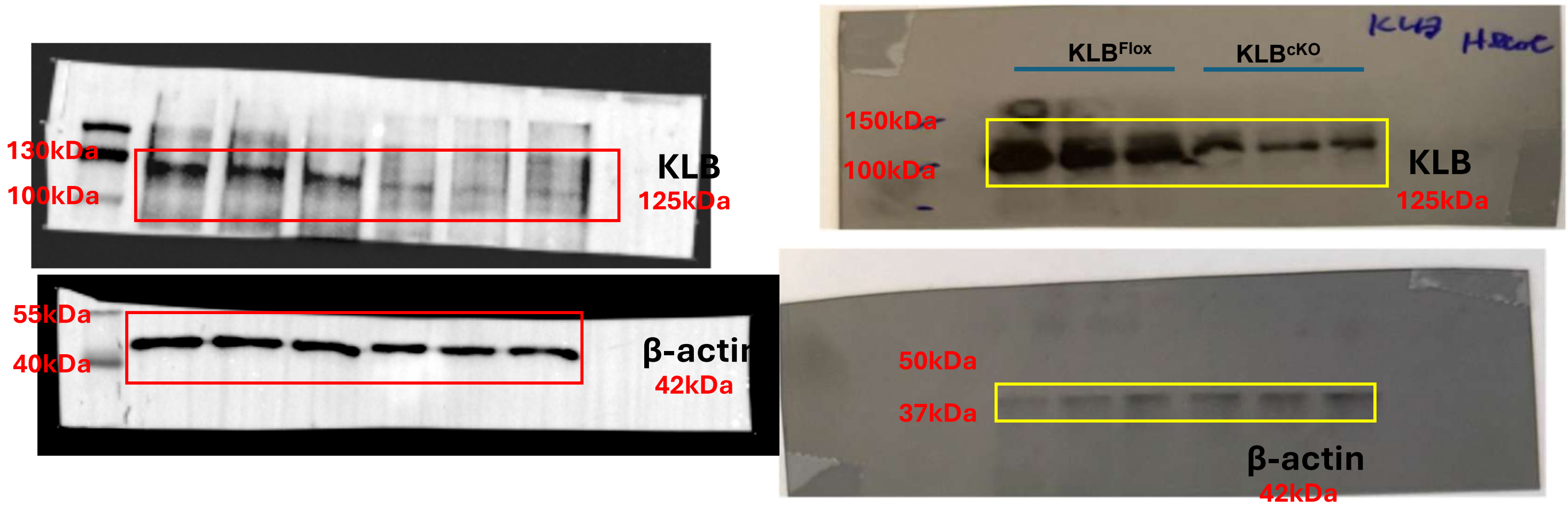

Liver

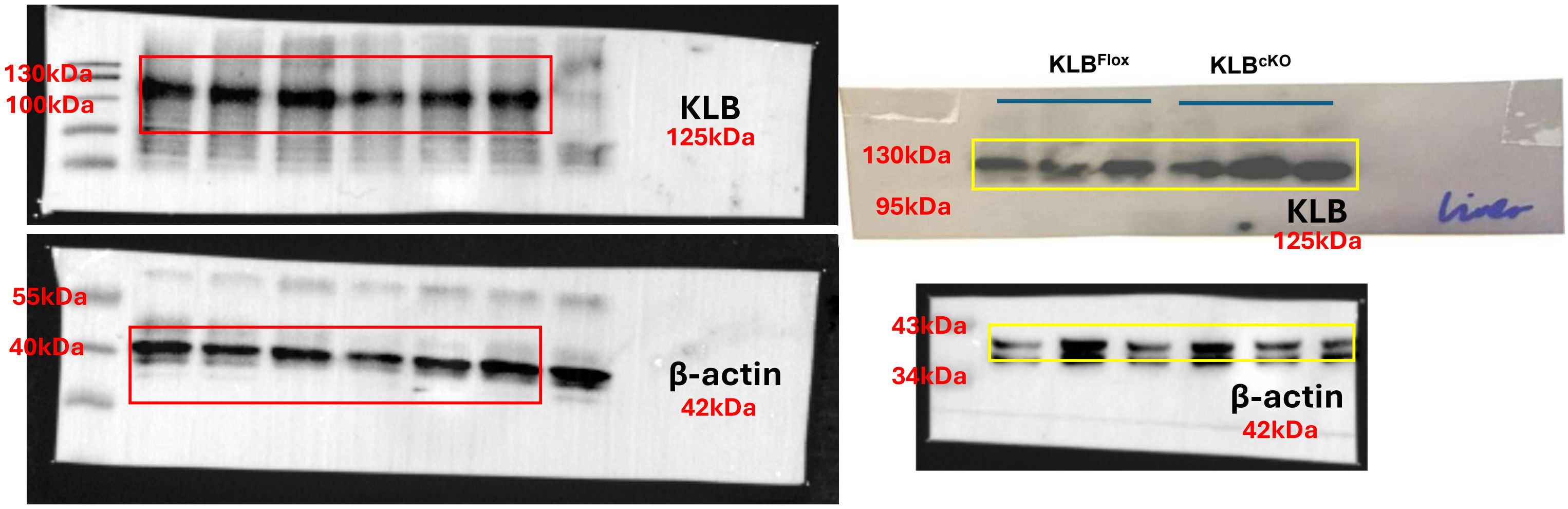

Brain

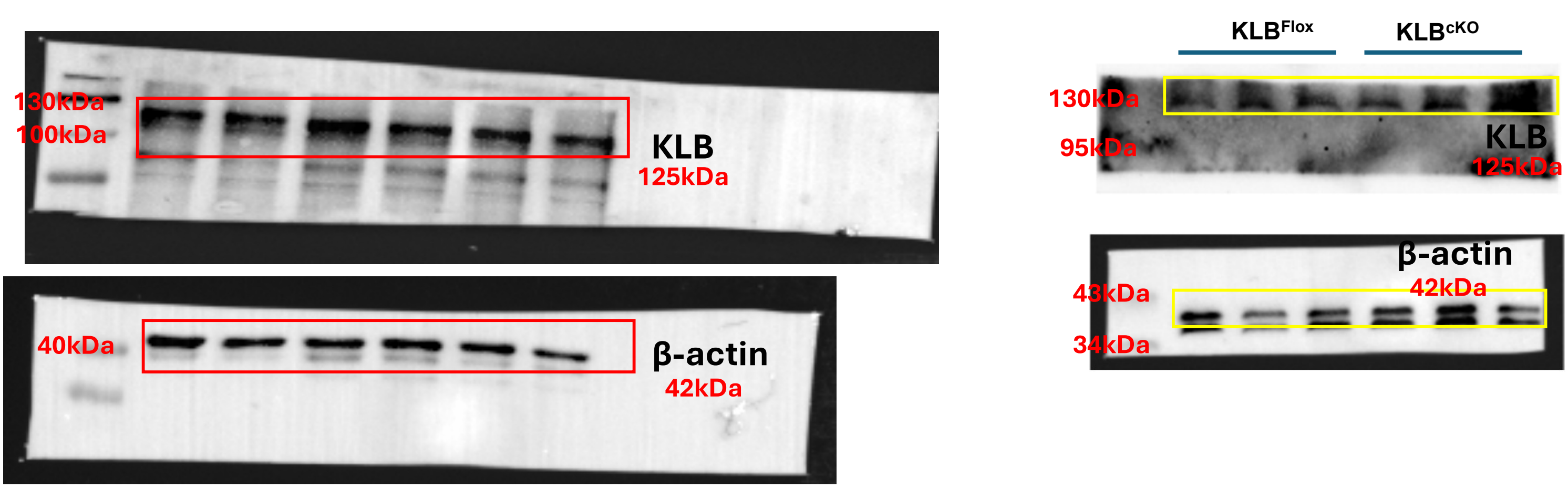

Figure S8 – Full unedited representative and quantification immunoblots

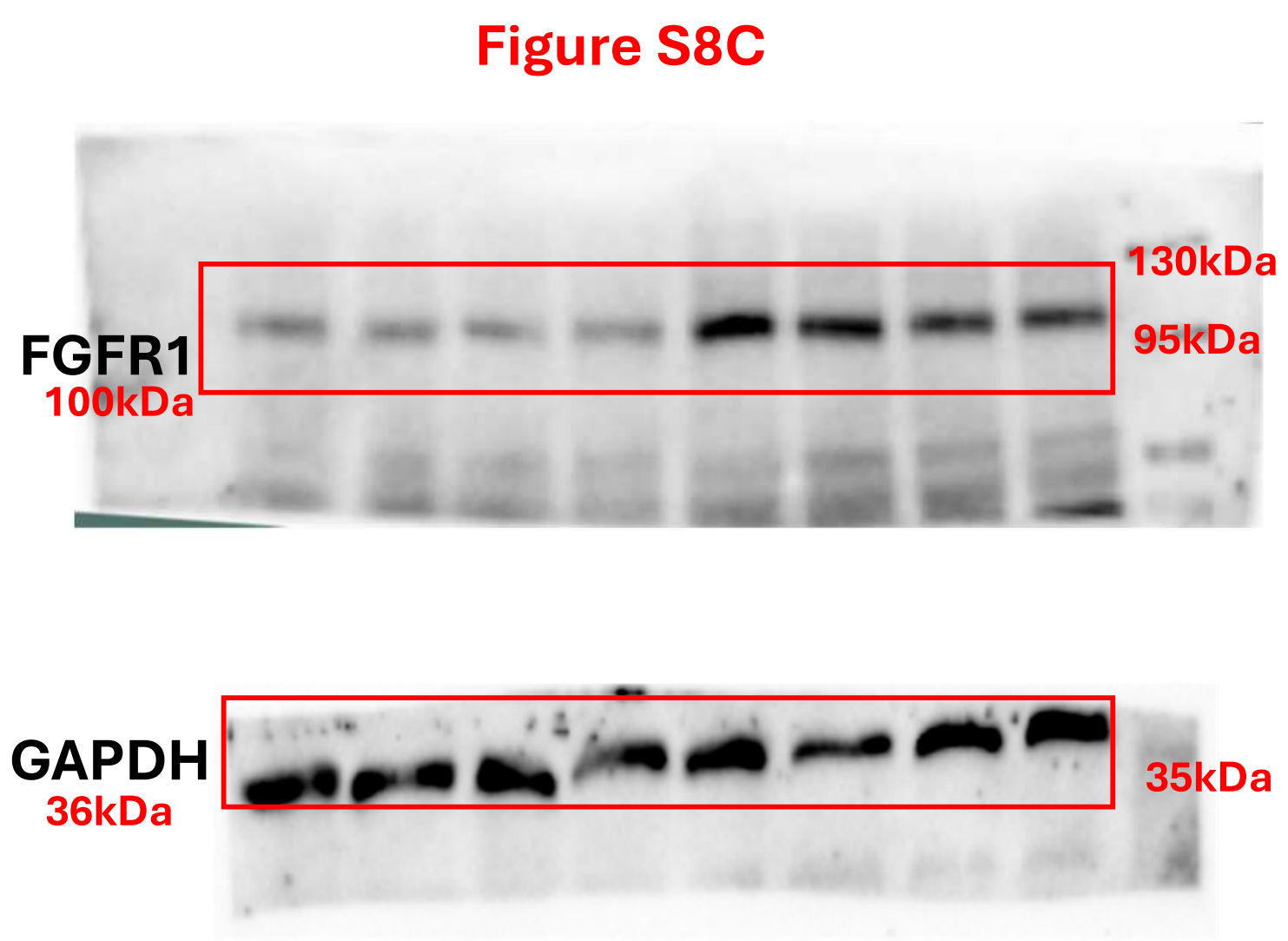

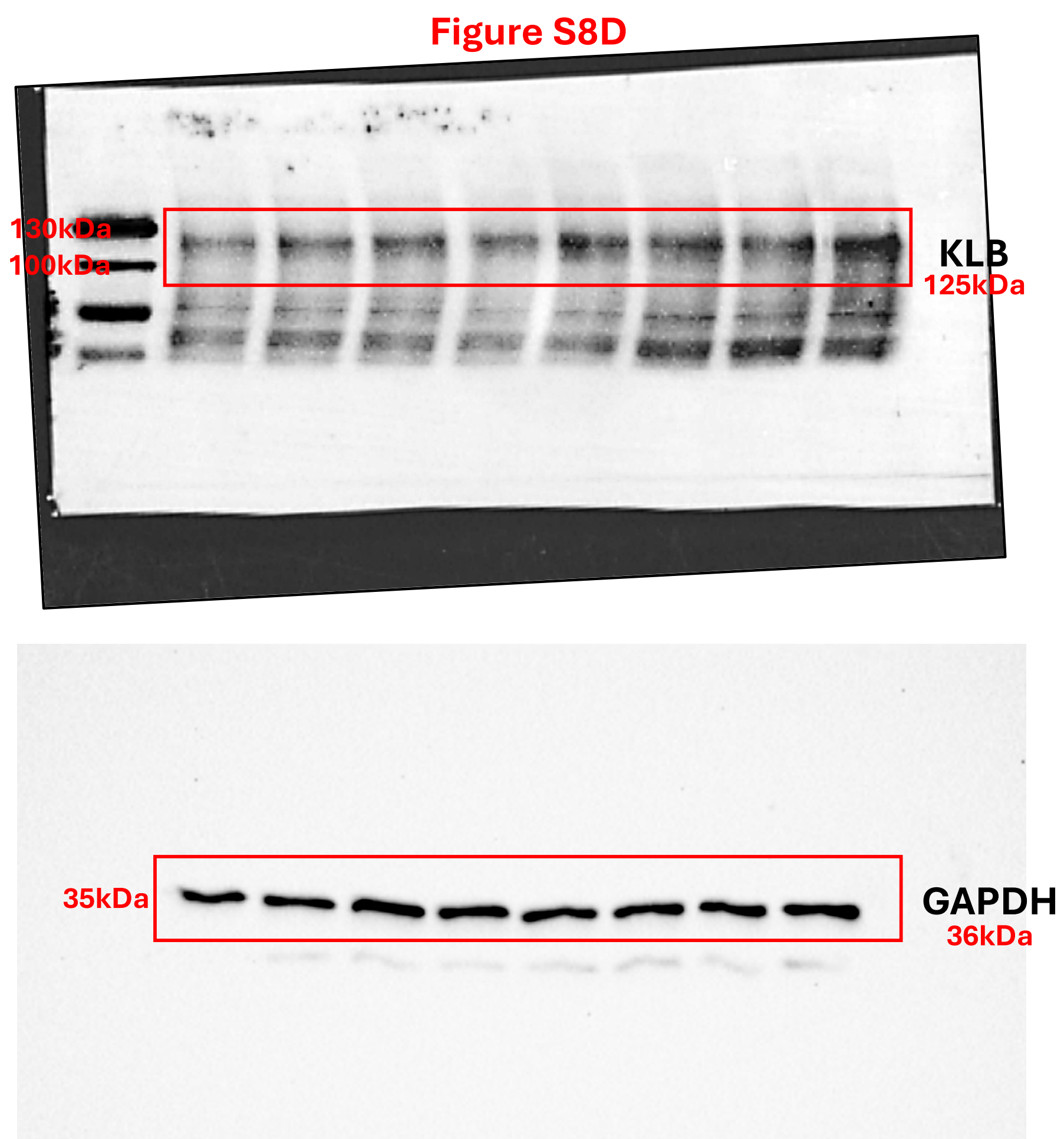

Figure S11 – Full unedited representative immunoblots

Figure S11A

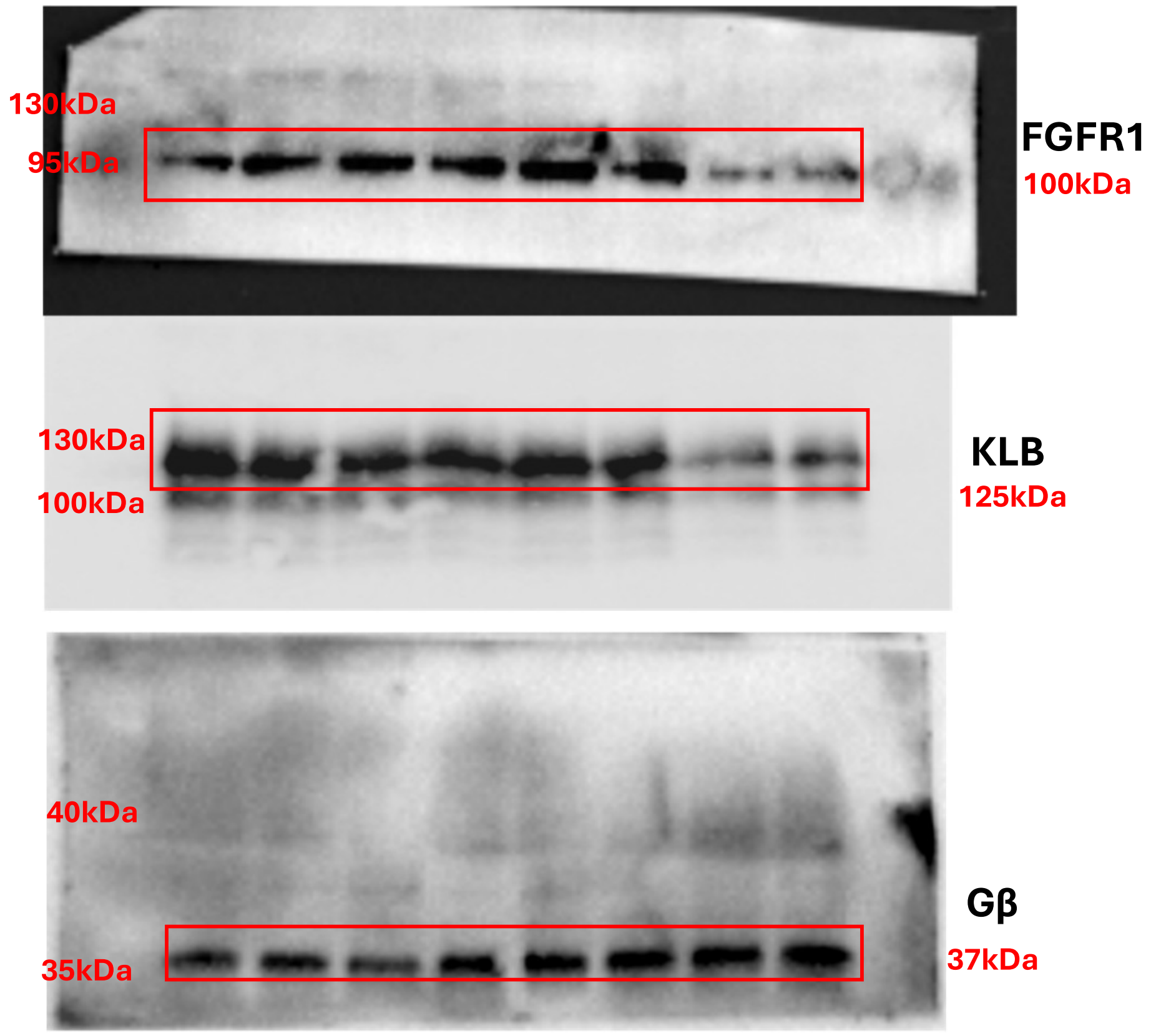

Figure S11B

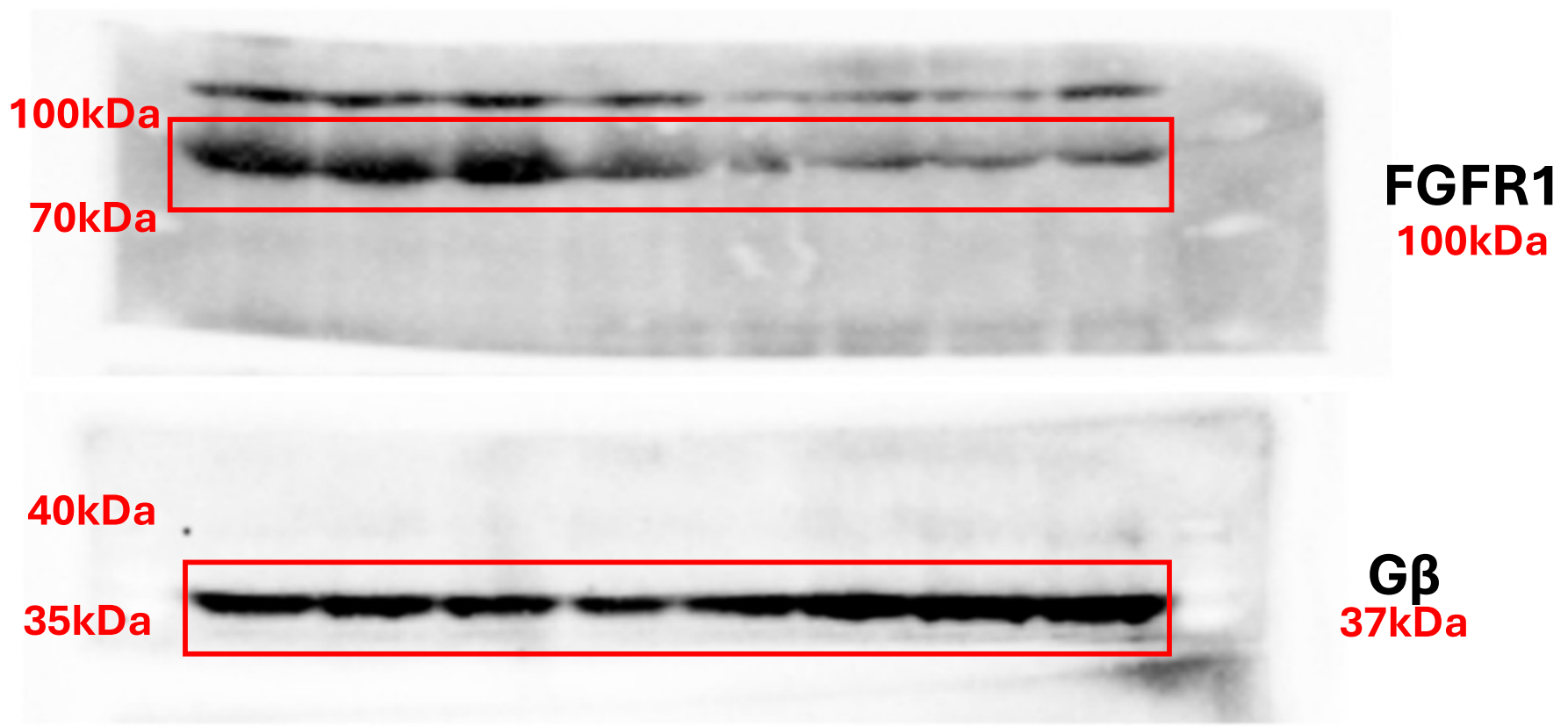

Figure S11C

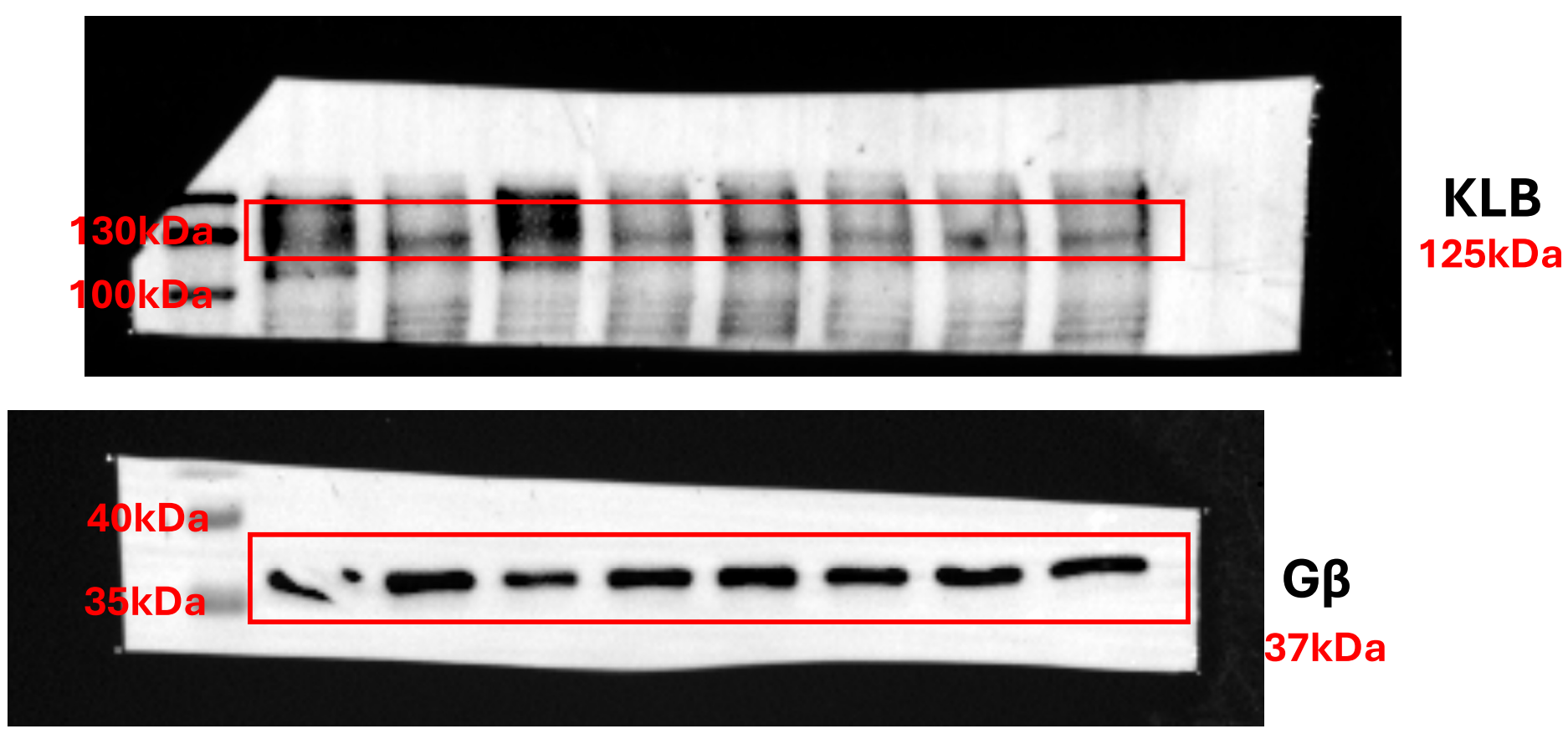

Figure S11 – Full unedited representative immunoblots continued

Figure S11E

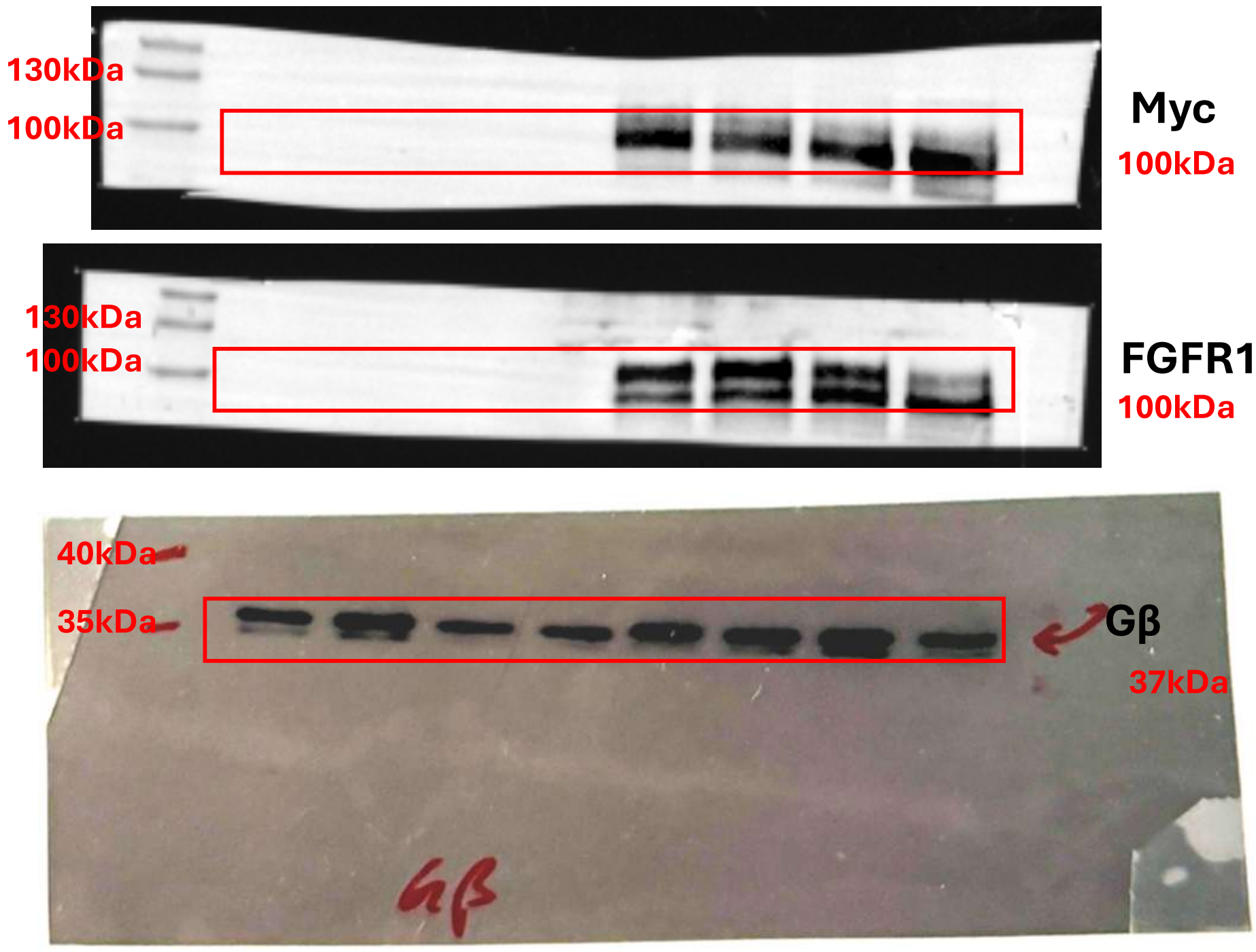

Figure S11F

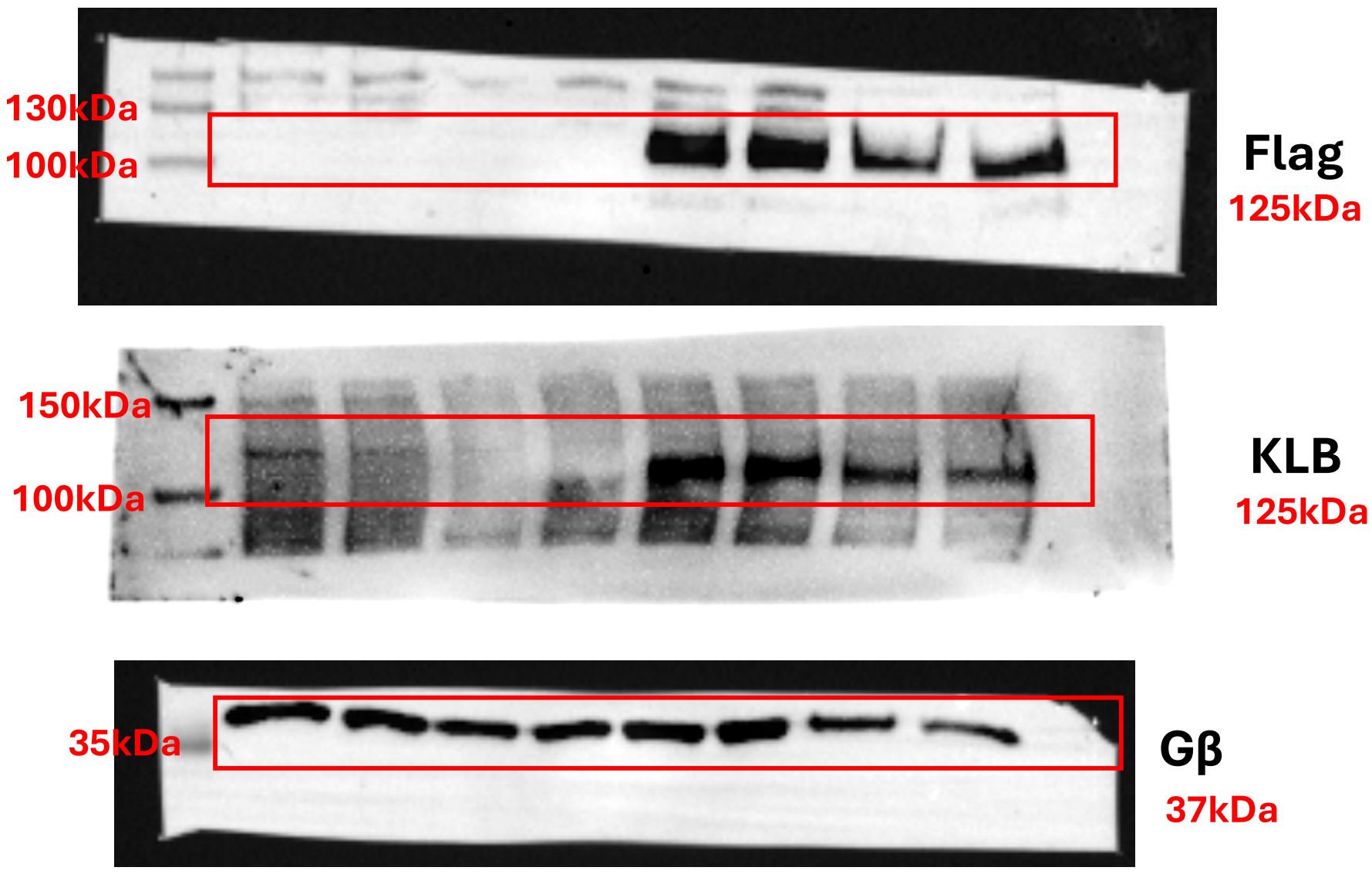

Figure S12 – Full unedited representative and quantification immunoblots

Figure S12A

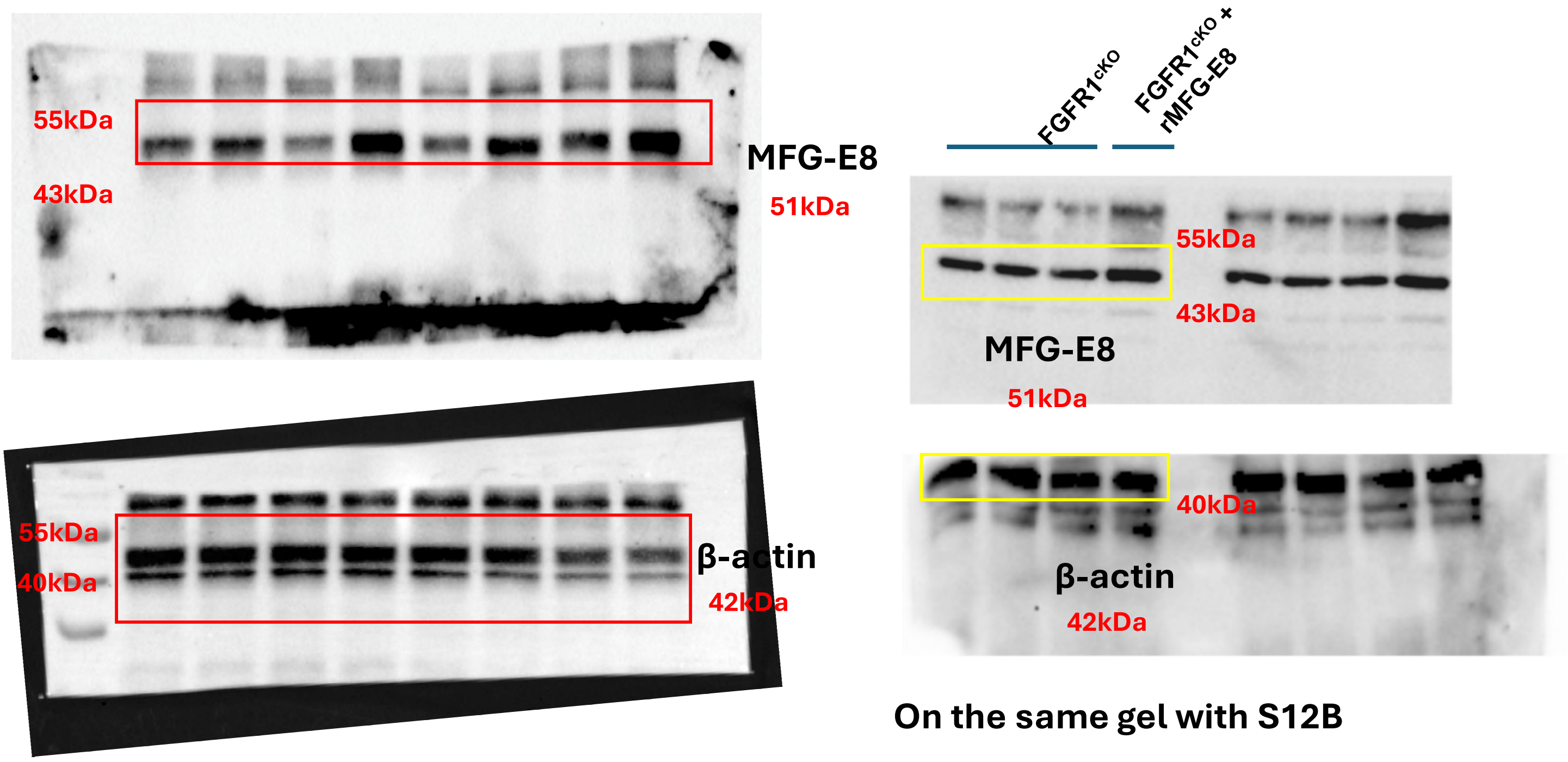

On the same gel with S12B

Figure S12B

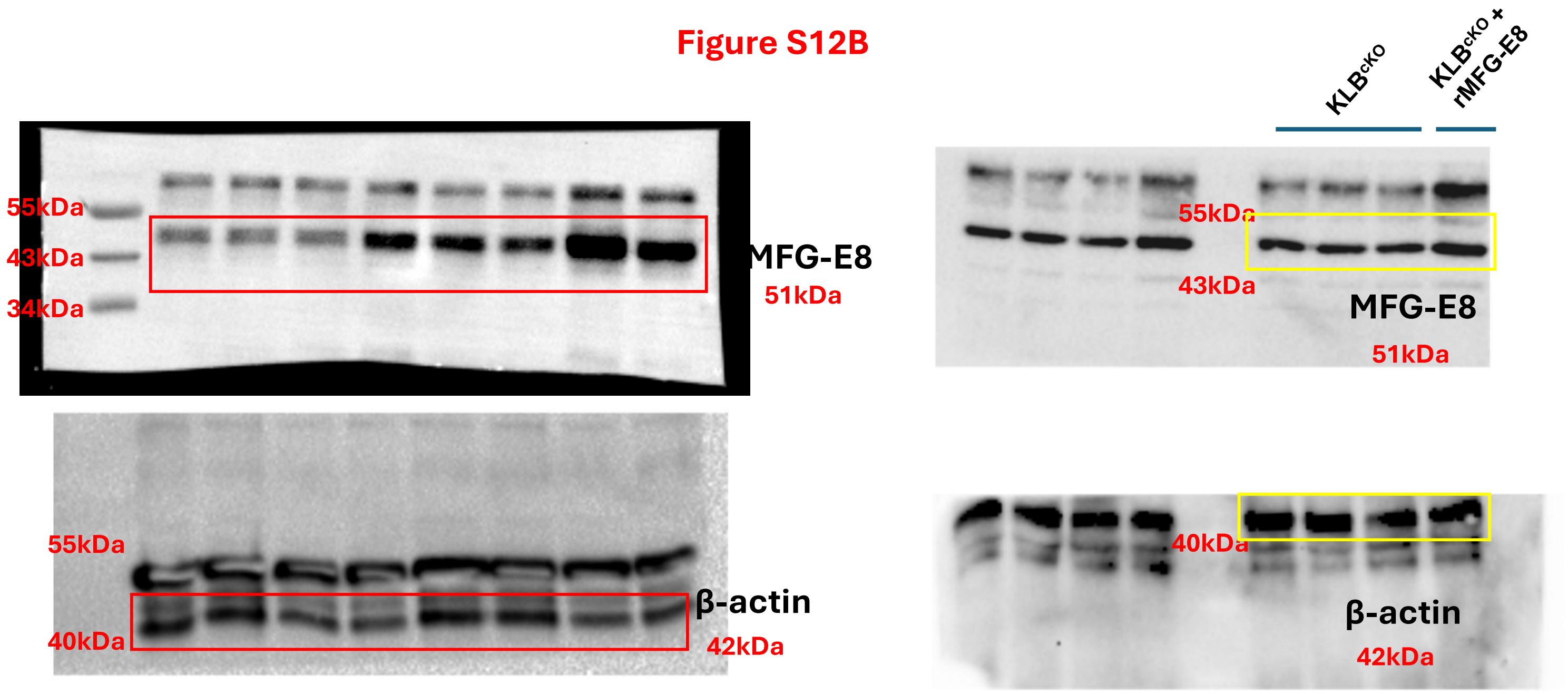

On the same gel with S12A

Figure S15 – Full unedited representative and quantification immunoblots

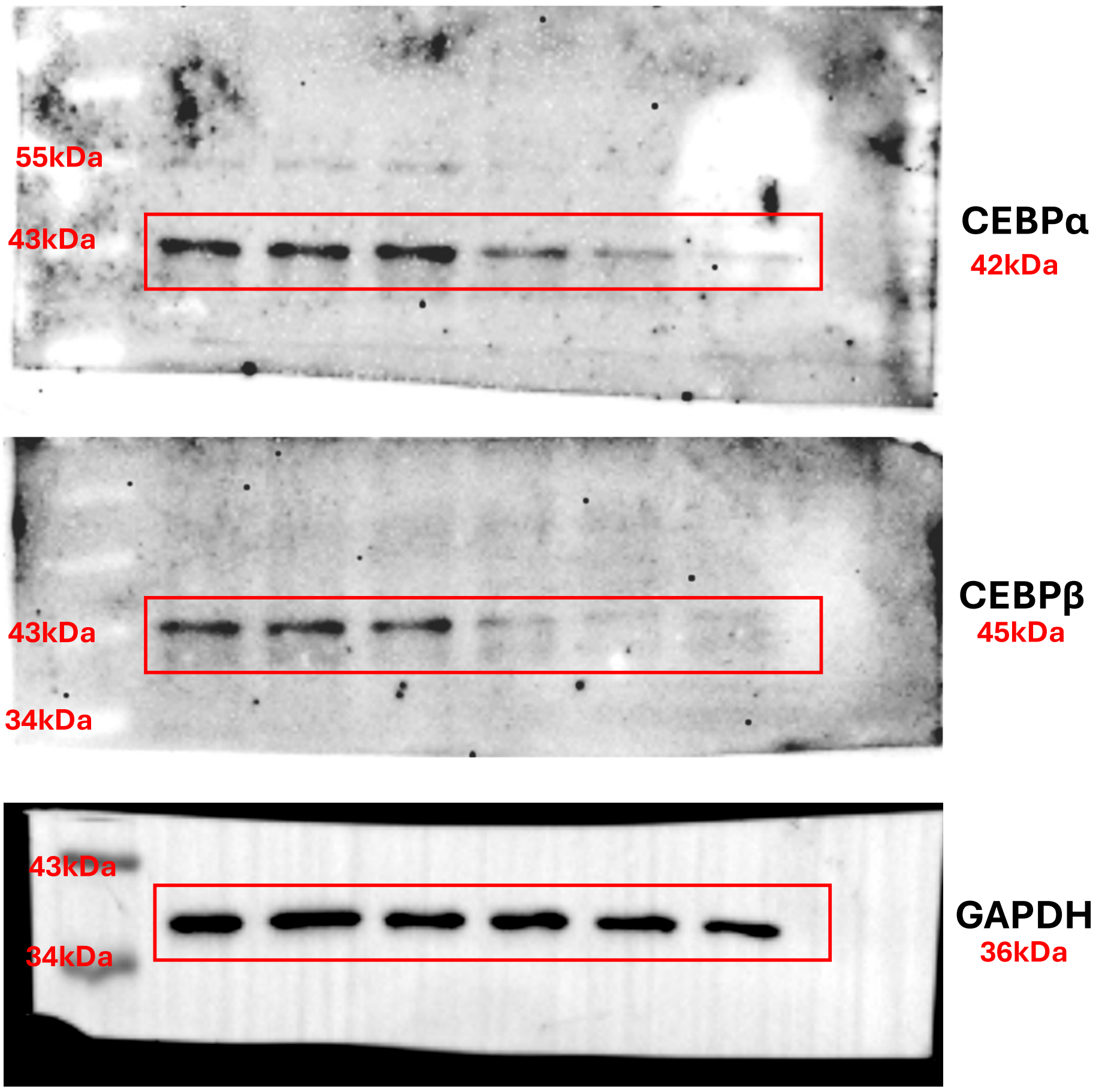

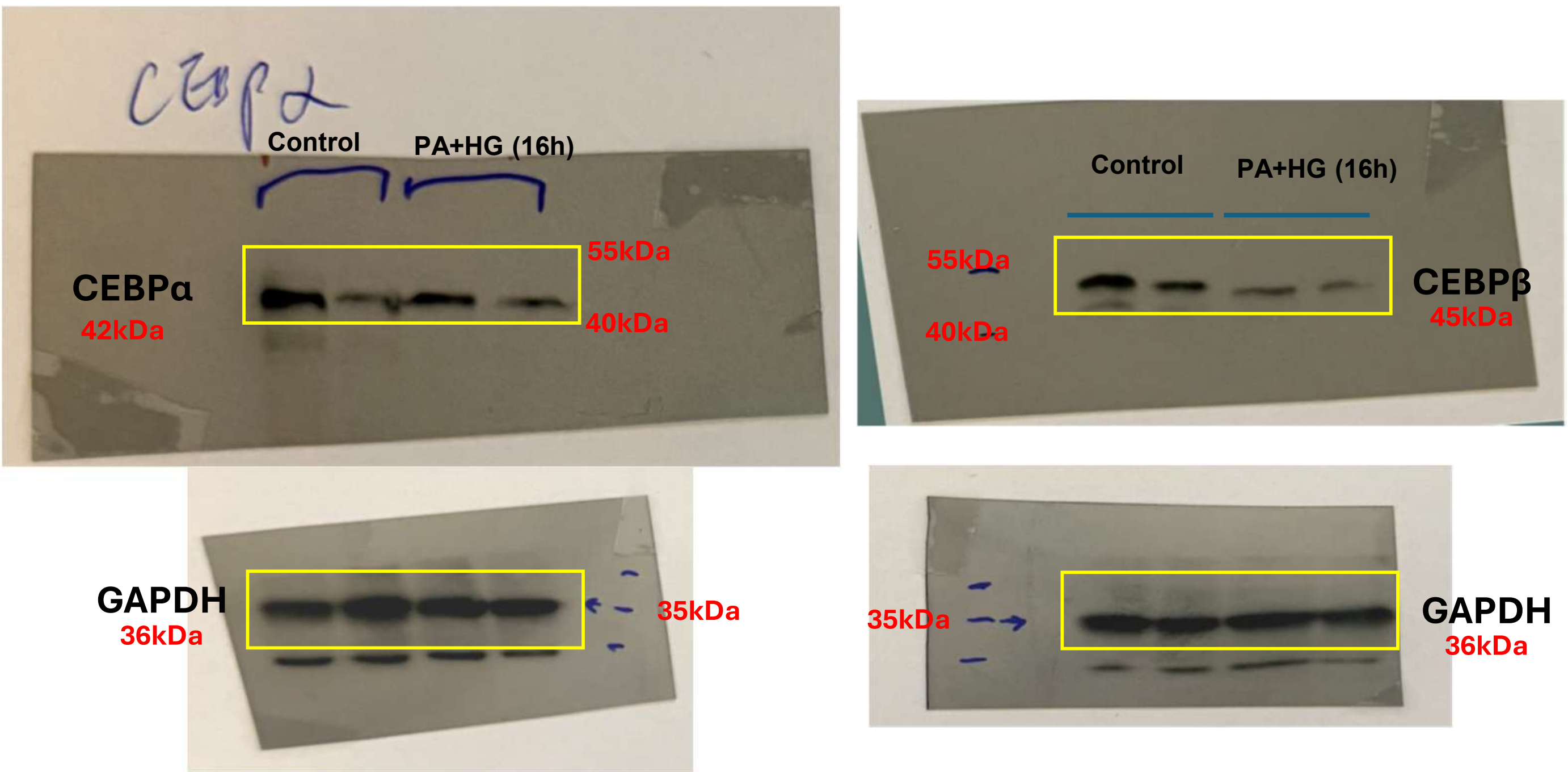

Figure S16 – Full unedited representative and quantification immunoblots

Figure S16A

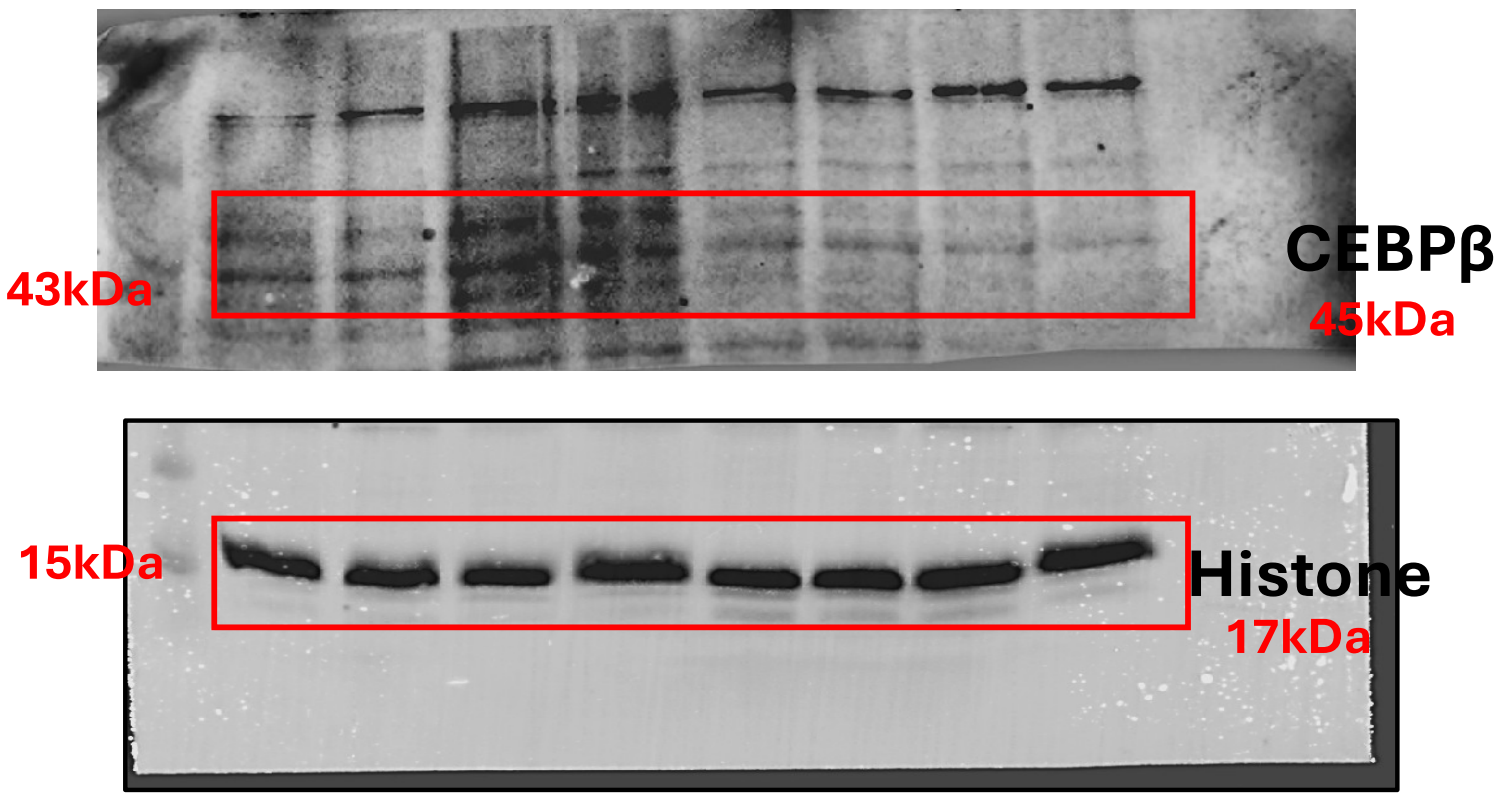

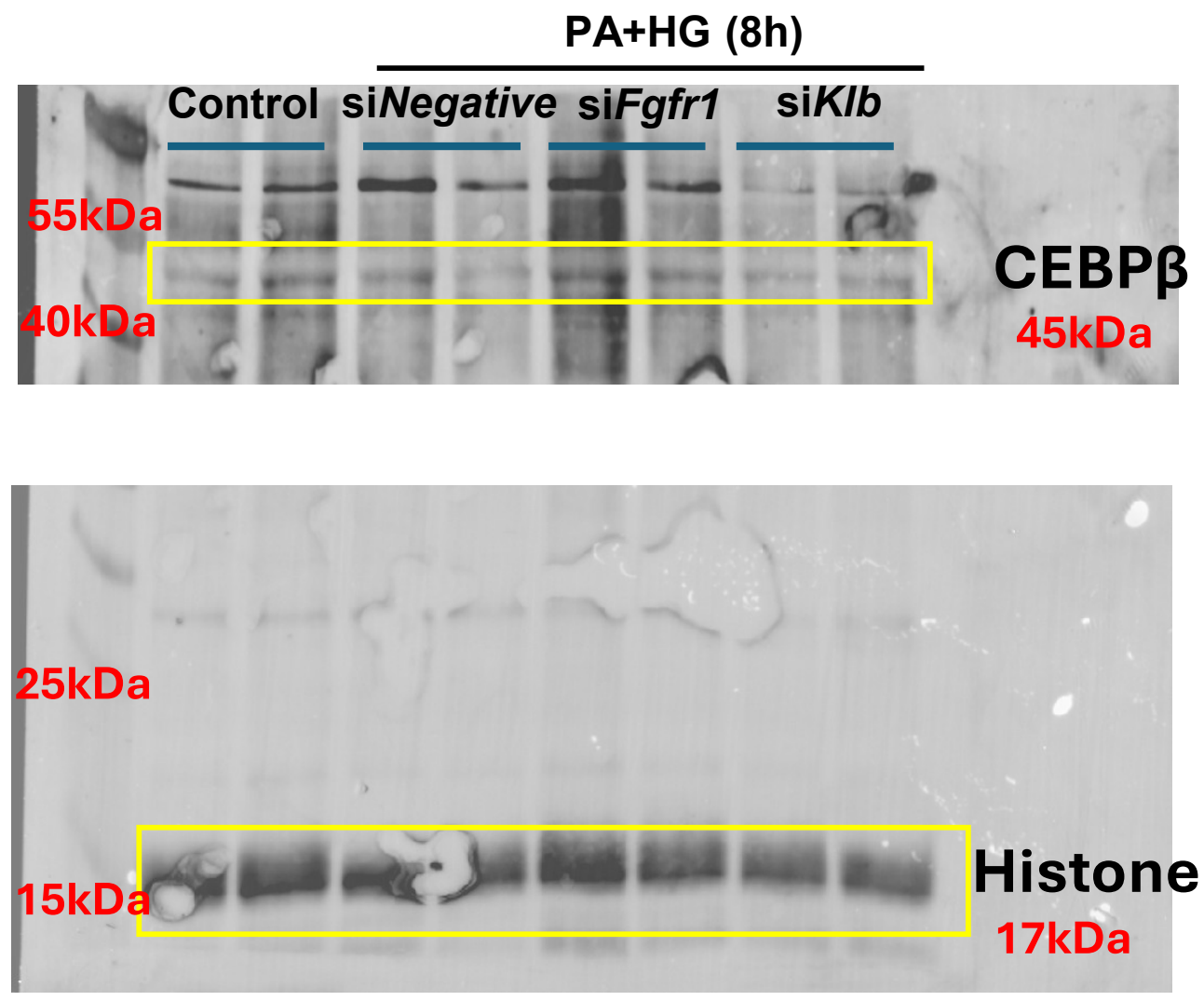

Figure S16C

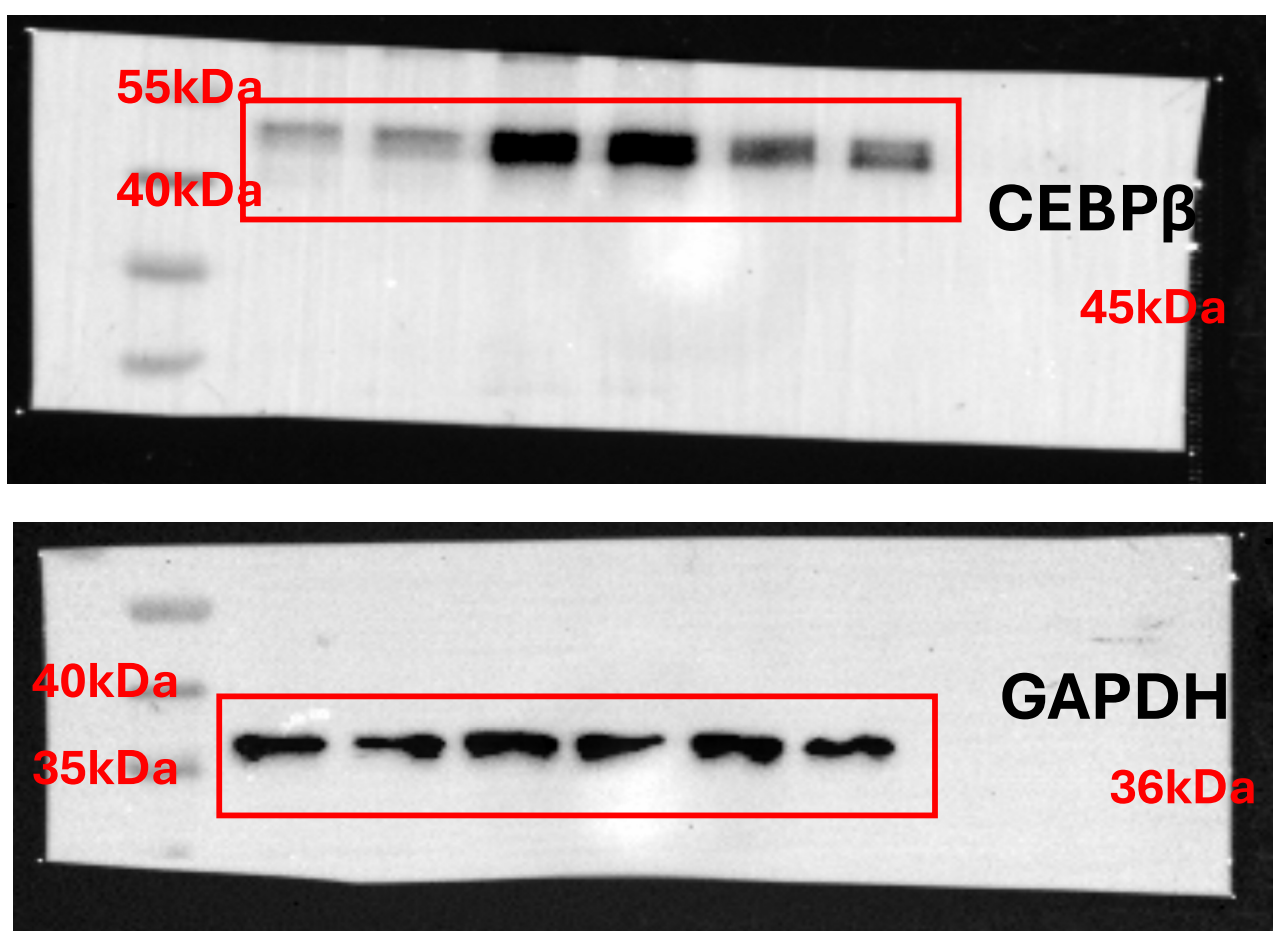

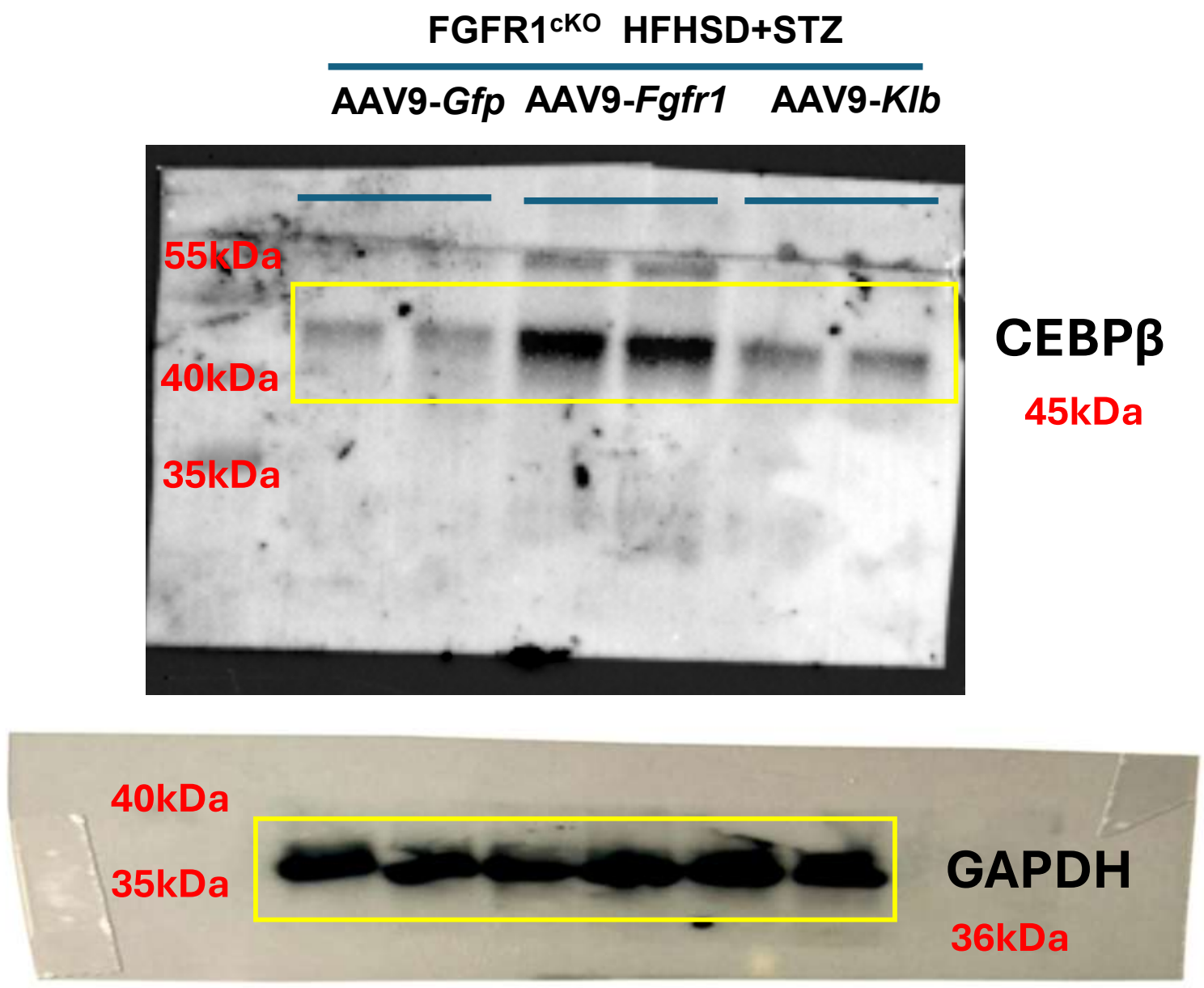

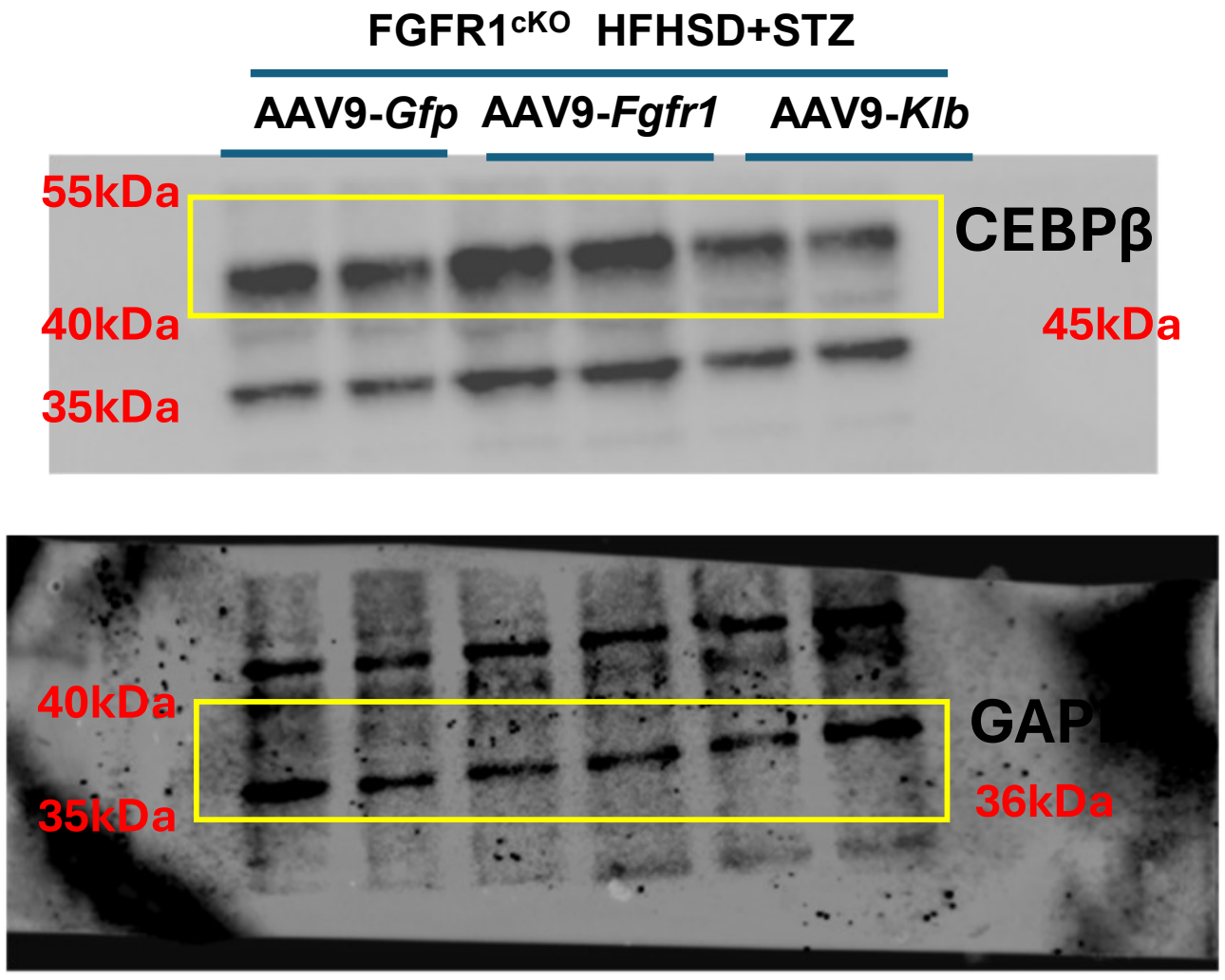

Figure S16 – Full unedited representative and quantification immunoblots continued

Figure S16D

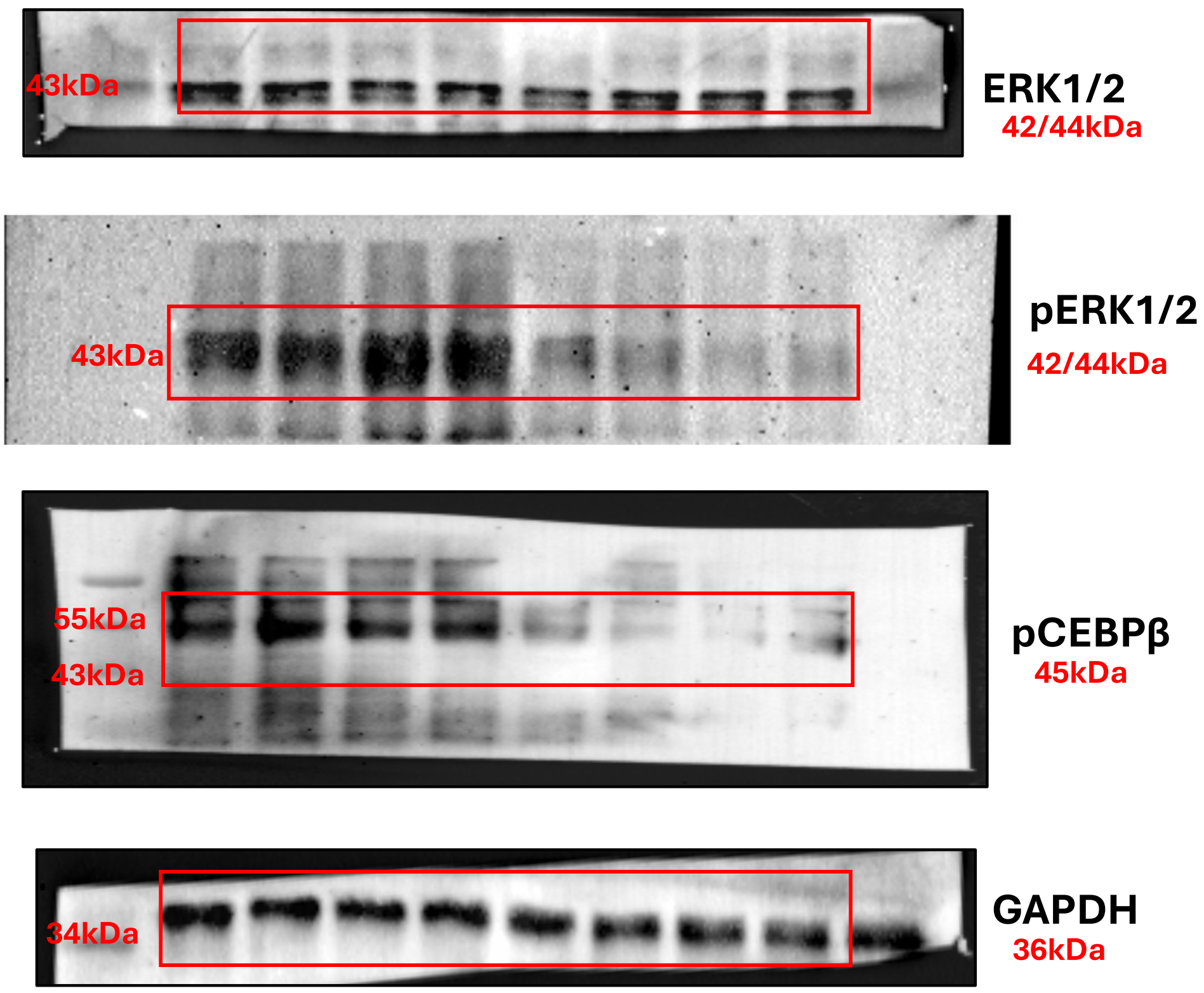

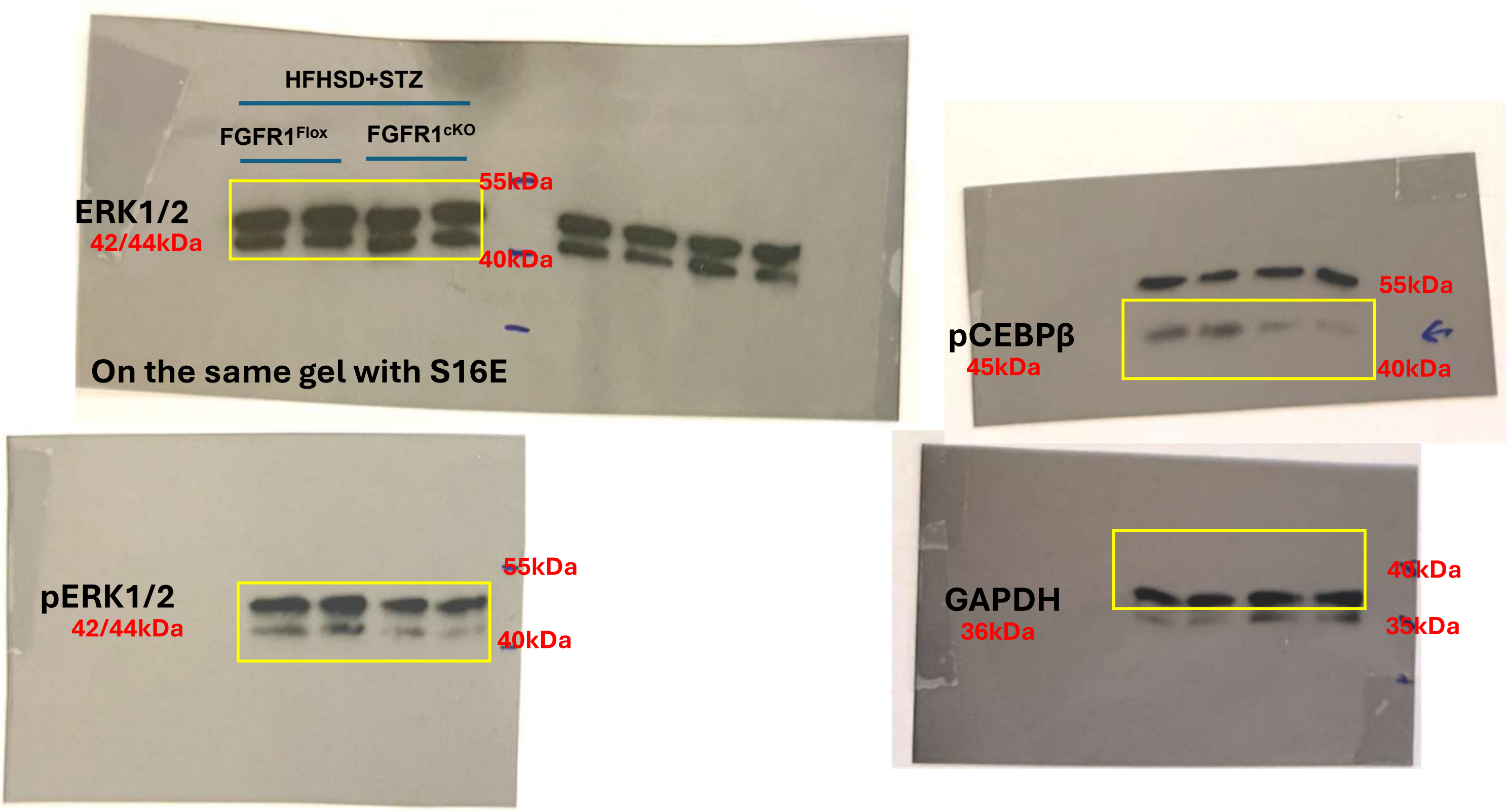

Figure S16 – Full unedited representative and quantification immunoblots continued

Figure S16E

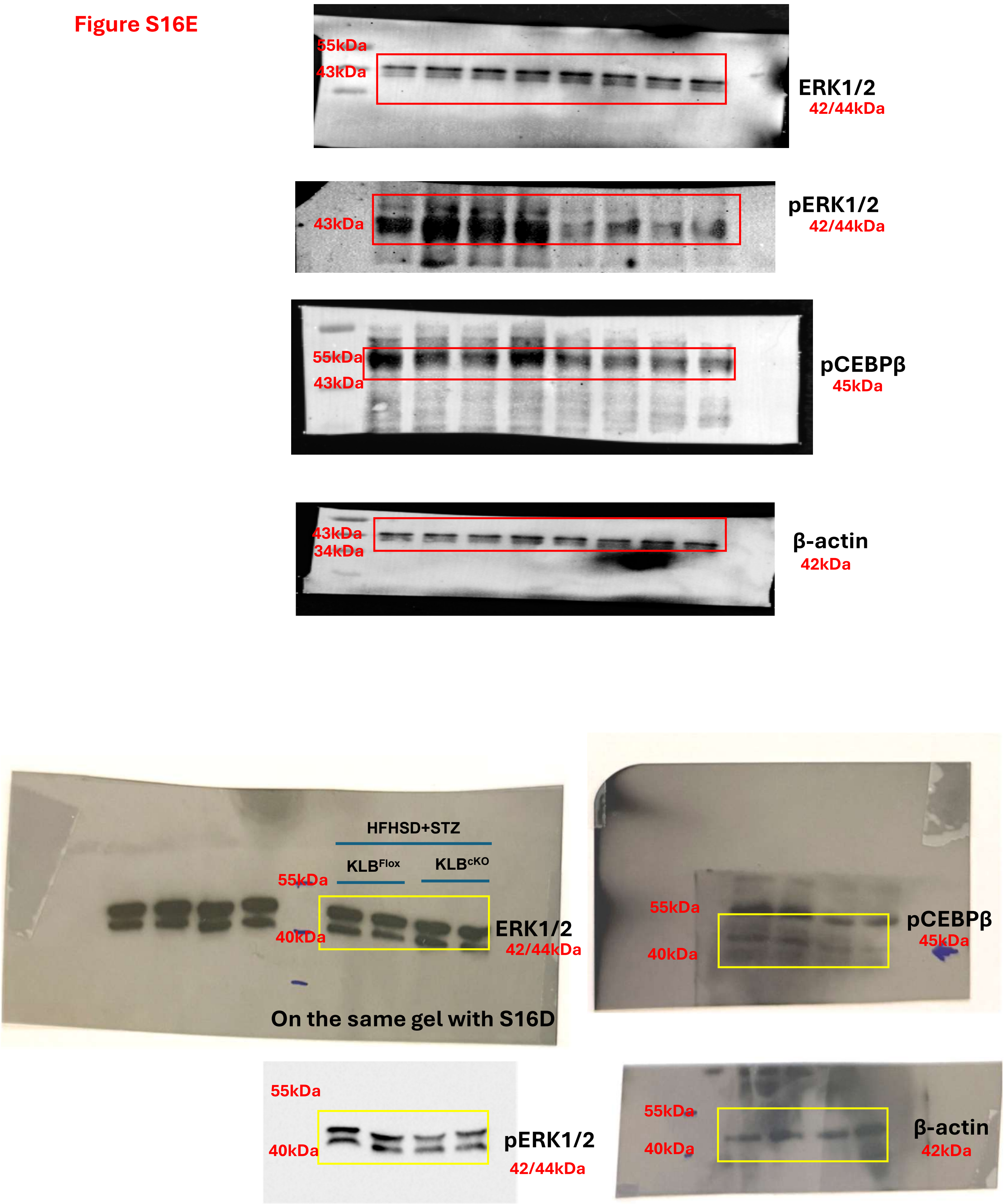

Figure S16 – Full unedited representative and quantification immunoblots continued

Figure S16F

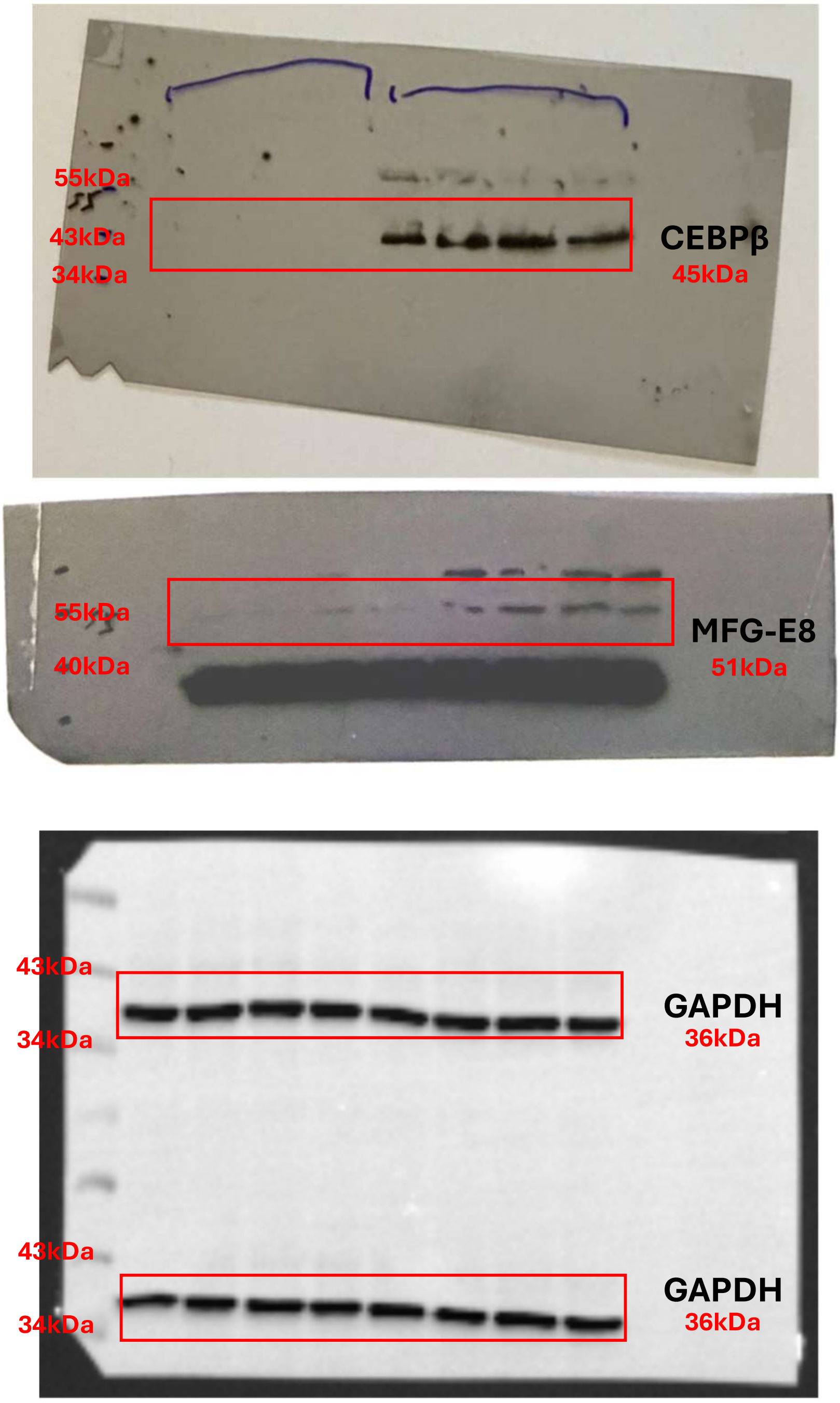

Figure S16 – Full unedited representative and quantification immunoblots continued

Figure S16G

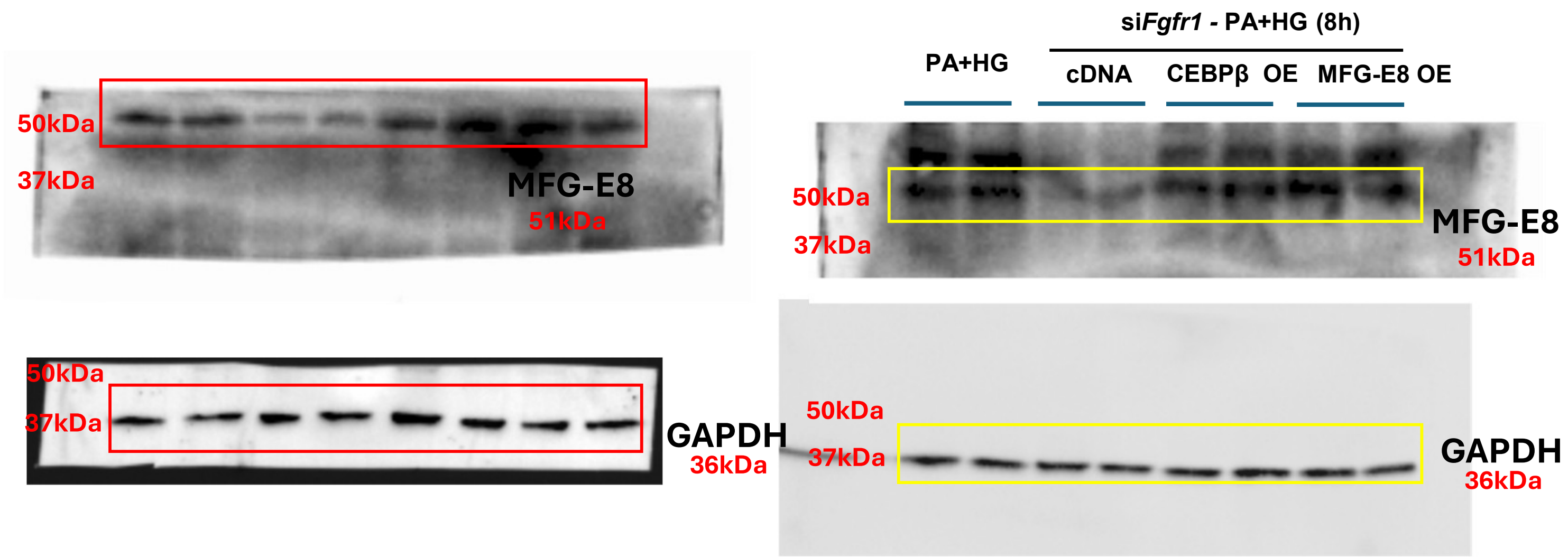

Figure S16H

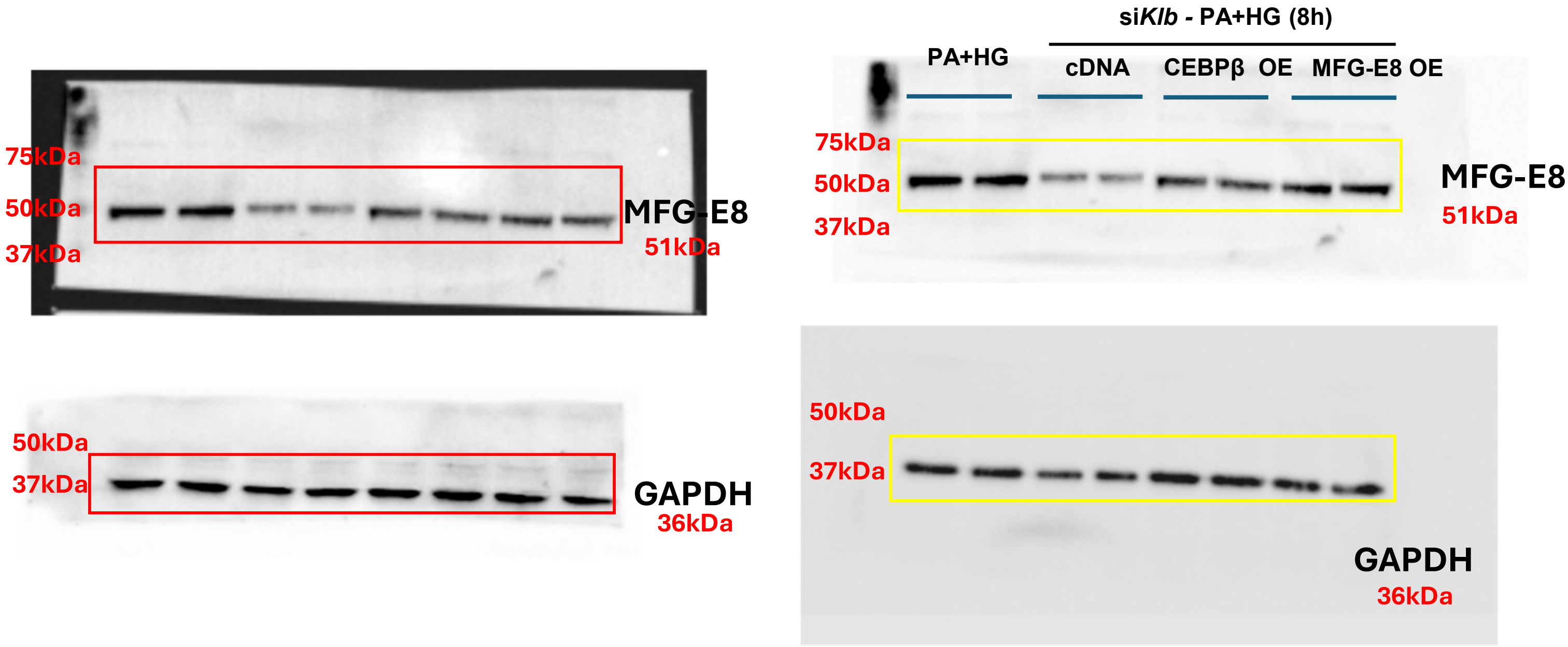

Figure S17 – Full unedited representative immunoblots

Figure S17E

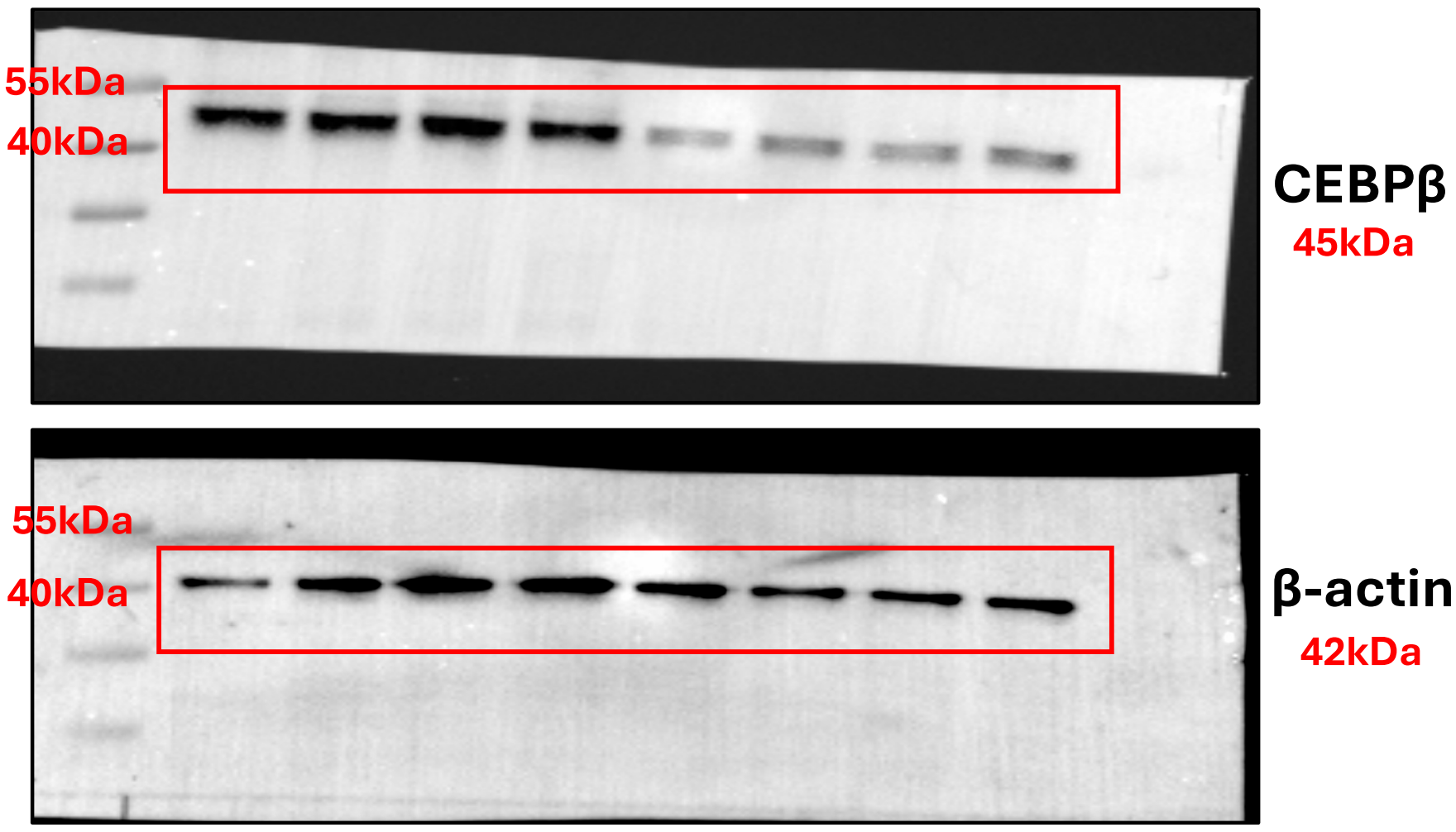

Figure S21 – Full unedited representative immunoblots

Figure S21B

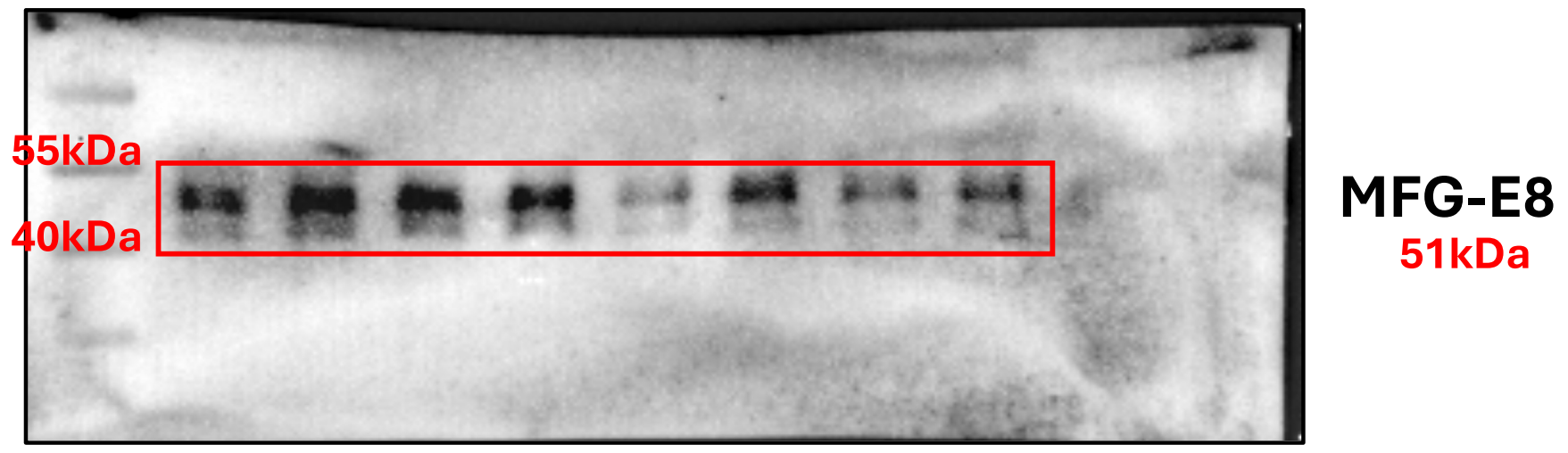

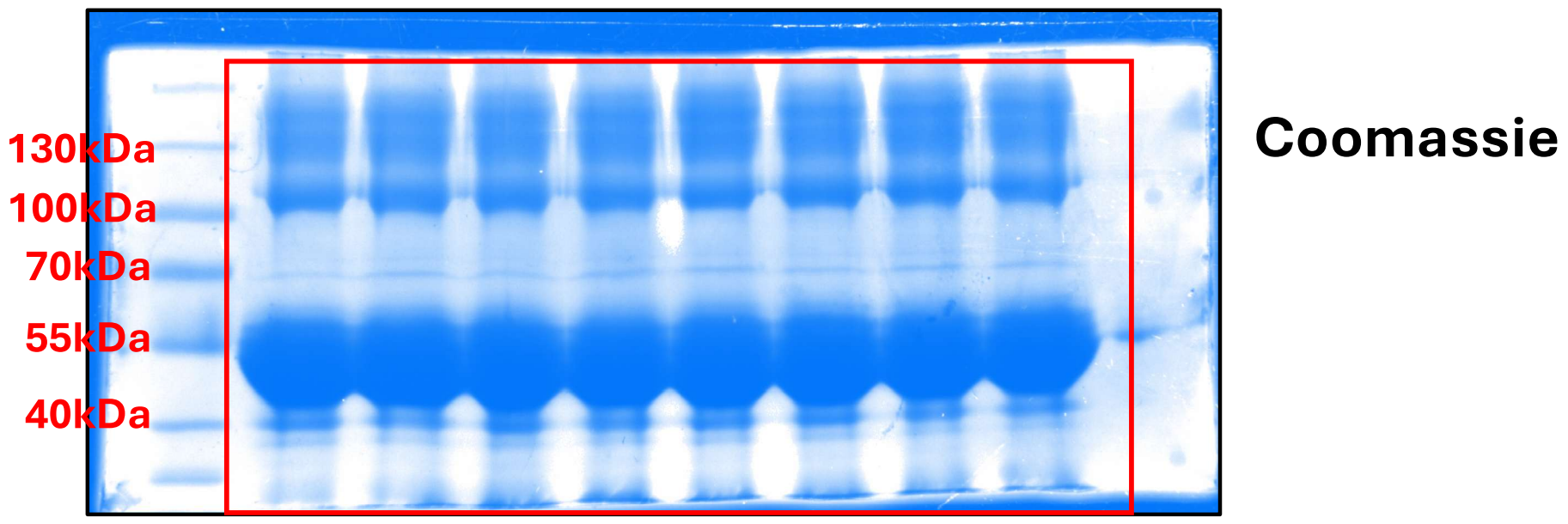

Figure S23 – Full unedited representative immunoblots

Figure S23A

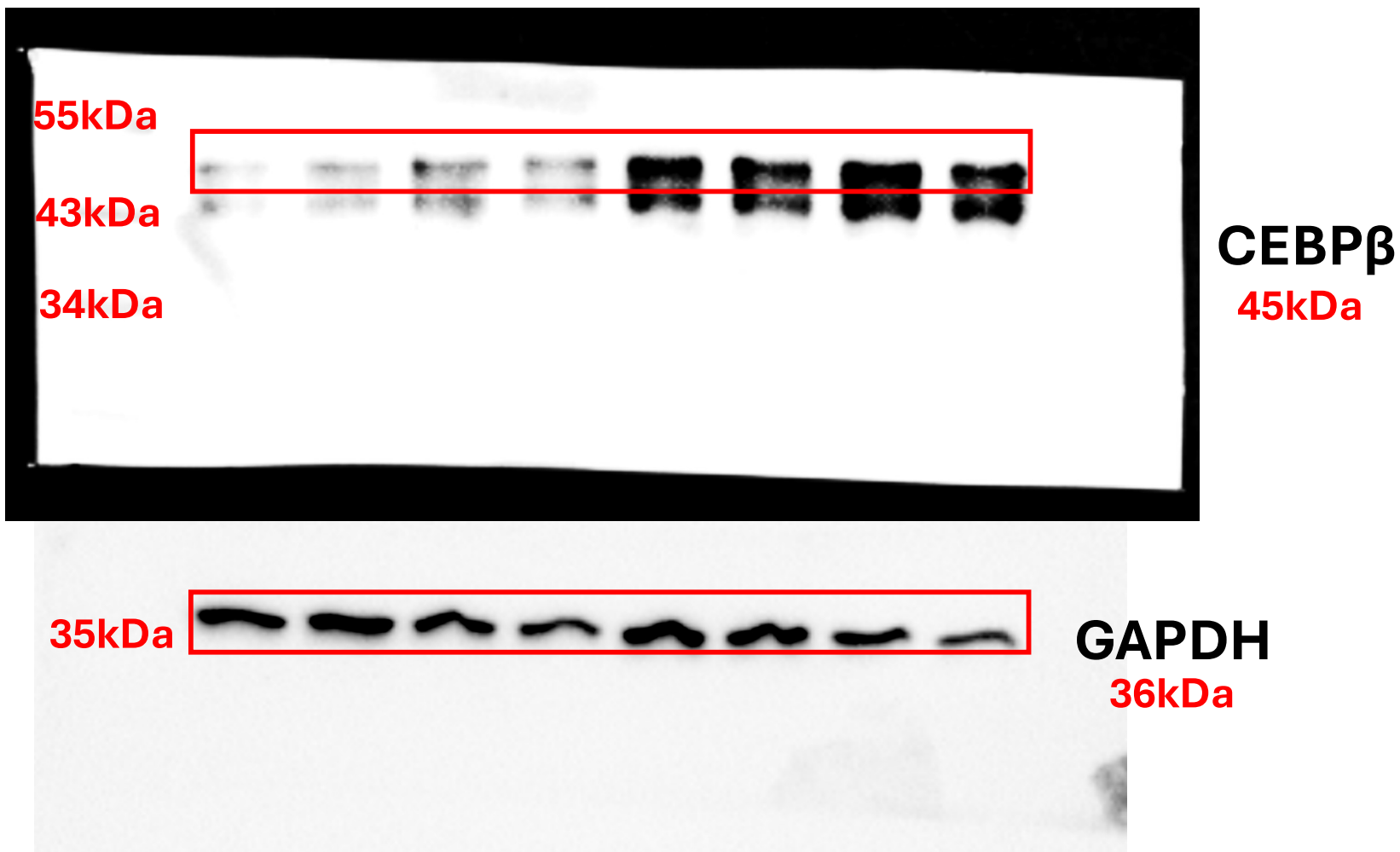

Figure S23B

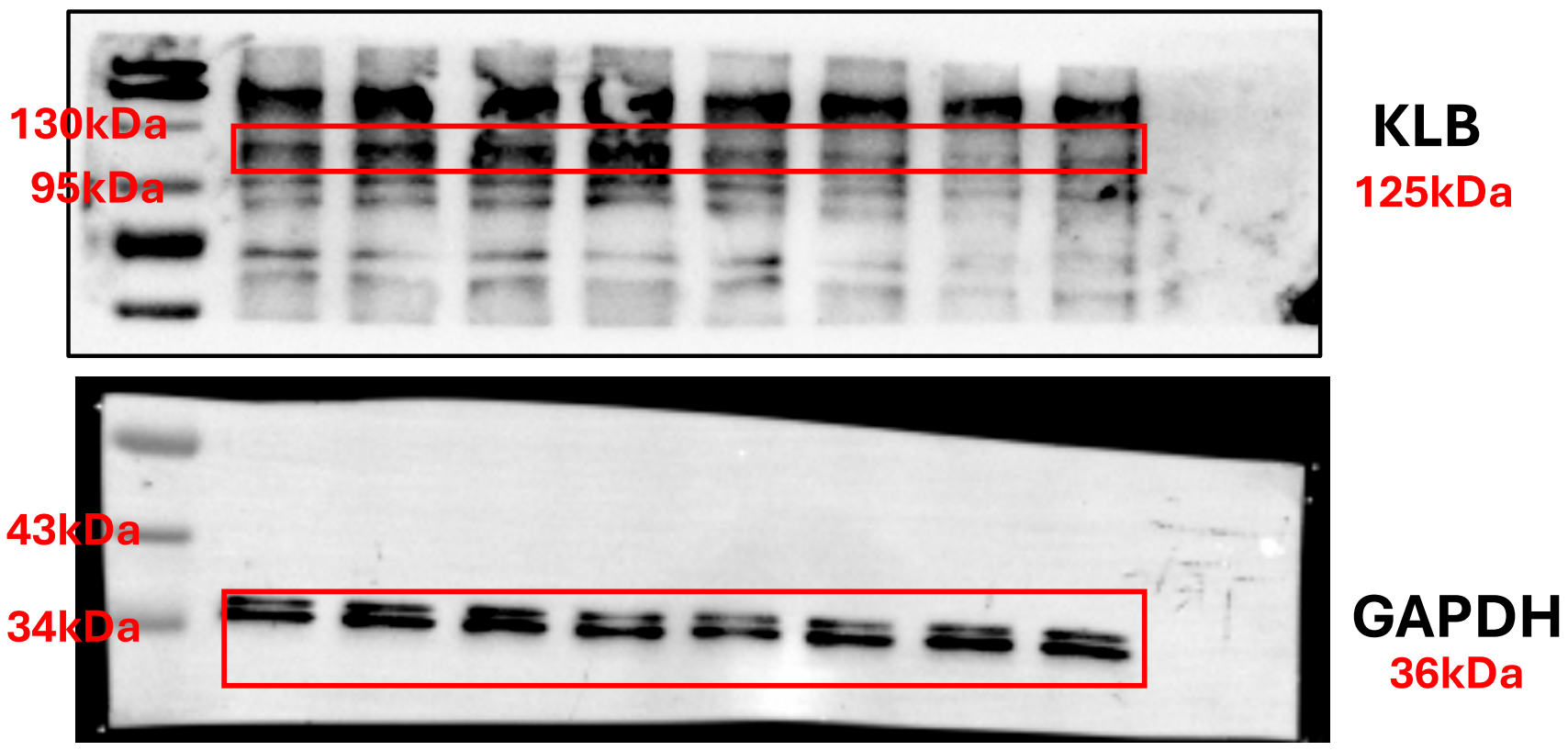

Figure S23C

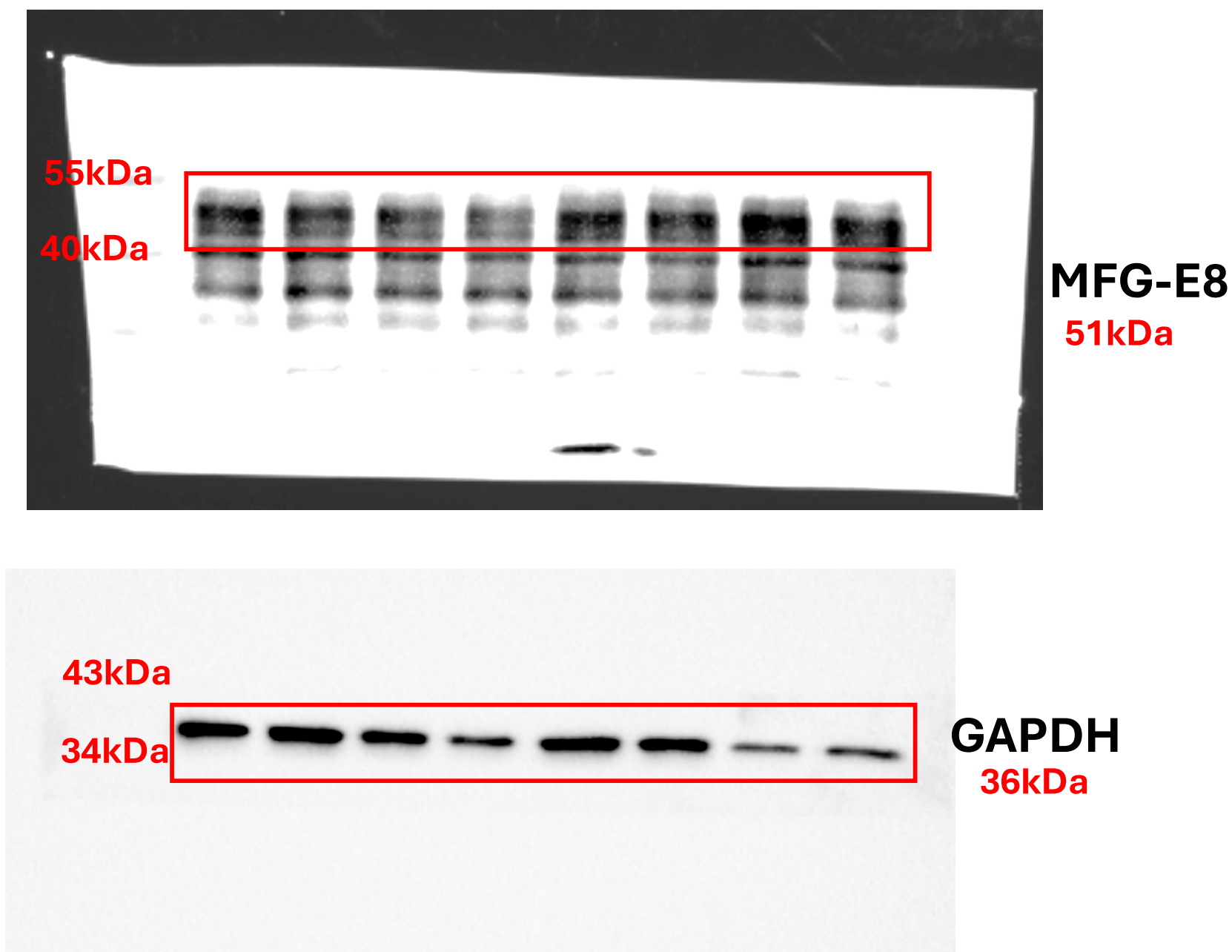

Supplement: Supplementary file 1 [file res-139-e327950-s001.pdf]
