# Supplementary material for: FGFR1/KLB-MFG-E8 Maintains Microvascular Integrity and Adaptive Cardiac Remodeling
Source: Circ Res. 2026 Sep 29;139(10):e327950. doi: 10.1161/CIRCRESAHA.125.327950 (PMC13633393; doi:10.1161/CIRCRESAHA.125.327950)
Supplement: Supplementary file 2 [file res-139-e327950-s004.pdf]

Supplemental Methods
Animal Studies
All animal experiments were conducted under UK Home Office Project License PA8E7CA4E in accordance with the United Kingdom Animals (Scientific Procedures) Act 1986 and the ARRIVE guidelines. Experiment protocols were designed by investigators and approved by the University of Manchester Ethics Committee and institutional animal welfare officers. Mice and rats were housed in a pathogen-free facility at the University of Manchester with food and water provided *ad libitum* in a 12-hour light/dark cycle. Pregnant Sprague-Dawley rats and C57BL/6J mice were purchased from Envigo (UK). This study utilized two strains of transgenic mice. Cardiomyocyte-specific fibroblast growth factor 1 (Fgfr1) knockout mice ($FGFR1^{cKO}$) were generated by mating hetero-floxed *Fgfr1* (*$Fgfr1^{fl/+}$*) mice (Jackson Laboratory, stock #007671, RRID:IMSR_JAX:007671) with transgenic mice expressing Cre under the α-myosin heavy chain promoter (αMHC-Cre) over more than 5 generations. Cardiomyocyte-specific beta klotho (Klb) knockout mice (KLB^{cKO}) were generated by mating floxed *Klb* mice (*$Klb^{fl/fl}$*, a gift from Prof. Steven Kliewer, University of Texas Southwestern Medical Centre, USA[48] with transgenic αMHC-Cre mice over more than 5 generations. Floxed littermates ($FGFR1^{Flox}$ and KLB^{Flox}) were used as controls for their respective experiments. Additional wild-type C57BL/6J mice were purchased from Envigo, Manchester. Male and female mice aged 7-8 weeks were used in all experiments following weaning. Humane endpoints included lethargy, impaired gait, dyspnea, blood glucose >600 mg/dL, >10% body weight loss, seizures, and other signs of distress. No adverse events were observed during the study. Around 280 animals were used. Sample sizes were determined based on power calculations from pilot data (power >80%, α=0.05). Apart from strain, age, and sex, no exclusion or inclusion criteria were applied. All animals used are represented in the data. Each data point represents one animal. Animals were randomly assigned to experimental groups using an online randomization tool. Investigators were blinded to genotypes and treatments during data collection and analysis. Potential confounders were not specifically controlled.

Neonatal Rat Cardiomyocytes (NRCMs)
NRCMs were isolated from 2- to 3-day-old Sprague-Dawley rats (male and female) as previously described.[55] Briefly, excised hearts were dissected and subjected to sequential serial cycles of enzymatic digestion in 7 mL of buffer [116 mmol/L NaCl, 20 mmol/L HEPES, 1 mmol/L NaH_2PO_4, 6 mmol/L glucose, 5 mmol/L KCl, 0.8 mmol/L $MgSO_4$, pH 7.4], supplemented with 0.33 U/mL collagenase A and 100 mg/mL pancreatin. Isolated NRCMs were cultured in a DMEM-based medium containing 80% (v/v) DMEM, 20% (v/v) M199, 1% (v/v) FBS, 100 µg/mL streptomycin, 100 U/mL penicillin, 1% (v/v) fungizone, and 1 µmol/L bromodeoxyuridine (see the Major Resources Table in the Supplemental Materials).

Human Induced Pluripotent Stem Cells-derived Cardiomyocytes (hiPSC-cardiomyocytes)
The hiPSC 02C9 was generated from adult human dermal fibroblasts (Invitrogen) using the CytoTune-iPS 2.0 Sendai Reprogramming Kit according to the manufacturer's instructions. The hiPSCs were maintained and differentiated into cardiomyocytes as described previously.[75] In short, hiPSCs were cultured in mTeSR Plus media on Geltrex-coated plates (Life Technologies, A1413302) and differentiated into cardiomyocytes using 4 µM CHIR99021 and 5 µM IWP2 for 48 hours each, in RPMI1640 HEPES GlutaMAX medium with B27 minus insulin supplement. After successful differentiation, hiPSC-cardiomyocytes were maintained in RPMI1640 HEPES GlutaMAX medium with B27 supplement, 100 µg/mL streptomycin, and 100 U/mL penicillin for 14-21 days before experiments (see the Major Resources Table in the Supplemental Materials).

Human Embryonic Stem Cells-derived Cardiomyocytes (hESC-cardiomyocytes)
H9 human embryonic stem cells (WA09; WiCell) were differentiated into cardiomyocytes using temporal WNT modulation. Briefly, cells were treated with 6 µmol/L CHIR99021 in RPMI1640 HEPES GlutaMAX medium with B27 minus insulin supplement for 2 days, followed by 2

µmol/L C59 (see the Major Resources Table in the Supplemental Materials) from Day 2-4. Cells were then maintained in RPMI1640 HEPES GlutaMAX medium with B27 supplement till Day 14 prior to experiments.[76]

Human Umbilical Vein Endothelial Cells (HUVEC)
HUVECs (PromoCell, C-12200) were cultured in endothelial growth medium (see the Major Resources Table in the Supplemental Materials) supplemented with a low serum supplement mix (PromoCell, C-39215), 100 µg/mL streptomycin, and 100 U/mL penicillin. Cells were authenticated by the supplier and routinely tested to confirm the absence of mycoplasma contamination. Experiments were performed using cells between passages 3 and 6. For treatment experiments, HUVECs were serum-starved overnight in supplement-free endothelial media.

Human Embryonic Kidney 293A (HEK293A) and H9C2 Cells
HEK293A (Invitrogen, R70507) and H9C2 (Sigma-Aldrich, 88092904) cells (see the Major Resources Table in the Supplemental Materials) were maintained in DMEM (Gibco, 11966) supplemented with 10% (v/v) heat-inactivated fetal bovine serum (FBS), 100 µg/mL streptomycin, and 100 U/mL penicillin. All cell lines were regularly tested for mycoplasma contamination and authenticated according to supplier guidelines.

Human Samples
All experimental procedures involving human samples received approval from the University of Manchester Research Ethics Committees and were carried out in accordance with the principles of the Declaration of Helsinki. Human hearts were obtained from the United Network for Organ Sharing (UNOS) through IIAM and Novabiosis, with next of kin informed consent. Human hearts were classified according to the following inclusion and exclusion criteria: Control healthy donors: inclusion criteria: 1) aged below 55, and 2) ejection fraction>55%; exclusion criteria: 1) history of myocardial infarction, 2) pregnant women, 3) decompensated heart failure, 4) severe kidney disease, 5) asthma or severe chronic lung disease, 6) cardiac pacemaker or implantable defibrillator, 7) cerebral aneurysm clip, 8) neural stimulator, and 9) history of diabetes. HF donors: inclusion criteria: 1) aged 50 or above, 2) history of diabetes, 3) ejection fraction≤55%; exclusion criteria: 1) history of myocardial infarction, 2) pregnant women, 3) asthma or severe chronic lung disease, 5) cerebral aneurysm clip, and 6) neural stimulator. The tissue samples (in table below) were embedded in optimal cutting temperature (OCT) embedding matrix and sectioned at a thickness of 10 µm.

	Age	Sex	Ethnicity	EF	Comorbidities	BMI	Cause of Death
**Control**							
1	36	Male	Black or African American	60	N/A	22.71	Cerebrovascular/Stroke
2	54	Female	White or Caucasian	58	N/A	24.98	Gunshot wound
3	37	Male	Black or African American	70	N/A	24.943	Cerebrovascular/Stroke
**HF**							
1	53	Male	White	55	Hypertension and diabetes mellitus	33.386	Anoxia
2	61	Male	Caucasian	50	Hypertension and diabetes mellitus	30.9	Intracerebral Hemorrhage /Stroke
3	56	Female	Hispanic	55	Hypertension and diabetes mellitus	38.7	Cerebrovascular/Stroke

4	52	Male	Hispanic	55	Hypertension, diabetes mellitus, and chronic kidney disease	36.59	Anoxia
5	57	Female	African American	23	Hypertension, diabetes mellitus, and neuropathy	36.1	Cerebrovascular accident /Stroke

For RNA sequencing, snap-frozen human heart left ventricular tissue was purchased from Asterand (BioIVT, UK). Asterand obtained ethical approval and consent following the United Kingdom Human Tissue Authority regulations. Detailed inclusion and exclusion criteria, along with the donor demographics for healthy controls and HF samples, have been previously described.[75]

Adeno-Associated Virus 9 (AAV9) Gene Delivery
To achieve gene-specific overexpression in the heart, mice were systemically administered adeno-associated virus serotype 9 (AAV9) vectors via tail vein injection at varying doses. Cardiac-specific overexpression of *Fgfr1* was accomplished using an AAV9 vector encoding mouse *Fgfr1* cDNA (VectorBuilder) under the control of the human cardiac troponin T (cTnT) promoter (AAV9-cTnT-*mFgfr1*-Myc, Boston Children's Hospital), delivered at a dose of 1×10^{11} viral particles per mouse. For overexpression of *Klb*, mice received AAV9-cTnT-*mKlb*-3XFlag (Boston Children's Hospital), carrying mouse *Klb* cDNA (VectorBuilder), at a dose of 1×10^{11} viral particles per mouse. For dual overexpression, mice received both AAV9-cTnT-*mFgfr1* and AAV9-cTnT-*mKlb* simultaneously at the same dose. Cardiac-specific overexpression of *CEBPβ* was achieved using AAV9-cTnT-*hCEBPB*,[54] which contains human *CEBPB* cDNA, administered at a dose of 1×10^{11} viral particles per mouse, as described previously.[77] Control mice received AAV9 encoding enhanced *Gfp* (AAV9-hTnT-e*Gfp*) under the cTnT promoter.

All AAVs, serotype 9, were generated according to previously described methods.[78,79] Briefly, HEK293T cells were co-transfected with the three plasmids of the AAV Helper-Free system (pAAV-GOI, pAAV-RC9, pAAV-helper) in a 1:1:1 molar ratio. Cells were collected 3 days later, and cell nuclei were extracted and lysed with high-salt buffer and sonicated. After pelleting cellular debris and clearance of DNA/RNA, AAVs were separated from the lysate using a discontinuous iodixanol gradient and filtered and concentrated using Millipore Amicon Ultra filter units (UFC910008; 100,000 MWCO). AAVs were then aliquoted and stored at -80°C

Induction of Diabetes
Male and female mice aged 7-8 weeks were randomly assigned to receive either a standard chow diet (Envigo, 2018 Teklad Global 13 kcal% fat, 18 kcal% protein Rodent diet) or a high-fat, high-sucrose diet (HFHSD; SDS, 82408, 45 kcal% fat, 20 kcal% sugar) *ad libitum* for 8-16 weeks according to experimental purpose indicated in Results. To induce a clinically relevant model of T2DM, mice on the HFHSD received intraperitoneal injections of streptozotocin (STZ; 40 mg/kg body weight) once daily for three consecutive days, beginning two weeks after the initiation of the HFHSD. This HFHSD+STZ model has been widely used to assess diabetes-induced complications, including cardiac and vascular dysfunction.[80] In a separate cohort, 6-week-old C57BL/6J mice were fed either a chow diet or a high-fat diet (HFD; SDS, 824054, 60 kcal% fat) *ad libitum* for 24 weeks.

Administration of Recombinant MFG-E8
To evaluate the therapeutic potential of MFG-E8 in diabetes-induced HF, recombinant MFG-E8 (see the Major Resources Table in the Supplemental Materials) was administered intraperitoneally to $FGFR1^{cKO}$ and KLB^{cKO} mice at a dose of 40 μg/kg body weight/week. The

dosing regimen and route of administration were based on previous studies.[27-29] Treatment commenced after 4 weeks of HFHSD+STZ exposure and continued for an additional 4 weeks.

Metabolic Measurements
Glucose tolerance tests (GTTs) were performed after a 16-hour overnight fast. Mice received an intraperitoneal injection of glucose (2 g/kg body weight), and blood glucose levels were measured from the lateral tail vein at baseline (0 min) and at 30-minute intervals up to 120 minutes post-injection using an Accu-Chek Aviva glucometer (Roche Diagnostics).

Echocardiography
Cardiac function was assessed via transthoracic echocardiography under anesthesia (2% isoflurane in 100% oxygen at a 1.5 L/min flow rate). Two-dimensional M-mode and pulse-wave Doppler images were acquired using the Acuson Sequoia C256 ultrasound system. Left ventricular (LV) structure and function were evaluated by measuring systolic and diastolic posterior wall (sPW, dPW) and interventricular septal thickness (sIVS, dIVS), systolic and diastolic chamber dimensions (sD, dD), and heart rate (HR). Diastolic function was assessed by isovolumetric relaxation time (IVRT) and the ratio of the early (E wave) to late (A wave) diastolic filling velocities of the left ventricle (E/A ratio), while systolic function was evaluated by fractional shortening and ejection fraction. Sample size calculations were based on IVRT as the primary outcome measure. Additional echocardiographic parameters, including sex-specific analyses, are detailed in the Supplemental Tables for each cohort.

Paraffin Embedding and Sectioning of Blocks
Freshly excised hearts were transversely bisected, and the apical half was fixed in 4% paraformaldehyde, sequentially dehydrated in ethanol (75%, 90%, 99%), and cleared overnight in xylene. Tissues were embedded in paraffin wax, and 5 µm myocardial tissue sections were prepared for histological analysis using a Leica RM2135 microtome.

OCT Embedding and Sectioning of Blocks
Freshly collected human hearts were embedded in OCT embedding matrix and stored at -80°C for histological analyses. Cryosections were cut to a thickness of 10 µm using the Leica CM3050S Cryostat microtome.

Hematoxylin and Eosin (H&E) Staining
Paraffin-embedded heart sections were deparaffinized in xylene overnight and rehydrated through a graded series of industrial methylated spirits (IMS; 100%, 75%, and 50%, 10 minutes each). Sections were stained with Harris' hematoxylin (Sigma-Aldrich, HHS32) for 5 minutes, followed by differentiation in acid alcohol (1% v/v HCl in 70% ethanol) for 10 seconds. Counterstaining was performed using eosin (Sigma-Aldrich, HT110132) for 1 minute. Samples were then dehydrated through ascending concentrations of IMS (75%, 95%, and 100%), cleared in xylene, and mounted using Eukitt (Sigma-Aldrich).

Masson's Trichrome Staining
To assess total (interstitial and perivascular) fibrosis, Masson's Trichrome staining was performed. The heart sections were deparaffinized in xylene overnight and rehydrated as described above. Samples were fixed in Bouin's solution (Sigma-Aldrich, HT10132) for 2 hours, then stained with Harris' Hematoxylin (Sigma-Aldrich, HHS32) for 5 minutes, and differentiated with acid alcohol (1% [v/v] hydrochloric acid and 70% ethanol). Cytoplasm and muscle fibers were stained using a red solution composed of 0.9% Biebrich scarlet, 0.1% fuchsin, and 1% acetic acid (Sigma, HT151) for 5 minutes. Sections were differentiated in 2.5% (w/v) phosphomolybdic acid (Sigma, 221856) for 15 minutes and stained with aniline blue (Sigma-Aldrich, B8563) for 5 minutes to visualize collagen. Final differentiation was carried out with 1% glacial acetic acid for 30 seconds. Slides were dehydrated, cleared in xylene, and mounted using Eukitt (Sigma-Aldrich).

TUNEL Staining
Terminal deoxynucleotidyl transferase dUTP nick end labeling (TUNEL) assay was performed using the in-situ Cell Death Detection Kit to detect endothelial and cardiomyocyte apoptotic cells. Sections were deparaffinized, rehydrated, and incubated with proteinase K (0.02 mg/mL in PBS; ThermoFisher Scientific, AM2546) for 15 minutes at 37 °C for enzymatic antigen retrieval. Permeabilization was achieved with 0.1% (v/v) Triton X-100 and 0.1% (w/v) sodium citrate in PBS for 8 minutes. The sections were blocked with 10% (v/v) normal donkey serum (NDS) and 0.1% Triton-X in PBS for 1 hour at room temperature and incubated overnight in 5% (v/v) NDS at 4°C with different primary antibodies. After PBS washes, the sections were incubated with secondary antibodies for 1 hour in a humid chamber at room temperature and later incubated with TUNEL reaction mixture (1:100 dilution of enzyme solution in label solution) at 37°C for 1 hour in a humidified chamber. After washing, sections were mounted using ProLong™ Gold Antifade Mountant containing 4′,6-diamidino-2-phenylindole (DAPI) (see the Major Resources Table in the Supplemental Materials).

CD31 Immunohistochemical Staining
To assess capillary density, immunohistochemical staining for CD31 was performed on paraffin-embedded heart sections. Sections were deparaffinized in xylene overnight and rehydrated as described above. The sections were incubated in 3% hydrogen peroxide in methanol for 30 minutes to quench endogenous peroxidase activity. Heat-mediated antigen retrieval was performed by microwaving in TEG buffer (10 mmol/L Tris Base + 0.5 mmol/L EDTA, pH 9.0) for 5 minutes at 700 W, followed by incubation at 95 °C in a Techne Hybridizer for 10 minutes. Sections were then quenched in 50 mM ammonium chloride in PBS for 30 minutes at room temperature. Blocking was performed with 1% (w/v) BSA in PBS for 1 hour at room temperature. Sections were incubated overnight at 4 °C with rabbit anti-CD31 antibody (see the Major Resources Table in the Supplemental Materials) in 0.1% (w/v) BSA + 0.3% (v/v) Triton X-100. The following day, sections were washed and incubated with HRP-conjugated anti-rabbit secondary antibody (SignalStain Boost, 8114P, RRID:AB_10544930) for 1 hour at room temperature. Signal development was achieved using DAB substrate (SigmaFast, D4293-50SET). Slides were briefly rinsed in PBS, counterstained with Harris' hematoxylin for 5 minutes, dehydrated through ascending concentrations of IMS (75%, 95%, and 100%), cleared in xylene, and mounted using Eukitt (Sigma-Aldrich). $CD31^+$ cells were quantified per cardiomyocyte and expressed as capillaries per cell.

Immunofluorescent Staining on Mouse Paraffin Sections
Immunofluorescent staining was done on genetically modified FGFR1 and KLB mice. The heart sections were deparaffinized in xylene overnight and rehydrated as described above. Protein retrieval was done by incubating slides in 10 mmol/L sodium citrate buffer in PBS (pH 6.0) at 95°C in a Techne Hybridizer for 30 minutes. After a quick rinse, slides were washed with 50 mmol/L ammonium chloride (w/v) in PBS for 10 minutes to quench free aldehydes. Sections were blocked with 10% (v/v) NDS and 0.1% Triton-X in PBS for 1 hour at room temperature and incubated in 5% (v/v) NDS in PBS containing different primary antibodies overnight at 4°C. After quick PBS washes, sections were incubated with secondary antibodies (see the Major Resources Table in the Supplemental Materials) for 1 hour in a humid chamber at room temperature. After PBS washes, the sections were mounted with DAPI-containing ProLong™ Gold Antifade Mountant (P36935, ThermoFisher Scientific) under a coverslip. All antibodies used for immunostaining were commercially validated by the manufacturers, including validation in knockdown/knockout or target-deficient cells where available. In addition, secondary antibody-only control staining was performed to assess nonspecific background fluorescence and confirm specificity of the observed signal.

Immunofluorescent Staining of Human Cryosections
Immunofluorescent staining was performed on frozen human hearts to assess the impact of HF on CD31 expression. Cryosections were fixed using an acetone: methanol solution. Subsequently, sections were blocked in 10% (v/v) normal donkey serum (NDS) and incubated

with the CD31 primary antibody overnight in 5% (v/v) NDS. Sections were then incubated with the corresponding secondary antibody (Major Resources Table) in 5% (v/v) NDS, and slides were mounted with ProLong™ Gold Antifade Mountant containing DAPI (see the Major Resources Table in the Supplemental Materials). $CD31^+$ capillaries were quantified within defined areas (mm^2) on ImageJ.

Transmission Electron Microscopy (TEM)
Endothelial cells and pericyte coverage were detected using TEM. Freshly excised heart tissue was fixed in fixative solution containing 2.5% (v/v) glutaraldehyde and 4% (v/v) formaldehyde in 0.1 M HEPES buffer (pH 7.2). Tissues were then post-fixed in 0.1 M cacodylate buffer (pH 7.2) with 1% (w/v) osmium tetroxide and 1.5% (w/v) potassium ferrocyanide for 1 hour, followed by 1% tannic acid in 0.1 M cacodylate buffer (pH 7.2) for 1 hour, and finally in 1% uranyl acetate for 1 hour. Samples were dehydrated in ethanol and embedded in TAAB 812 resin and polymerized for 24 hours at 60°C. Sections were cut with a Reichert Ultracut Ultramicrotome, imaged with Gatan Orius SC1000 CCD camera, and analyzed using Talos L120C transmission electron microscope at 100 kV accelerating voltage.

Real-Time Quantitative Polymerase Chain Reaction (RT-qPCR)
Total RNA was extracted from human and mouse heart tissue, NRCMs or hiPSC-cardiomyocytes using Trizol reagent, followed by DNase treatment to remove genomic DNA contamination. Complementary DNA (cDNA) was synthesized from total RNA using LunaScript reverse transcription kit. Quantitative PCR was performed using 30 ng of cDNA with SYBR Select PCR Master Mix and specific primers (see the Major Resources Table in the Supplemental Materials), using the Applied Biosystems QuantStudio™ Flex System. The fold change was calculated using the comparative threshold cycle (ΔΔCt) method.[81] Target gene expression was normalized to phosphoglycerate kinase 1 (*PGK1*) for human samples and *18S* ribosomal RNA for mouse samples.

RNA Sequencing
Total RNA was extracted from the left ventricular tissue of control and HF human donors and subjected to RNA sequencing as described previously.[75] Due to the higher biological variability inherent to the human dataset and limited subject number, we considered a more lenient P value to indicate alterations between groups. Differentially expressed genes with adjusted p-values less than 0.1 ($P_{adj.}$<0.1 for HF versus controls) were used for subsequent pathway analyses using Enrichr (https://maayanlab.cloud/Enrichr/; RRID:SCR_001575). Altered pathways (p<0.1) were visualized as pathway enrichment dot plots generated in RStudio (RRID:SCR_000432), focusing on Gene Ontology (GO) pathways and associated biological processes. Finally, only pathways relevant to the focus of the current study theme were presented in the figure.

Total RNA from chow-fed $FGFR1^{Flox}$ mice and HFHSD+STZ-stressed $FGFR1^{Flox}$, $FGFR1^{cKO}$, and KLB^{cKO} mice was extracted using Trizol and treated with DNAse to eliminate genomic DNA contamination. The quality and integrity of RNA were initially checked using Agilent 2100 Bioanalyzer and library preparation was performed using the TruSeq Stranded mRNA Library Prep Kit (Illumina) employing poly(A)-based enrichment of mRNA with concurrent removal of rRNA as described previously.[55,82] Briefly, unmapped paired-end sequences from an Illumina HiSeq4000 / NovaSeq 6000 sequencer were quality controlled by in-house software using several open-source tools (https://github.com/fls-bioinformatics-core/auto_process_ngs). Sequence adapters were removed, and reads were quality trimmed using Trimmomatic_0.39.[83] The reads were mapped against the reference mouse genome (mm10/GRCm38), and counts per gene were calculated using annotation from GENCODE M25 (http://www.gencodegenes.org/; RRID:SCR_014966) using STAR_2.7.7a.[84] Normalization, Principal Components Analysis, and differential expression were calculated with DESeq2_1.40.2 (RRID:SCR_015687).[85] Adjusted p-values were corrected for multiple testing (Benjamini and Hochberg method), and adjusted p-values less than 0.1 ($P_{adj.}$<0.1) were

the default cutoff.[82] Thus, differentially expressed genes ($P_{adj.}$<0.1 in HFHSD+STZ stressed FGFR1^{cKO} and KLB^{cKO} mice and $P_{adj.}$<0.05 in FGFR1^{Flox} mice) were used for subsequent pathway analyses using Enrichr (https://maayanlab.cloud/Enrichr/; RRID:SCR_001575) or ShinyGo v0.82 (http://ge-lab.org/go/; RRID:SCR_019213). Significantly altered pathways ($P_{adj.}$<0.05) were used to generate the pathway enrichment dot plots using RStudio software (RRID:SCR_000432), focusing on Gene Ontology (GO) pathways and associated biological processes. Finally, the pathways closely relevant to the molecular mechanisms studied in the current project were presented.
The raw data are available at ArrayExpress (RRID:SCR_002964) under accession numbers E-MTAB-16722 and E-MTAB-16712.

Liquid Chromatography-Mass Spectrometry (LC-MS/MS)
Protein profiling was performed via LC-MS/MS as previously described[17] to assess treatment-induced changes in cardiac tissue from three experimental groups:
• Mice subjected to 24 weeks of HFD feeding vs chow-fed controls
• KLB^{cKO} mice treated with AAV9-*CEBPB* vs AAV9-*Gfp* treated KLB^{cKO} controls
• KLB^{cKO} mice treated with rMFG-E8 vs vehicle-treated controls

Briefly, 40 µg of protein was transferred to a 130 µL AFA tube (Covaris®, 520045) with 50 µL of S-trap solubilization buffer [5% (w/v) SDS in 7.55 triethyl ammonium bicarbonate]. The sample was disrupted using AFA sonication in a Covaris® LE220+ for 1 minute. The supernatant was quantified using Millipore Direct Detect and trapped in S-trap columns using 1.2% phosphoric acid and S-Trap binding buffer [90% aqueous methanol containing a final concentration of 100 mmol/L triethyl ammonium bicarbonate, pH 7.1]. Protein identification and quantification were performed using Thermo Proteome Discoverer (version 3.2.0.450; RRID:SCR_014477) with a CHIMERYS-based label-free quantification workflow. MS/MS spectra were prefiltered using a Top N Peaks Filter (Top 20 peaks per 100 Da window) and searched against the Mus musculus (sp_canonical, TaxID=10090, Release=407) FASTA database. Search parameters included a static modification of carbamidomethylation on cysteine (+57.021 Da), dynamic oxidation of methionine, a precursor mass tolerance of 20 ppm, fragment mass tolerance of 0.5 Da, peptide lengths of 7-30 amino acids, charge states 1-4, and up to two missed cleavages.

Peptide-spectrum matches (PSMs) and peptides were validated using q-value-based false discovery rate (FDR) control, with strict and relaxed FDR thresholds set at 0.01 and 0.05, respectively. Only peptides and master proteins passing the strict (1% FDR, high confidence) threshold were included in quantification and downstream analyses, and protein grouping was performed using strict parsimony. Label-free quantification was based on unique and razor peptides, using MS2 apex intensities. Abundances were normalized to the same total peptide amount per channel and scaled so that the average abundance per protein and peptide was 100. No imputation was performed for missing values. Statistical analysis included background-based t-tests for hypothesis testing. Only proteins and protein groups passing a 1% FDR were included in the final results, and peptides required a minimum length of six amino acids and high confidence. Additionally, the analysis in Supplemental Figure 1A was based on analysis from six biological samples per group. Equal amounts of protein from phenotypically similar animals were pooled prior to analysis to minimize inter-individual variability. This resulted in two pooled experimental replicates per group (N=2), and statistical analyses were therefore performed on the pooled replicates rather than the original individual samples. Differentially expressed proteins ($P_{adj.}$<0.05 in KLB^{cKO} mice and $P_{adj.}$<0.1 in mice after a 24-week HFD) were selected and further analyzed using Enrichr (https://maayanlab.cloud/Enrichr/; RRID:SCR_001575) or ShinyGo 0.82 (http://ge-lab.org/go/; RRID:SCR_019213). Significantly altered pathways ($P_{adj.}$<0.05) were used to generate the pathway enrichment dot plots using RStudio software (RRID:SCR_000432), focusing on Gene Ontology (GO) pathways and associated biological processes. The mass spectrometry proteomics data have been deposited to the ProteomeXchange Consortium via the PRIDE

repository (RRID:SCR_012052) with the dataset identifier PXD075219 and 10.6019/PXD075219.

Stimulation with Fatty Acids and High Glucose in Cells
Palmitic acid Major Resources Table) was complexed with 0.5% (w/v) fatty acid-free bovine serum albumin at a molar ratio of 7:1 to mimic pathophysiological states. 0.5% (w/v) bovine serum albumin served as the control. NRCMs, hiPSC-cardiomyocytes and hESC-cardiomyocytes were exposed to palmitic acid (500 µmol/L) and high glucose (25 mmol/L) or control medium for the various durations as stipulated in the Results.

Treatment on Cells
Before immunofluorescent cardiac troponin T staining, H9C2s were treated with recombinant mouse MFG-E8 (rMFG-E8) at a concentration of 1 µg/mL for 24 hours. For LEGENDPlex™ Angiogenesis Assay, HUVECs were treated with 1 µg/mL anti-MFG-E8 antibody for 4 hours before analysis. Treatment doses were selected based on previous studies.[86] To assess the FGFR1/KLB pathway in response to FGF21, recombinant FGF21 (rFGF21) was administered at a concentration of 500 ng/mL for 24 hours. The dose for treatment was selected based on prior studies (see the Major Resources Table in the Supplemental Materials).[17]

Additionally, NRCMs were treated with lysosomal inhibitor chloroquine (50 µmol/L) or the proteasomal inhibitor MG132 (10 µmol/L) in the presence of cycloheximide (10 µg/mL) (see the Major Resources Table in the Supplemental Materials) to determine the degradation pathways responsible for FGFR1 and KLB downregulation under diabetes-like stress conditions.

Adenovirus Production
PacI linearized plasmids of mouse FGFR1 (VectorBuilder, pAdv-CMV-m*Fgfr1*-Myc) and mouse KLB (VectorBuilder, pAdv-CMV-m*Klb*-3XFlag) were transfected into HEK293A cells using Lipofectamine™ 2000 (see the Major Resources Table in the Supplemental Materials). Viral plagues were harvested 7 days post-transfection and amplified in HEK293A cells. Adenoviruses were purified using cesium chloride (CsCl) gradient centrifugation (1.45 g/mL and 1.33 g/mL CsCl, 100,000 g, 18 hours) using a SW40 rotor and swing-bucket Beckman Optima. Final viral preparations were dialyzed for 2 hours using dialysis tubing (Medicell, cellulose 14 kDa, 28.7 mm diameter) soaked in the chemical exchanging buffer [10 mmol/L Tris base, 1 mmol/L $MgCl_2$, 135 mmol/L NaCl, 10% (v/v) glycerol, pH 7.4].

Overexpression of Genes
To overexpress CEBPβ or MFG-E8, NRCMs were transfected with 2.5 µg human *CEBPB* (isoform of LAP, RRID:Addgene_15738) or human *MFGE8* cDNA (RRID:Addgene_15738) using FuGENE® 4K Transfection reagent, while hiPSC-cardiomyocytes were transfected using Lipofectamine Stem Transfection Reagent, per manufacturer's protocols. FGFR1 (Source Bioscience, IRATp970D123D) or KLB (RRID:Addgene_45531) or HA-ubiquitin-WT (RRID:Addgene_17608) or HA-ubiquitin-KO plasmid (RRID:Addgene_17603) was transfected into H9C2 using Lipofectamine 2000 (see the Major Resources Table in the Supplemental Materials). FGFR1 and KLB were overexpressed in NRCMs and hiPSC-cardiomyocytes by infection with Adv-*Fgfr1*-Myc (adenovirus overexpressing FGFR1) and Adv-*Klb*-Flag (adenovirus overexpressing KLB), respectively, at a multiplicity of infection (MOI) of 50 for 48 hours. Controls included p3xFLAG-CMV-7.1 and Adv-*LacZ* for plasmid and adenovirus experiments, respectively.

siRNA Knockdown of Genes
In NRCMs, FGFR1, KLB, and CEBPβ were knocked down using 50 nmol/L Silencer Select Pre-designed rat si*Fgfr1,* rat si*Klb,* and rat *siCebpb* delivered via Lipofectamine LTX & Plus Reagent following the manufacturer’s instructions. In hiPSC-cardiomyocytes, KLB knockdown was achieved using Lipofectamine Stem Transfection Reagent-mediated transfection with 50

nmol/L of human si*Klb* (see the Major Resources Table in the Supplemental Materials). A non-targeting *siRNA* (si*Negative*; sequence: AGGUAGUGUAAUCGCCUUG) served as the control. Cells were incubated for 48 hours post-transfection at 37°C before further treatments.

Angiogenesis Assay
To evaluate the paracrine effects of treated cardiomyocytes on endothelial angiogenesis, a tube formation angiogenesis assay (see the Major Resources Table in the Supplemental Materials) was performed following the manufacturer's instructions. Conditioned media were collected from treated NRCMs or hiPSC-cardiomyocytes (as described in the Results). HUVECs were seeded at 30,000 cells/well in a black-walled 96-well plate and incubated with a 1:1 mixture of conditioned hiPSC-CM media and HUVEC media. After 16 hours, staining dye was added for 30 minutes, and tube structures were acquired on an Eclipse Ti inverted microscope (Nikon) using a 10× objective. Tube length and other additional parameters for angiogenesis assay (presented in the Supplemental Tables for each cohort) were assessed using Wimasis Imaging software.

Cell Viability Assay
HUVEC viability following exposure to NRCM-conditioned media was assessed using the CellTiter-Glo® Luminescent Cell Viability Assay (see the Major Resources Table in the Supplemental Materials) according to the manufacturer's guidelines. NRCMs-treated media were collected as described in the Results. HUVECs were plated at a density of 25,000 cells/well in an opaque-walled 96-well plate and incubated with conditioned media for 6 hours. CellTiter-Glo® Reagent was added and subsequently incubated at room temperature for 10 minutes. Luminescence was determined utilizing a Promega GloMax® Explorer plate reader.

Cytotoxicity Assay
HUVEC cytotoxicity was determined using the CellTox™ Green Cytotoxicity Assay (see the Major Resources Table in the Supplemental Materials). NRCM-treated conditioned media were collected as described in the Results. HUVECs were seeded at 10,000 cells/well in a black-walled 96-well plate and incubated with conditioned media for 6 hours. CellTox™ Green reagent was then added, followed by a 15-minute dark incubation. Fluorescence was measured using a Promega GloMax® Explorer plate reader (Ex/Em: 475/500-550 nm).

Cytokine Array
To analyze cytokine profiles in murine heart tissue from the FGFR1 strain, a label-based cytokine array (see the Major Resources Table in the Supplemental Materials) was conducted per the manufacturer's protocol. Hearts from the following experimental groups were used:
• $FGFR1^{Flox}$ HFHSD+STZ vs $FGFR1^{Flox}$ Chow
• $FGFR1^{cKO}$ + AAV9-*Fgfr1* HFHSD+STZ vs. $FGFR1^{cKO}$ HFHSD+STZ

Pooled heart tissue samples were purified by centrifugation at 1,000 g, labeled for 30 minutes, and quenched. Membranes were blocked for 1 hour, incubated with samples for 2 hours, and then incubated with HRP-conjugated streptavidin for 2 hours. Chemiluminescence was visualized using a ChemiDoc MP Imaging System (Bio-Rad) or Carestream BioMax XAR film (F5513-50EA, Kodak) in Hypercassette (Amersham Biosciences) to visualize cytokine intensity via chemiluminescence. Raw signal intensities were normalized to Z scores and cytokines significantly altered between the two experimental groups in each cohort (q<0.05) were presented in a heatmap.

MFG-E8 ELISA
Rat MFGE8 ELISA (see the Major Resources Table in the Supplemental Materials) was conducted on the media obtained from NRCMs following treatments as indicated in the Results section. Samples were incubated with rat MFG-E8 antibody and streptavidin-HRP for 1 hour at 37°C. Following sequential washes, the plate was incubated with substrate solutions A and B for 10 minutes at 37°C, followed by the Stop solution. Optical density was measured

at 450 nm using a Promega GloMax® Explorer plate reader. Mouse MFG-E8 ELISA (see the Major Resources Table in the Supplemental Materials) was conducted on mouse serum, heart, liver, and brain tissue lysates from the different mouse models described in the Results. Briefly, mouse serum samples were diluted 1:100 in the sample dilution buffer while 1-3 mg/mL of tissue lysates used for analysis. Standards or diluted sample were added to the pre-coated 96-well strips and incubated for 90 minutes at 37°C. After washing, biotin-labelled detection antibody was added and incubated for 60 minutes at 37°C. Plates were then washed and incubated with HRP-streptavidin (SABC) working solution for 30 minutes at 37°C. After washes, TMB substrate was added and incubated for 15 minutes at 37°C, followed by the addition of stop solution. Optical density was measured at 450 nm using a Promega GloMax® Explorer plate reader.

LEGENDplex™ Angiogenesis Assay

A 10-plex LEGENDplex™ angiogenesis assay (see the Major Resources Table in the Supplemental Materials) was conducted to quantify secreted angiogenic factors from HUVECs exposed to human cardiomyocyte-conditioned media. hiPSC-cardiomyocytes were treated as detailed in the Results; conditioned media were applied to HUVECs (200,000 cells/well, 24-well plate) for 4 hours. Media were then replaced with fresh endothelial media for an additional 4 hours. Collected media were assayed in a V-bottom plate. Samples were incubated with premixed beads for 2 hours, followed by detection antibody (1 hour) and SA-PE (30 minutes). After final washes, samples were analyzed using the Luminex Guava easyCyte™ System. Gating was applied to differentiate between the 10 beads, and data were analyzed using BioLegend's LegendPlex™ Data Analysis Software. Cytokines significantly altered between the two experimental groups in each cohort (q<0.05) were presented in a heatmap showing fold changes.

Luciferase Reporter Assay

To evaluate the regulatory effect of CEBPβ on *Mgfe8* transcription, two luciferase reporter constructs were generated, each containing ~1,000 bp upstream of the transcription start site (TSS) of mouse *Mgfe8*. Promoter regions were amplified from mouse genomic DNA using two sets of primers:
Set 1: 5'-GTCGGTACCGGCCTTTTCATGGTTT-3' and
5'-GTCCTCGAGGAGCCACGCCCTGATG-3' and
Set 2: 5'-GTCGGTACCCTCCTTGTTCTTTTTCCA-3' and
5'-GTCCTCGAGTGCCTGTTACTGGTTATG-3'

The resulting PCR product was subcloned into the pGL3-Promoter luciferase vector (Promega, E1751, RRID:Addgene_172006) at the *KpnI* and *XhoI* restriction sites. Three additional site-directed mutations were introduced into putative CEBPβ binding regions within the above MFGE8 promoter pGL3 constructs. Nucleotides 496-504 (5′-TTCTGTAAT-3′) in region 1, and nucleotides 137-145 (5′-TTTTGAAAG-3′) and 671-679 (5′-TTCAGGAAG-3′) in region 2 were substituted with 5′-AAAAAAAAA-3′ to disrupt the predicted CEBPβ binding sequence. Purified vector (20 ng) and the corresponding gBlock (10 ng, Integrated DNA Technologies) were assembled using the NEB HiFi DNA Assembly Mix (New England Biolabs, E2621S) according to the manufacturer's instructions. Plasmids were isolated from single colonies using the SmartPure Plasmid Kit (Eurogentec, SK-PLPU-100), and sequences were verified via whole-plasmid sequencing (Genewiz, Plasmid-EZ).

The empty pGL3-Promoter vector served as a negative control. Reporter plasmids were co-transfected with a Renilla luciferase control plasmid into NRCMs using Lipofectamine 3000 (ThermoFisher Scientific, L3000015). Following CEBPβ overexpression, luciferase activity was assessed using the Dual-Luciferase Reporter Assay System (Promega, E1910), and luminescence was measured with a GloMax® Explorer multimode plate reader (Promega).

Immunofluorescent Staining of Cells

Cellular co-immunofluorescent staining was performed on H9C2s, NRCMs, hiPSC-cardiomyocytes, or hESC-cardiomyocytes following treatments as stipulated in the Results. Cells were fixed with cold methanol (4°C, 20 minutes), blocked in 1% BSA (w/v in PBS), and incubated overnight at 4°C with primary antibodies diluted in 0.5% (w/v) BSA. Fluorescent-conjugated secondary antibodies (see the Major Resources Table in the Supplemental Materials) in 0.5% (w/v) BSA were applied for 1 hour at room temperature. The coverslips were then washed with 0.5% (w/v) BSA in PBS and mounted with ProLong™ Gold Antifade Mountant with DAPI.

Regarding CD31 staining of HUVECs, the cells were fixed with 4% PFA, permeabilized with 0.5% (w/v) BSA and 0.1% (v/v) Tween 20 in PBS and blocked with 3% (w/v) BSA for 1 hour. The cells were incubated overnight at 4°C with Alexa Fluor® 488-conjugated CD31 primary antibody (see the Major Resources Table in the Supplemental Materials) in 3% (w/v) BSA. The coverslips were then washed with PBS and mounted with ProLong™ Gold Antifade Mountant with DAPI.

Protein Lysate Extraction and Immunoblotting

Total protein tissue lysates were prepared utilizing Triton lysis buffer [137 mmol/L NaCl, 20 mmol/L Tris, 0.1% (w/v) SDS, 2 mmol/L EDTA, 10% (v/v) glycerol, 1% (Triton-X), 25 mmol/L glycerophosphate, protease and phosphatase inhibitor cocktail, pH 7.4]. Membrane protein lysates were isolated using differential centrifugation. The lysates were obtained by homogenizing with cytoplasmic buffer (20 mmol/L Tris, 10 mmol/L NaCl, 2 mmol/L $MgCl_2$, 2 mmol/L EDTA, 25 mmol/L β-glycerophosphate, protease and phosphatase inhibitor cocktail, pH 7.4) and were subjected to a series of centrifugations at increasing speeds. After disposal of nuclei and mitochondria/lysosomes/peroxisomes by centrifugation at 2,000 g and 10,000 g, respectively, plasma membrane fractions were enriched by centrifugation at 100,000 g. NRCM nuclear fraction isolation was also performed using differential centrifugation. Cells lysed in a cytoplasmic buffer were centrifuged at 5,000 g for 5 minutes, and pellets were resuspended in nuclear extraction buffer (pH 7.4), composed of 50 mmol/LTris, 500 mmol/L NaCl, 2 mmol/L EDTA, 10% glycerol, 1% Triton X-100, 25 mmol/L β-glycerophosphate, and protease and phosphatase inhibitor cocktail. Following incubation, samples were centrifuged at 10,000 g for 10 minutes, and the supernatants containing nuclear proteins were collected for subsequent analyses. Proteins were precipitated from conditioned media by adding methanol [1:1, (v/v)] and chloroform [1:0.25, (v/v)], followed by centrifugation at 14,000 g for 10 minutes at 4°C. The supernatant was removed, and the pellet was washed with methanol, recentrifuged, air-dried, and resuspended in distilled water for protein quantification.

Protein concentration was determined using Bradford assay (Bio-Rad, 500-0006). Approximately 30 µg of protein was loaded per lane for SDS-PAGE and transferred to membranes for immunoblotting. Primary antibodies and HRP-conjugated secondary antibodies (see the Major Resources Table in the Supplemental Materials) were diluted in 5% (w/v) fat-free milk in TBS-T. Signals were visualized using Amersham ECL Prime or Select reagents and imaged using a ChemiDoc MP Imaging System (Bio-Rad) or Carestream BioMax XAR film (F5513-50EA, Kodak) in Hypercassette (Amersham Biosciences). For media samples, total protein loading was confirmed using Coomassie blue staining.

Immunoprecipitation

To examine the ubiquitination of FGFR1 and KLB in human hearts or H9C2 cells under basal and PA+HG conditions following transfection of FGFR1 or KLB cDNA, in the presence of HA-ubiquitin-WT or HA-ubiquitin-KO plasmids, immunoprecipitation was performed. IP was conducted using Protein G agarose beads (20398, ThermoFisher Scientific) according to the manufacturer's instructions. Briefly, treated cells were fixed with 0.4% (w/v) PFA, and total protein was extracted using RIPA buffer [150 mmol/L NaCl, 50 mmol/L Tris-HCl, 0.1% (w/v) sodium dodecyl sulphate, 0.5% (w/v) sodium deoxycholate, 1 mmol/L EDTA, 1% (v/v) NP-40, 25 mmol/L β-glycerophosphate, 1 mmol/L sodium orthovanadate, 1mmol/L

phenylmethylsulphonyl fluoride, 0.00154 mmol/L aprotinin, and 0.0216 mmol/L leupeptin]. Approximately 2 mg of input protein was incubated with anti-ubiquitin antibody (see the Major Resources Table in the Supplemental Materials) to precipitate ubiquitinated proteins. Immune complexes were captured with Protein G agarose beads and eluted in 2xLaemmli sample buffer [125 mmol/L Tris-HCl, 4% (w/v) SDS, 20% (v/v) glycerol, 10% (v/v) β-mercaptoethanol, and bromophenol blue]. The precipitated proteins were subsequently analyzed by SDS-PAGE followed by immunoblotting using anti-FGFR1 or anti-KLB antibody.

Imaging of Histology

Paraffin-embedded sections stained with H&E, Masson's Trichrome, or CD31 immunohistochemistry were scanned using a 3D-Histech Pannoramic-250 microscope slide-scanner using a Zeiss 20x/0.80 Plan Apochromat objective. Snapshots of the scanned slides were taken using the Case Viewer software (3D-Histech) with individual magnification. Fluorescently labeled sections (TUNEL, PDGFRβ, CD31 immunofluorescence, CEBPβ nuclear localization, Rab11b localization, and cardiac markers in hiPSC-cardiomyocytes) were imaged using a Zeiss Axioimager D2 upright microscope with Zeiss LD Plan-Neofluar objectives (x20) and captured using a Coolsnap HQ2 camera (Photometrics) through Micromanager software v1.4.23. Specific band-pass filter sets for DAPI, FITC, and Texas Red were used to prevent bleeding from one channel to the next. Images were then further processed and analyzed using the ImageJ software (RRID:SCR_003070). Tube formation assays (Brightfield and fluorescent) were imaged on an Eclipse Ti inverted microscope (Nikon) using a 10× objective. Point visiting was used to allow multiple positions to be imaged within the same time-course, and cells were maintained at 37°C and 5% CO_2. The images were collected using a Retiga R6 (Q-Imaging) camera and analyzed using NIS-Elements AR v4.60.00 imaging software.
All representative images were selected based on their close correspondence to the mean/average quantified response observed for the respective experimental group and are representative of the overall findings across biological replicates.

Statistical Analysis

Data are presented as bar/dot plots showing mean ± standard error of the mean (SEM). Where sample sizes were ≥6, the Shapiro-Wilk test was conducted to determine whether the data were normally distributed. The non-parametric equivalent tests were utilized for skewed data and data sets where sample sizes were <6. Normally distributed data sets with three or more groups were analyzed using ordinary 1-way or 2-way ANOVA, followed by appropriate post-hoc tests, whereas comparisons between only 2 groups were performed using 2-tailed unpaired Student *t*-test. If the data were not normally distributed, non-parametric tests (equivalent to 1-way ANOVA and 2-tailed unpaired Student *t*-test), such as the Kruskal-Wallis test or the Mann-Whitney U test, were used. Regarding 2-way ANOVA test, permutation-based analyses were performed if N<6 in any group. Statistical analysis was performed using the GraphPad Prism 10 software (RRID:SCR_002798) or RStudio (R 4.6.21), and a P or q<0.05 was considered statistically significant.

Only the comparisons presented in the figures were statistically evaluated and, where applicable, adjusted for multiple comparisons and FDR. For Figures 1E and 3G, FDR correction was performed across a broader set of tested parameters than those presented in the figures, as detailed in the corresponding figure legends. In contrast, for Figures 8D-E, FDR correction was performed only across the parameters presented in the figures, as specified in the respective figure legends.

Multiple-comparison corrections were applied within individual experiments or datasets only, and no experiment-wide or across-test multiple-testing correction was performed. Sample sizes represent biological replicates and are detailed in the corresponding figure legends, together with information about the statistical tests applied following assessment of normality and the respective post hoc analyses performed.
