# Supplementary material for: FGFR1/KLB-MFG-E8 Maintains Microvascular Integrity and Adaptive Cardiac Remodeling
Source: Circ Res. 2026 Sep 29;139(10):e327950. doi: 10.1161/CIRCRESAHA.125.327950 (PMC13633393; doi:10.1161/CIRCRESAHA.125.327950)
Supplement: Supplementary file 3 [file res-139-e327950-s005.pdf]

| | Chow | | | | HFHSD+STZ | | | |
|---|---|---|---|---|---|---|---|---|
| **CYTOKINE** | | | | | | | | |
| **Activin A** | 159723 | 112252 | 160255 | 142015 | 74825 | 77470 | 85210 | 76954 |
| **b FGF** | 123490 | 157630 | 120365 | 148521 | 76807 | 84377 | 80014 | 70254 |
| **beta-Catenin** | 156586 | 168344 | 154026 | 159747 | 98248 | 97900 | 90247 | 100401 |
| **CCR3** | 116597 | 129123 | 109573 | 130147 | 63553 | 65383 | 70341 | 59486 |
| **CD40** | 186651 | 179684 | 165423 | 170419 | 103070 | 113601 | 110025 | 95687 |
| **Coagulation Factor III** | 229009 | 234778 | 212665 | 245987 | 87887 | 86996 | 96888 | 102422 |
| **CXCR2/IL-8 RB** | 146906 | 181499 | 175420 | 195633 | 52011 | 54042 | 72541 | 69854 |
| **CXCR4** | 144945 | 138630 | 154210 | 147326 | 98879 | 92493 | 100046 | 99975 |
| **Decorin** | 152672 | 233963 | 185742 | 210448 | 104806 | 109491 | 124105 | 110258 |
| **EG-VEGF/PK1** | 216750 | 213611 | 197304 | 208947 | 120817 | 125656 | 103654 | 132247 |
| **Endocan** | 206348 | 212121 | 199774 | 204774 | 113680 | 114808 | 134562 | 140408 |
| **Endoglin/CD105** | 217470 | 206512 | 224971 | 243157 | 97097 | 105370 | 125415 | 130089 |
| **Endostatin** | 236245 | 233620 | 235418 | 245179 | 86076 | 93737 | 112014 | 102405 |
| **Eotaxin** | 222812 | 225453 | 218996 | 204156 | 91817 | 95802 | 108752 | 123045 |
| **Eotaxin-2** | 232518 | 237624 | 215436 | 227890 | 102471 | 111369 | 124015 | 95688 |
| **Epigen** | 225671 | 226018 | 214026 | 238741 | 117932 | 126404 | 135791 | 124036 |
| **Epiregulin** | 216996 | 234699 | 220179 | 246170 | 108906 | 126503 | 140566 | 102888 |
| **Fas Ligand** | 138584 | 129915 | 140742 | 133665 | 44448 | 43687 | 59874 | 56423 |
| **Fractalkine** | 208299 | 223376 | 198324 | 240674 | 113816 | 118524 | 140211 | 129974 |
| **Gremlin** | 343150 | 290442 | 284101 | 295734 | 87515 | 82956 | 102410 | 95432 |
| **HGF** | 178296 | 173186 | 159624 | 210547 | 100614 | 93022 | 124056 | 99741 |
| **IL-1 alpha** | 120220 | 124129 | 134999 | 147254 | 170246 | 233140 | 201451 | 224415 |
| **IL-1 beta** | 104389 | 101036 | 98432 | 121447 | 177207 | 230902 | 186954 | 197264 |
| **IL-10** | 144829 | 149379 | 129743 | 143666 | 188224 | 186791 | 156425 | 154822 |
| **IL-12 p70** | 96909 | 87530 | 98712 | 99687 | 139031 | 124159 | 142561 | 119985 |
| **IL-18 R alpha/IL-1 R5** | 305273 | 310901 | 254126 | 267195 | 84965 | 84129 | 122114 | 134551 |
| **IL-6** | 218212 | 198909 | 221041 | 237411 | 153713 | 144020 | 123045 | 119487 |
| **Leptin R** | 151465 | 149615 | 150004 | 134261 | 198385 | 204214 | 183652 | 190623 |
| **MFG-E8** | 154842 | 158811 | 173418 | 186492 | 103965 | 103409 | 85863 | 99410 |
| **MMP-14/LEM-2** | 132711 | 116441 | 144558 | 149632 | 234273 | 225509 | 195420 | 202456 |
| **MMP-2** | 157796 | 177406 | 170264 | 188991 | 138634 | 129055 | 134026 | 145896 |
| **NOV/CCN3** | 80333 | 133640 | 102458 | 124715 | 70420 | 98014 | 80245 | 79634 |
| **PDGF R alpha** | 206108 | 190496 | 189324 | 221402 | 137903 | 127481 | 117298 | 143102 |
| **PDGF R beta** | 203287 | 190101 | 185364 | 200441 | 142535 | 120398 | 132456 | 148571 |
| **PF-4** | 192407 | 173871 | 182452 | 200147 | 131059 | 123382 | 140405 | 135763 |
| **Progranulin** | 157855 | 147336 | 166498 | 158291 | 108328 | 114594 | 108569 | 100255 |
| **Prolactin** | 145412 | 150205 | 169730 | 144772 | 99506 | 116598 | 105423 | 94652 |
| **Shh-N** | 112545 | 104437 | 142016 | 129874 | 203413 | 189547 | 179554 | 198972 |
| **SPARC** | 47485 | 43847 | 52140 | 56985 | 104582 | 108051 | 94265 | 96655 |
| **Thrombospondin** | 153197 | 154825 | 164782 | 170241 | 119777 | 122137 | 102545 | 111467 |
| **Tie-2** | 144236 | 143298 | 164220 | 148993 | 109926 | 114050 | 100411 | 97416 |
| **VEGF R1** | 128725 | 127056 | 124567 | 138724 | 93949 | 101046 | 88525 | 90044 |
| **VEGF R2** | 159607 | 163363 | 149776 | 170241 | 104549 | 104022 | 95412 | 89666 |
| **VEGF R3** | 143368 | 150263 | 160028 | 154226 | 96824 | 95134 | 85642 | 97412 |
| **VEGFB** | 123218 | 122836 | 132004 | 139705 | 94509 | 90093 | 112240 | 98723 |
| **VEGFD** | 130631 | 115963 | 129475 | 140266 | 85558 | 90250 | 94566 | 104263 |

**Supplemental Table 1. Raw chemiluminescent intensities of cytokines altered by HFHSD+STZ treatment.**

Raw chemiluminescent intensities of cytokines significantly altered after 12 weeks of high-fat, high-sucrose diet combined with streptozotocin (HFHSD+STZ) treatment. To ensure sufficient protein input for the assay, equal amounts of protein from two biological replicates were pooled to form a single experimental sample (8 hearts from both sexes were used in each group). Statistical significance was assessed using multiple unpaired t-tests with false discovery rate (FDR) correction using the two-stage step-up method of Benjamini, Krieger, and Yekutieli, with the FDR set at 1%. For each analysis, multiple-comparison correction was performed across all 57 tested parameters within the dataset. Parameters with $q<0.05$ vs. the control cohort are presented in the heatmap shown in Figure 1E.

| 8 weeks | Chow | | | | HFHSD+STZ | | | |
|---|---|---|---|---|---|---|---|---|
| | FGFR1$^{Flox}$ | | FGFR1$^{cKO}$ | | FGFR1$^{Flox}$ | | FGFR1$^{cKO}$ | |
| | Males | Females | Males | Females | Males | Females | Males | Females |
| **IVRT (ms)** | 8.40±0.24 | 9.60±0.24 | 9.00±0.31 | 9.2±0.66 | 11.17±0.47# | 11.33±0.42# | 14.67±0.55* | 14.14±0.55* |
| **E/A** | 1.49±0.15 | 1.43±0.06 | 1.31±0.04 | 1.48±0.04 | 1.39±0.06 | 1.64±0.12 | 1.86±0.14* | 1.73±0.11 |
| **sD (mm)** | 2.46±0.04 | 2.2±0.05 | 2.44±0.10 | 2.63±0.13 | 2.42±0.04 | 2.11±0.07 | 2.87±0.04* | 2.85±0.08* |
| **dD (mm)** | 4.25±0.03 | 3.77±0.06 | 4.04±0.04 | 4.01±0.12 | 4.07±0.08 | 3.83±0.05 | 4.05±0.05 | 3.91±0.07 |
| **sIVS (mm)** | 0.99±0.04 | 0.92±0.03 | 0.87±0.04 | 0.93±0.02 | 0.97±0.01 | 0.96±0.05 | 0.89±0.02 | 0.93±0.03 |
| **dIVS (mm)** | 0.70±0.01 | 0.71±0.02 | 0.66±0.05 | 0.72±0.03 | 0.78±0.01 | 0.74±0.06 | 0.73±0.01 | 0.69±0.02 |
| **sPW (mm)** | 0.90±0.02 | 0.85±0.03 | 0.86±0.02 | 0.87±0.03 | 1.05±0.02# | 0.93±0.04 | 0.89±0.02* | 0.77±0.02* |
| **dPW (mm)** | 0.62±0.01 | 0.57±0.01 | 0.58±0.01 | 0.61±0.01 | 0.76±0.01# | 0.64±0.04 | 0.63±0.01* | 0.61±0.01 |
| **EF (%)** | 80.45±1.17 | 79.70±1.66 | 77.76±2.51 | 71.71±1.82 | 78.76±1.59 | 82.83±1.84 | 64.22±0.70* | 60.87±1.96* |
| **FS (%)** | 42.05±1.15 | 41.42±1.66 | 39.72±2.10 | 34.48±1.43 | 40.53±1.52 | 44.77±1.96 | 29.02±0.45* | 26.98±1.22* |
| **HR (bpm)** | 485.2±9.65 | 449.6±9.56 | 432.2±13.36 | 494.2±24.29 | 486.5±10.22 | 447.3±14.4 | 456.4±13.85 | 469.9±9.33 |
| **HW/BW (mg/g)** | 4.83±0.43 | 5.18±0.33 | 5.20±0.30 | 4.70±0.12 | 5.56±0.25 | 4.97±0.20 | 5.38±0.44 | 5.13±0.46 |

**Supplemental Table 2. Echocardiographic assessment of FGFR1$^{cKO}$ mice after 8 weeks on chow or high-fat, high-sucrose diet (HFHSD) plus streptozotocin (STZ) in both sexes.**

Parameters include isovolumic relaxation time (IVRT), ratio of early (E-wave) to late (A-wave) ventricular filling velocities (E/A), left ventricular end-systolic diameter (sD), end-diastolic diameter (dD), end-systolic and end-diastolic interventricular septal thickness (sIVS, dIVS), end-systolic and end-diastolic posterior wall thickness (sPW, dPW), ejection fraction (EF%), fractional shortening (FS%), heart rate (HR), and heart weight/body weight (HW/BW). Data are shown as mean±SEM. (N=5 mice per sex/chow-fed group and N=6-7 mice per sex/HFHSD+STZ-fed group). Statistical significance was determined by permutation-based 2-way ANOVA test. #P<0.05 vs. FGFR1$^{Flox}$ chow-fed group; *P<0.05 vs. FGFR1$^{Flox}$ HFHSD+STZ mice of the same sex.

| | Chow | | | | HFHSD+STZ | | | |
|---|---|---|---|---|---|---|---|---|
| **8 weeks** | **KLB$^{Flox}$** | | **KLB$^{cKO}$** | | **KLB$^{Flox}$** | | **KLB$^{cKO}$** | |
| | **Males** | **Females** | **Males** | **Females** | **Males** | **Females** | **Males** | **Females** |
| **IVRT (ms)** | 7.60±0.24 | 8.20±0.40 | 7.80±0.37 | 9.40±0.40 | 10.50±0.56# | 9.33±0.33$^{p=0.06}$ | 14.67±0.61* | 16.67±1.43* |
| **E/A** | 1.41±0.09 | 1.73±0.02 | 1.46±0.07 | 1.43±0.06 | 1.41±0.07 | 1.48±0.03# | 1.76±0.07* | 1.73±0.08* |
| **sD (mm)** | 2.31±0.08 | 2.25±0.05 | 2.26±0.07 | 2.40±0.01 | 2.34±0.08 | 2.46±0.05# | 2.90±0.05* | 3.02±0.04* |
| **dD (mm)** | 3.97±0.12 | 3.73±0.09 | 3.88±0.07 | 3.94±0.02 | 3.91±0.05 | 3.90±0.06 | 4.18±0.10 | 4.25±0.05* |
| **sIVS (mm)** | 0.96±0.01 | 0.93±0.01 | 0.93±0.02 | 0.94±0.01 | 1.03±0.01 | 1.04±0.02 | 0.89±0.03* | 0.90±0.03* |
| **dIVS (mm)** | 0.70±0.01 | 0.71±0.02 | 0.71±0.01 | 0.70±0.01 | 0.76±0.01# | 0.74±0.02 | 0.69±0.01* | 0.70±0.01 |
| **sPW (mm)** | 0.90±0.04 | 0.84±0.02 | 0.85±0.04 | 0.94±0.02 | 1.06±0.04# | 0.97±0.01# | 0.95±0.01* | 0.92±0.01 |
| **dPW (mm)** | 0.61±0.02 | 0.59±0.01 | 0.57±0.01 | 0.58±0.00 | 0.73±0.01# | 0.73±0.01# | 0.64±0.01* | 0.63±0.00* |
| **EF (%)** | 80.25±0.46 | 77.78±1.68 | 80.15±0.86 | 77.33±0.24 | 78.37±1.56 | 74.88±1.12 | 66.19±1.10* | 63.78±2.77* |
| **FS (%)** | 41.78±0.45 | 39.58±1.50 | 41.71±0.86 | 39.03±0.22 | 40.15±1.46 | 36.97±0.92 | 30.38±0.76* | 28.95±1.79* |
| **HR (bpm)** | 460.2±11.46 | 454.4±4.50 | 447.2±10.59 | 442.2±12.49 | 473.0±10.02 | 447.5±16.91 | 425.2±12.96* | 438.5±8.79 |
| **HW/BW (mg/g)** | 4.84±0.18 | 4.83±0.17 | 4.62±0.12 | 5.05±0.06 | 4.33±0.06 | 5.29±0.20 | 4.82±0.29 | 5.43±0.25 |

**Supplemental Table 3. Echocardiographic assessment of KLB$^{cKO}$ mice after 8 weeks on chow or high-fat, high-sucrose diet (HFHSD) plus streptozotocin (STZ) in both sexes.**

Parameters include isovolumic relaxation time (IVRT), ratio of early (E-wave) to late (A-wave) ventricular filling velocities (E/A), left ventricular end-systolic diameter (sD), end-diastolic diameter (dD), end-systolic and end-diastolic interventricular septal thickness (sIVS, dIVS), end-systolic and end-diastolic posterior wall thickness (sPW, dPW), ejection fraction (EF%), fractional shortening (FS%), heart rate (HR), and heart weight/body weight (HW/BW). Data are shown as mean±SEM. (N=5 mice per sex/chow-fed group and N=6 mice per sex/HFHSD+STZ-fed group). Statistical significance was determined by permutation-based 2-way ANOVA test. #P<0.05 vs. KLB$^{Flox}$ chow-fed group; *P<0.05 vs. KLB$^{Flox}$ HFHSD+STZ mice of the same sex.

| | HFHSD+STZ | | | |
|---|---|---|---|---|
| | FGFR1$^{cKO}$ | | KLB$^{cKO}$ | |
| GENE | $Log_2$ Fold Change | $P_{adj.}$ | $Log_2$ Fold Change | $P_{adj.}$ |
| *Gm14165* | 3.356566309 | 2.2E-08 | -3.13488872 | 2.34872E-08 |
| *Pdlim3* | 1.82980039 | 2.26881E-08 | -2.26811519 | 1.3254E-13 |
| *Fut10* | -2.185463501 | 9.64888E-08 | 2.01091676 | 5.80674E-07 |
| *Gm9625* | 3.03661567 | 1.58152E-07 | -3.36023298 | 1.11962E-09 |
| *Gm8730* | 4.649075874 | 1.58152E-07 | -4.82151031 | 1.55773E-08 |
| *Zfp979* | -1.407280378 | 3.07115E-06 | 0.90530009 | 0.003481248 |
| *App* | -0.502520601 | 3.86629E-06 | 0.658717 | 1.9882E-11 |
| *Nr4a3* | 2.967708252 | 3.86629E-06 | -2.95057521 | 1.4835E-06 |
| *Clec16a* | 0.723690336 | 4.11145E-06 | -0.44699036 | 0.005215025 |
| *Gcn1* | 1.181020914 | 4.97725E-06 | -1.00472463 | 8.4527E-05 |
| *Gm46565* | -2.065947337 | 5.95131E-06 | 1.67470198 | 0.000294136 |
| *mt-Co1* | -0.742265656 | 1.70448E-05 | 0.36648612 | 0.042830323 |
| *Tnfsf10* | -0.96564003 | 2.27618E-05 | 1.33662095 | 2.02254E-11 |
| *Gck* | 1.154454289 | 0.000168145 | -1.47909487 | 3.41694E-08 |
| *Itpripl2* | -0.575474154 | 0.000242458 | 0.603234 | 2.03554E-05 |
| *Myh7b* | 0.913023719 | 0.000242638 | -1.15977792 | 1.02386E-07 |
| *Colec12* | -0.904358154 | 0.000242638 | 0.70759977 | 0.002468766 |
| *Rplp0-ps1* | 7.505950648 | 0.000242638 | -7.58545195 | 5.48128E-05 |
| *Dnajb5* | 0.636334612 | 0.00051348 | -0.76500822 | 1.64308E-06 |
| *Aspn* | -0.699840277 | 0.000536569 | 0.37516482 | 0.059746576 |
| *Gm7292* | -6.240201623 | 0.001093215 | 8.0342843 | 6.222E-07 |
| *Ets1* | -0.526296292 | 0.001093215 | 0.56765777 | 6.20158E-05 |
| *Gm6278* | 4.459113608 | 0.001093215 | -5.16584513 | 6.04957E-05 |
| *Flrt2* | -0.997703383 | 0.001093215 | 0.81922342 | 0.003582681 |
| *Prdm1* | -1.577162665 | 0.001103068 | 1.15641202 | 0.010654545 |
| *Aplnr* | -1.259303602 | 0.001483536 | 1.03691526 | 0.004093699 |
| *Myo18b* | 0.61501931 | 0.001548375 | -0.78979663 | 1.4835E-06 |
| *Rnf144a* | -0.578884 | 0.002709642 | 0.45072617 | 0.008990997 |
| *Cd34* | -0.711874657 | 0.002709642 | 0.58422369 | 0.005596083 |
| *Ahcyl* | -3.829315192 | 0.002724392 | 4.09889466 | 0.000205598 |
| *Reep3* | -0.533269794 | 0.002724392 | 0.93794819 | 1.12998E-11 |
| *Clec14a* | -0.754767692 | 0.002919064 | 0.52101892 | 0.023994082 |
| *mt-Nd4l* | -1.655839799 | 0.002919064 | 1.51771767 | 0.001956944 |
| *St8sia4* | -1.010986387 | 0.002919064 | 1.31443078 | 2.70404E-06 |
| *Arhgef3* | -0.528679441 | 0.003068504 | -1.17429996 | 0.035474142 |
| *Slc38a1* | 0.899033131 | 0.004027849 | 0.58926814 | 0.026396686 |
| *mt-Atp8* | -1.846408227 | 0.004154804 | 1.82452567 | 0.001035896 |
| *Myh7* | 2.355426487 | 0.004217743 | -1.27248783 | 0.099617052 |
| *Tmod3* | -0.470563041 | 0.004217743 | 0.338516 | 0.021450669 |
| *Gm3375* | 2.902923433 | 0.004391931 | -2.81599397 | 0.001270643 |
| *Usp28* | 0.379331635 | 0.004631031 | -0.45760843 | 4.52231E-05 |
| *Noct* | 1.00494933 | 0.00577854 | -1.0535182 | 0.000644894 |
| *Micall1* | 0.715212213 | 0.005840659 | -0.50028442 | 0.030155816 |
| *Hlf* | -1.240875451 | 0.005840659 | 1.0932553 | 0.005183391 |
| *Nrp2* | -0.559487609 | 0.006099861 | 0.7099318 | 2.33165E-05 |
| *Cd274* | -0.824801797 | 0.006650847 | 0.7376043 | 0.004929974 |
| *Wwtr1* | -0.397215565 | 0.007889221 | 0.32764978 | 0.010913437 |
| *Cyp2j9* | -0.859389742 | 0.008764184 | 0.62478393 | 0.030306962 |

| | HFHSD+STZ | | | |
|---|---|---|---|---|
| | FGFR1$^{cKO}$ | | KLB$^{cKO}$ | |
| GENE | $Log_2$ Fold Change | $P_{adj.}$ | $Log_2$ Fold Change | $P_{adj.}$ |
| *Meox2* | -0.807561558 | 0.008882236 | 0.91332623 | 0.00036052 |
| *Ide* | 0.650821107 | 0.009473403 | -0.62690479 | 0.003050902 |
| *Uchl1* | 1.031726647 | 0.009473403 | -1.09150265 | 0.000997801 |
| *6430548M08Rik* | 0.774370083 | 0.010686218 | -0.45747631 | 0.089384177 |
| *Nrep* | -0.8927134 | 0.010717166 | 1.03303049 | 0.000325056 |
| *Zfp568* | 0.88892002 | 0.010717166 | -0.56558998 | 0.065049385 |
| *Ptma* | -0.506900252 | 0.010717166 | 0.31856935 | 0.071935579 |
| *Olfml1* | -1.153414646 | 0.011782079 | 1.27449732 | 0.000636299 |
| *Ackr4* | -0.889925832 | 0.012956752 | 0.65485079 | 0.033797666 |
| *Vangl2* | -1.087963893 | 0.012975367 | 1.23130089 | 0.000460441 |
| *Ehbp1l1* | 0.459464678 | 0.013069648 | -0.43979115 | 0.004429512 |
| *C1qtnf7* | -0.798881645 | 0.013069648 | 0.88708568 | 0.000744837 |
| *Rgs5* | -0.368720043 | 0.013069648 | 0.55942115 | 1.20276E-06 |
| *Plau* | -1.060975915 | 0.013916249 | 1.01485606 | 0.004649118 |
| *Olfml3* | -1.060975915 | 0.013916249 | 0.6103346 | 0.006412256 |
| *Pcdh19* | -1.060975915 | 0.013916249 | 0.41224811 | 0.065242029 |
| *Myo18a* | -1.060975915 | 0.013916249 | -0.25021791 | 0.042516177 |
| *Casp12* | -1.060975915 | 0.013916249 | 0.44473108 | 0.071985657 |
| *Or2v2* | -1.060975915 | 0.013916249 | 1.53630848 | 0.001670356 |
| *Plat* | -0.764331575 | 0.01899093 | 0.84142298 | 0.001225321 |
| *Cxxc4* | -0.869930048 | 0.019213712 | 0.96392752 | 0.001119105 |
| *Adam10* | -0.393042854 | 0.019708328 | 0.44688125 | 0.000870177 |
| *Frmd5* | 0.897604028 | 0.020667507 | -0.7777884 | 0.014286763 |
| *Gbp5* | -0.945412012 | 0.020667507 | 0.96533283 | 0.003413473 |
| *Lurap1l* | -1.1455366 | 0.021108986 | 1.04974655 | 0.009618884 |
| *Rassf9* | -0.749899331 | 0.021277356 | 0.73312783 | 0.005463384 |
| *Tbc1d2b* | -0.427789949 | 0.021277356 | 0.48151886 | 0.001119105 |
| *Ifih1* | -0.701886096 | 0.021303515 | 0.69741231 | 0.00481993 |
| *Gbp2* | -0.912273236 | 0.022711451 | 0.69948646 | 0.036168192 |
| *Plxdc2* | -0.571076673 | 0.023189005 | 0.45187489 | 0.030199565 |
| *Cpd* | -0.372047941 | 0.02361828 | 0.57411336 | 3.23196E-06 |
| *Whrn* | 0.63790087 | 0.02361828 | -0.6412049 | 0.004824355 |
| *Enpp2* | -0.643154582 | 0.02361828 | 0.51458628 | 0.028913719 |
| *Ets2* | -0.535350739 | 0.02361828 | 0.52618711 | 0.005961324 |
| *Ywhab* | -0.378162984 | 0.024085655 | 0.35249648 | 0.009618884 |
| *Ociad2* | -1.535834724 | 0.024257793 | 1.20757577 | 0.033264978 |
| *mt-Co2* | -1.46893046 | 0.024257793 | 1.54783457 | 0.003132521 |
| *Sparc* | -0.628383316 | 0.024257793 | 0.6147777 | 0.006485832 |
| *Spred1* | -0.414724336 | 0.024825132 | 0.31217347 | 0.044089906 |
| *Ppp1r15a* | 0.686949293 | 0.024854862 | -0.72039948 | 0.003428351 |
| *Scd4* | 1.526146925 | 0.024967241 | 1.09028743 | 0.055092797 |
| *Foxred1* | 0.403030249 | 0.029401841 | -0.45724529 | 0.001673586 |
| *Cav2* | -0.524052012 | 0.029401841 | 0.69138639 | 0.000205715 |
| *Dock1* | -0.4416777 | 0.031426001 | 0.6643463 | 1.39124E-05 |
| *Cavin2* | -0.334632621 | 0.031426001 | 0.43475957 | 0.000297579 |
| *Gnb3* | 0.950735855 | 0.031426001 | -0.78571189 | 0.027058535 |
| *Ctbs* | -0.669005664 | 0.031426001 | 0.81843455 | 0.000601941 |
| *Pla2g2d* | 2.182357608 | 0.03161505 | 1.5544179 | 0.036462631 |
| *Pprc1* | -1.944620549 | 0.03161505 | -0.32452245 | 0.039162701 |
| *Dpt* | 0.415190759 | 0.032248836 | 0.33079762 | 0.052995498 |
| *Plac9* | -1.341095977 | 0.034859029 | 0.92802724 | 0.081293712 |
| *Anks1* | 0.445811983 | 0.034859029 | -0.59549031 | 0.000256021 |
| *P2rx4* | 0.449340575 | 0.034859029 | -0.50860864 | 0.002235556 |

| | HFHSD+STZ | | | |
|---|---|---|---|---|
| | FGFR1$^{cKO}$ | | KLB$^{cKO}$ | |
| **GENE** | **$Log_2$ Fold Change** | **$P_{adj.}$** | **$Log_2$ Fold Change** | **$P_{adj.}$** |
| *Tpst1* | -0.606469751 | 0.035003936 | 0.66333751 | 0.003323025 |
| *mt-Nd2* | -0.74261564 | 0.035457245 | 0.70744472 | 0.011874507 |
| *Slc7a1* | 0.629435774 | 0.035530952 | -0.53488764 | 0.026704251 |
| *Dcn* | -0.442297544 | 0.035530952 | 0.37976111 | 0.025452188 |
| *Tmem254* | -1.335396631 | 0.036437447 | 1.44169137 | 0.004119732 |
| *Ifit2* | -0.509383076 | 0.037677076 | 0.7548745 | 3.58322E-05 |
| *Col5a3* | 1.181514773 | 0.037839984 | -1.53855877 | 0.000436235 |
| *Itga2b* | 1.668231381 | 0.03789149 | -2.81311159 | 2.70404E-06 |
| *Cd302* | -0.779374592 | 0.039053204 | 0.90760113 | 0.001844032 |
| *Kdr* | -0.422814781 | 0.039053204 | 0.4349216 | 0.006888479 |
| *Fbrsl1* | 0.543057422 | 0.039053204 | -0.79002842 | 7.02486E-05 |
| *Misp3* | 1.322622209 | 0.040260671 | -1.22193712 | 0.015904692 |
| *Faap24* | -1.136338702 | 0.042416651 | 1.23718309 | 0.00419598 |
| *Sgce* | -0.463748733 | 0.044848804 | 0.40545163 | 0.026704251 |
| *Sdc3* | -0.520001052 | 0.045555156 | 0.41881466 | 0.044857004 |
| *Itih5* | -0.770158536 | 0.046011078 | 0.91122945 | 0.001976623 |
| *Fzd8* | -0.620254004 | 0.046011078 | 0.89376911 | 8.20954E-05 |
| *Afdn* | -0.408556979 | 0.046011078 | 0.36326318 | 0.024647147 |
| *Prkab1* | 0.378444741 | 0.04691985 | -0.46177912 | 0.001451961 |
| *Pcdhb22* | -0.812775649 | 0.04691985 | 0.72262083 | 0.025538095 |
| *mt-Co3* | -1.346702622 | 0.047091187 | 1.38211278 | 0.0087345 |
| *Ifit3b* | -0.997739128 | 0.047883841 | 0.89261186 | 0.024839261 |
| *Scn5a* | 0.470982855 | 0.048393089 | -0.68275329 | 0.000128751 |
| *Rhbdf2* | 0.677382061 | 0.048393089 | -0.94738866 | 0.000256796 |
| *Ptgfrn* | -0.472074175 | 0.051607368 | 0.56039492 | 0.002269841 |
| *Dach1* | -0.837374164 | 0.052932317 | 0.88733855 | 0.007388506 |
| *Chmp1b2* | -0.462777622 | 0.054786005 | 0.38070049 | 0.04614564 |
| *Enpp5* | -0.503893048 | 0.055906474 | 0.67097902 | 0.000600402 |
| *Gm48226* | -0.634597349 | 0.05647575 | 0.44764111 | 0.099534997 |
| *Rtp4* | -0.697920317 | 0.058718777 | 0.49778991 | 0.09562561 |
| *Sulf1* | -0.511542765 | 0.058925342 | 0.52781414 | 0.01035192 |
| *Slc38a2* | 0.465266445 | 0.059321815 | -0.5679549 | 0.002166408 |
| *Grk3* | 1.173593688 | 0.059321815 | -1.61344541 | 0.000469627 |
| *Vsir* | -0.56865306 | 0.059342007 | 0.48743617 | 0.039222055 |
| *Gnpda2* | -0.579814084 | 0.060811608 | 0.66742455 | 0.004108735 |
| *9930014A18Rik* | -1.142553035 | 0.062818414 | 0.88940956 | 0.069300307 |
| *Lrrfip1* | -2.008474673 | 0.062818414 | -0.96917601 | 0.000255242 |
| *Ifi206* | -1.975541888 | 0.06644083 | 1.92089769 | 0.018771596 |
| *Eprn* | 1.610086488 | 0.066860446 | 1.21966406 | 0.032722984 |
| *Zkscan5* | 0.518776651 | 0.066860446 | -0.47217885 | 0.028913719 |
| *Irs2* | 1.825835902 | 0.068864551 | -1.98459916 | 0.008271629 |
| *Gm17029* | 1.534265968 | 0.070150002 | -1.38195779 | 0.030838407 |
| *Tek* | -0.488166246 | 0.072322936 | 0.40000237 | 0.038777914 |
| *Tgfbr2* | -0.3120708 | 0.072724933 | 0.66930576 | 0.000663999 |
| *Bicc1* | -0.41419261 | 0.075823354 | 0.44356425 | 0.000382457 |
| *Selenop* | -1.429276552 | 0.077227197 | 0.55644228 | 0.000997801 |
| *Gm57446* | -1.429276552 | 0.077227197 | 1.09337479 | 0.088142681 |
| *Nck1* | -0.425989425 | 0.077227197 | 0.46773431 | 0.008406943 |
| *Rad21* | -0.328962553 | 0.080682869 | 0.51759108 | 8.4086E-05 |
| *Slc2a3* | 1.442509581 | 0.083258734 | -1.85796292 | 0.002175172 |
| *Pigu* | -0.453562815 | 0.083329535 | 0.47803145 | 0.012740044 |
| *Fv1* | 0.918961543 | 0.086787391 | 0.67309941 | 0.065993831 |
| *Ifitm3* | -0.500828742 | 0.087554145 | 0.38057308 | 0.091651022 |

| | HFHSD+STZ | | | |
|---|---|---|---|---|
| | FGFR1$^{cKO}$ | | KLB$^{cKO}$ | |
| **GENE** | **Log$_2$ Fold Change** | $P_{adj.}$ | **Log$_2$ Fold Change** | $P_{adj.}$ |
| ***Anxa1*** | -0.393961999 | 0.087946705 | 0.47236886 | 0.004440834 |
| ***Adam19*** | 0.661874193 | 0.092735532 | -0.64915126 | 0.024234317 |
| ***Igsf10*** | -0.733286305 | 0.092735532 | 0.87361434 | 0.004866977 |
| ***P2rx5*** | 0.662931494 | 0.092735532 | -0.73720211 | 0.009577911 |
| ***Pcbp4*** | 0.386898329 | 0.092735532 | -0.4017313 | 0.016302355 |
| ***Gm11407*** | -1.588044663 | 0.092735532 | 1.68802436 | 0.012580285 |
| ***Ifi208*** | -0.875236974 | 0.092735532 | 0.96296714 | 0.009916598 |
| ***Zbtb33*** | -0.675922347 | 0.093323762 | 1.17230989 | 1.18433E-05 |
| ***Nrbp2*** | 0.822692214 | 0.095094603 | -0.92448274 | 0.009061839 |
| ***Etaa1*** | -0.758124488 | 0.095367022 | 1.00529084 | 0.001334186 |
| ***Cd38*** | -0.559876777 | 0.096974537 | 0.50516134 | 0.043079338 |
| ***Arhgap33*** | 1.226806997 | 0.098486549 | -1.32731765 | 0.012855597 |
| ***Chmp5*** | -0.26849705 | 0.098825759 | 0.35667899 | 0.001699535 |

**Supplemental Table 4. RNA sequencing analyses of the gene expression profiles in FGFR1$^{cKO}$ and KLB$^{cKO}$ mice compared to controls under high-fat, high-sucrose diet (HFHSD) and streptozotocin (STZ).**

A total of 57,132 transcripts were detected. Differential expression analysis identified 299 significantly altered transcripts in FGFR1$^{cKO}$ mice and 3,316 in KLB$^{cKO}$ mice relative to controls. 167 transcripts presented in this table were commonly differentially expressed in both FGFR1$^{cKO}$ and KLB$^{cKO}$ mice. These shared transcripts, with adjusted *P* value ($P_{adj.}$) less than 0.1, were selected for pathway enrichment analyses presented in Figure 2J (N=4 mice from both sexes/group).

| 8 weeks | FGFR1$^{cKO}$ HFHSD+STZ | | | | | |
|---|---|---|---|---|---|---|
| | AAV9-*Gfp* | | AAV9-*Fgfr1* | | AAV9-*Klb* | |
| | Males | Females | Males | Females | Males | Females |
| IVRT (ms) | 14.83±0.40 | 15.83±0.94 | 11.33±0.21* | 11.83±0.30* | 16.33±0.84 | 18.67±1.54 |
| E/A | 1.95±0.06 | 1.83±0.17 | 1.38±0.07* | 1.48±0.05 | 1.89±0.06 | 2.15±0.18 |
| sD (mm) | 3.03±0.11 | 2.91±0.09 | 2.45±0.06* | 2.42±0.08 | 3.22±0.19 | 3.51±0.18 |
| dD (mm) | 4.21±0.09 | 3.99±0.08 | 4.08±0.03 | 4.09±0.10 | 4.36±0.11 | 4.39±0.14* |
| sIVS (mm) | 0.87±0.03 | 0.93±0.01 | 1.01±0.02* | 0.91±0.04 | 0.93±0.02 | 0.90±0.02 |
| dIVS (mm) | 0.71±0.01 | 0.69±0.02 | 0.74±0.03 | 0.71±0.01 | 0.70±0.01 | 0.70±0.02 |
| sPW (mm) | 0.88±0.02 | 0.81±0.01 | 0.97±0.01* | 0.96±0.01* | 0.91±0.02 | 0.84±0.03 |
| dPW (mm) | 0.64±0.00 | 0.60±0.00 | 0.71±0.02* | 0.72±0.01* | 0.62±0.01 | 0.60±0.01 |
| EF (%) | 62.32±2.73 | 61.18±1.97 | 78.02±1.42* | 78.80±2.34* | 59.22±4.87 | 48.63±3.34* |
| FS (%) | 27.96±1.63 | 27.15±1.20 | 39.70±1.30* | 40.76±2.14* | 26.33±2.62 | 20.11±1.76* |
| HR (bpm) | 463.5±11.79 | 440.8±10.81 | 421.8±8.13* | 437.0±11.97 | 450.7±9.55 | 450.2±14.61 |
| HW/BW (mg/g) | 5.51±0.22 | 5.89±0.27 | 5.18±0.17 | 5.28±0.26 | 5.52±0.16 | 5.97±0.14 |

**Supplemental Table 5. Echocardiographic assessment of FGFR1$^{cKO}$ mice under high-fat, high-sucrose diet (HFHSD) with streptozotocin (STZ) stress, followed by AAV9-*Gfp*, AAV9-*Fgfr1*, or AAV9-*Klb* treatment in both sexes.**

Parameters include isovolumic relaxation time (IVRT), ratio of early (E-wave) to late (A-wave) ventricular filling velocities (E/A), left ventricular end-systolic diameter (sD), end-diastolic diameter (dD), end-systolic and end-diastolic interventricular septal thickness (sIVS, dIVS), end-systolic and end-diastolic posterior wall thickness (sPW, dPW), ejection fraction (EF%), fractional shortening (FS%), heart rate (HR), and heart weight/body weight (HW/BW). Data are shown as mean±SEM. (N=6 mice per sex/group). Statistical significance was determined using the Kruskal-Wallis with Dunn's multiple comparison test (Males-IVRT, EF, FS) and ordinary 1-way ANOVA followed by Holm-Šidák multiple comparisons (other parameters). *P<0.05 vs. AAV9-*Gfp* mice of the same sex.

| | FGFR1$^{cKO}$ HFHSD+STZ | | | | | | | |
|---|---|---|---|---|---|---|---|---|
| | AAV9-*Gfp* | | | | AAV9-*Fgfr1* | | | |
| **CYTOKINE** | | | | | | | | |
| **Angiopoietin-like 2** | 4750 | 4520 | 3260 | 3890 | 7430 | 7510 | 8010 | 7630 |
| **CCR3** | 5270 | 4490 | 4410 | 4020 | 7590 | 7410 | 7620 | 8010 |
| **CD40** | 6410 | 6510 | 4560 | 5210 | 7310 | 7310 | 6960 | 7240 |
| **CXCR2/IL-8 RB** | 6160 | 3070 | 5420 | 4980 | 7160 | 5400 | 6660 | 7220 |
| **EG-VEGF/PK1** | 6320 | 5980 | 5260 | 6000 | 7290 | 7140 | 7310 | 7460 |
| **Endocan** | 6410 | 6440 | 5940 | 5540 | 7360 | 7330 | 7520 | 7620 |
| **Endoglin/CD105** | 6590 | 6450 | 5200 | 5410 | 7440 | 7540 | 7420 | 7330 |
| **Endostatin** | 6690 | 6580 | 5420 | 5980 | 7440 | 7530 | 7640 | 7290 |
| **Eotaxin-2** | 6060 | 5250 | 5120 | 4620 | 6780 | 6770 | 7000 | 6890 |
| **Epigen** | 5830 | 3160 | 4100 | 4250 | 7030 | 6940 | 6840 | 7040 |
| **Epiregulin** | 6130 | 4960 | 4590 | 5010 | 7250 | 6480 | 7140 | 6880 |
| **IL-1 alpha** | 5200 | 5010 | 4560 | 6030 | 6130 | 5820 | 6050 | 5810 |
| **IL-1 beta** | 5080 | 5130 | 5840 | 5970 | 6000 | 6370 | 6210 | 5990 |
| **IL-10** | 5900 | 5400 | 4580 | 4970 | 6730 | 6990 | 7410 | 7240 |
| **IL-12 p70** | 6800 | 6520 | 6660 | 6780 | 4020 | 4830 | 5050 | 4480 |
| **IL-6** | 6450 | 6450 | 5460 | 5970 | 7330 | 7400 | 7580 | 7790 |
| **Integrin beta 2/CD18** | 5360 | 5550 | 5200 | 5460 | 6780 | 7140 | 7040 | 6940 |
| **Leptin R** | 5700 | 5750 | 5690 | 5420 | 7070 | 7200 | 7540 | 7190 |
| **MFG-E8** | 5470 | 5200 | 4560 | 4720 | 6730 | 6840 | 7500 | 6970 |
| **MMP-14/LEM-2** | 5310 | 5230 | 6060 | 6620 | 7120 | 7220 | 7450 | 7110 |
| **MMP-2** | 6060 | 6020 | 5060 | 5140 | 7060 | 7120 | 7200 | 6850 |
| **Progranulin** | 5630 | 5950 | 5870 | 5720 | 6880 | 6910 | 6720 | 7110 |
| **Prolactin** | 5140 | 5380 | 5130 | 5260 | 7060 | 7360 | 7480 | 7510 |
| **Shh-N** | 5510 | 5710 | 5470 | 5320 | 7220 | 7630 | 7360 | 7440 |
| **TGF-beta RII** | 6020 | 6280 | 5820 | 5670 | 7130 | 7300 | 6740 | 6590 |
| **Thrombospondin** | 5560 | 5650 | 5040 | 4870 | 7640 | 7720 | 7810 | 7240 |
| **Tie-2** | 3770 | 4680 | 4890 | 4230 | 7680 | 7570 | 7460 | 7670 |
| **TLR3** | 6140 | 6170 | 6230 | 6540 | 7670 | 7650 | 8110 | 8080 |
| **TWEAK/TNFSF12** | 4290 | 5040 | 5550 | 5960 | 7870 | 7530 | 6850 | 6660 |
| **VCAM-1** | 6130 | 5560 | 5240 | 5010 | 7070 | 7010 | 8030 | 7760 |
| **VE-Cadherin** | 6000 | 6080 | 5530 | 5380 | 7150 | 7170 | 7540 | 7260 |
| **VEGF** | 5680 | 5590 | 5250 | 5160 | 7020 | 7480 | 7810 | 7690 |
| **VEGF R1** | 5630 | 5240 | 4850 | 4520 | 7470 | 7570 | 8240 | 7680 |
| **VEGF R2** | 4550 | 4790 | 4660 | 4470 | 7580 | 7530 | 7990 | 7650 |
| **VEGF R3** | 4420 | 4830 | 4360 | 4770 | 7410 | 7470 | 7550 | 7340 |
| **VEGFB** | 4950 | 5480 | 5040 | 4960 | 7090 | 7210 | 7150 | 7260 |
| **VEGFC** | 5610 | 6220 | 5240 | 5550 | 7510 | 7490 | 7270 | 7680 |
| **VEGFD** | 6430 | 6600 | 6230 | 5980 | 7560 | 7530 | 7480 | 7770 |

**Supplemental Table 6. Raw chemiluminescent intensities of cytokines altered by AAV9-*Fgfr1* treatment in HFHSD+STZ-stressed FGFR1$^{cKO}$ mice.**

Raw chemiluminescent intensities of cytokines significantly altered 4 weeks post AAV9-*Gfp* or AAV9-*Fgfr1* administration in FGFR1$^{cKO}$ mice subjected to high-fat, high-sucrose diet combined with streptozotocin (HFHSD+STZ). Data correspond to the heatmap shown in Figure 3G. To ensure sufficient protein input for the assay, equal amounts of protein from two biological replicates were pooled to form a single experimental sample (8 hearts from both sexes were used in each group). Statistical significance was assessed using multiple unpaired *t*-tests with false discovery rate (FDR) correction using the two-stage step-up method of Benjamini, Krieger, and Yekutieli, with the FDR set at 1%. For each analysis, multiple-comparison correction was performed across all 57 tested parameters within the dataset. Parameters with $q<0.05$ vs. the control cohort are presented in the heatmap shown in Figure 3G.

| 16 weeks | Chow | | HFHSD+STZ | | | | | | | |
|---|---|---|---|---|---|---|---|---|---|---|
| | AAV9-*Gfp* | | AAV9-*Gfp* | | AAV9-*Fgfr1* | | AAV9-*Klb* | | Dual OE | |
| | **Males** | **Females** | **Males** | **Females** | **Males** | **Females** | **Males** | **Females** | **Males** | **Females** |
| **IVRT (ms)** | 10.40±0.50 | 9.00±0.54 | 16.50±0.61# | 16.83±0.87# | 16.17±0.30 | 15.67±0.21 | 14.83±0.30 | 14.83±0.60 | 12.33±0.42* | 12.50±0.34* |
| **E/A** | 1.39±0.03 | 1.59±0.09 | 1.83±0.04# | 1.81±0.05 | 1.78±0.06 | 1.82±0.06 | 1.75±0.05 | 1.78±0.07 | 1.36±0.04* | 1.42±0.02* |
| **sD (mm)** | 2.48±0.01 | 2.15±0.09 | 3.18±0.05# | 2.86±0.06# | 3.07±0.04 | 2.89±0.03 | 2.91±0.06 | 2.80±0.03 | 2.52±0.04* | 2.46±0.05* |
| **dD (mm)** | 4.07±0.01 | 3.72±0.08 | 4.53±0.06# | 4.27±0.07# | 4.26±0.03 | 4.22±0.03 | 4.16±0.04* | 4.16±0.02 | 4.15±0.03* | 4.17±0.03 |
| **sIVS (mm)** | 0.98±0.01 | 0.89±0.03 | 1.04±0.01 | 1.03±0.02# | 0.94±0.01* | 0.94±0.02 | 0.97±0.02 | 0.96±0.02 | 0.96±0.01* | 0.92±0.01* |
| **dIVS (mm)** | 0.68±0.01 | 0.67±0.02 | 0.77±0.01# | 0.77±0.01# | 0.69±0.00* | 0.71±0.00 | 0.72±0.00 | 0.71±0.00 | 0.74±0.01 | 0.72±0.01 |
| **sPW (mm)** | 0.92±0.01 | 0.87±0.02 | 1.11±0.04# | 1.01±0.01# | 0.87±0.02* | 0.93±0.01 | 1.00±0.02 | 0.97±0.01 | 0.94±0.01* | 0.91±0.02* |
| **dPW (mm)** | 0.60±0.00 | 0.62±0.01 | 0.73±0.00# | 0.72±0.02# | 0.67±0.00 | 0.68±0.00 | 0.70±0.00 | 0.69±0.00 | 0.65±0.01* | 0.67±0.01 |
| **EF (%)** | 77.42±0.34 | 80.57±1.59 | 65.26±1.09# | 69.86±1.66# | 62.57±1.52 | 67.62±1.17 | 65.73±1.40 | 69.43±1.10 | 77.64±0.62* | 79.45±0.97* |
| **FS (%)** | 39.11±0.31 | 42.25±1.58 | 29.74±0.74# | 33.02±1.23# | 28.00±0.95 | 31.38±0.83 | 30.09±0.96 | 32.68±0.79 | 39.33±0.58* | 41.07±0.97* |
| **HR (bpm)** | 477.2±10.9 | 467.0±6.59 | 423.3±8.90# | 461.3±8.44 | 455.2±9.49 | 449.5±5.47 | 457.0±6.77 | 448.8±5.47 | 456.5±4.14 | 452.7±3.93 |
| **HW/BW (mg/g)** | 5.36±0.34 | 4.92±0.13 | 4.86±0.11 | 5.46±0.24 | 4.68±0.07 | 5.35±0.13 | 4.45±0.22 | 4.93±0.26 | 4.48±0.18 | 5.14±0.27 |

**Supplemental Table 7. Echocardiographic assessment of wild-type mice under high-fat, high-sucrose diet (HFHSD) with streptozotocin (STZ) stress for 12 weeks, followed by AAV9-*Gfp*, AAV9-*Fgfr1*, AAV9-*Klb* or dual overexpression for 4 more weeks in both sexes.**

Parameters include isovolumic relaxation time (IVRT), ratio of early (E-wave) to late (A-wave) ventricular filling velocities (E/A), left ventricular end-systolic diameter (sD), end-diastolic diameter (dD), end-systolic and end-diastolic interventricular septal thickness (sIVS, dIVS), end-systolic and end-diastolic posterior wall thickness (sPW, dPW), ejection fraction (EF%), fractional shortening (FS%), heart rate (HR), and heart weight/body weight (HW/BW). Data are shown as mean±SEM. (N=5 mice per sex/chow-fed group and N=6 mice per sex/HFHSD+STZ-fed group). Statistical significance was determined using the Kruskal-Wallis with Dunn's multiple comparison test. #P<0.05 vs. chow-fed group; *P<0.05 vs. AAV9-*Gfp* treated HFHSD+STZ mice of the same sex.

| 8h | Control | PA+HG | | |
|---|---|---|---|---|
| | | si*Negative* | si*Fgfr1* | si*Klb* |
| Covered area (%) | 16.60±1.59 | 17.78±1.56 | 10.13±0.59* | 10.98±0.47* |
| Total tubes | 130.2±34.27 | 169.7±15.53 | 65.50±19.02* | 73.33±18.32* |
| Total loops | 21.83±6.79 | 26.50±2.14 | 7.667±4.23* | 9.50±4.70 *p*=0.056 |
| Total nets | 2.67±0.61 | 3.33±0.33 | 2.833±0.40 | 3.167±0.60 |
| Total branching points | 67.33±18.82 | 80.17±6.11 | 31.50±10.15* | 35.17±10.19 *p*=0.050 |

| 16h | Control | PA+HG | | | |
|---|---|---|---|---|---|
| | | Adv-*LacZ* | Adv-*Fgfr1* | Adv-*Klb* | Dual OE |
| Covered area (%) | 19.65±0.88 | 7.50±0.98# | 7.15±1.05 | 7.35±0.95 | 17.18±0.74* |
| Total tubes | 161.3±13.71 | 47.17±12.50# | 48.83±7.77 | 45.00±8.76 | 51.67±7.52 |
| Total loops | 28.50±2.88 | 0.50±0.34# | 0.66±0.34 | 1.00±0.36 | 3.833±0.90* |
| Total nets | 4.50±0.88 | 6.0±2.79 | 6.83±1.40 | 6.83±1.77 | 2.833±0.47 |
| Total branching points | 69.33±8.64 | 15.67±4.00# | 15.17±2.12 | 16.00±2.44 | 24.17±2.96 |

**Supplemental Table 8. Angiogenic capacity of human umbilical vein endothelial cells (HUVECs) cultured in conditioned media from neonatal rat cardiomyocytes (NRCMs) with FGFR1 and KLB knockdown (top) or overexpression (bottom), following 8 or 16 hours of diabetic-like stress induced by palmitic acid (PA) and high glucose (HG) *in vitro*.**

Parameters assessed include total tube-covered area, total tubes, total loops, total nets, and total branching points. Data are shown as mean±SEM. (N=6 independent biological replicates/group). Statistical significance was determined using ordinary 1-way ANOVA with Šidák multiple comparisons. *P<0.05 vs. si*Negative* or Adv-*LacZ* cohort. #P<0.05 vs. control cohort.

| 8 weeks | HFHSD+STZ | | | |
|---|---|---|---|---|
| | FGFR1$^{cKO}$ | | FGFR1$^{cKO}$ + rMFG-E8 | |
| | Males | Females | Males | Females |
| IVRT (ms) | 15.83±0.30 | 14.67±0.33 | 11.00±0.36* | 12.67±0.66* |
| E/A | 1.78±0.03 | 1.69±0.06 | 1.33±0.05* | 1.52±0.07 |
| sD (mm) | 2.74±0.02 | 2.66±0.03 | 2.38±0.04* | 2.26±0.06* |
| dD (mm) | 4.09±0.02 | 4.03±0.04 | 3.87±0.09 | 3.83±0.06* |
| sIVS (mm) | 0.95±0.01 | 0.99±0.01 | 1.01±0.03 | 0.96±0.03 |
| dIVS (mm) | 0.70±0.01 | 0.70±0.01 | 0.76±0.01* | 0.74±0.01 |
| sPW (mm) | 0.95±0.01 | 0.98±0.01 | 0.86±0.02* | 0.86±0.04* |
| dPW (mm) | 0.70±0.01 | 0.70±0.01 | 0.64±0.01* | 0.64±0.01* |
| EF (%) | 69.82±0.48 | 71.08±0.34 | 76.74±0.85* | 78.96±0.93* |
| FS (%) | 32.93±0.36 | 33.88±0.26 | 38.54±0.73* | 40.59±0.89* |
| HR (bpm) | 436.8±4.90 | 424.2±6.78 | 454.7±16.68 | 461.8±11.53* |
| HW/BW (mg/g) | 5.06±0.09 | 5.87±0.31 | 5.39±0.37 | 5.16±0.20 |

**Supplemental Table 9. Echocardiographic assessment of FGFR1$^{cKO}$ mice treated with recombinant MFG-E8 (rMFG-E8) in both sexes under stress.**

Parameters include isovolumic relaxation time (IVRT), ratio of early (E-wave) to late (A-wave) ventricular filling velocities (E/A), left ventricular end-systolic diameter (sD), end-diastolic diameter (dD), end-systolic and end-diastolic interventricular septal thickness (sIVS, dIVS), end-systolic and end-diastolic posterior wall thickness (sPW, dPW), ejection fraction (EF%), fractional shortening (FS%), heart rate (HR), and heart weight/body weight (HW/BW). Data are shown as mean±SEM. (N=6 mice per sex/group). Statistical significance was determined using the Mann-Whitney U test (Males-E/A) or 2-tailed unpaired Student *t*-test (other parameters). *P<0.05 vs. FGFR1$^{cKO}$ untreated cohort of the same sex.

| 8 weeks | HFHSD+STZ | | | |
|---|---|---|---|---|
| | KLB$^{cKO}$ | | KLB$^{cKO}$ + rMFG-E8 | |
| | Males | Females | Males | Females |
| IVRT (ms) | 16.00±0.36 | 16.83±1.27 | 11.83±0.30* | 13.00±0.36* |
| E/A | 1.85±0.04 | 1.84±0.07 | 1.51±0.08* | 1.46±0.02* |
| sD (mm) | 2.88±0.02 | 3.03±0.04 | 2.38±0.04* | 2.43±0.04* |
| dD (mm) | 4.27±0.02 | 4.41±0.05 | 4.00±0.03* | 4.00±0.04* |
| sIVS (mm) | 0.97±0.01 | 0.94±0.01 | 1.01±0.02 | 0.99±0.01* |
| dIVS (mm) | 0.74±0.01 | 0.71±0.00 | 0.72±0.01 | 0.72±0.01 |
| sPW (mm) | 0.95±0.01 | 0.92±0.01 | 1.02±0.02* | 1.01±0.02* |
| dPW (mm) | 0.66±0.00 | 0.64±0.00 | 0.69±0.01* | 0.68±0.01* |
| EF (%) | 69.28±0.80 | 67.52±0.62 | 78.31±0.79* | 77.45±0.43* |
| FS (%) | 32.55±0.58 | 31.28±0.43 | 39.96±0.74* | 39.15±0.39* |
| HR (bpm) | 445.5±5.98 | 423.8±7.39 | 467.3±7.85 | 444.8±9.28 |
| HW/BW (mg/g) | 5.52±0.11 | 5.74±0.11 | 5.09±0.07* | 5.45±0.11 |

**Supplemental Table 10. Echocardiographic assessment of KLB$^{cKO}$ mice treated with recombinant MFG-E8 (rMFG-E8) in both sexes under stress.**

Parameters include isovolumic relaxation time (IVRT), ratio of early (E-wave) to late (A-wave) ventricular filling velocities (E/A), left ventricular end-systolic diameter (sD), end-diastolic diameter (dD), end-systolic and end-diastolic interventricular septal thickness (sIVS, dIVS), end-systolic and end-diastolic posterior wall thickness (sPW, dPW), ejection fraction (EF%), fractional shortening (FS%), heart rate (HR), and heart weight/body weight (HW/BW). Data are shown as mean±SEM. (N=6 mice per sex/group). Statistical significance was determined using 2-tailed unpaired Student *t*-test. *P<0.05 vs. KLB$^{cKO}$ untreated mice of the same sex.

| 8h | si*Fgfr1* - PA+HG | |
|---|---|---|
| | cDNA | CEBPβ OE |
| Covered area (%) | 10.10±0.915 | 19.73±1.265* |
| Total tubes | 84.50±23.20 | 183.8±14.50* |
| Total loops | 11.50±5.485 | 33.75±3.66* |
| Total nets | 3.25±0.25 | 4.50±0.2887 |
| Total branching points | 41.25±12.70 | 94.50±7.643* |

| 8h | si*Klb* - PA+HG | |
|---|---|---|
| | cDNA | CEBPβ OE |
| Covered area (%) | 11.60±0.2677 | 25.58±2.609* |
| Total tubes | 87.00±25.53 | 160.0±3.873* |
| Total loops | 12.25±6.897 | 32.25±2.428* |
| Total nets | 3.50±0.866 | 4.0±0.9129 |
| Total branching points | 42.75±14.21 | 85.0±2.345* |

**Supplemental Table 11. Angiogenic capacity of HUVECs cultured in conditioned media from FGFR1- (top) and KLB-knockdown (bottom) neonatal rat cardiomyocytes (NRCMs) with or without CEBPβ overexpression, followed by 8 hours of palmitic acid (PA) and high glucose (HG) stress *in vitro*.**

Parameters assessed include total tube-covered area, total tubes, total loops, total nets, and total branching points. Data are shown as mean±SEM. (N=4 independent biological replicates/group). Statistical significance was determined using the Mann-Whitney U test. *P<0.05 vs. cDNA cohort.

| 8 weeks | HFHSD+STZ | | | |
|---|---|---|---|---|
| | FGFR1$^{cKO}$ | | FGFR1$^{cKO}$ + AAV9-*CEBPB* | |
| | Males | Females | Males | Females |
| IVRT (ms) | 16.50±0.42 | 15.50±1.02 | 12.00±0.25* | 12.67±1.11 |
| E/A | 1.93±0.08 | 1.88±0.14 | 1.48±0.04* | 1.51±0.01* |
| sD (mm) | 3.23±0.08 | 2.91±0.08 | 2.47±0.03* | 2.47±0.02* |
| dD (mm) | 4.37±0.05 | 4.02±0.08 | 4.03±0.03* | 4.00±0.01 |
| sIVS (mm) | 0.93±0.04 | 0.94±0.01 | 0.98±0.00 | 0.92±0.01 |
| dIVS (mm) | 0.71±0.01 | 0.73±0.01 | 0.72±0.01 | 0.71±0.00 |
| sPW (mm) | 0.95±0.03 | 0.85±0.02 | 0.96±0.00 | 0.93±0.00* |
| dPW (mm) | 0.66±0.01 | 0.61±0.01 | 0.69±0.01* | 0.67±0.01* |
| EF (%) | 59.16±2.64 | 61.87±2.17 | 76.95±0.69* | 76.25±0.59* |
| FS (%) | 25.97±1.57 | 27.61±1.34 | 38.72±0.62* | 38.09±0.51* |
| HR (bpm) | 450.0±11.39 | 438.3±9.85 | 460.3±9.47 | 448.2±5.56 |
| HW/BW (mg/g) | 5.05±0.24 | 5.70±0.29 | 5.07±0.57 | 5.51±0.52 |

**Supplemental Table 12. Echocardiographic assessment of AAV9-*CEBPB* treatment in FGFR1$^{cKO}$ mice after diabetic stress induced by high-fat, high-sucrose diet (HFHSD) with streptozotocin (STZ).**

Parameters include isovolumic relaxation time (IVRT), ratio of early (E-wave) to late (A-wave) ventricular filling velocities (E/A), left ventricular end-systolic diameter (sD), end-diastolic diameter (dD), end-systolic and end-diastolic interventricular septal thickness (sIVS, dIVS), end-systolic and end-diastolic posterior wall thickness (sPW, dPW), ejection fraction (EF%), fractional shortening (FS%), heart rate (HR), and heart weight/body weight (HW/BW). Data are shown as mean±SEM. (N=6 mice per sex/group). Statistical significance was determined using the Mann-Whitney U test (Males-sIVS, sPW, dPW; Females-dD) or 2-tailed unpaired Student *t*-test (other parameters) *P<0.05 vs. FGFR1$^{cKO}$ untreated cohort of the same sex.

| 8 weeks | HFHSD+STZ | | | |
|---|---|---|---|---|
| | KLB$^{cKO}$ | | KLB$^{cKO}$ + AAV9-*CEBPB* | |
| | Males | Females | Males | Females |
| **IVRT (ms)** | 16.33±0.42 | 16.50±1.38 | 12.33±0.42* | 14.00±0.36 |
| **E/A** | 1.84±0.04 | 1.81±0.08 | 1.46±0.03* | 1.39±0.03* |
| **sD (mm)** | 2.98±0.05 | 3.02±0.04 | 2.51±0.03* | 2.49±0.03* |
| **dD (mm)** | 4.31±0.03 | 4.39±0.05 | 4.06±0.01* | 4.01±0.00* |
| **sIVS (mm)** | 0.96±0.01 | 0.95±0.01 | 0.97±0.01 | 0.97±0.02 |
| **dIVS (mm)** | 0.72±0.02 | 0.73±0.01 | 0.81±0.02* | 0.78±0.01* |
| **sPW (mm)** | 0.94±0.01 | 0.91±0.01 | 0.97±0.01 | 0.96±0.01* |
| **dPW (mm)** | 0.65±0.00 | 0.63±0.00 | 0.64±0.01 | 0.64±0.01 |
| **EF (%)** | 66.67±1.53 | 67.61±0.73 | 76.27±0.64* | 76.11±0.84* |
| **FS (%)** | 30.74±1.04 | 31.34±0.51 | 38.11±0.55* | 38.00±0.74* |
| **HR (bpm)** | 448.7±4.97 | 429.8±7.49 | 476.0±11.02* | 472.8±5.44* |
| **HW/BW (mg/g)** | 5.42±0.15 | 5.68±0.13 | 5.28±0.18 | 5.41±0.21 |

**Supplemental Table 13. Echocardiographic assessment of AAV9-*CEBPB* treatment in KLB$^{cKO}$ mice after diabetic stress induced by high-fat, high-sucrose diet (HFHSD) with streptozotocin (STZ).**

Parameters include isovolumic relaxation time (IVRT), ratio of early (E-wave) to late (A-wave) ventricular filling velocities (E/A), left ventricular end-systolic diameter (sD), end-diastolic diameter (dD), end-systolic and end-diastolic interventricular septal thickness (sIVS, dIVS), end-systolic and end-diastolic posterior wall thickness (sPW, dPW), ejection fraction (EF%), fractional shortening (FS%), heart rate (HR), and heart weight/body weight (HW/BW). Data are shown as mean±SEM. (N=6 mice per sex/group). Statistical significance was determined using the Mann-Whitney U test (Females-sD) or 2-tailed unpaired Student *t*-test (other parameters). *$P<0.05$ vs. KLB$^{cKO}$ untreated cohort of the same sex.

| CYTOKINES | Dual OE | | | | | | Dual OE + MFG-E8 Ab | | | | | |
|---|---|---|---|---|---|---|---|---|---|---|---|---|
| IL-6 | 33.8607 | 224.4063 | 256.8983 | 435.7229 | 323.7519 | 10.7903 | 5.1517 | 7.5358 | 4.8238 | 4.3941 | 9.0937 | 6.5143 |
| Angiopoietin-1 | 47.15 | 26.5 | 29.85 | 24.27 | 23.7 | 22.47 | 25.94 | 22.27 | 19.49 | 21.08 | 22.59 | 18.85 |
| Angiopoietin-2 | 412.7273 | 696.6659 | 604.3596 | 553.5122 | 599.8094 | 207.9415 | 172.14 | 176.464 | 111.0192 | 151.9002 | 141.894 | 120.7038 |
| EGF | 33.9403 | 29.7444 | 19.0974 | 13.256 | 14.0389 | 17.351 | 14.0456 | 16.7282 | 9.5786 | 8.8027 | 20.3717 | 14.2498 |
| FGF-basic | 2443.61 | 44994.46 | 50704.37 | 2409.48 | 2235.17 | 3191.29 | 5068.23 | 4764.54 | 3802.73 | 2345.22 | 5673.45 | 5429.62 |
| CXCL8 (IL-8) | 93.6414 | 782.4039 | 576.9456 | 496.5654 | 522.2336 | 317.837 | 20.9294 | 13.9695 | 11.8346 | 12.2145 | 20.516 | 15.6103 |
| PECAM-1 | 1519.447 | 1234.859 | 2910.303 | 1656.64 | 1327.189 | 1755.208 | 59.819 | 80.152 | 97.549 | 67.519 | 404.005 | 97.696 |
| PIGF | 35.88 | 67.87 | 70.96 | 21.97 | 19.29 | 24.17 | 12.7 | 10.05 | 10 | 10.37 | 11.75 | 11.14 |
| VEGF | 0.6718 | 1.4409 | 0.7893 | 0.4046 | 1.1963 | 0.9337 | 0.2589 | 0.3836 | 0.2988 | 0.3742 | 0.4285 | 0.4907 |
| TNF-α | 0.627 | 0.6282 | 0.5004 | 0.6946 | 0.4767 | 0.4625 | 0.3874 | 0.4362 | 0.4167 | 0.4138 | 0.4711 | 0.4105 |

| CYTOKINES | CEBPβ OE | | | | | | CEBPβ OE + MFG-E8 Ab | | | | | |
|---|---|---|---|---|---|---|---|---|---|---|---|---|
| IL-6 | 6.5257 | 25.9418 | 143.3333 | 32.3944 | 34.5298 | 172.6596 | 4.0723 | 12.9849 | 3.4559 | 3.683 | 3.5911 | 3.4821 |
| Angiopoietin-1 | 17.84 | 34.89 | 30.55 | 17.62 | 18.2 | 17.86 | 17.97 | 24.49 | 18.6 | 32.22 | 17.36 | 19.96 |
| Angiopoietin-2 | 432.027 | 791.7074 | 462.6581 | 136.5799 | 593.3482 | 997.8395 | 173.9065 | 338.3163 | 155.1491 | 170.5473 | 168.0128 | 171.4991 |
| EGF | 44.2814 | 49.3652 | 26.6237 | 36.3425 | 29.3177 | 32.1857 | 16.3169 | 12.8949 | 13.466 | 14.6522 | 14.3249 | 14.5446 |
| FGF-basic | 5708.46 | 14728.21 | 7307.99 | 7867.48 | 5943.55 | 5259.43 | 2801.78 | 13490.45 | 3810.27 | 2876.03 | 2749.05 | 2231.52 |
| CXCL8 (IL-8) | 145.6178 | 70.7363 | 185.9244 | 66.8154 | 67.4793 | 229.675 | 18.0499 | 16.629 | 16.7667 | 8.1208 | 9.4201 | 13.019 |
| PECAM-1 | 6116.438 | 3916.858 | 248.655 | 533.728 | 850.644 | 1113.45 | 209.839 | 74.833 | 165.79 | 191.831 | 27.004 | 185.719 |
| PIGF | 11.91 | 16.71 | 24.8 | 17.75 | 11.96 | 11.13 | 12.74 | 16.44 | 11.8 | 12.17 | 11.89 | 12.51 |
| VEGF | 1.2352 | 1.6971 | 1.674 | 1.3656 | 0.6298 | 0.5716 | 0.4442 | 0.2466 | 0.4203 | 0.4872 | 0.3901 | 0.2704 |
| TNF-α | 0.5199 | 0.4796 | 0.4097 | 0.5084 | 0.36 | 0.5162 | 0.9593 | 0.7707 | 0.878 | 0.7849 | 0.4231 | 0.8799 |

**Supplemental Table 14. Angiogenic cytokine expression in HUVECs cultured with conditioned media from hiPSC-cardiomyocytes with FGFR1+KLB overexpression (Dual OE) or CEBPβ overexpression in the presence or absence of MFG-E8 antibody**

Raw concentrations (pg/mL) of angiogenic cytokines measured using the LEGENDplex™ Human Angiogenesis Panel 1 V02. Human umbilical vein endothelial cells (HUVECs) were cultured in conditioned media collected from human induced pluripotent stem cell-derived cardiomyocytes (hiPSC-cardiomyocytes) subjected to dual FGFR1 and KLB overexpression (Dual OE; top panel) or CEBPβ overexpression (bottom panel), in the presence or absence of an anti-MFG-E8 neutralizing antibody (MFG-E8 Ab; 1 µg/mL). N=6 biological replicates per group. Statistical significance was assessed using multiple Mann-Whitney tests with false discovery rate (FDR) correction using the two-stage step-up method of Benjamini, Krieger, and Yekutieli, with the FDR set at 1%. For each analysis, multiple-comparison correction was performed across all 10 tested parameters within the dataset. Parameters with $q<0.05$ vs. the control cohort are presented in the heatmap shown in Figure 8D (Dual OE) and Figure 8E (CEBPβ OE) and are marked with *.

| 8h | si*Klb* - PA+HG | |
|---|---|---|
| | cDNA | CEBPβ OE |
| Covered area (%) | 14.45±1.513 | 27.08±1.282* |
| Total tubes | 137.0±12.99 | 200.0±18.85* |
| Total loops | 23.0±2.972 | 38.75±4.171* |
| Total nets | 3.5±0.866 | 4.75±0.75 |
| Total branching points | 71.25±7.341 | 99.50±14.51 |

**Supplemental Table 15. Angiogenic capacity of HUVECs cultured in conditioned media from KLB-knockdown human induced pluripotent stem cell-derived cardiomyocytes (hiPSC-cardiomyocytes) with CEBPβ overexpression after 8 hours of diabetes-like stress induced by palmitic acid (PA) and high glucose (HG) *in vitro*.**

Parameters assessed include total tube-covered area, total tubes, total loops, total nets, and total branching points. Data are shown as mean±SEM. (N=4 independent biological replicates/group). Statistical significance was determined using the Mann-Whitney U test. *P<0.05 vs. cDNA cohort.

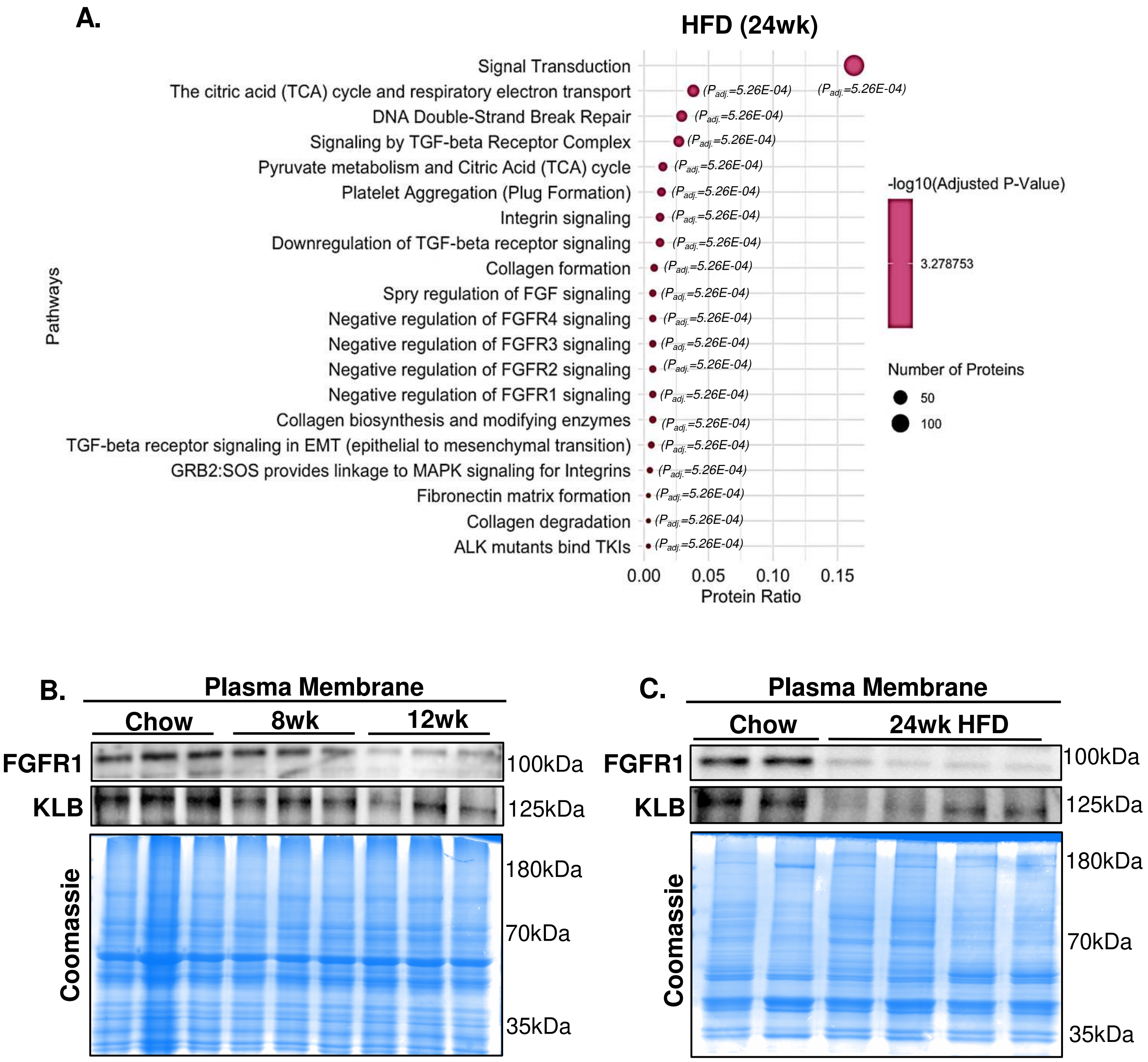


**Supplemental Figure 1. Cardiac expression of FGFR1 and KLB on plasma membranes are diminished in metabolically stressed mouse hearts.**

**A,** Gene Ontology pathway enrichment analyses of the differentially expressed proteins ($P_{adj.}$<0.1) identified by liquid chromatography-mass spectrometry in mouse hearts following 24 weeks of high-fat diet (HFD) feeding versus chow-fed mice (N=2 experimental samples/group; 3 individual hearts pooled as one experimental sample). Enriched pathways ($P_{adj.}$<0.05) are presented in the figure. **B,** Representative immunoblots showing cardiac plasma membrane fractions of FGFR1 and KLB expression reduced after 12 weeks of high-fat, high-sucrose diet (HFHSD) and streptozotocin (STZ) treatment (HFHSD+STZ). Coomassie blue staining was used to assess total protein as a loading control. **C,** Representative immunoblots showing reduced cardiac plasma membrane expression of FGFR1 and KLB expression diminished in mouse hearts fed with a HFD for 24 weeks. Coomassie blue staining was used to assess total protein as a loading control.

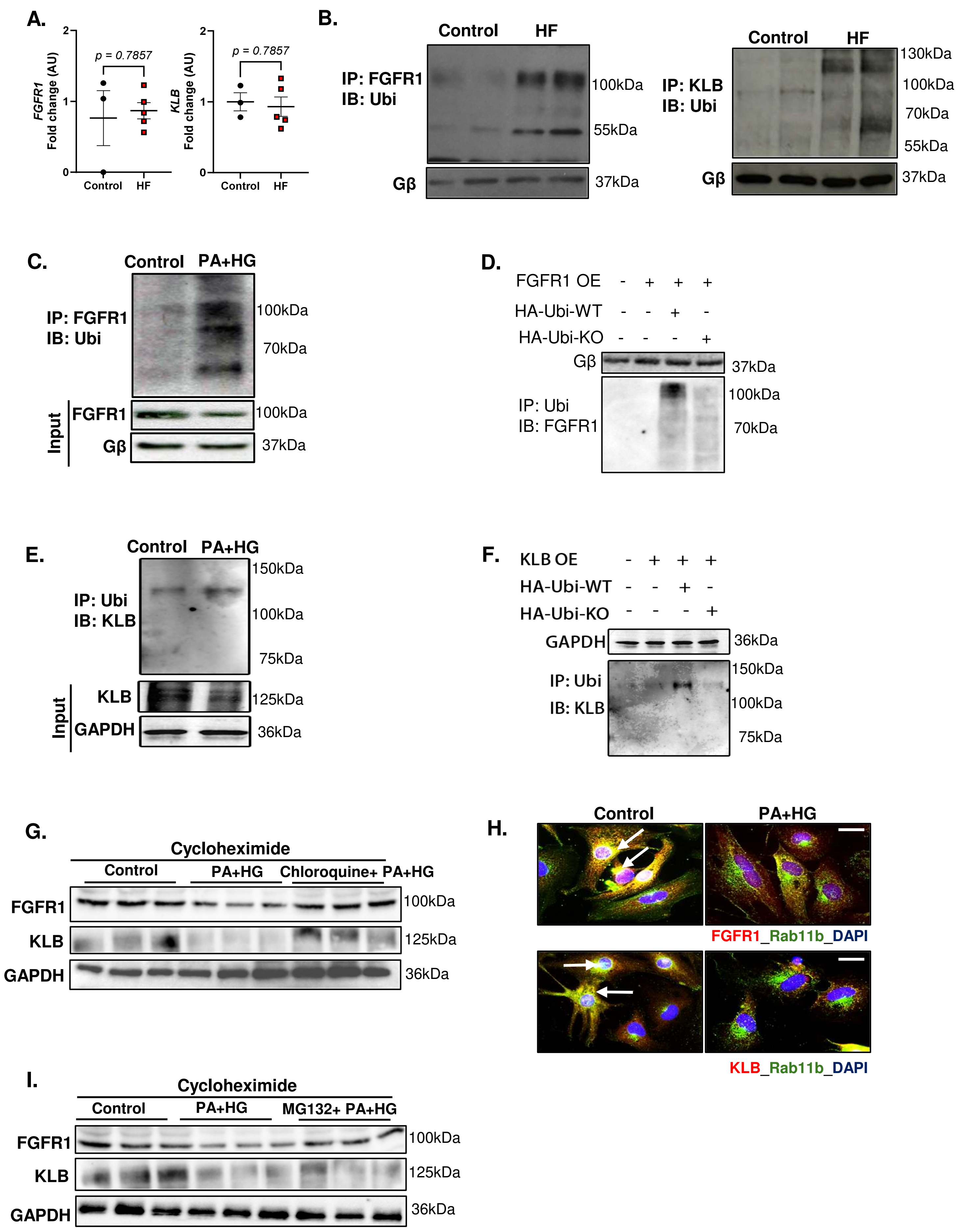
A.
FGFR1 Fold change (AU)
p = 0.7857
Control
HF
KLB Fold change (AU)
p = 0.7857
B.
Control HF
IP: FGFR1
IB: Ubi
100kDa
55kDa
Gβ
37kDa
IP: KLB
IB: Ubi
130kDa
100kDa
70kDa
55kDa
C.
Control PA+HG
IP: FGFR1
IB: Ubi
70kDa
Input
FGFR1
D.
FGFR1 OE - + + +
HA-Ubi-WT - - + -
HA-Ubi-KO - - - +
IP: Ubi
IB: FGFR1
E.
IP: Ubi
IB: KLB
150kDa
75kDa
KLB
125kDa
GAPDH
36kDa
F.
KLB OE - + + +
HA-Ubi-WT - - + -
HA-Ubi-KO - - - +
G.
Cycloheximide
Control
PA+HG
Chloroquine+ PA+HG
H.
FGFR1_Rab11b_DAPI
KLB_Rab11b_DAPI
I.
MG132+ PA+HG

**Supplemental Figure 2**. **Prolonged diabetic stress promotes ubiquitination-related degradation of FGFR1 and KLB.**

**A,** Quantitative PCR analysis of *FGFR1* and *KLB* in human diabetic failing hearts (N=3 hearts/control group and N=5 hearts/HF group). **B,** Immunoprecipitation demonstrating increased ubiquitination of FGFR1 and KLB in human HF hearts. **C,** Immunoprecipitation demonstrating increased ubiquitination of FGFR1 in H9C2 cells following 16-hour exposure to palmitic acid (PA; 500 μmol/L and high glucose (HG, 25 mmol/L) stress (PA+HG). **D,** Immunoprecipitation demonstrating enhanced ubiquitination of FGFR1 in H9C2 cells co-transfected with HA-tagged wild-type ubiquitin (HA-Ubi-WT) during overexpression of FGFR1. The effect was abolished by mutant ubiquitin (HA-Ubi-KO, all lysines mutated to arginines). **E,** Immunoprecipitation demonstrating increased ubiquitination of KLB after prolonged PA+HG stress in H9C2 cells. **F**, Immunoprecipitation demonstrating enhanced ubiquitination of KLB in H9C2 cells co-transfected with HA-Ubi-WT during overexpression of KLB. The effect was abolished by HA-Ubi-KO. **G,** Representative immunoblot showing FGFR1 and KLB expression in neonatal rat cardiomyocytes treated with cycloheximide (10 μg/mL) under control or PA+HG conditions for 16 hours, with or without lysosomal inhibitor, chloroquine (50 μmol/L). GAPDH served as a loading control. **H,** Representative immunofluorescence images showing subcellular localization of FGFR1 (red, top panel) and KLB (red; bottom panel) with Rab11b (green) after 16 hours of PA+HG treatment. Nuclei were counterstained with DAPI (blue). White arrows indicate regions of co-localization (scale=20 μm). **I,** Representative immunoblot showing FGFR1 and KLB expression in neonatal rat cardiomyocytes treated with cycloheximide (10 μg/mL) under control or PA+HG conditions for 16 hours, with or without proteasomal inhibitor, MG132 (10 μmol/L). GAPDH served as a loading control. Data represent mean±SEM. Statistical significance values (indicated in each panel) were calculated versus corresponding controls using the Mann-Whitney U test (**A)**.

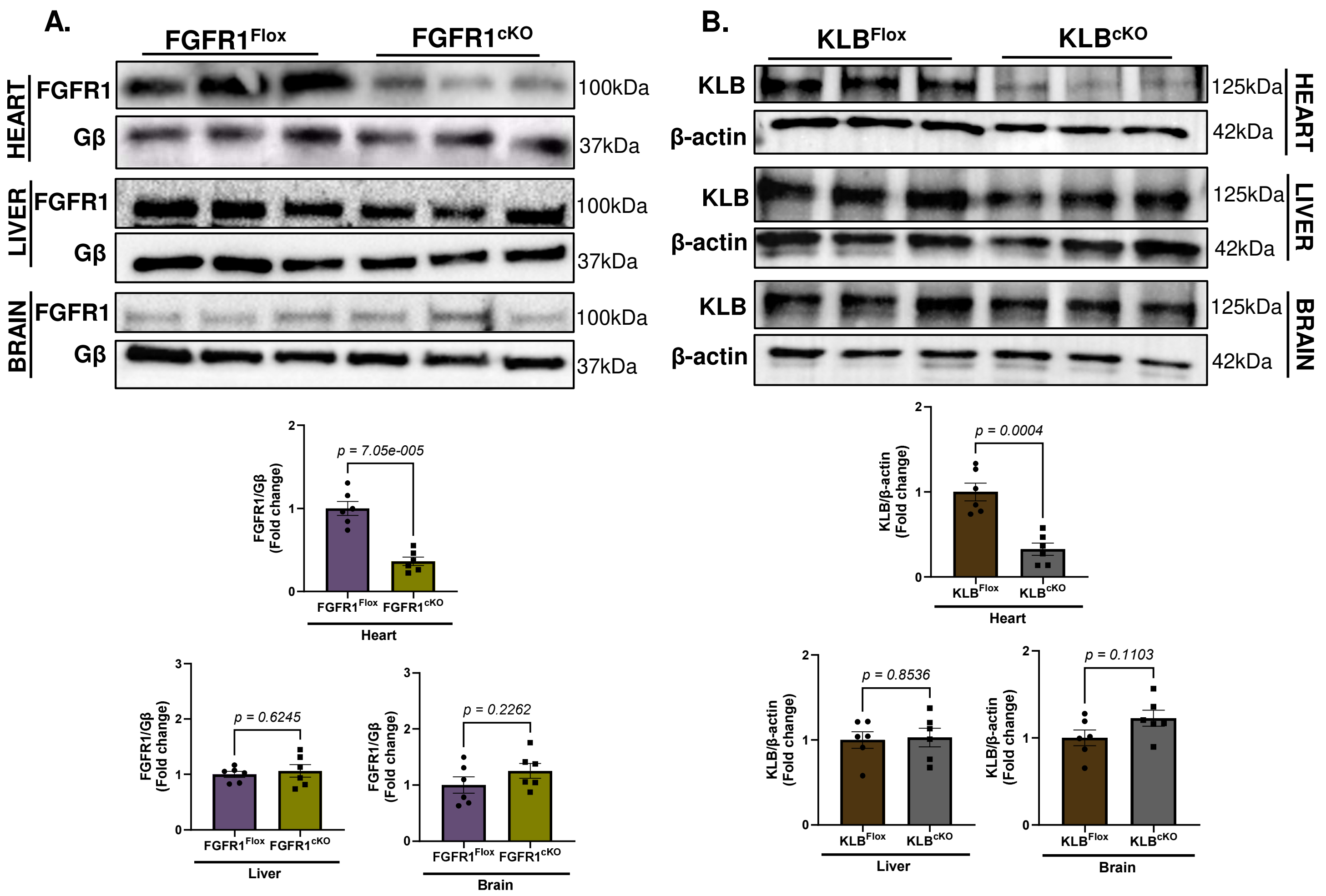


**Supplemental Figure 3. Characterization of cardiomyocyte-specific FGFR1 (FGFR1$^{cKO}$) and KLB (KLB$^{cKO}$) knockout mouse models.**

**A,** Representative immunoblots and quantification graphs showing FGFR1 expression in heart, liver, and brain lysates from FGFR1$^{Flox}$ and FGFR1$^{cKO}$ mice. Gβ was used as the loading control (N=6 mice from both sexes/group). **B,** Representative immunoblots and quantification graphs showing KLB expression in heart, liver, and brain lysates from KLB$^{Flox}$ and KLB$^{cKO}$ mice (N=6 mice from both sexes/group). β-actin was used as the loading control. Data are presented as mean±SEM. Statistical comparisons (shown in the panel) were made using 2-tailed unpaired Student *t*-test (**A-B)**.

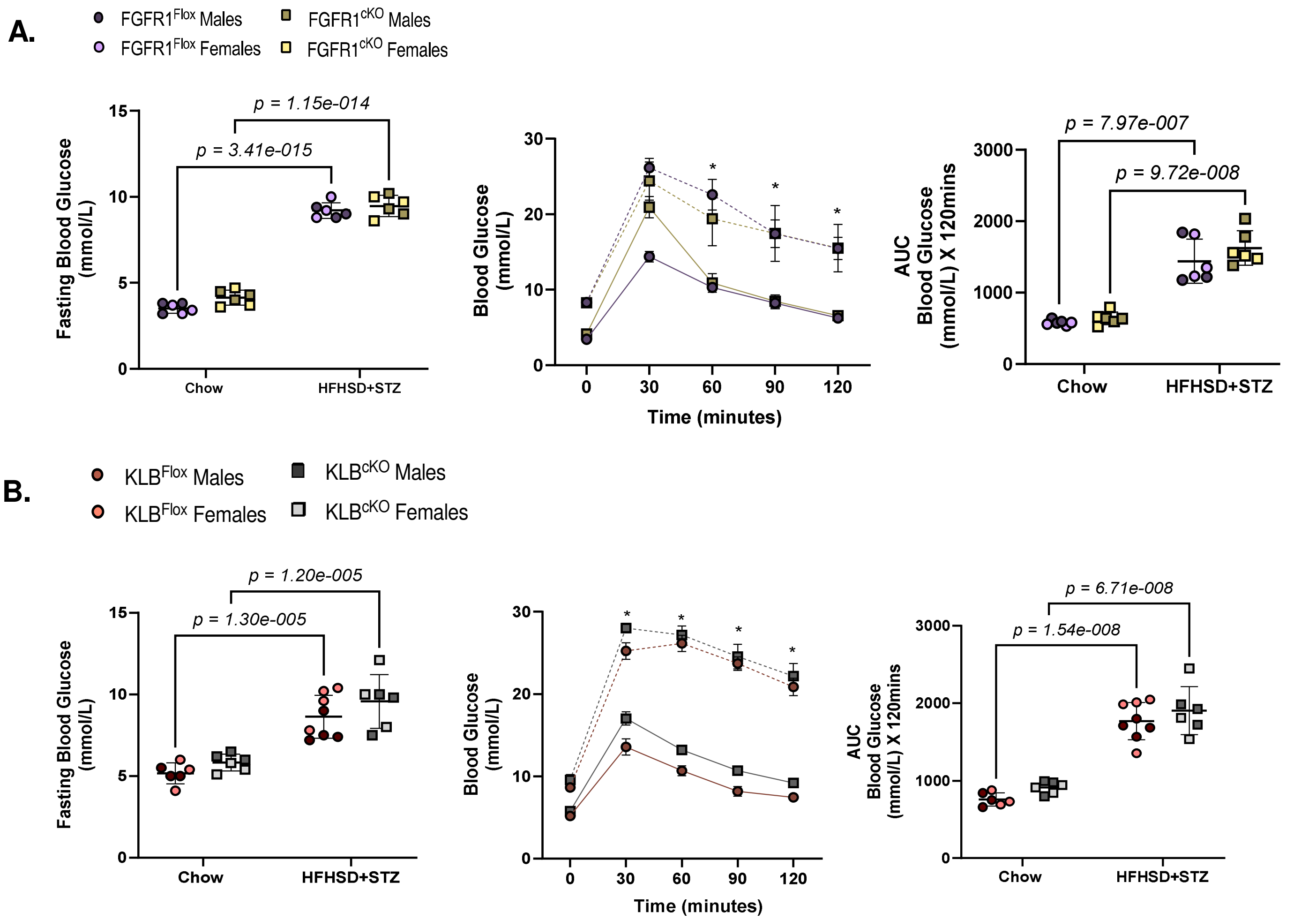


**Supplemental Figure 4**. **HFHSD+STZ-induced systemic metabolic alterations.**

**A,** Data graphs showing fasting blood glucose (left) and a glucose tolerance test (middle) performed after 8 weeks on chow or high-fat, high-sucrose diet (HFHSD) with streptozotocin (STZ) in FGFR1$^{Flox}$ and FGFR1$^{cKO}$ mice. Glucose was measured at 0, 30, 60, 90, and 120 min post injection of glucose; area under the curve (AUC) is shown in the right (N=6 mice from both sexes/group). **B,** Data graphs showing fasting blood glucose (left) and an intraperitoneal glucose tolerance test (middle) performed after 8 weeks on chow or HFHSD+STZ in KLB$^{Flox}$ and KLB$^{cKO}$ mice. Glucose was measured at 0, 30, 60, 90, and 120 min post injection of glucose; AUC is shown in the right (N=6 mice from both sexes/chow-fed group and N=6-8 mice from both sexes/HFHSD+STZ-fed group). *P<0.05 vs to Chow group regardless of genotypes. Data are presented as mean±SEM. Statistical comparisons (indicated in the panel versus respective chow groups) were performed using ordinary 2-way ANOVA followed by Šidák multiple comparisons (**A**).

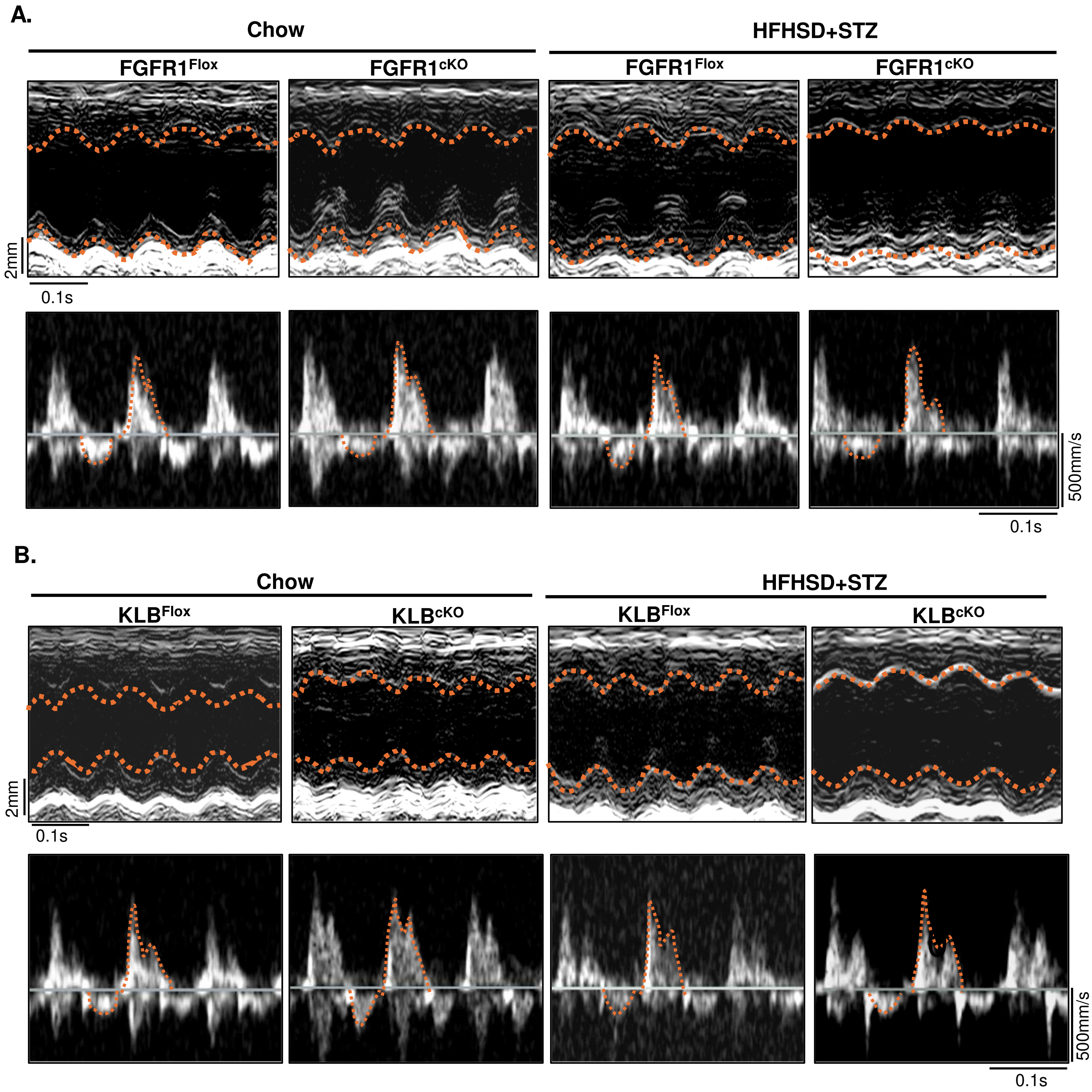


**Supplemental Figure 5. Representative echocardiographic images obtained from mice.**

**A,** Representative echocardiographic M-mode images (top, short-axis view) and pulsed-wave Doppler tracings (bottom) from FGFR1$^{Flox}$ and FGFR1$^{cKO}$ mice fed either chow diet or subjected to high-fat, high-sucrose diet (HFHSD) combined with streptozotocin (STZ) treatment (HFHSD+STZ) for 8 weeks. **B,** Representative echocardiographic M-mode images (top, short-axis view) and pulsed-wave Doppler tracings (bottom) from KLB$^{Flox}$ and KLB$^{cKO}$ mice fed either chow diet or subjected to HFHSD+STZ treatment for 8 weeks.

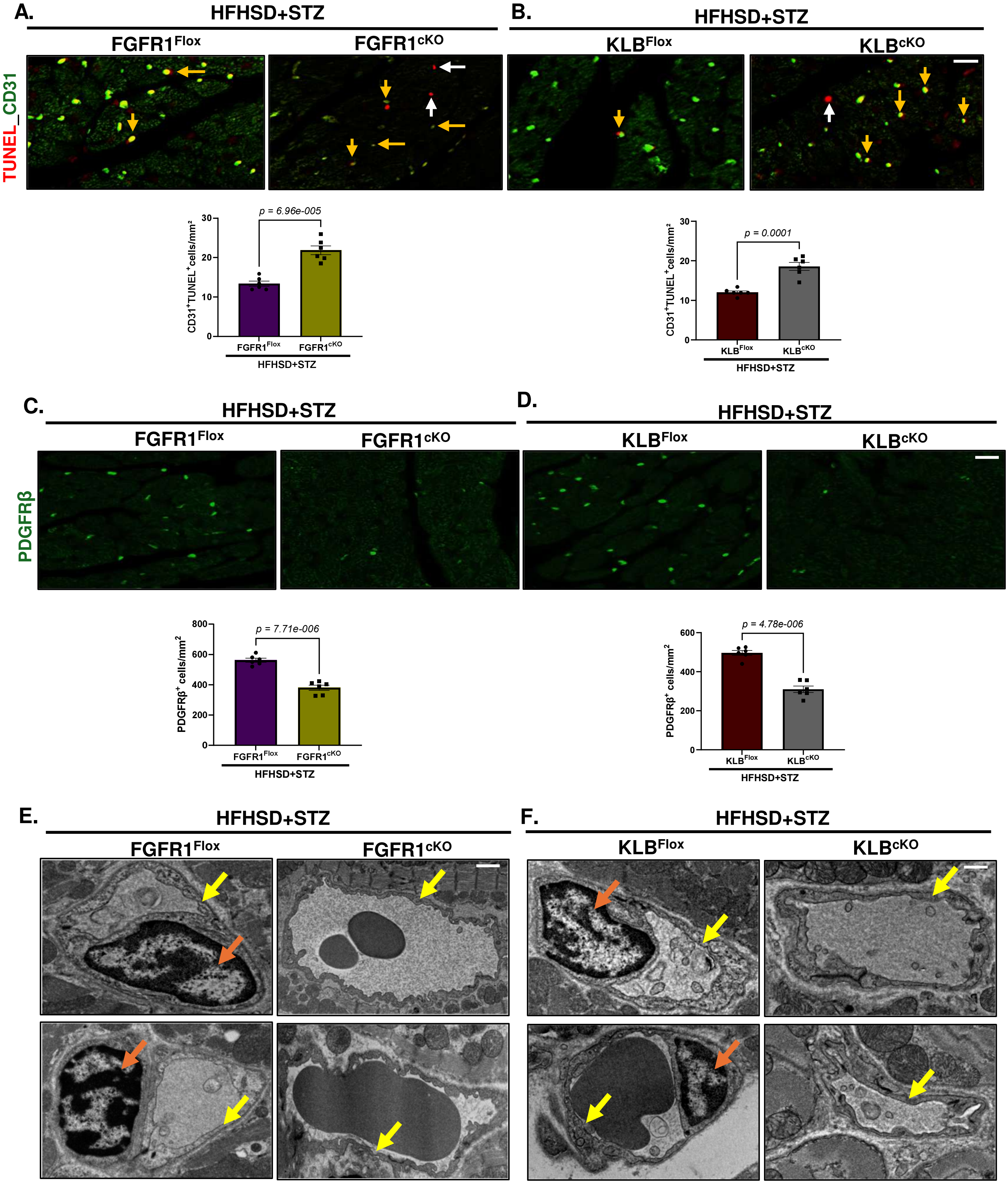
A.
HFHSD+STZ
FGFR1Flox
FGFR1cKO
TUNEL_CD31
B.
HFHSD+STZ
KLBFlox
KLBcKO
p = 6.96e-005
CD31+TUNEL+cells/mm²
FGFR1Flox
FGFR1cKO
HFHSD+STZ
p = 0.0001
CD31+TUNEL+cells/mm²
KLBFlox
KLBcKO
HFHSD+STZ
C.
HFHSD+STZ
FGFR1Flox
FGFR1cKO
PDGFRβ
D.
HFHSD+STZ
KLBFlox
KLBcKO
p = 7.71e-006
PDGFRβ+ cells/mm²
FGFR1Flox
FGFR1cKO
HFHSD+STZ
p = 4.78e-006
PDGFRβ+ cells/mm²
KLBFlox
KLBcKO
HFHSD+STZ
E.
HFHSD+STZ
FGFR1Flox
FGFR1cKO
F.
HFHSD+STZ
KLBFlox
KLBcKO

G.

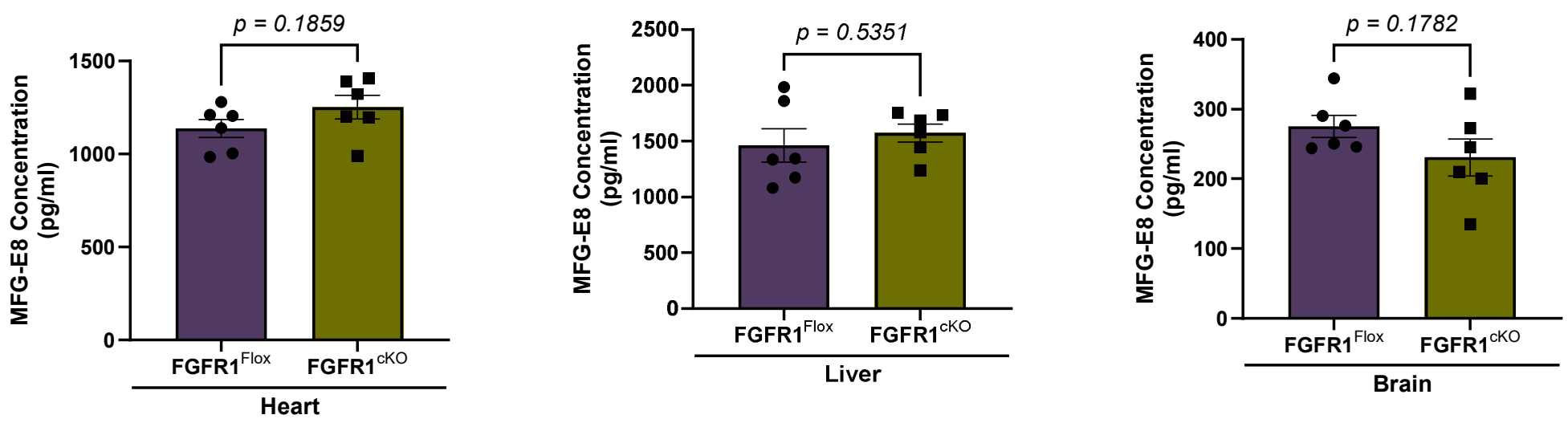


H.

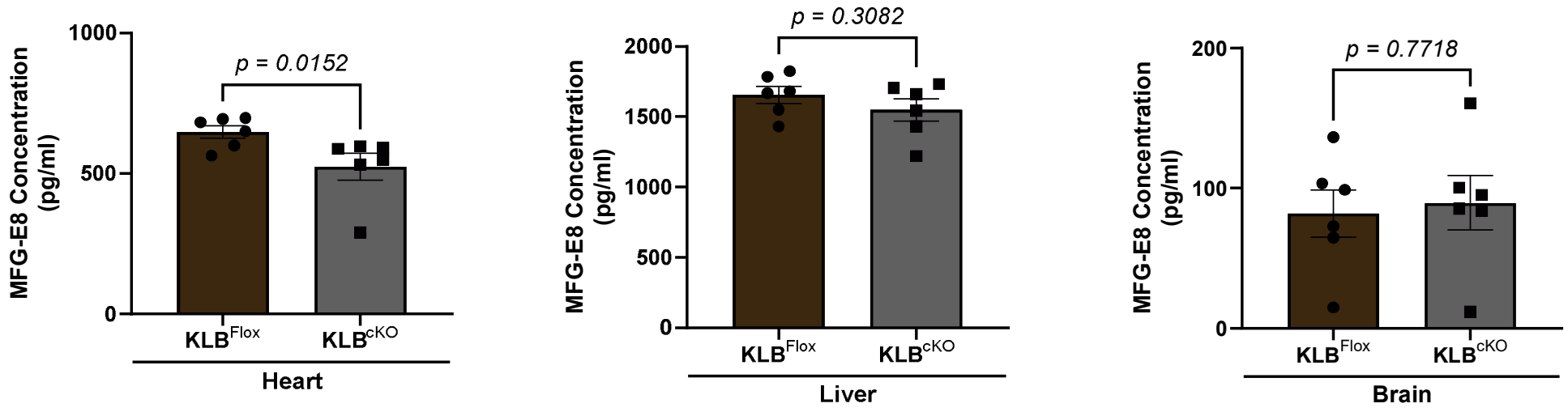


**Supplemental Figure 6. Cardiomyocyte-specific FGFR1 and KLB knockout exacerbates endothelial apoptosis and pericyte loss in diabetes.**

**A-B,** Representative TUNEL-stained heart sections showing TUNEL-positive nuclei (red) and respective quantification graphs in high-fat, high-sucrose diet combined with streptozotocin (HFHSD+STZ)-treated (**A**) FGFR1$^{cKO}$ and (**B**) KLB$^{cKO}$ mice versus respective floxed controls. CD31 (green) marks endothelial cells. Yellow arrow indicates apoptotic endothelial cells while white arrows indicate apoptotic cardiomyocytes (N=6 hearts from both sexes/group; scale=20 µm). **C-D,** Representative PDGFRβ immunostaining (green) and respective quantification of pericyte density in (**C**) FGFR1$^{cKO}$ and (**D**) KLB$^{cKO}$ mice following HFHSD+STZ stress (N=6 hearts from both sexes/group; scale=20 µm). **E-F,** Representative transmission electron microscopy images from (**E**) FGFR1$^{cKO}$ and (**F**) KLB$^{cKO}$ hearts under HFHSD+STZ stress. Yellow arrows denote endothelial cells, and orange arrows denote pericytes (scale=1 µm). **G-H,** MFG-E8 ELISA in the heart, liver, and brain tissues from (**G**) FGFR1$^{cKO}$ and (**H**) KLB$^{cKO}$ mice under basal chow diet (N=6 mice from both sexes/group). Data represent mean±SEM. Statistical significance values (indicated in each panel) were calculated versus corresponding controls using 2-tailed unpaired Student *t*-test (**A**-**D, G, H middle and right panel**) and the Mann-Whitney U test (**H left panel**).

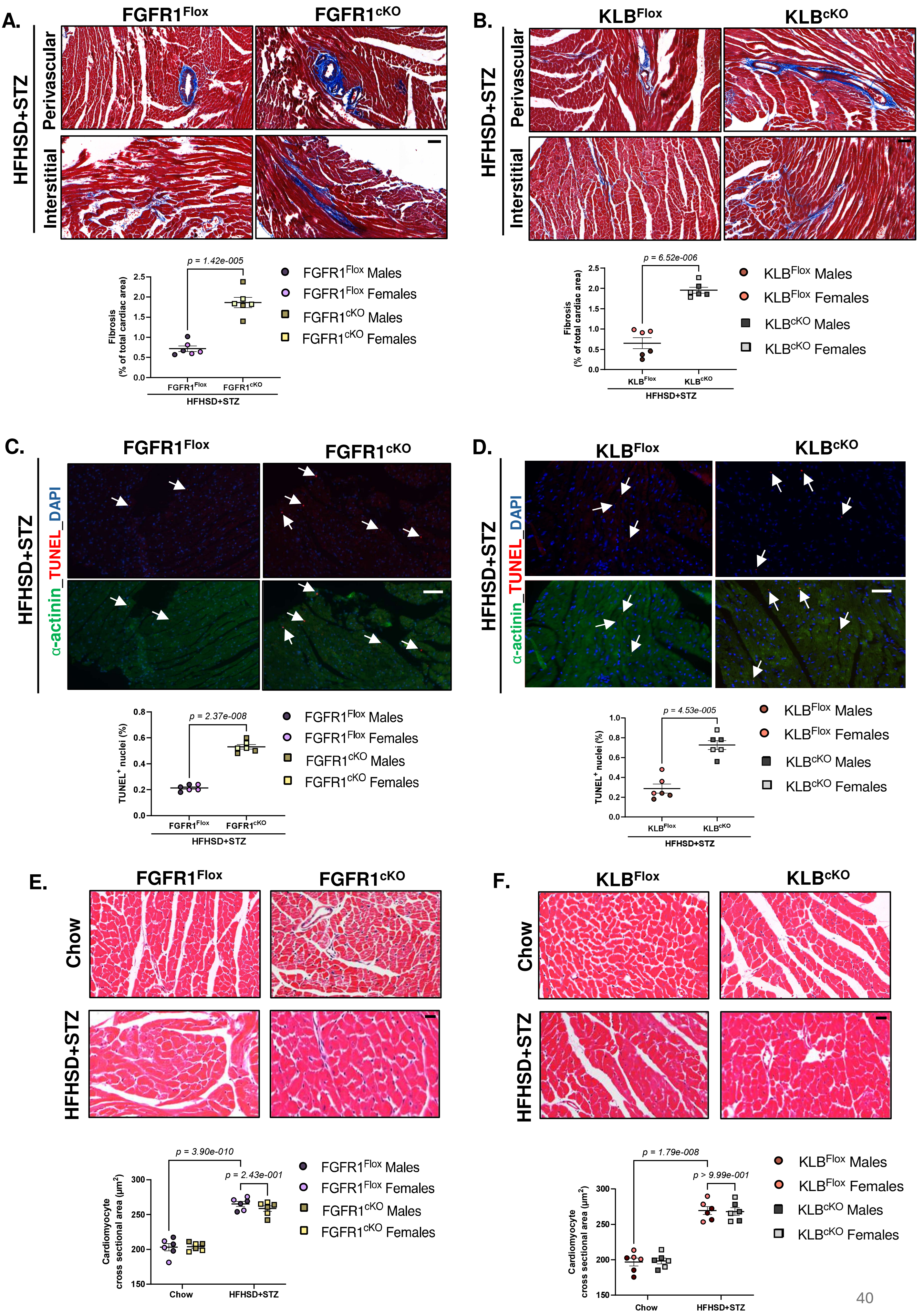
A.
FGFR1Flox
FGFR1cKO
HFHSD+STZ
Perivascular
Interstitial
p = 1.42e-005
Fibrosis (% of total cardiac area)
FGFR1Flox Males
FGFR1Flox Females
FGFR1cKO Males
FGFR1cKO Females
B.
KLBFlox
KLBcKO
p = 6.52e-006
KLBFlox Males
KLBFlox Females
KLBcKO Males
KLBcKO Females
C.
α-actinin_TUNEL_DAPI
p = 2.37e-008
TUNEL+ nuclei (%)
D.
p = 4.53e-005
E.
Chow
p = 3.90e-010
p = 2.43e-001
Cardiomyocyte cross sectional area (µm²)
F.
p = 1.79e-008
p > 9.99e-001

G.

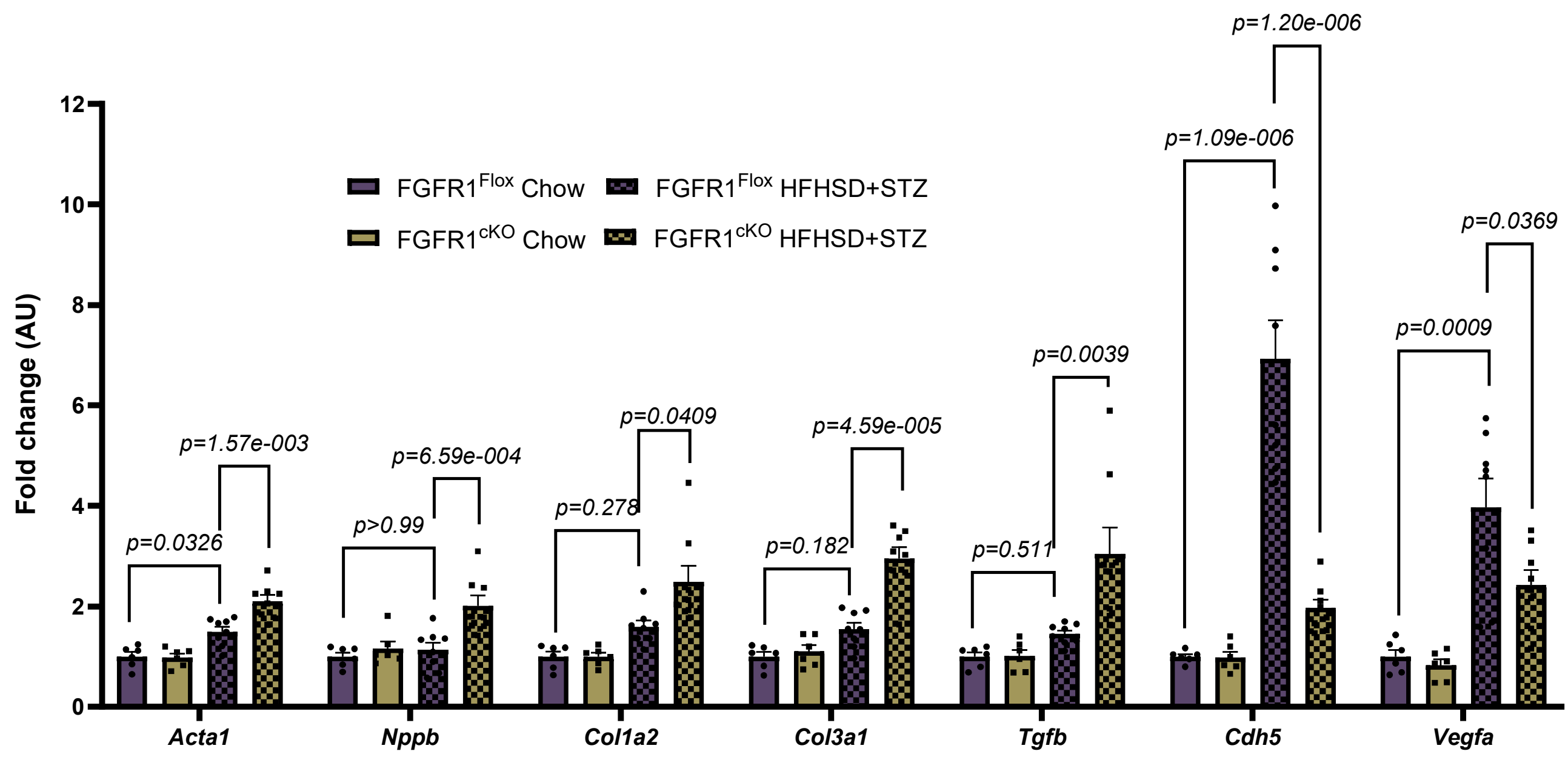


H.

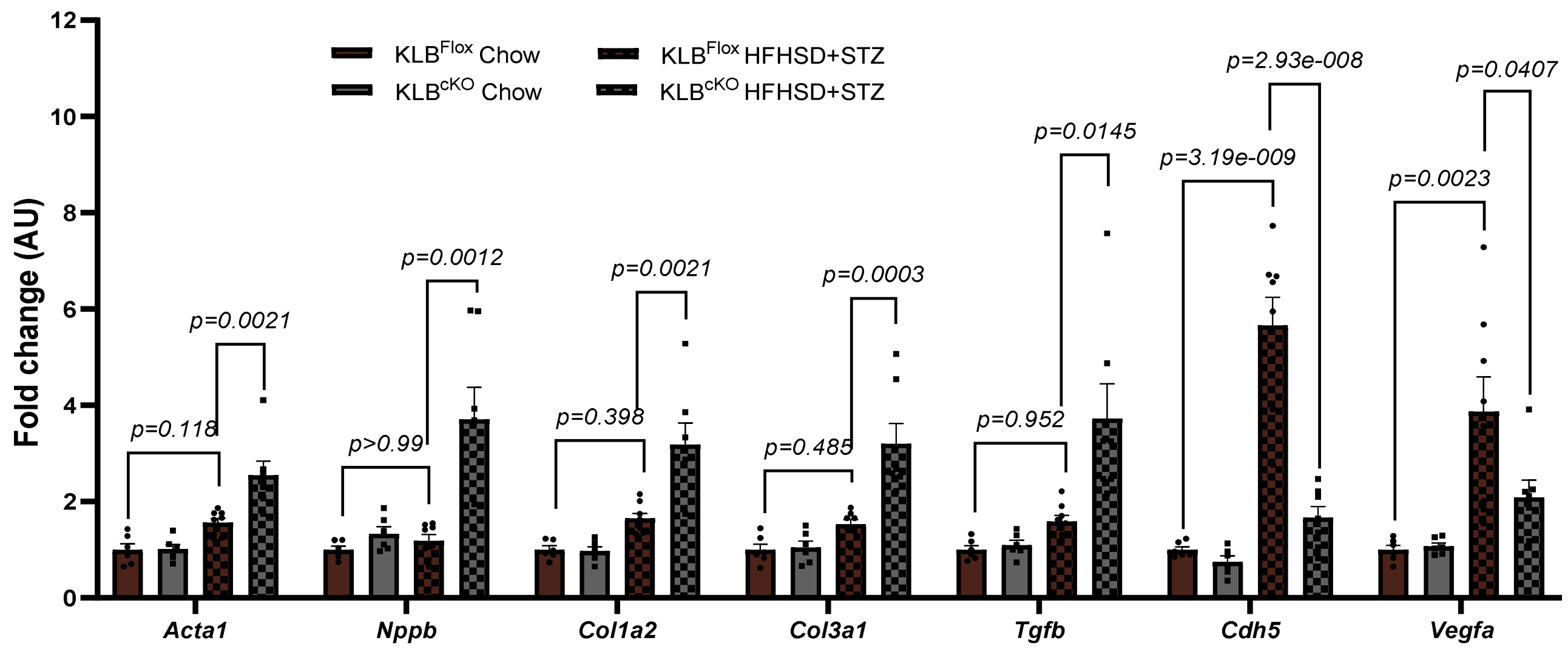


**Supplemental Figure 7. HFHSD+STZ-induced cardiac remodeling is exacerbated in cardiomyocyte-specific FGFR1 and KLB knockout mice under diabetic stress.**

**A-B,** Representative Masson's Trichrome-stained heart sections showing perivascular and interstitial fibrosis along with quantification graph under high-fat, high-sucrose diet (HFHSD) plus streptozotocin (STZ) treated males and females (**A**) FGFR1$^{cKO}$ and (**B**) KLB$^{cKO}$ mice versus respective floxed controls (N=6 hearts from both sexes/group; scale=50 µm). **C-D,** TUNEL-stained heart sections showing TUNEL-positive nuclei (red; white arrows) and respective quantification graphs in HFHSD+STZ-treated males and females (**C**) FGFR1$^{cKO}$ and (**D**) KLB$^{cKO}$ mice versus respective floxed controls (N=6 hearts from both sexes/group; scale=50 µm). DAPI is shown in blue, and green represents α-actinin. **E-F,** Representative H&E-stained cardiomyocyte cross sections and respective quantification graphs in (**E**) FGFR1$^{cKO}$ and (**F**) KLB$^{cKO}$ mice under chow or HFHSD+STZ stress (N=6 hearts from both sexes/group; scale=20 µm). **G-H,** Quantitative PCR analysis of transcripts involved in remodeling (*Acta1*, *Nppb*, *Col1a2*, *Col3a1*, *Tgfb*) and angiogenesis (*Cdh5*, *Vegfa*) in heart tissue of (**G**) FGFR1$^{cKO}$ and (**H**) KLB$^{cKO}$ mice under chow or HFHSD+STZ stress (N=4-5 mice of both sexes/chow-fed group and N=7-8 mice of both sexes/HFHSD+STZ-fed group). Data represent mean±SEM. Statistical significance values (indicated in each panel) were calculated versus corresponding controls using 2-tailed unpaired Student *t*-test (**A**-**D**) and ordinary 2-way ANOVA followed by Šidák multiple comparisons (**E-H**).

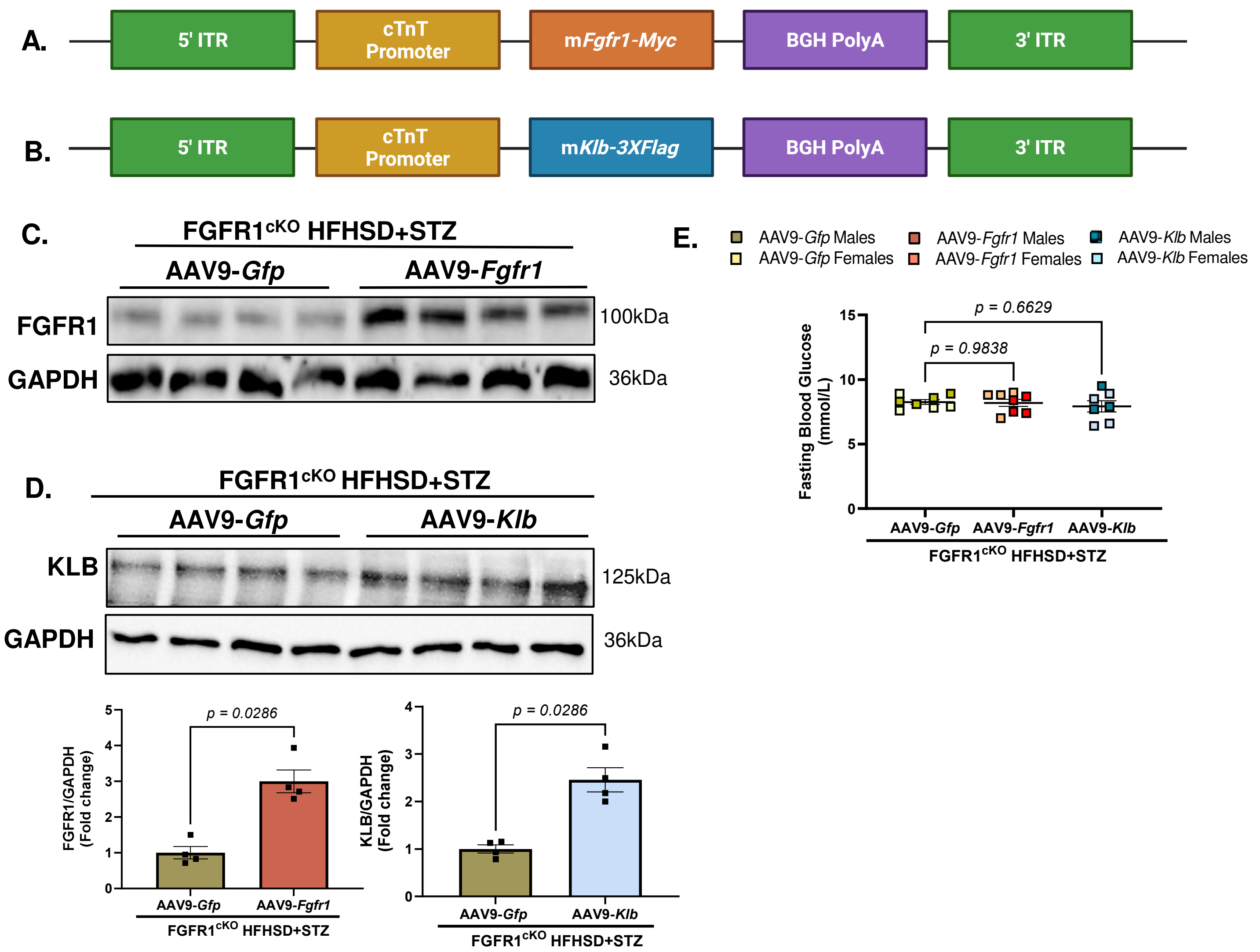


**Supplemental Figure 8. Characterization of AAV9-*Fgfr1* and AAV9-*Klb* treatment in FGFR1$^{cKO}$ mice under HFHSD+STZ stress.**

**A-B,** Adeno-associated virus serotype 9 (AAV9) viral vector construct of (**A**) AAV9-*Fgfr1* and (**B**) AAV9-*Klb.* **C-D,** Representative immunoblots and corresponding quantification from the hearts of FGFR1$^{cKO}$ mice treated with (**C**) AAV9-*Fgfr1* or (**D**) AAV9-*Klb,* respectively under high-fat, high-sucrose diet (HFHSD) plus streptozotocin (STZ) stress. GAPDH was used as the loading control (N=4 hearts from both sexes/group). **E,** Fasting blood glucose levels in male and female FGFR1$^{cKO}$ mice treated with AAV9-*Gfp,* AAV9-*Fgfr1* or AAV9-*Klb* following HFHSD+STZ stress (N=7-8 mice from both sexes/group). Data are presented as mean±SEM. Statistical comparisons (indicated in the panel) were performed using the Mann-Whitney U test (**C-D**) and ordinary 1-way ANOVA followed by Dunnett's multiple comparisons (**E**).

*Supplemental Figure 9*

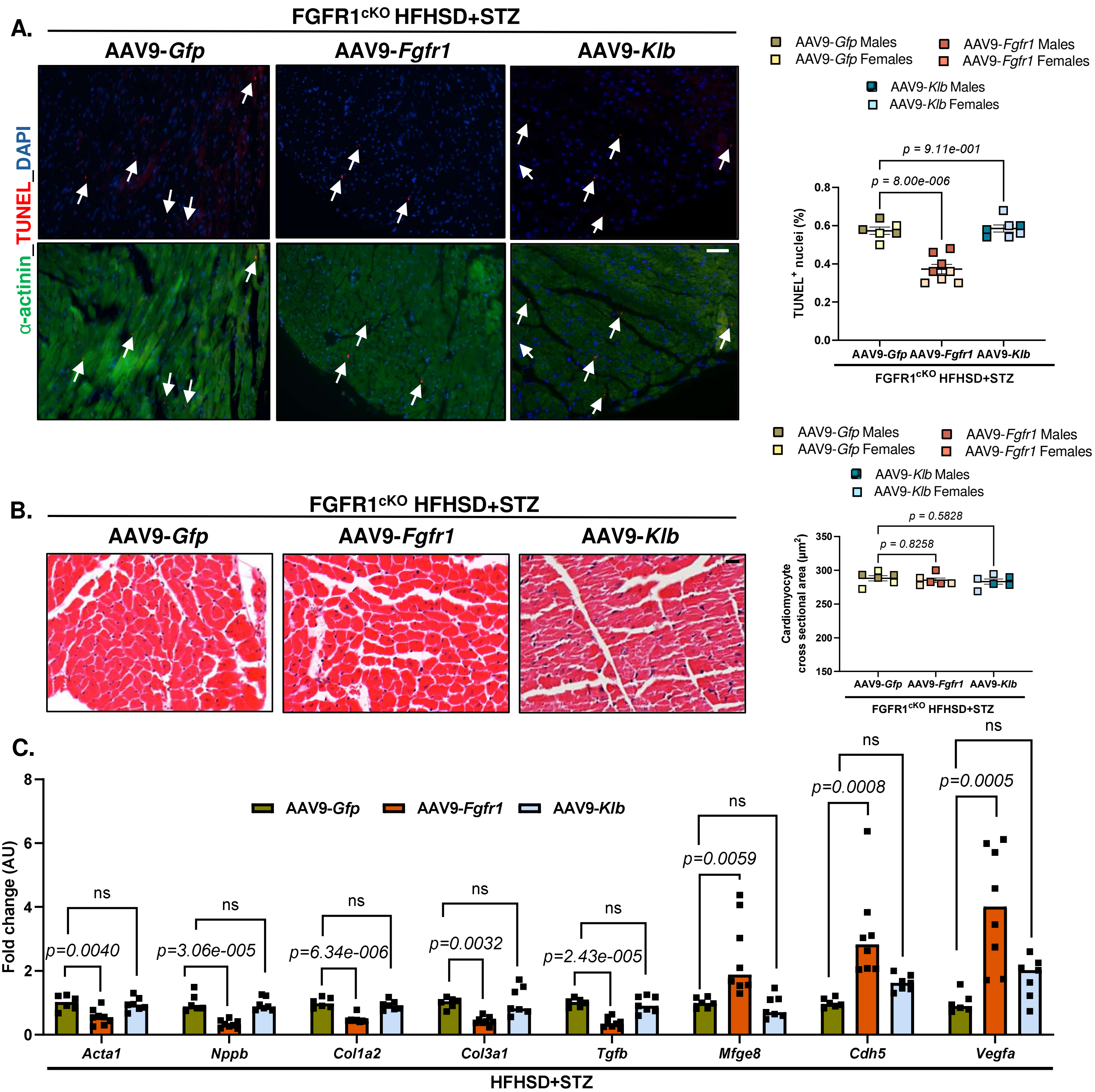


**Supplemental Figure 9. AAV9-*Fgfr1* treatment attenuates HFHSD+STZ-induced cardiac pathological remodeling in FGFR1$^{cKO}$ mice, while AAV9-*Klb* treatment has no effects.**

**A,** TUNEL-stained heart sections showing TUNEL-positive nuclei (red; white arrows) and quantification graph in FGFR1$^{cKO}$ mice treated with adeno-associated virus serotype 9 (AAV9) mediated treatment (AAV9-*Gfp*, AAV9-*Fgfr1*, or AAV9-*Klb)* following high-fat, high-sucrose diet (HFHSD) plus streptozotocin (STZ) stress (N=6 mice of both sexes/AAV9-*Gfp*-treated group and N=7-8 mice of both sexes/AAV9-*Fgfr1* and AAV9-*Klb*-treated groups; scale=50 μm). DAPI is shown in blue, and green represents α-actinin. **B,** Representative H&E-stained cardiac sections and corresponding quantification of cardiomyocyte size (N=6 mice of both sexes/group; scale=20 μm). **C,** Quantitative PCR analyses of cardiac pathological remodeling markers (*Acta1*, *Nppb*, *Col1a2*, *Col3a1*, *Tgfb*) and angiogenesis-associated markers (*Cdh5*, *Vegfa*) in the myocardium (N=6 mice of both sexes/AAV9-*Gfp*-treated group and N=7-8 mice of both sexes/AAV9-*Fgfr1* and AAV9-*Klb*-treated groups). Data represent mean±SEM. Statistical significance values (indicated in each panel) were calculated versus corresponding controls using ordinary 1-way ANOVA with Šidák multiple comparisons (**A-C**).

## Supplemental Figure 10

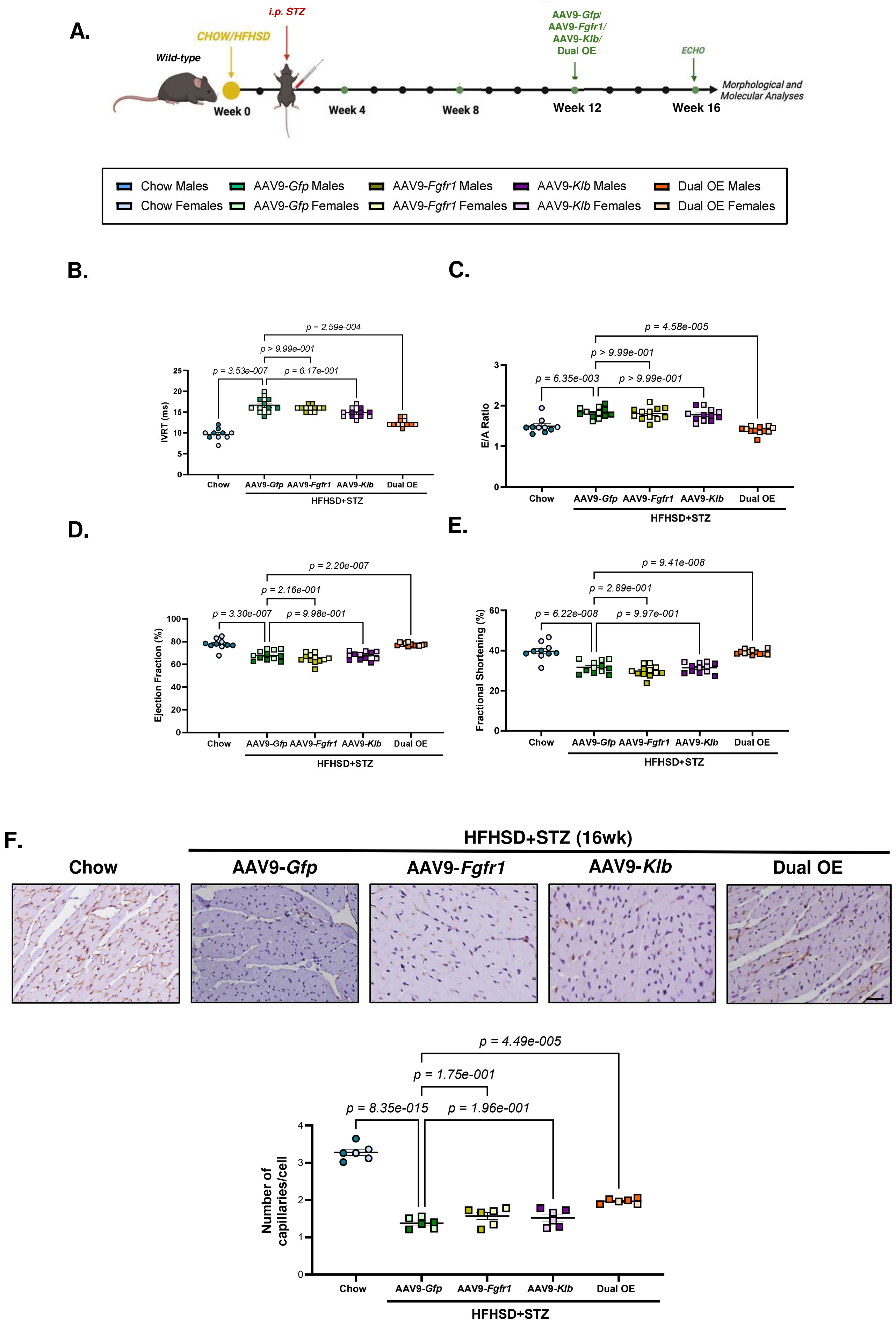

G.

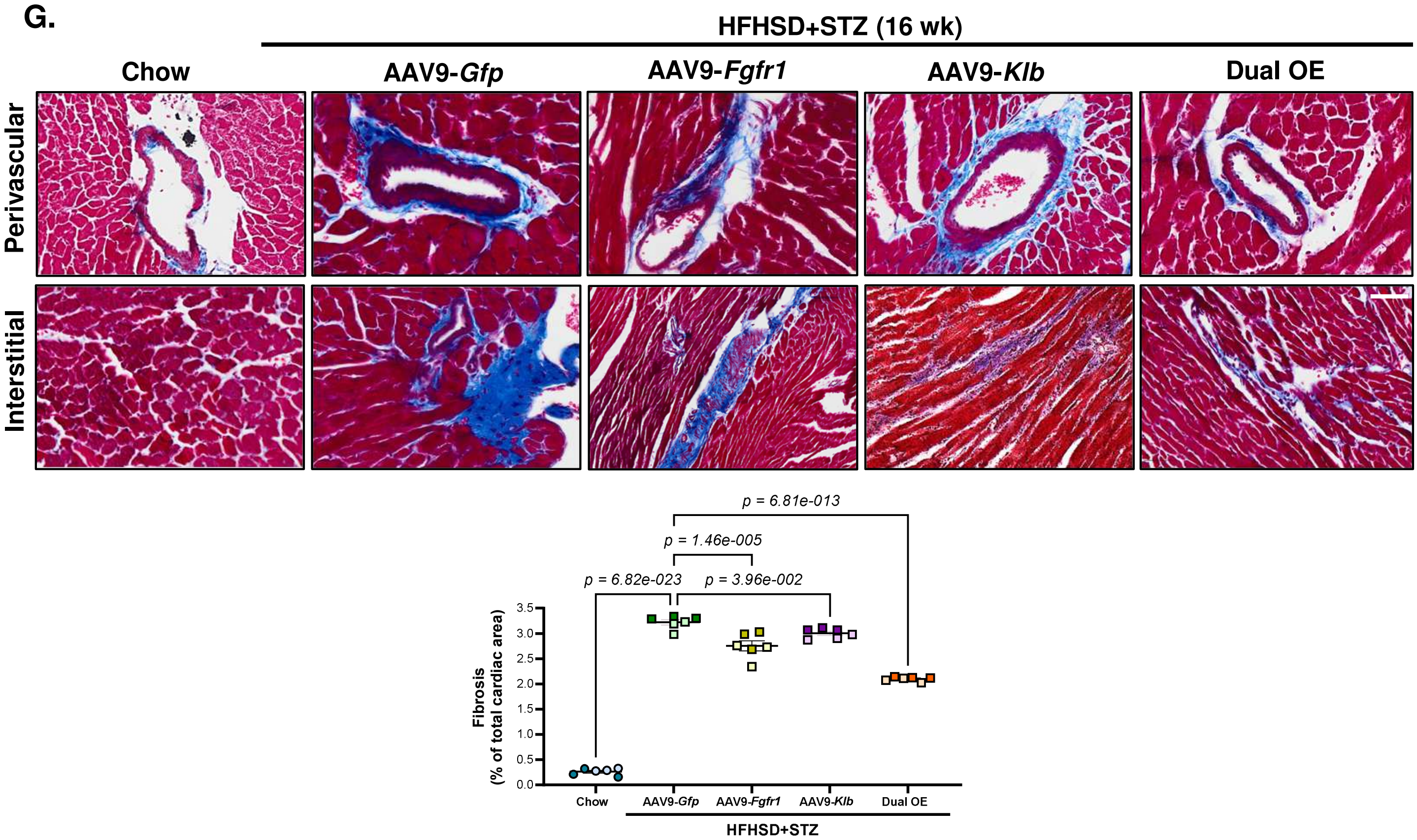


H.

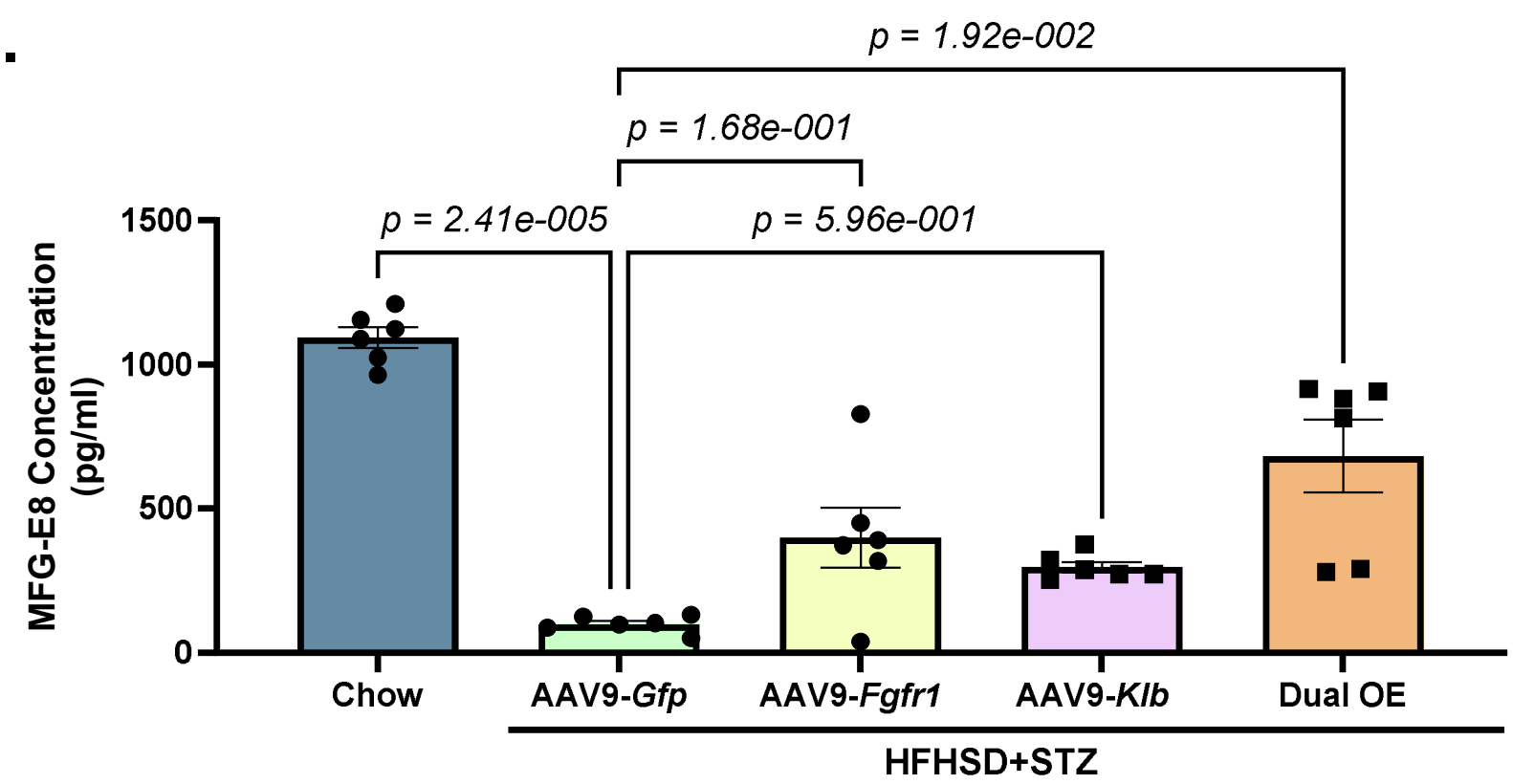


**Supplemental Figure 10**. **Dual overexpression of FGFR1 and KLB ameliorates diabetes-induced cardiovascular impairment.**

**A,** Schematic illustration of experimental design. Wild-type mice subjected to a high-fat, high-sucrose diet combined with streptozotocin (HFHSD+STZ) for 16 weeks were injected with AAV9-*Gfp*, AAV9-*Fgfr1*, AAV9-*Klb,* or both AAV9-F*gfr1* and AAV9-*Klb* (Dual OE) after 12 weeks. **B-E,** Cardiac functional parameters post-treatment. (**B**) Isovolumic relaxation time (IVRT), (**C**) Ratio of peak velocity blood flow from left ventricular relaxation in early diastole to that in late diastole (E/A), (**D**) Percentage of left ventricular ejection fraction, (**E**) Percentage of left ventricular fractional shortening (N=10-12 mice of both sexes/group). **F,** Immunohistochemical staining of CD31 and quantification of capillary density (capillaries/cell) (N=6 hearts from both sexes/group; scale=20 µm). **G,** Representative Masson's Trichrome-stained heart sections showing perivascular and interstitial fibrosis along with quantification in wild-type mice after different treatments under HFHSD+STZ stress (N=6 from both sexes/group; scale=50 µm). **H,** ELISA of MFG-E8 in the heart tissue obtained from mice after said treatments (N=6 independent biological replicates/group). Data are presented as mean±SEM. Statistical significance (indicated in each panel) was assessed using ordinary 1-way ANOVA with Holm-Sidak multiple comparisons (**D-G**) and the Kruskal-Wallis with Dunn's multiple comparisons (**B, C, H**).

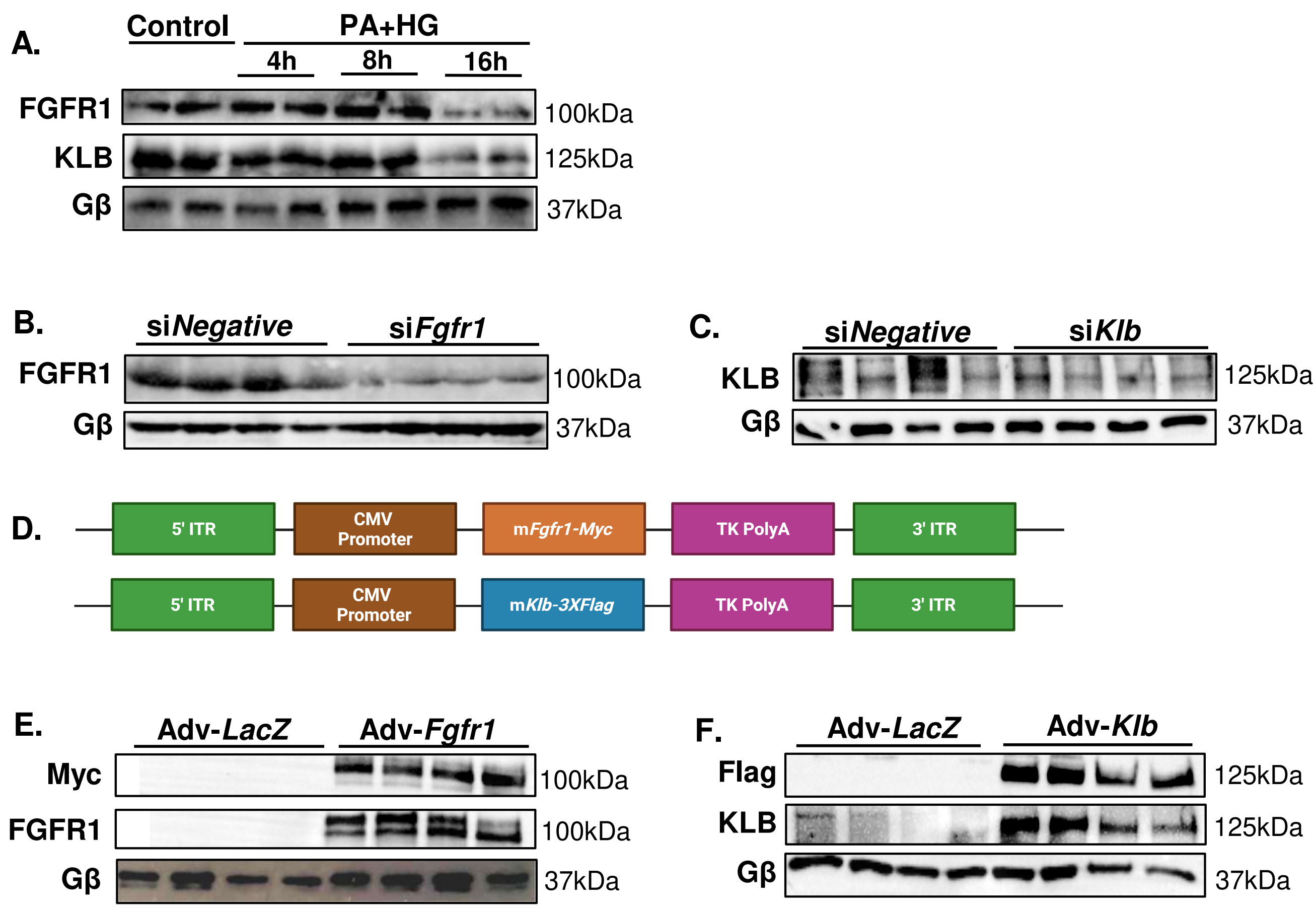


**Supplemental Figure 11. Characterization of FGFR1 and KLB knockdown and overexpression *in vitro*.**

**A,** Representative immunoblot showing endogenous FGFR1 and KLB expression in response to palmitic acid (PA, 500 μmol/L) and high glucose (HG, 25 mmol/L) stimulation for 4, 8, and 16 hours in H9C2s. Gβ was used as the loading control. **B-C,** Representative immunoblots showing si*RNA*-mediated knockdown of (**B**) FGFR1 and (**C**) KLB in neonatal rat cardiomyocytes (NRCMs). Gβ was used as the loading control. **D,** Construct of adenovirus (Adv) overexpressing mouse FGFR1 (Adv-*Fgfr1*; top) and mouse KLB (Adv-*Klb*; bottom). **E-F,** Representative immunoblots confirming overexpression of (**E**) FGFR1 with a Myc epitope tag and (**F**) KLB with a Flag epitope tag by adenoviral infection with Adv-*Fgfr1* and Adv-*Klb,* respectively, in NRCMs. Gβ was used as the loading control.

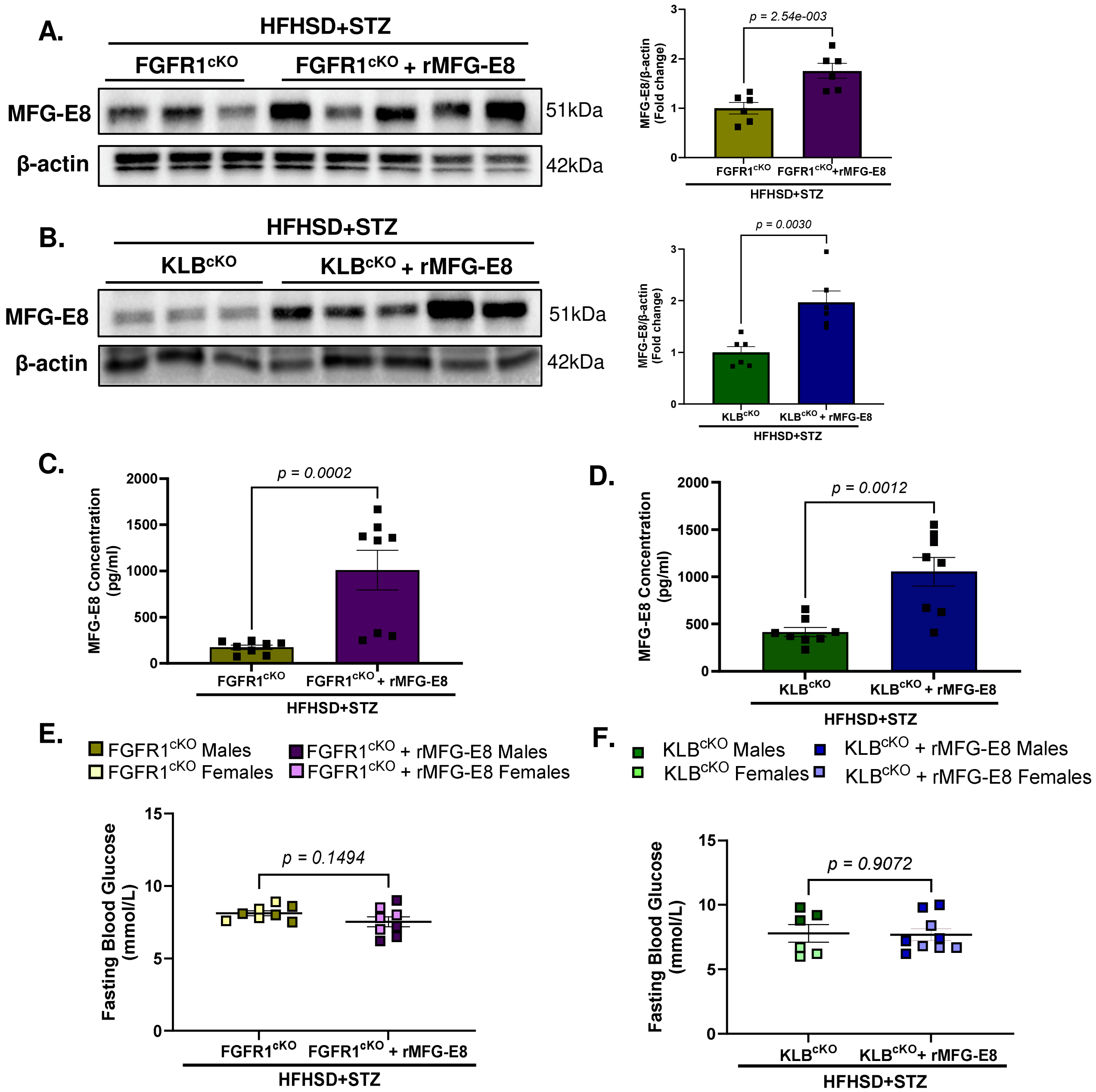


**Supplemental Figure 12. Characterization of recombinant MFG-E8 treatment.**

**A-B,** Representative immunoblots and corresponding quantification of MFG-E8 expression in the hearts of (**A**) FGFR1$^{cKO}$ mice and (**B**) KLB$^{cKO}$ mice with or without recombinant MFG-E8 (rMFG-E8; 40 μg/kg weekly) treatment (N=6 hearts from both sexes/group) under high-fat, high-sucrose diet plus streptozotocin (HFHSD+STZ) stress. β-actin was used as the loading control. **C-D,** Serum MFG-E8 levels in (**C**) FGFR1$^{cKO}$ and (**D**) KLB$^{cKO}$ mice following rMFG-E8 treatment (N=8 hearts from both sexes/group). **E-F,** Fasting blood glucose levels in male and female (**E**) FGFR1$^{cKO}$ (N=8 mice from both sexes/group) and (**F**) KLB$^{cKO}$ (N=6 in KLB$^{cKO}$ and 9 in rMFG-E8 treated KLB$^{cKO}$ mice from both sexes/group) mice treated with rMFG-E8 following HFHSD+STZ stress. Data represent mean±SEM. Statistical comparisons (indicated in each panel) versus corresponding controls were performed using 2-tailed unpaired Student *t*-test (**A-B**, **D-F**) and the Mann-Whitney U test (**C**).

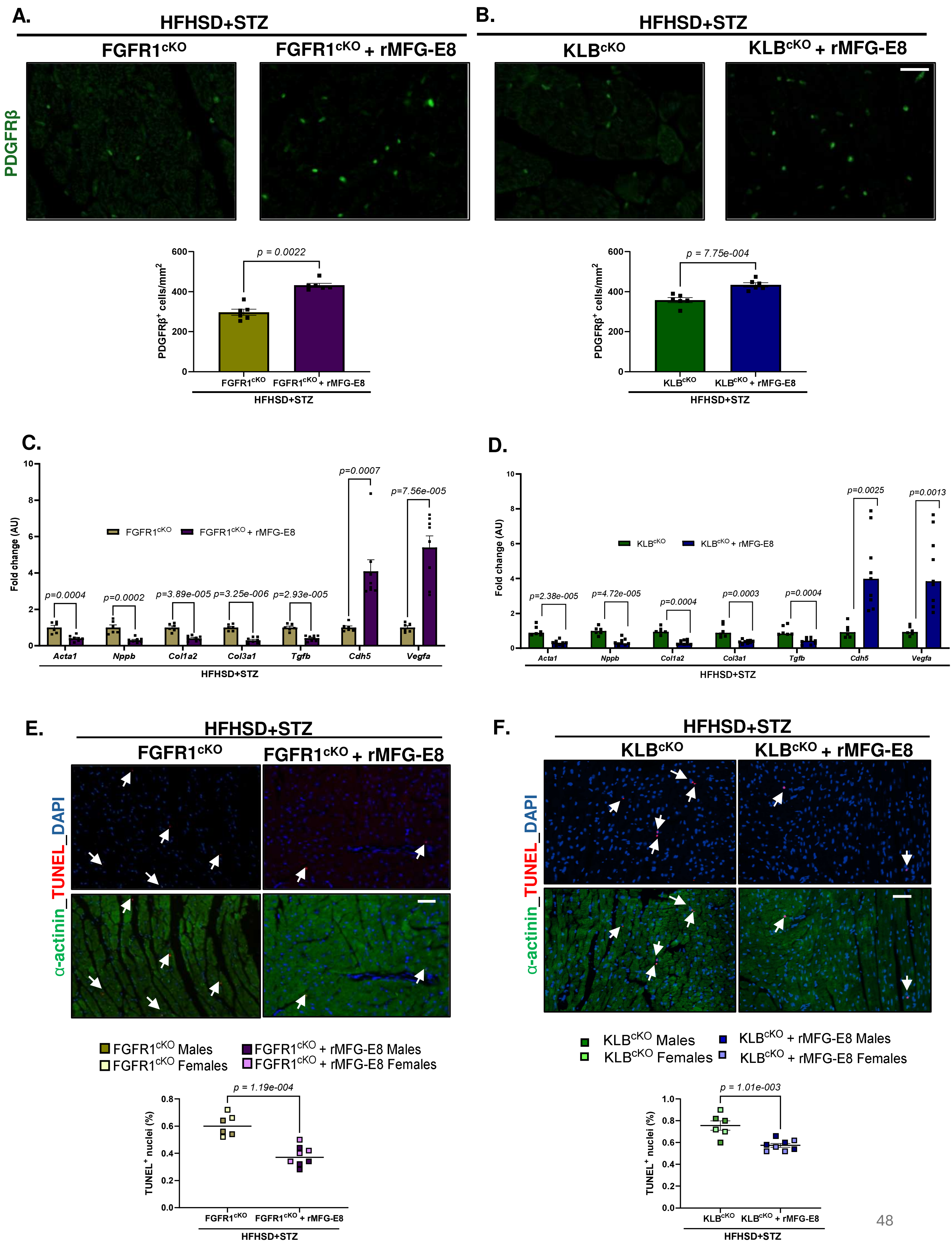
A.
HFHSD+STZ
FGFR1cKO
FGFR1cKO + rMFG-E8
PDGFRβ
B.
HFHSD+STZ
KLBcKO
KLBcKO + rMFG-E8
p = 0.0022
PDGFRβ+ cells/mm2
FGFR1cKO
FGFR1cKO + rMFG-E8
HFHSD+STZ
p = 7.75e-004
KLBcKO
KLBcKO + rMFG-E8
HFHSD+STZ
C.
Fold change (AU)
FGFR1cKO
FGFR1cKO + rMFG-E8
p=0.0004
p=0.0002
p=3.89e-005
p=3.25e-006
p=2.93e-005
p=0.0007
p=7.56e-005
Acta1
Nppb
Col1a2
Col3a1
Tgfb
Cdh5
Vegfa
HFHSD+STZ
D.
KLBcKO
KLBcKO + rMFG-E8
p=2.38e-005
p=4.72e-005
p=0.0004
p=0.0003
p=0.0004
p=0.0025
p=0.0013
HFHSD+STZ
E.
HFHSD+STZ
FGFR1cKO
FGFR1cKO + rMFG-E8
α-actinin_TUNEL_DAPI
F.
HFHSD+STZ
KLBcKO
KLBcKO + rMFG-E8
FGFR1cKO Males
FGFR1cKO Females
FGFR1cKO + rMFG-E8 Males
FGFR1cKO + rMFG-E8 Females
p = 1.19e-004
TUNEL+ nuclei (%)
KLBcKO Males
KLBcKO Females
KLBcKO + rMFG-E8 Males
KLBcKO + rMFG-E8 Females
p = 1.01e-003

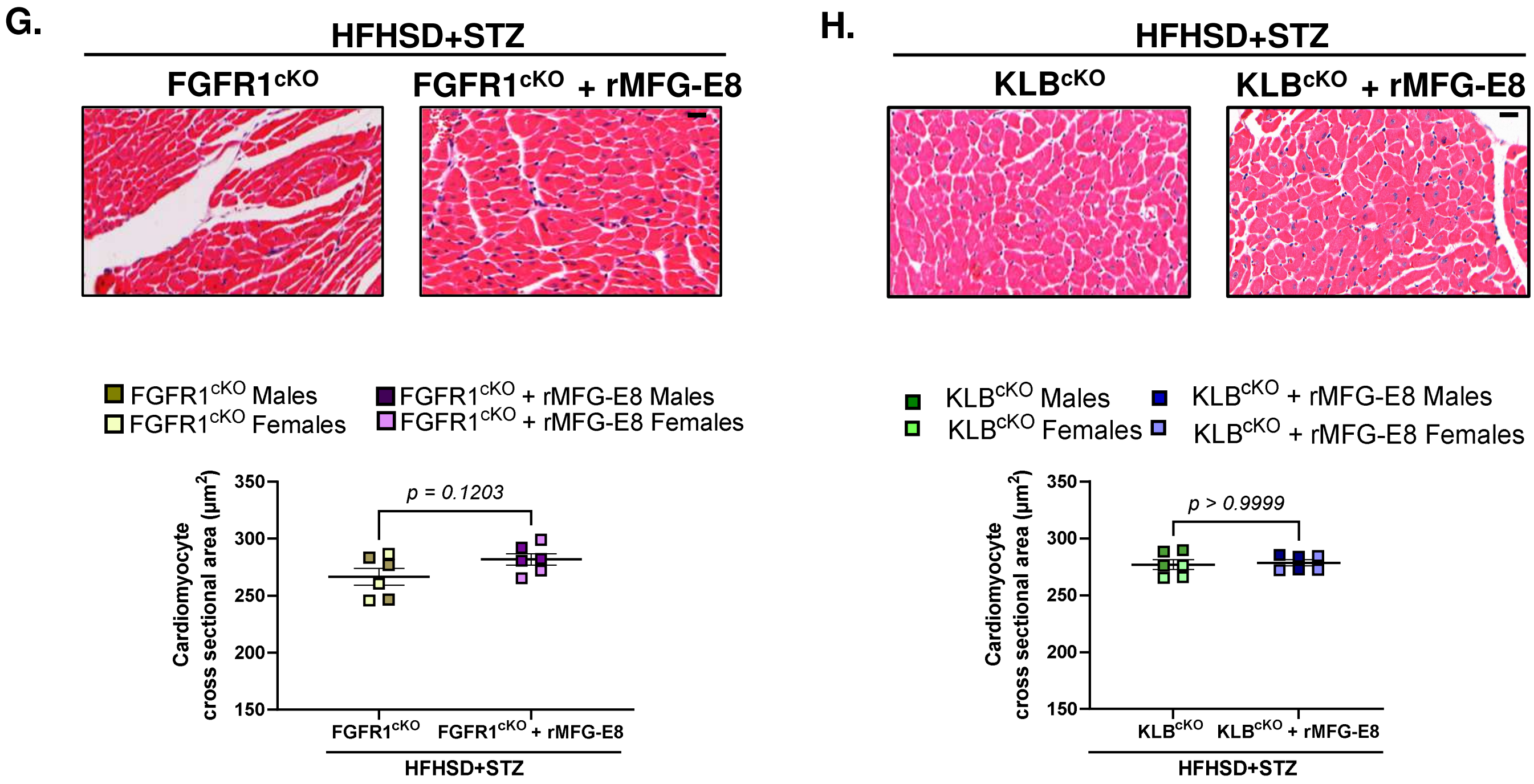


**Supplemental Figure 13. Recombinant MFG-E8 treatment alleviates pericyte number, apoptosis, and pathological remodeling in FGFR1$^{cKO}$ and KLB$^{cKO}$ hearts.**

**A-B,** Representative PDGFRβ immunostaining (green) and respective quantification of pericyte density in (**A**) FGFR1$^{cKO}$ and (**B**) KLB$^{cKO}$ mice following recombinant MFG-E8 (rMFG-E8; 40 µg/kg weekly) treatment under high-fat, high-sucrose diet with streptozotocin (HFHSD+STZ) stress (N=6 hearts from both sexes/group; scale=20 µm). **C-D,** Quantitative PCR analyses of cardiac pathological remodeling markers (*Acta1*, *Nppb*, *Col1a2*, *Col3a1*, *Tgfb*) and angiogenesis-associated genes (*Cdh5*, *Vegfa*) in (**C**) FGFR1$^{cKO}$ hearts and (**D**) KLB$^{cKO}$ mice with or without treatment (N=6 mice of both sexes/control group and N=8-9 mice of both sexes/rMFG-E8 treated group). **E-F,** TUNEL-stained heart sections showing TUNEL-positive nuclei (red; white arrows) and quantification graphs in (**E**) FGFR1$^{cKO}$ and (**F**) KLB$^{cKO}$ mice treated with rMFG-E8 following HFHSD+STZ stress ((N=6 mice of both sexes/control group and N=8 mice of both sexes/rMFG-E8 treated group; scale=50 µm). DAPI is shown in blue, and green represents α-actinin. **G-H,** Representative H&E-stained cardiac cross-sections and corresponding quantification of cardiomyocyte size in (**G**) FGFR1$^{cKO}$ and (**H**) KLB$^{cKO}$ mice treated with rMFG-E8 (N=6 mice of both sexes /group; scale=20 µm). Data represent mean±SEM. Statistical comparisons (indicated in each panel) versus corresponding controls were performed using the Mann-Whitney U test (**A, C-*Cdh5*, D-*Col1a2*, D-*Tgfb*, H**) and 2-tailed unpaired Student *t*-test (**B, C-other genes, D-other genes, E-G**).

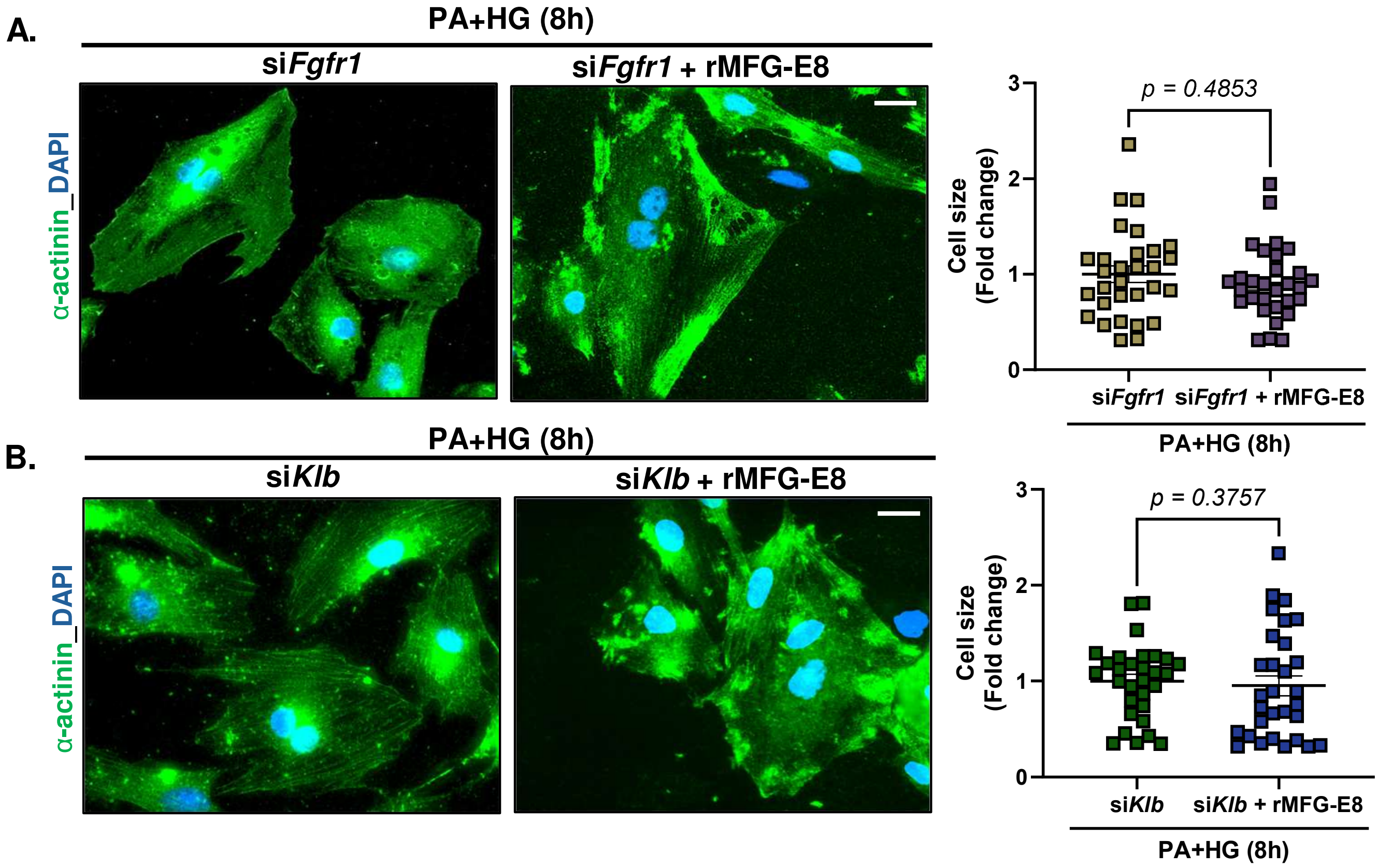


**Supplemental Figure 14. Recombinant MFG-E8 treatment does not affect cell size in FGFR1- or KLB-knockdown H9C2 cells.**

**A,** Representative images and quantification of immunofluorescent co-staining of α-actinin (green) and DAPI (blue) in FGFR1-knockdown H9C2 cardiomyoblasts by its si*RNA* (si*Fgfr1*) or in **B,** KLB-knockdown H9C2 cardiomyoblasts by its si*RNA* (si*Klb*) in the presence and absence of recombinant milk fat globule-EGF factor 8 (rMFG-E8, 1 µg/mL) for 24 hours under palmitic acid (PA, 500 µmol/L) and high glucose (HG, 25 mmol/L) stress (N=30 cells from 3 independent biological replicates; scale=20 µm). Data are presented as mean±SEM. Statistical comparisons (indicated in each figure) versus respective controls were performed using the Mann-Whitney U test.

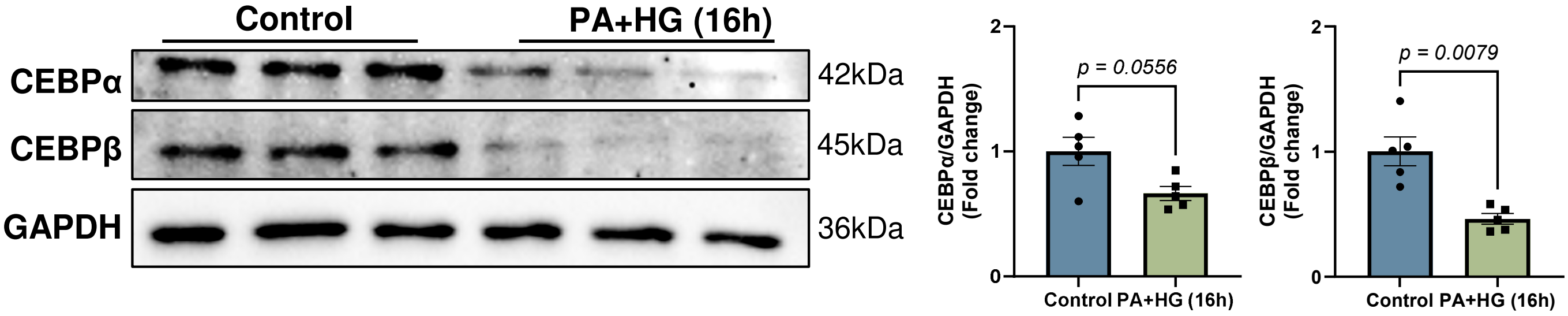


**Supplemental Figure 15. CEBPβ is affected most by prolonged diabetes-like stress in H9C2 cells.**

Representative immunoblots and quantification of CEBPα (CCAAT/enhancer-binding protein alpha) and CEBPβ (CCAAT/enhancer-binding protein beta) expression in H9C2 cardiomyoblasts under prolonged palmitic acid (PA; 500 μmol/L) and high glucose (HG, 25 mmol/L) stress (N=5 independent biological replicates/group). GAPDH was used as the loading control. Data are presented as mean±SEM. Statistical comparisons (indicated in each figure) versus respective controls were performed using the Mann-Whitney U test.

***Supplemental Figure 16***

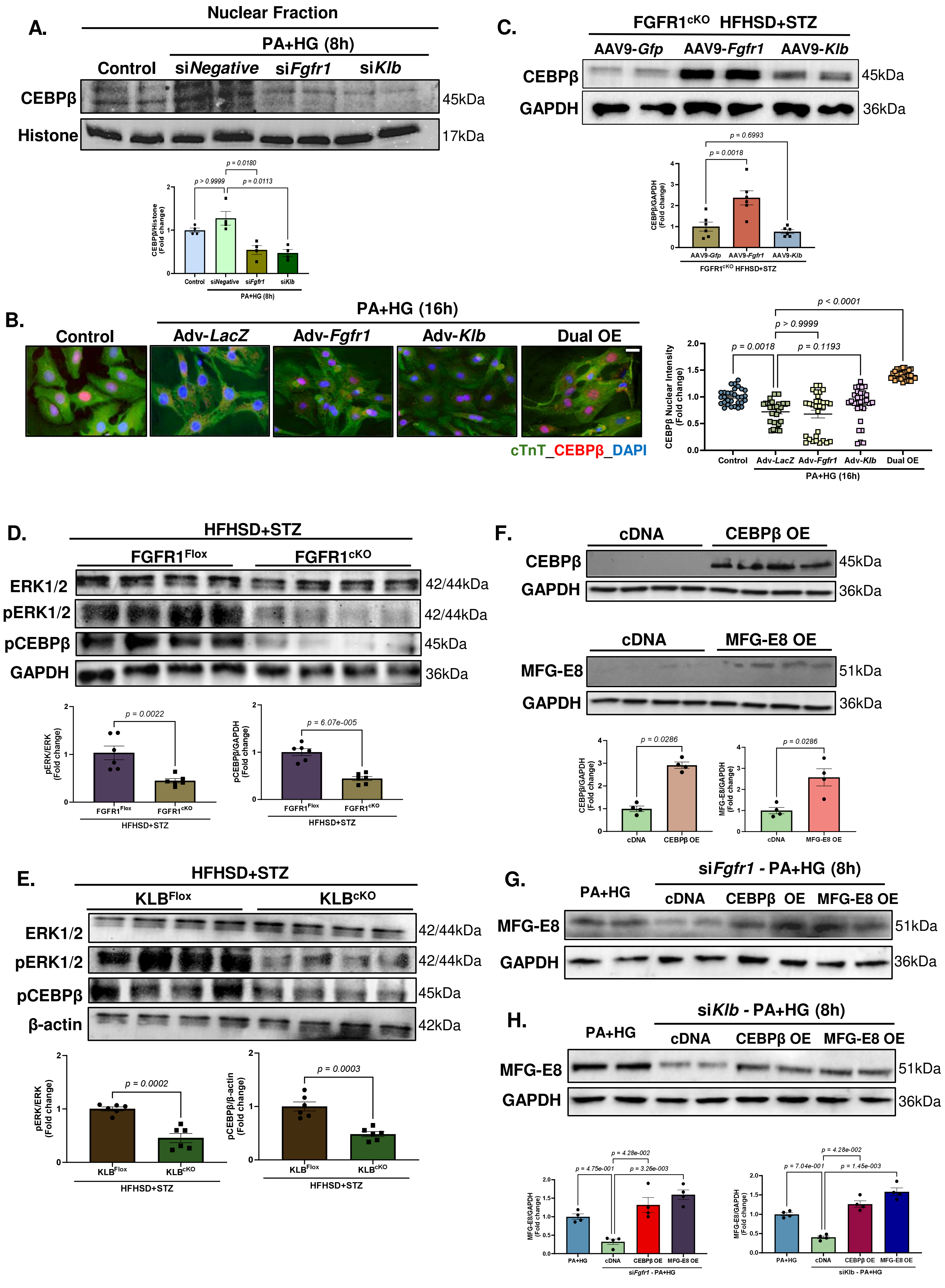

**Supplemental Figure 16. Cardiac FGFR1/KLB signaling regulates MFG-E8 through CEBPβ under diabetes-like stress.**

**A,** Representative immunoblot and quantification of nuclear CEBPβ expression in nuclear fraction isolated from FGFR1 (si*Fgfr1*) and KLB (si*Klb*) knockdown neonatal rat cardiomyocytes (NRCMs) under short term palmitic acid (PA; 500 μmol/L) and high glucose (HG, 25 mmol/L) stress (N=4 independent biological replicates/group). Histone was used as the nuclear loading control. **B,** Immunofluorescent co-staining of cTnT (cardiac troponin T; green), CEBPβ (CCAAT/enhancer-binding protein beta; red), and DAPI (blue) in NRCMs with single or dual adenoviral (Adv) overexpression of FGFR1 (Adv-*Fgfr1*) and KLB (Adv-*Klb*) under prolonged PA+HG stress with corresponding quantification of nuclear CEBPβ intensity compared to controls (N=30 cells from 3 independent biological replicates/group; scale=20 μm). **C,** Representative immunoblot and quantification of cardiac CEBPβ expression in FGFR1$^{cKO}$ mice under diabetic stress induced by high-fat, high-sucrose diet plus streptozotocin (HFHSD+STZ) stress, treated with adeno-associated virus serotype 9 (AAV9)-*Gfp*, AAV9-*Fgfr1,* or AAV9-*Klb* (N=6 hearts from both sexes/group). GAPDH was used as the loading control. **D-E,** Representative immunoblots of total ERK1/2, phosphorylated-ERK1/2 (pERK1/2), and phosphorylated-CEBPβ (pCEBPβ) in (**D**) FGFR1$^{cKO}$ and (**E**) KLB$^{cKO}$ hearts under HFHSD+STZ stress. GAPDH and β-actin were used as the loading controls, respectively (N=6 hearts from both sexes/group). **F,** Representative immunoblots validating overexpression of CEBPβ (top) and MFG-E8 (bottom) in NRCMs. GAPDH was used as the loading control (N=4 independent biological replicates/group). **G-H,** Representative immunoblots showing cardiac MFG-E8 expression following CEBPβ or MFG-E8 overexpression in (**G**) FGFR1- or (**H**) KLB-knockdown NRCMs under PA+HG stress. GAPDH was used as the loading control (N=4 independent biological replicates/group). Data are presented as mean±SEM. Statistical comparisons (indicated in the panel) versus corresponding controls were performed using the Kruskal-Wallis test with Dunn's multiple comparisons (**A-B, G-H**), 2-tailed unpaired Student *t*-test (**D-E**), the Mann-Whitney U test (**F**), and ordinary 1-way ANOVA with Holm-Šidák multiple comparisons (**C).**

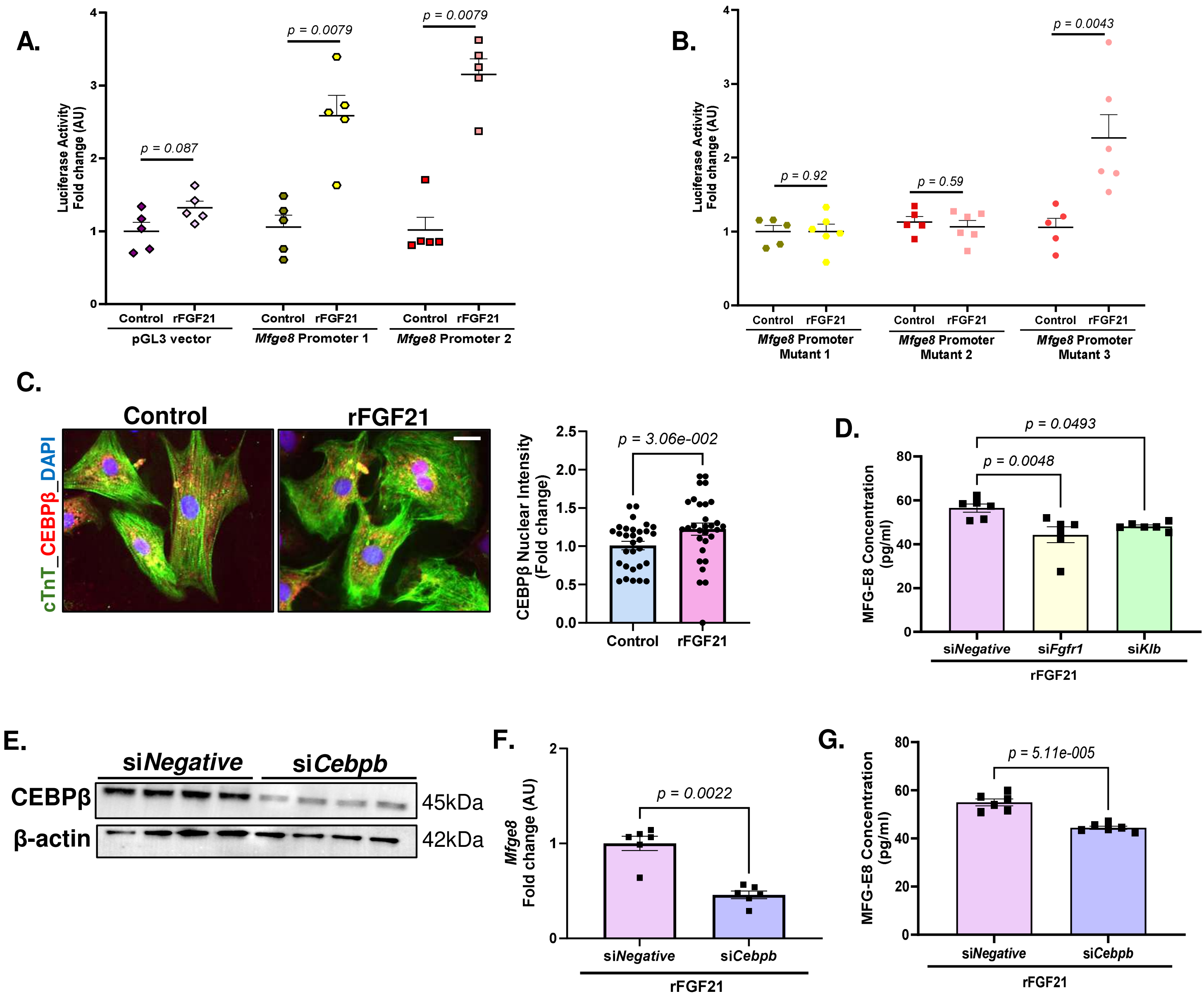


**Supplemental Figure 17. FGF21 induces MFG-E8 expression and secretion through the FGFR1/KLB-CEBPβ signaling axis.**

**A,** Luciferase reporter assays demonstrating that recombinant FGF21 (rFGF21; 500 ng/mL) significantly increased *Mfge8* promoter activity in neonatal rat cardiomyocytes (NRCMs) (N=5 independent biological replicates/group). **B,** Mutation of the CEBPβ binding sites within the MFG-E8 promoter abolished rFGF21-induced luciferase activation (N=5-6 independent biological replicates/group). **C,** Immunofluorescent co-staining of cTnT (cardiac troponin T; green), CEBPβ (CCAAT/enhancer-binding protein beta; red), and DAPI (blue) in NRCMs with rFGF21 treatment with corresponding quantification of nuclear CEBPβ intensity compared to controls (N=30 cells from 3 independent biological replicates/group; scale=20 μm). **D,** Quantification of secreted MFG-E8 in conditioned media from FGFR1- or KLB-deficient NRCMs treated with rFGF21 (N=6 independent biological replicates/group). **E,** Representative immunoblots validating knockdown of CEBPβ using 4 biological replicates in NRCMs using its siRNA. β-actin were used as the loading control. **F,** Quantitative PCR analysis of *Mfge8* with rFGF21 treatment in CEBPβ knockdown NRCMs (N=6 independent biological replicates/group). **G,** Quantification of secreted MFG-E8 in conditioned media from CEBPβ-deficient NRCMs treated with rFGF21 (N=6 independent biological replicates/group). Data are presented as mean±SEM. Statistical comparisons (indicated in the panel) versus corresponding controls were performed using permutation-based 2-way ANOVA test (**A-B**), the Mann-Whitney U test (**F**), ordinary 1-way ANOVA followed by Šidák multiple comparisons (**D**), and two-tailed unpaired Student's *t*-test (**C, G**).

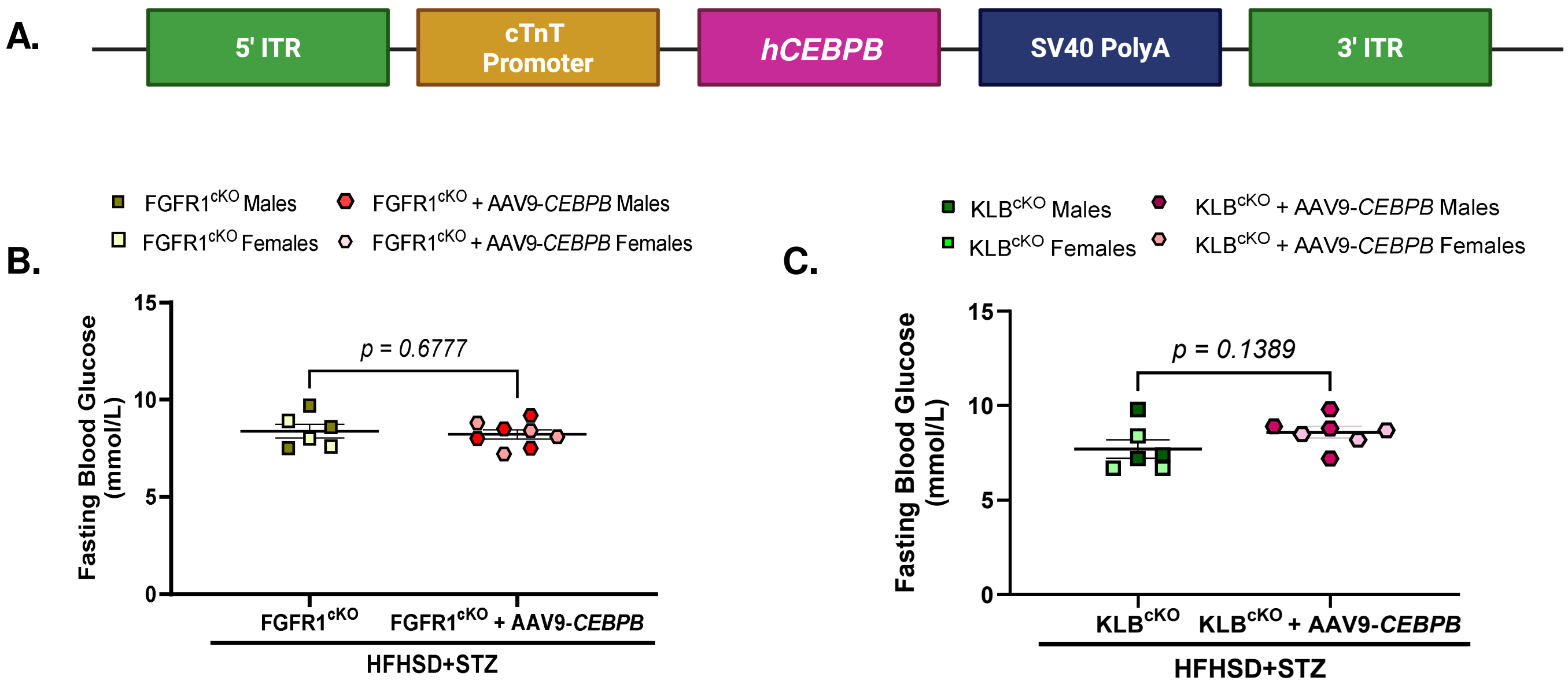


**Supplemental Figure 18. AAV9-*CEBPB* treatment does not alter the systemic metabolic profile in FGFR1$^{cKO}$ or KLB$^{cKO}$ mice under HFHSD+STZ stress.**

**A,** Adeno-associated virus serotype 9 (AAV9) viral vector construct of human CCAAT/enhancer-binding protein beta (AAV9-*CEBPB*). **B-C,** Fasting blood glucose levels in male and female (**B**) FGFR1$^{cKO}$ and (**C**) KLB$^{cKO}$ mice treated with AAV9-*CEBPB* following high-fat, high-sucrose diet (HFHSD) and streptozotocin (STZ) stress (N=6 mice of both sexes/chow-fed group and N=7-8 mice of both sexes/HFHSD+STZ-fed group). Data are presented as mean±SEM. Statistical comparison (indicated in the panel) versus corresponding controls was performed using 2-tailed unpaired Student *t*-test.

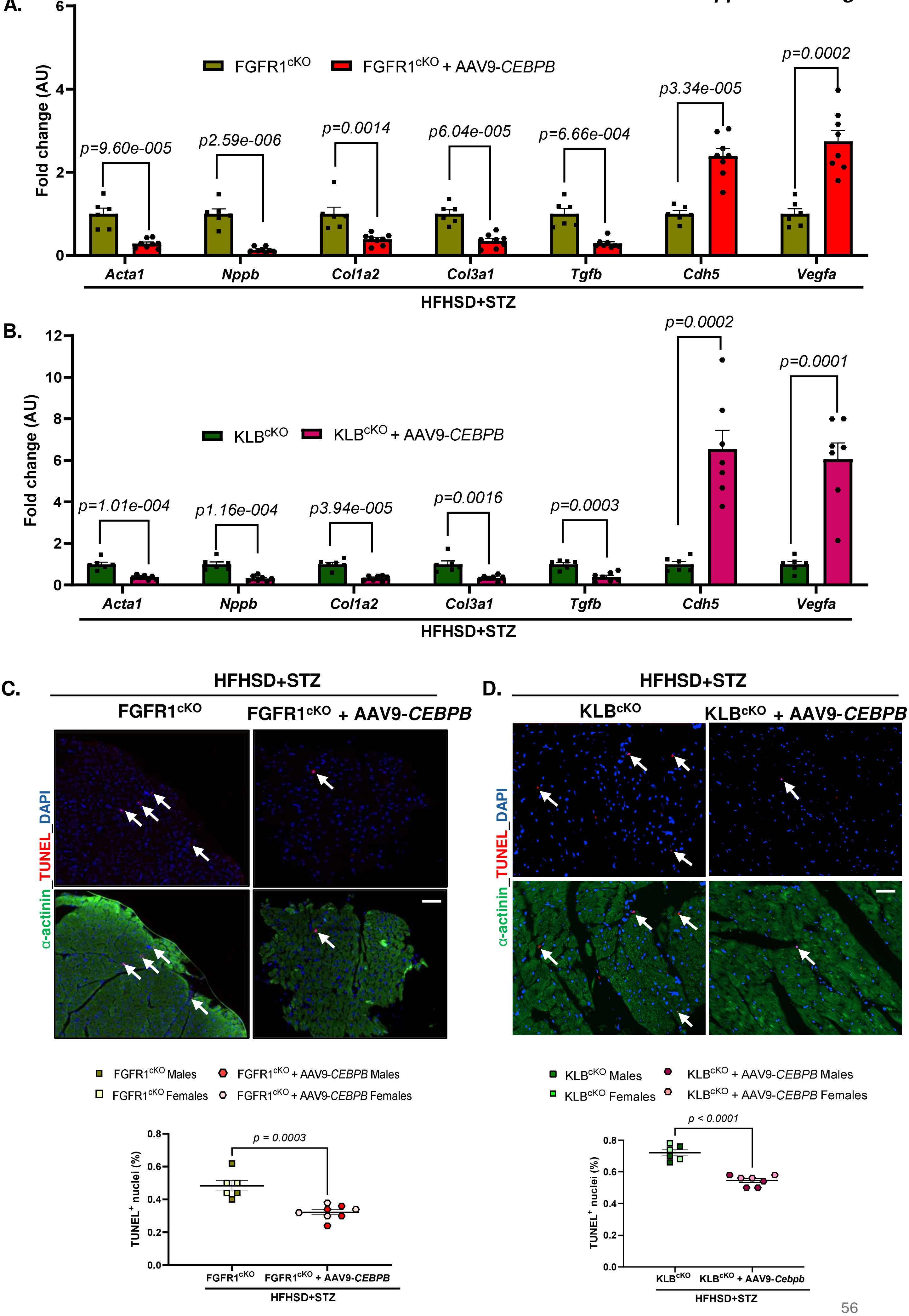
A.
Fold change (AU)
FGFR1cKO
FGFR1cKO + AAV9-CEBPB
p=9.60e-005
p2.59e-006
p=0.0014
p6.04e-005
p=6.66e-004
p3.34e-005
p=0.0002
Acta1
Nppb
Col1a2
Col3a1
Tgfb
Cdh5
Vegfa
HFHSD+STZ
B.
Fold change (AU)
KLBcKO
KLBcKO + AAV9-CEBPB
p=1.01e-004
p1.16e-004
p3.94e-005
p=0.0016
p=0.0003
p=0.0002
p=0.0001
Acta1
Nppb
Col1a2
Col3a1
Tgfb
Cdh5
Vegfa
HFHSD+STZ
C.
HFHSD+STZ
FGFR1cKO
FGFR1cKO + AAV9-CEBPB
α-actinin_TUNEL_DAPI
D.
HFHSD+STZ
KLBcKO
KLBcKO + AAV9-CEBPB
α-actinin_TUNEL_DAPI
FGFR1cKO Males
FGFR1cKO Females
FGFR1cKO + AAV9-CEBPB Males
FGFR1cKO + AAV9-CEBPB Females
TUNEL+ nuclei (%)
p = 0.0003
FGFR1cKO
FGFR1cKO + AAV9-CEBPB
HFHSD+STZ
KLBcKO Males
KLBcKO Females
KLBcKO + AAV9-CEBPB Males
KLBcKO + AAV9-CEBPB Females
TUNEL+ nuclei (%)
p < 0.0001
KLBcKO
KLBcKO + AAV9-Cebpb
HFHSD+STZ

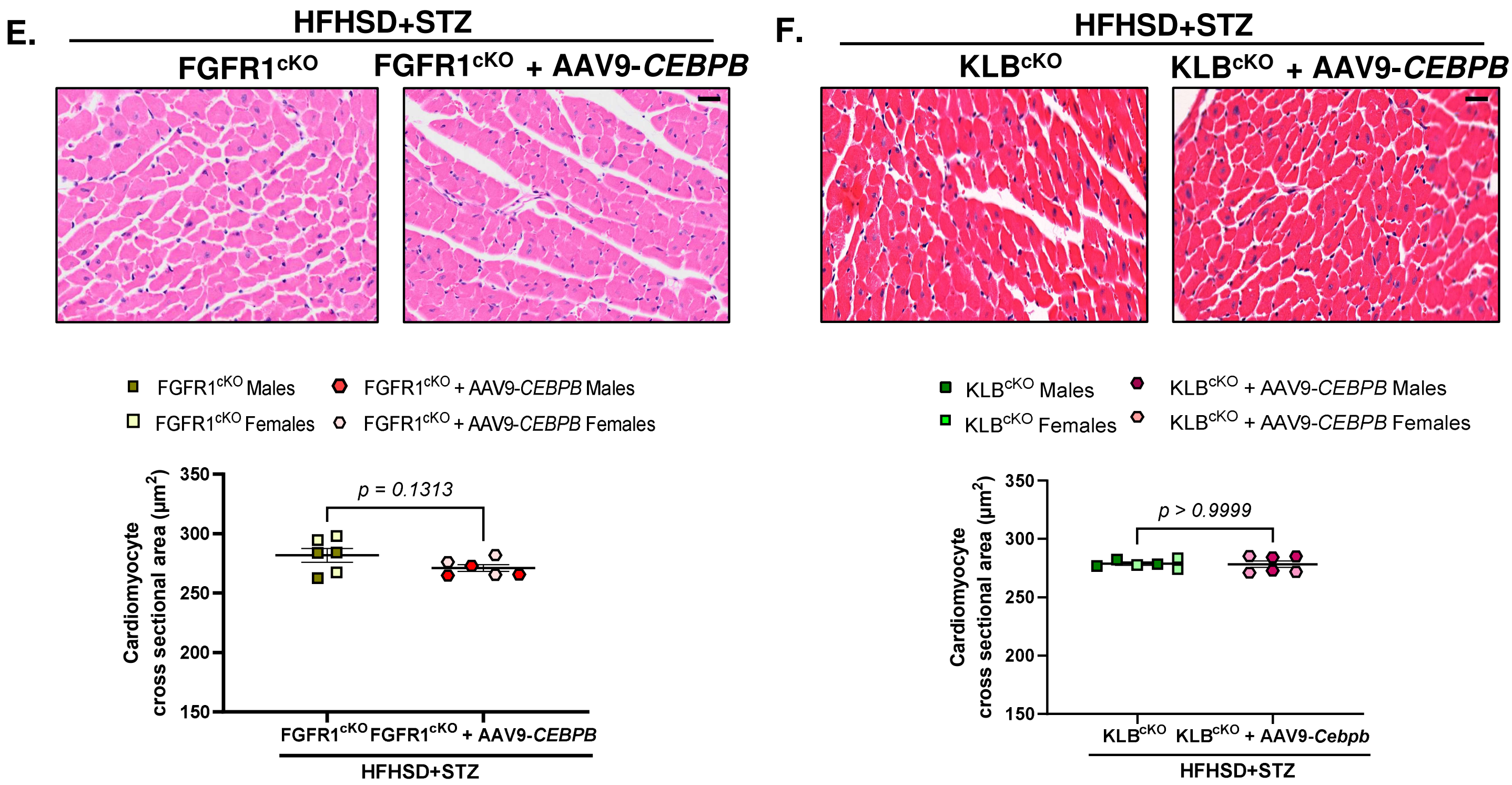


**Supplemental Figure 19. AAV9-*CEBPB* treatment mitigates pathological remodeling in FGFR1$^{cKO}$ and KLB$^{cKO}$ mice under HFHSD+STZ stress.**

**A-B,** Quantitative PCR analyses of cardiac pathological remodeling markers (*Acta1*, *Nppb*, *Col1a2*, *Col3a1*, *Tgfb*) and angiogenesis-associated genes (*Cdh5*, *Vegfa*) in (**A**) FGFR1$^{cKO}$ hearts and (**B**) KLB$^{cKO}$ mice with or without adeno-associated virus serotype 9-CCAAT/enhancer-binding protein beta (AAV9-*CEBPB*) treatment under high-fat, high-sucrose diet and streptozotocin (HFHSD+STZ) stress (N=6 mice of both sexes/chow-fed group and N=7-8 mice of both sexes/HFHSD+STZ-fed group). **C-D,** TUNEL-stained heart sections showing TUNEL-positive nuclei (red; white arrows) and quantification graphs in (**C**) FGFR1$^{cKO}$ and (**D**) KLB$^{cKO}$ mice treated with AAV9-*CEBPB* under HFHSD+STZ stress (N=6 mice of both sexes/chow-fed group and N=7-8 mice of both sexes/HFHSD+STZ-fed group; scale=50 μm). DAPI is shown in blue, and green represents α-actinin. **E-F,** Representative H&E-stained cardiac cross-sections and corresponding quantification of cardiomyocyte size in (**E**) FGFR1$^{cKO}$ and (**F**) KLB$^{cKO}$ mice treated with AAV9-*CEBPB* (N=6 hearts from both sexes/group; scale=20 μm). Data are presented as mean±SEM. Statistical comparisons (indicated in each panel were performed using the Mann-Whitney U test (**A-*Tgfb*, F**) and 2-tailed unpaired Student *t*-test (**A-other genes, B-E**).

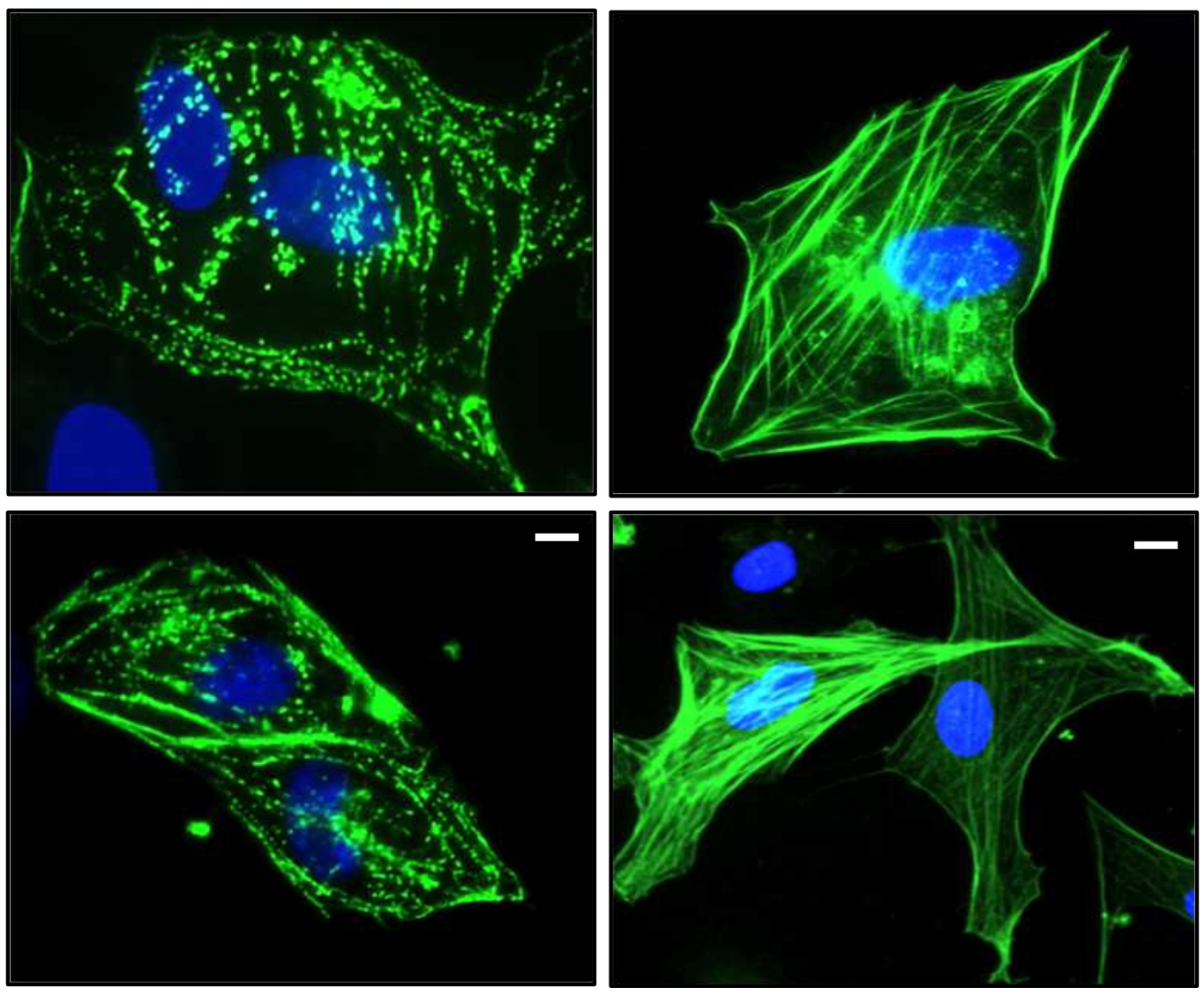


**Supplemental Figure 20**. **Characterization of hiPSC-cardiomyocytes.**

Representative immunofluorescence images showing cardiomyocyte markers α-actinin (left, green) and cTnT (cardiac troponin T; right, green) in human induced pluripotent stem cells derived cardiomyocytes (hiPSC-cardiomyocytes). Characterization was performed after each differentiation; scale=50 μm.

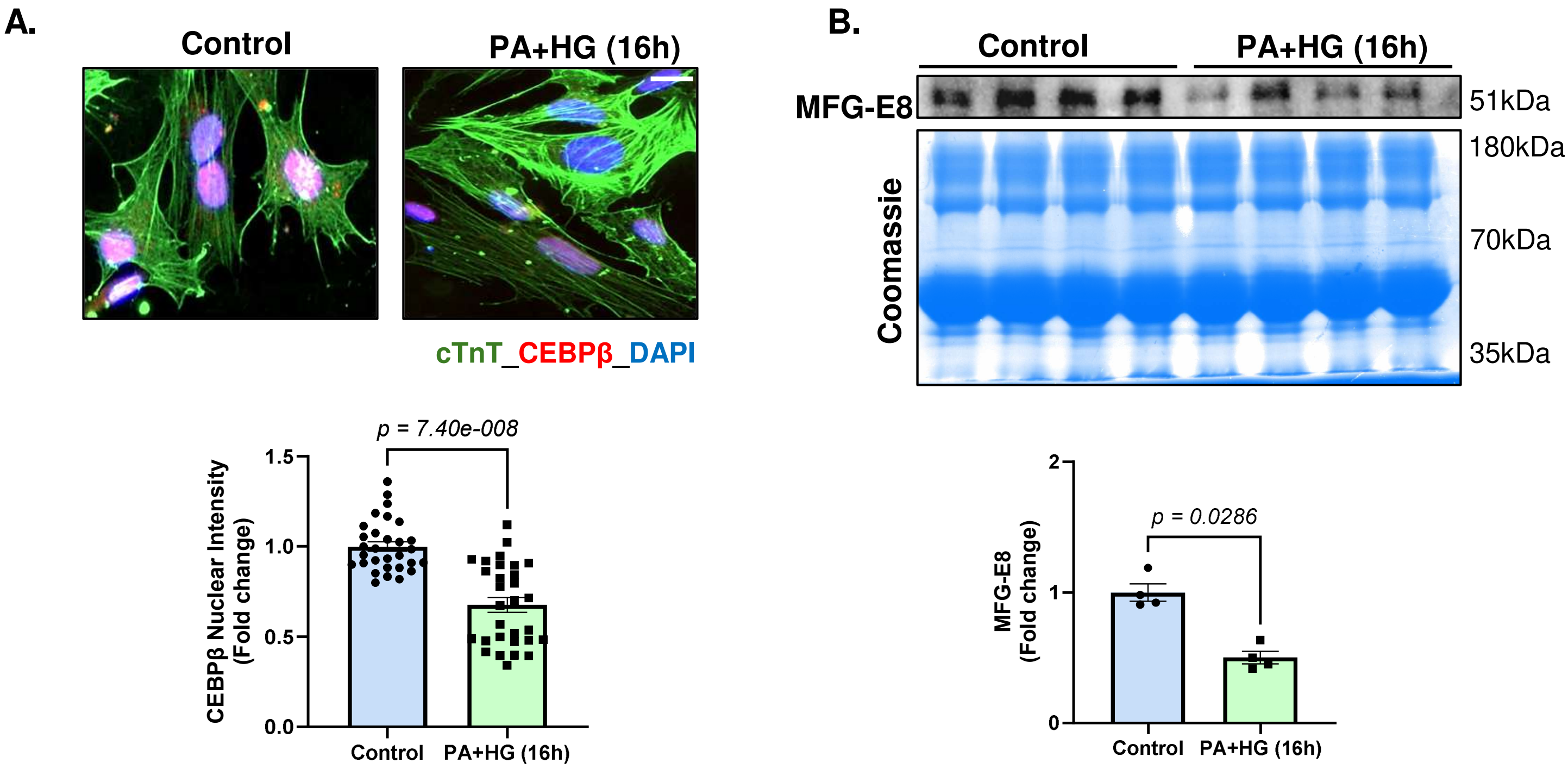


**Supplemental Figure 21**. **Reproducibility of CEBPβ-MFG-E8 responses in hESC-cardiomyocytes.**

**A,** Immunofluorescent co-staining of cTnT (green), CEBPβ (CCAAT/enhancer-binding protein beta; red), and DAPI (blue) in human embryonic stem cells-derived cardiomyocytes (hESC-cardiomyocytes) under prolonged palmitic acid (PA; 500 µmol/L) and high glucose (HG, 25 mmol/L) stress with corresponding quantification of nuclear CEBPβ intensity compared to controls (N=30 cells from 3 independent biological replicates; scale=20 µm). **B,** Representative immunoblots and quantification of secreted MFG-E8 in the media culturing hESC-cardiomyoctyes under PA+HG stress (N=4 biological replicates/group). Coomassie blue staining assessed total protein as a loading control. Data are presented as mean±SEM. Statistical significance (indicated in the panel) was assessed using the Mann-Whitney U test (**A-B**).

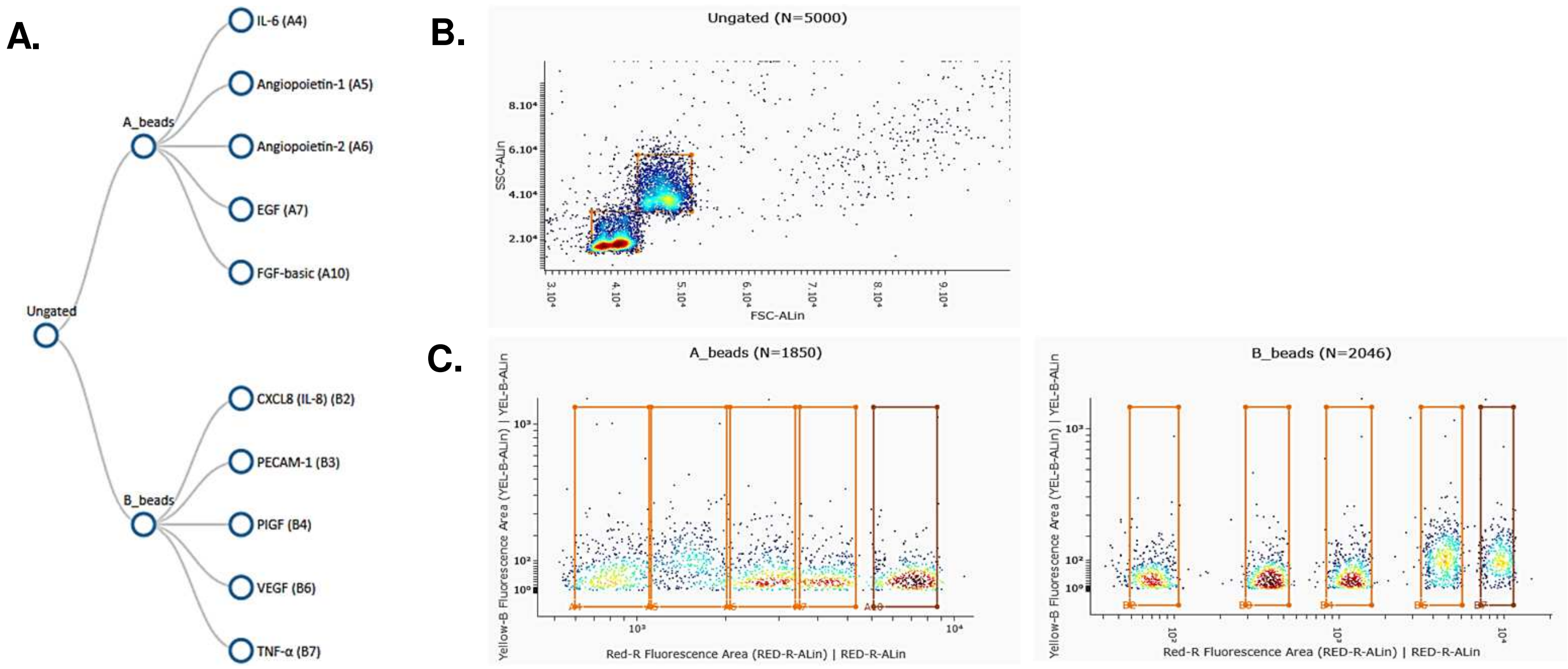


**Supplemental Figure 22. Analyte classification and gating strategy for LEGENDplex™ human angiogenesis panel.**

**A,** Schematic representation of the analyte-specific bead subsets for the LEGENDplex™ Human Angiogenesis Panel. Beads are divided into Beads A and Beads B, each encoding five unique analytes based on internal dye signatures. **B,** Forward scatter (FSC-A) vs. side scatter (SSC-A) plot showing initial gating to isolate bead populations from debris and noise (Ungated, N=5000). **C,** Bead populations were further separated into A Beads (left) and B Beads (right) based on their distinct Red-R vs. Yellow-B fluorescence profiles, corresponding to analyte-specific bead subsets. A beads (N=1850) and B beads (N=2046) were individually gated. Each vertical gate denotes a unique analyte population. Quantification was performed based on PE signal intensity for each analyte (N=6 biological replicates/group).

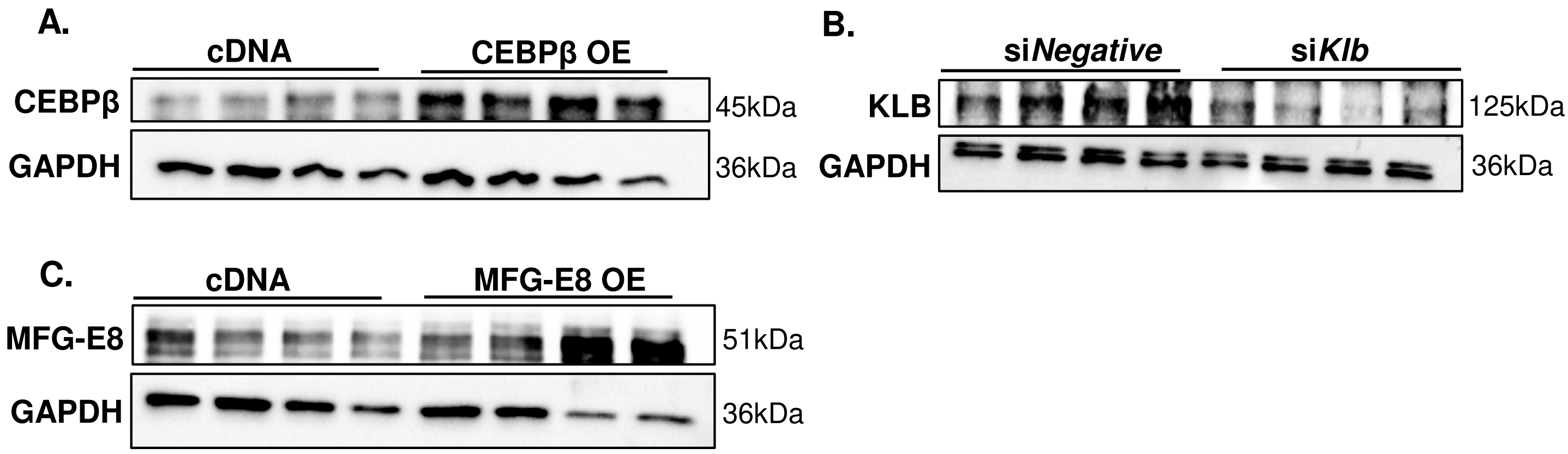


**Supplemental Figure 23**. **Validation of various gene manipulation on hiPSC-cardiomyocytes.**

**A,** Representative immunoblots validating overexpression of CEBPβ in human induced pluripotent stem cell-derived cardiomyocytes (hiPSC-cardiomyocytes). GAPDH was used as the loading control. **B,** Representative immunoblots validating si*RNA*-mediated knockdown of KLB (si*Klb*) in hiPSC-cardiomyocytes. GAPDH was used as the loading control **C,** Representative immunoblots validating overexpression of MFG-E8 in hiPSC-cardiomyocytes. GAPDH was used as the loading control. All immunoblots were performed using 4 biological replicates.
